# Statistical inference of keystone taxa reshaping the assembly rules of forest root microbiomes

**DOI:** 10.1038/s41467-026-78401-3

**Published:** 2026-10-28

**Authors:** Mikihito Noguchi, Kenta Suzuki, Hiroaki Fujita, Hirokazu Toju

**Affiliations:** 1 Center for Ecological Research, Kyoto University, Otsu, Japan; 2 Laboratory of Ecosystems and Coevolution, Graduate School of Biostudies, Kyoto University, Kyoto, Japan; 3 Environmental Management Research Institute, National Institute of Advanced Industrial Science and Technology (AIST), Tsukuba Center, Tsukuba, Japan; 4 Integrated Bioresource Information Division, BioResource Research Center, RIKEN, Tsukuba, Japan; 5 Institute for Multidisciplinary Sciences, Yokohama National University, Yokohama, Japan; 6 Center for Living Systems Information Science (CeLiSIS), Graduate School of Biostudies, Kyoto University, Kyoto, Japan

**Keywords:** Community ecology, Computational science, Microbiome, Computational biology and bioinformatics

## Abstract

Root-associated fungi and bacteria play pivotal roles in terrestrial ecosystem processes. However, identifying keystone taxa driving below-ground microbial assembly remains a major challenge. Here, we present a statistical framework for identifying microbes that reorganize microbiome assembly. Using 1,270 root samples collected from a cool-temperate forest in Japan, we reconstruct assembly landscapes describing the probabilistic structure of community states. We then find that several endophytic fungi (e.g., *Phialocephala* and *Oidiodendron*) are strongly associated with the reorganization of assembly landscape topography along abundance gradients, coinciding with shifts in mycorrhizal symbiosis. Independent datasets from Europe confirm that the highlighted genera are significantly associated with changes in mycorrhizal colonization patterns. Prokaryotic communities exhibit transitions among states characterized by core taxa, Pseudomonadota, or Actinomycetota, strongly associated with abundance gradients of *Candidatus Udaeobacter* and several endophytic fungi. These findings provide a scalable framework for prioritizing candidate keystone taxa and linking microbiome assembly to ecosystem-scale processes.

## Abstract (web summary)

Root microbiomes shape terrestrial ecosystems, yet identifying keystone microbes remains difficult. By statistically inferring assembly rules, the authors reveal endophytic fungi as hidden drivers reorganizing below-ground symbioses.

## Introduction

Fungi and bacteria associated with plant roots support host survival, nutrient acquisition, and ecosystem functioning in terrestrial environments^1–4^. Numerous studies have suggested that such plant microbiomes may harbor keystone taxa exerting strong influences on community structure and ecosystem processes^5–10^. However, identifying such taxa remains difficult under traditional definitions of keystone taxa, which emphasize disproportionate impacts relative to abundance and typically require targeted perturbations of specific taxa for empirical validation^9,10^. Many plant-associated microbes depend strongly on host-derived carbon and are therefore difficult to examine using culture-based experiments^11^. Even when culturing is feasible, demonstrating ecosystem-scale impacts would generally require large spatial-scale manipulations conducted over long temporal scales. Conducting such experiments for thousands of microbial taxa present in natural communities is therefore impractical. Consequently, data-driven analytical frameworks that translate keystone concepts into operational criteria for inferring microbial roles from observational data are essential for advancing our understanding of below-ground ecosystem processes.

**Fig. 1:**
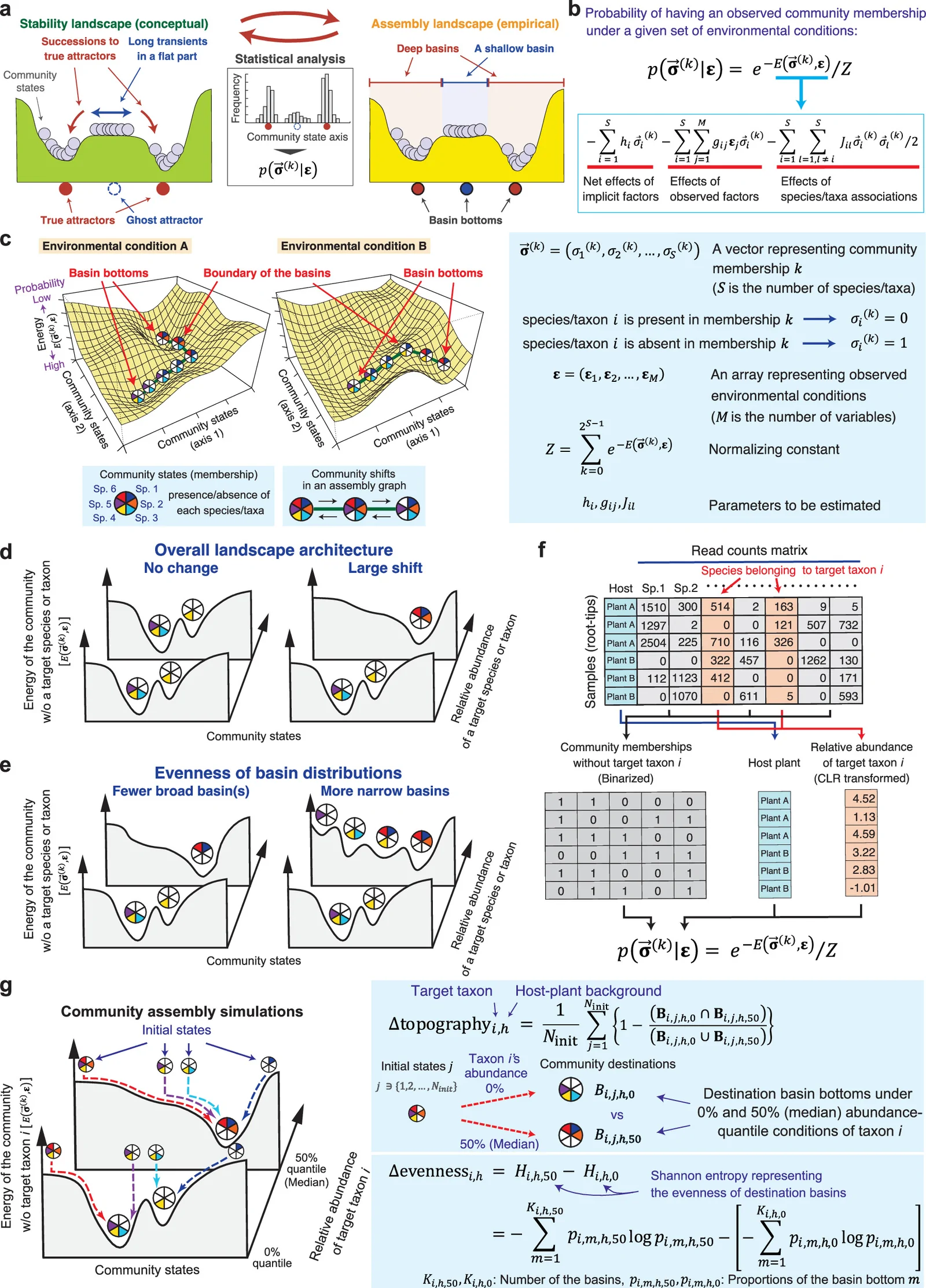
Landscape representation of community assembly. **a** Stability landscape concept and empirically reconstructed assembly landscapes. The stability landscape conceptually represents community structure–stability relationships (left), whereas the assembly landscape, inferred from empirical data, statistically describes them as community state–observation probability relationships (right). On a stability landscape, community assembly converges toward true attractors or exhibits prolonged transients (e.g., ghost attractors), both appearing as clusters of observations. True attractors are represented as deep basins on the assembly landscape; however, prolonged transients may also be detected as shallow basins. **b**, **c** Energy landscape analysis. Based on the Ising model, the probability of observing a given community state (membership) can be expressed as a linear combination of effects from implicit factors, observed factors, and dependencies among species/taxa (**b**). The lower the resulting energy metric, the higher the probability of the corresponding state. The topography of the assembly landscape varies with background environmental conditions (**c**). Community assembly is assumed to proceed toward basin bottoms within the assembly graphs, where states differing by a single species are connected by a transition. **d**, **e** Exploring keystones. Given that assembly landscapes reflect community assembly rules, keystone species/taxa can be operationally defined as those whose abundance changes induce substantial shifts in landscape architecture. This reorganization is quantified as changes in basin-bottom composition and distribution (Δtopography; **d**) and in basin-distribution evenness (Δevenness; **e**) along a focal species/taxon’s abundance gradient. **f**, **g** Calculating the keystone metrics. The abundance of a focal species is extracted from the community data matrix and used as an environmental factor in energy landscape analysis (**f**). Assembly landscape architecture inferred in the absence of the focal species/taxon is compared with that under an intermediate abundance level (the median abundance among samples where the species/taxon was present; **g**). Across 20,000 community assembly simulations on the inferred landscapes, the mean Jaccard dissimilarity between corresponding destinations (basin bottoms; Δtopography) and the difference in Shannon entropy of basin frequencies (Δevenness) were calculated. Both indices were standardized and tested based on randomized null simulations, and species/taxa with large, significant Δtopography and/or Δevenness values are highlighted as keystone candidates.

In exploring factors that shape community-scale organization, the well-established concept of stability landscapes provides useful conceptual insights^12–16^. In this framework, community composition is envisioned as a ball moving across a landscape of valleys and hills, where valleys (basins) represent stable states and hilltops define the boundaries between them^12,17^ (Fig. 1a-left). The bottoms of these valleys are interpreted as alternative stable states toward which community structures tend to converge from nearby configurations^18,19^. Large shifts in community states are often interpreted as stochastic transitions between neighboring basins^17,19^, whereas major shifts may also occur when the landscape topography itself is reorganized^18,19^. Factors altering the number or positions of basins are therefore central to understanding community-scale dynamics. Importantly, such factors are not necessarily abiotic. Because species interactions contribute to the constraints governing community assembly (assembly rules), changes in the abundance of particular taxa (or species) that strongly influence other community members can reshape stability landscape topography. Therefore, quantifying such reorganizations of landscape architecture, reflected in changes in the number and configuration of basins, provides a statistical basis for evaluating the extent to which a taxon reorganizes the rules underlying community assembly.

Here, we present a statistical framework designed to highlight species or taxa potentially capable of reorganizing microbiome assembly processes. Although directly reconstructing stability landscapes from empirical data remains challenging^20,21^, statistical-physics-inspired approaches enable reconstruction of landscapes describing community-state probability distributions^14,15,22,23^. We refer to these empirically derived representations as assembly landscapes (Fig. 1a-right). Basins identified on such landscapes do not necessarily correspond directly to theoretical alternative stable states because empirical observations are incomplete, may include observational noise, and typically require dimensional reduction to render the immense state space computationally tractable^14,15,22–24^. Nevertheless, the assembly-landscape approach provides a practical means of linking community assembly rules with environmental and biotic contexts^14^. This framework offers a systematic basis for identifying taxa potentially exerting community-wide influences. Thus, we ask whether changes in the abundance of particular microbes reshape the overall architecture of microbiome assembly landscapes and whether such changes can systematically identify candidate keystone taxa across diverse microbial communities.

In this work, we apply this empirical research framework to a large dataset of root-associated microbiomes collected in a cool-temperate forest comprising host plants with contrasting mycorrhizal types. Specifically, we reconstruct assembly landscapes for fungal and prokaryotic communities associated with ectomycorrhizal plants (*Betula*, *Pinus*, and *Larix*) and with plants capable of forming both arbuscular- and ectomycorrhizal symbioses (hereafter, dual-mycorrhizal plants^25^; *Populus*, *Acer*, and *Juglans*). Basins of community assembly are identified for each host-plant background, and sensitivity analyses are conducted to evaluate how assembly landscape topography varies along abundance gradients of individual microbial taxa. This systematic exploration highlights microbes strongly associated with community-scale transitions, including shifts between ectomycorrhizal and arbuscular mycorrhizal symbiotic states in dual-mycorrhizal plants. To evaluate ecological relevance, we further analyze independent root microbiome datasets spanning European forest ecosystems. Overall, the proposed statistical framework provides a general platform for prioritizing candidate keystone taxa across diverse microbial (and macro-organismal) communities.

## Results

### Root microbiome structure

**Fig. 2:**
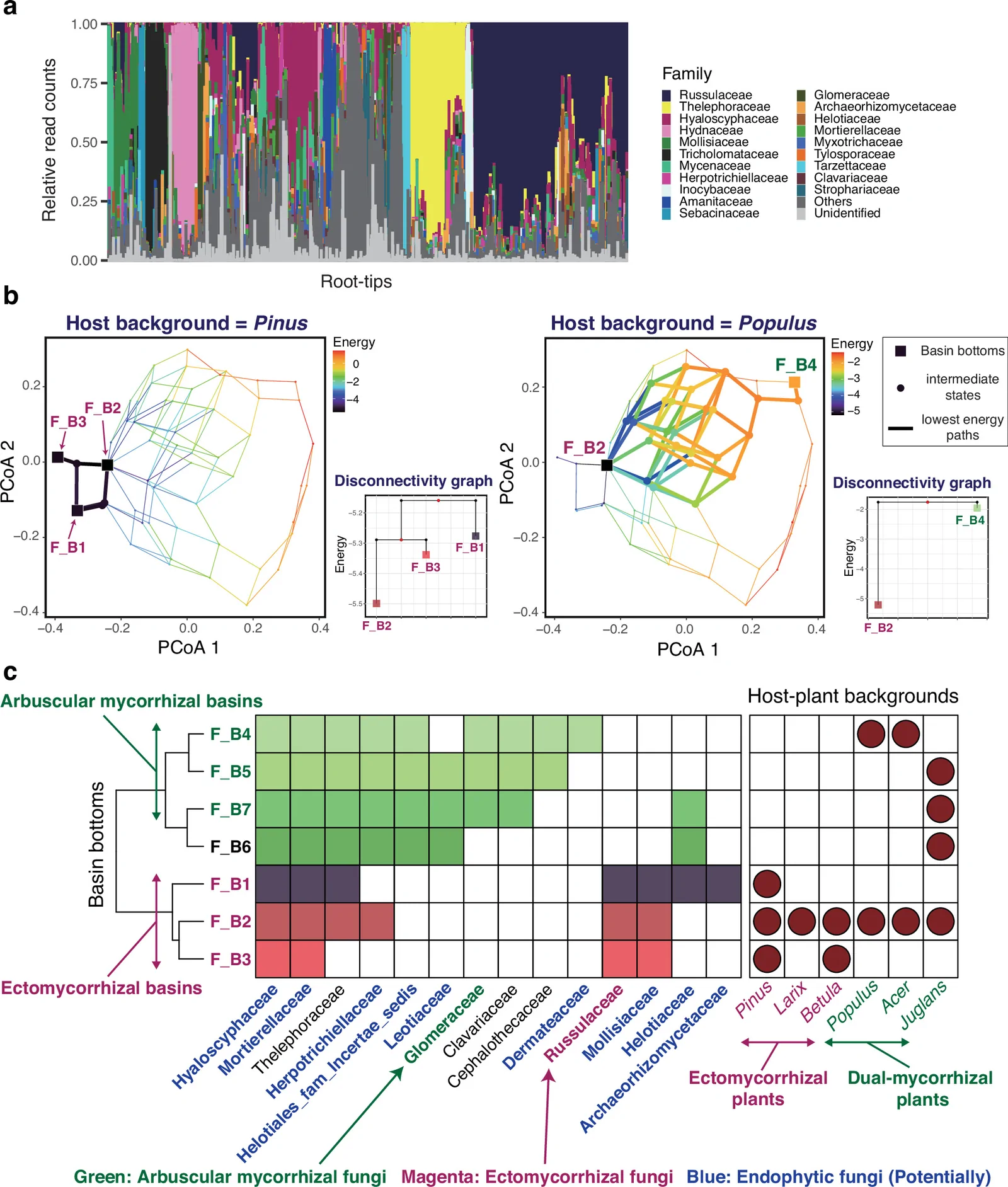
Assembly landscape analysis of the root fungal community. **a** Family-level taxonomic compositions of fungi across 1270 root-tip samples. **b** Assembly landscapes reconstructed under different host-plant backgrounds. Energy landscape analysis was applied to binarized family-level fungal community data, with host-plant identity included as an explicit environmental variable (Fig. 1b). Basin bottoms (squares), intermediate states (circles), and lowest-energy transition pathways (thick lines) are visualized on a principal coordinate analysis (PCoA) surface representing community-state relationships. Pathways and intermediate states associated with basins inferred under other host backgrounds are shown as thin lines and small circles. Disconnectivity graphs illustrate relative basin depths in the reconstructed assembly landscapes. Landscapes reconstructed under other host backgrounds are shown in Supplementary Fig. S15. **c** Community composition of basin bottoms. The seven basin bottoms identified across six host-plant backgrounds are shown with a dendrogram representing similarity in taxonomic membership. Depending on the presence or absence of Glomeraceae and Russulaceae, basins are tentatively categorized as arbuscular mycorrhizal or ectomycorrhizal. The repertoire of basins detected under each host-plant background is shown in the right panel. Source data are provided as a Source data file.

**Fig. 3:**
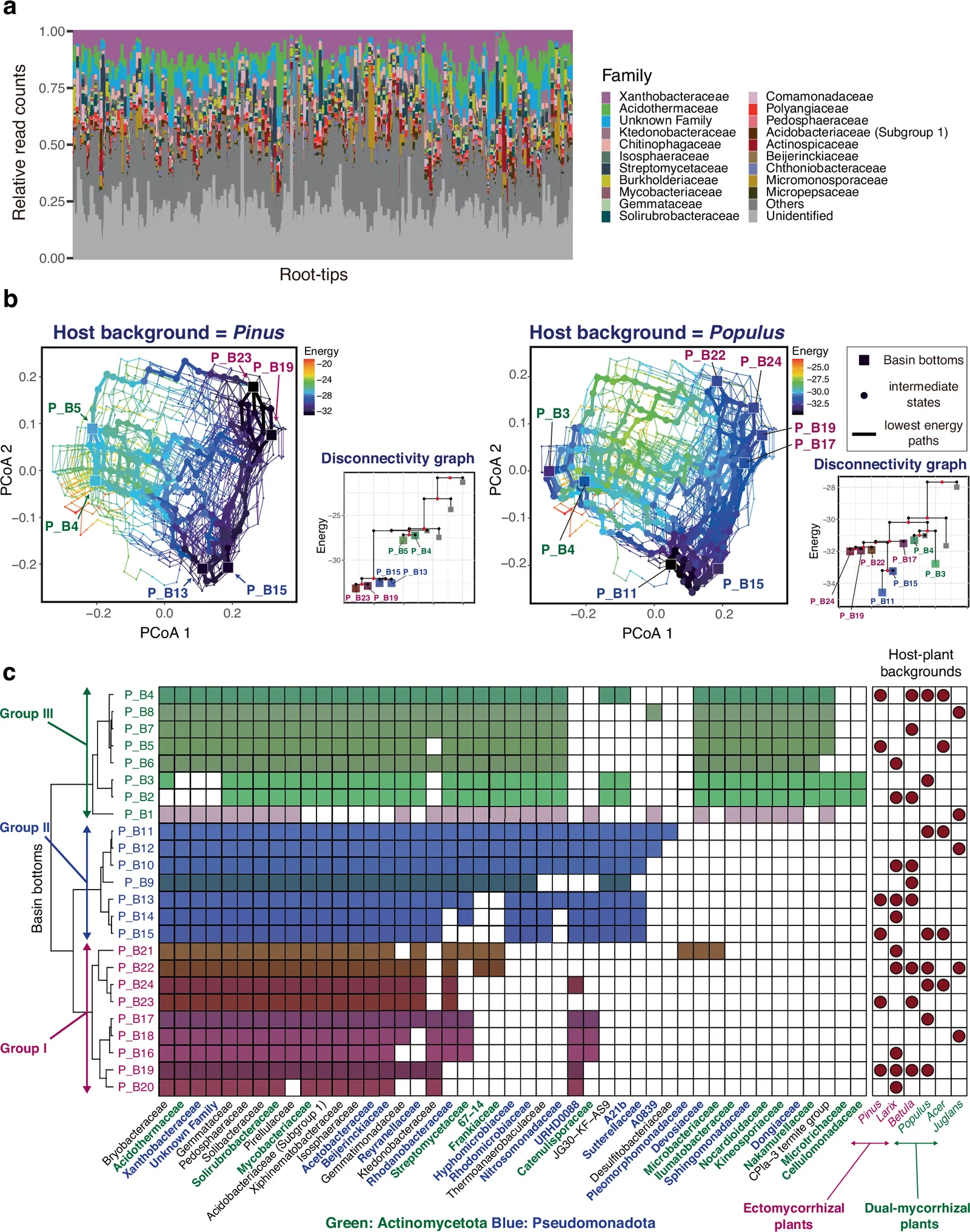
Assembly landscape analysis of the root prokaryote community. **a** Family-level taxonomic compositions of prokaryotes across 1270 root-tip samples. **b** Assembly landscapes reconstructed under different host-plant backgrounds. Energy landscape analysis was applied to binarized family-level taxonomic composition data of root-associated prokaryotes, with host-plant identity included as an explicit environmental variable in the statistical model (Fig. 1b). Basin bottoms (squares), intermediate states (circles), and lowest-energy transition pathways between them (thick lines) are visualized on a principal coordinate analysis (PCoA) surface representing community-state relationships. Pathways and intermediate states associated with basins inferred under other host-plant backgrounds are shown as thin lines and small circles. Disconnectivity graphs illustrate the relative depths of basins within the reconstructed assembly landscapes. Landscapes reconstructed under other host-plant backgrounds are shown in Supplementary Fig. S16. **c** Community composition of basin bottoms. The 24 basin bottoms identified across the six host-plant backgrounds are shown together with a dendrogram representing similarity in taxonomic membership. The basins are tentatively grouped into three categories: Group I, consisting mainly of commonly observed taxa; Group II, characterized by additional Pseudomonadota families; and Group III, characterized by additional Actinomycetota families. The repertoire of basin bottoms detected under each host-plant background is shown in the right panel. Source data are provided as a Source data file.

To obtain a large dataset for exploring key drivers in high-dimensional microbiome dynamics, we conducted fieldwork in a cool-temperate forest in central Japan^26^. The forest consisted mainly of ectomycorrhizal trees **(***Betula platyphylla*, *Pinus densiflora*, and *Larix kaempferi*) and trees capable of forming both arbuscular- and ectomycorrhizal symbioses^26^ (dual-mycorrhizal; *Populus tremula*, *Acer rufinerve*, and *Juglans mandshurica*). After surface sterilization of the root samples, both the fungal ITS and prokaryotic 16S rRNA sequence data were obtained for 1270 root-tip samples (Supplementary Figs. S1–S3). In total, the microbiome dataset consisted of 2249 fungal operational taxonomic units (OTUs) representing 524 genera and 249 families and 12,479 prokaryotic OTUs representing 590 genera and 309 families (Figs. 2a and 3a and Supplementary Figs. S1–S2). In the fungal community data, the ectomycorrhizal genera *Russula* (Russulaceae) and *Tomentella* (Thelephoraceae) were the most abundant, followed by a possibly endophytic genus *Hyaloscypha* (Hyaloscyphaceae) (Fig. 2a and Supplementary Fig. S2a, b). The prokaryotic data lacked dominant taxa at the genus or family levels (Fig. 3a and Supplementary Fig. S2c, d). The fungal and prokaryotic taxa showed varying degrees of preferences for hosts (Supplementary Figs. S4–S5).

### Assembly landscapes enable keystone exploration

To characterize the organization of microbiome assembly, we applied a statistical framework inspired by the stability-landscape concept^12,16,17^ (Fig. 1b). This approach reconstructs a probabilistic representation of community states, hereafter referred to as an assembly landscape, based on model-implied probabilities derived from an Ising-model framework in statistical physics^14,27,28^. In this context, community states are represented in binary format (presence or absence of taxa), following the assembly-graph concept in community ecology^29–31^. For a system containing *S* taxa, the corresponding state space consists of 2^*S*^ possible states. The probability of each community state was estimated as a function of background environmental conditions and interspecific (inter-taxon) associations. The resulting assembly landscape provides a structured view of how community states are distributed in probability space depending on environmental contexts (Fig. 1b, c).

Based on this framework, we developed a sensitivity-analysis approach to identify candidate keystone taxa associated with assembly landscape reorganization (Fig. 1d–g; see Methods for details). The central idea is to quantify how landscape architecture changes along the abundance gradient of each focal species or taxon. If the number or positions of basins vary markedly across abundance levels, the focal taxon is considered strongly associated with shifts in assembly processes (Fig. 1d, e). In this analysis, the relative abundance of the focal taxon is treated as an external variable influencing the modeled probabilities of community states (i.e., assembly landscape architecture) for the remaining community members (Fig. 1f, g).

By quantifying these patterns of landscape reorganization, we introduced two complementary indices of operational keystoneness. The first index, Δtopograph*y*, measures changes in topographical attributes of the assembly landscape, including the number, positions, and relative sizes of basins (Fig. 1d). The second index, Δevenness, captures changes in the evenness of the probability distribution of community states along the abundance gradient of the focal taxon (Fig. 1e). A positive Δevenness value is associated with redistribution toward many narrower basins, implying the focal taxon’s destabilizing effects on community assembly. In contrast, a negative Δevenness value indicates assembly landscapes with fewer but broader basins, implying increased dominance of major community states. Sequential evaluation of these indices across taxa enabled systematic identification of candidate keystone organisms associated with the reorganization of assembly rules (Fig. 1f, g).

Before applying this framework to empirical microbiome data, we conducted in silico benchmark analyses based on generalized Lotka–Volterra models incorporating diverse types of microbial interactions (Supplementary Fig. S6). These simulations indicated that the proposed approach reproduced key features of multispecies community dynamics relevant to keystone-associated landscape reorganization (Supplementary Figs. S7–S10; see Supplementary Notes 1–3 for details).

### Distinct basin groups recur in assembly landscapes

To explore potential keystone taxa of the forest root microbiome, we first characterized the overall architecture of assembly landscapes. Specifically, the fungal and prokaryotic datasets were separately subjected to assembly landscape analysis, in which host-plant identity was included as a categorical (dummy) variable (see Methods for details). To focus on substantial events in community assembly while maintaining computational feasibility in exploring assembly graphs (2^*S*^ possible community states in systems with *S* taxa), community states were defined by the family-level taxonomic compositions of statistically selected 34 fungal and 45 prokaryotic taxa (Supplementary Figs. S11–S13; see Supplementary Note 4 for details).

The reconstructed assembly landscapes of the root-associated fungal community comprised seven basins differing in the properties of mycorrhizal symbiosis (Fig. 2b, c and Supplementary Figs. S14a, b and S15). Specifically, three basins (basin IDs, F_B1–B3) were characterized by the presence of a dominant ectomycorrhizal fungal family, Russulaceae, together with the endophytic–saprotrophic family *Mollisiaceae* (Fig. 2c). These basins varied in their compositions of other ectomycorrhizal–saprotrophic (Thelephoraceae) and endophytic (Herpotrichiellaceae, Helotiaceae, and Archaeorhizomycetaceae) fungal families (Fig. 2c). In contrast, the basins F_B4, F_B5, and F_B7 were characterized by the arbuscular mycorrhizal family Glomeraceae and the saprotrophic family Clavariaceae (Fig. 2c). In addition to the ectomycorrhizal and arbuscular-mycorrhizal basins described above, one basin with diverse endophytic–saprotrophic families was identified (F_B6).

The topography of the fungal assembly landscape varied among host plants, suggesting that assembly rules differ depending on host ecological and physiological traits. When the ectomycorrhizal plant *Pinus* was assumed in the statistical model, three basins associated with ectomycorrhizal symbiosis (F_B1–B3) were identified (Fig. 2b-left; Fig. 2c). Visualization of the relationships among the three basins in a disconnectivity graph^14^ indicated that the basin F_B2 was the most probable, corresponding to the lowest energy score (Fig. 2b). When *Betula* was assumed as the host background, two basins with comparable energy scores were characterized, whereas for *Larix*, only a single basin was identified (Supplementary Fig. S15).

In contrast to the assembly landscapes reconstructed for ectomycorrhizal host backgrounds, those for dual-mycorrhizal host plants (*Acer*, *Populus*, and *Juglans*) included both ectomycorrhizal (F_B1–B3) and arbuscular-mycorrhizal basins (F_B4, F_B5, and F_B7) (Fig. 2b, c and Supplementary Fig. S15). These results suggest that, in the root systems of dual-mycorrhizal plants, community assembly can proceed toward either arbuscular-mycorrhizal symbiosis or distinct root states dominated by ectomycorrhizal fungi, representing alternative assembly outcomes.

In the prokaryotic dataset, a total of 24 major basins were identified across the six host-plant backgrounds, each representing more than 1% of the samples (Fig. 3b, c and Supplementary Figs. S14c, d and S16). These basins were broadly grouped into three categories based on their taxonomic profiles. Group I (P_B16–B24) comprised bacterial families commonly detected in root samples, whereas Groups II (P_B9–B15) and III (P_B1–B8) were characterized by additional *Pseudomonadota* and *Actinomycetota* families, respectively. These three basin groups appeared consistently across all six host-plant backgrounds (Fig. 3c). Thus, the assembly of root-associated prokaryotes on each host plant can be broadly described as three alternative community states differing primarily in their *Pseudomonadota* and *Actinomycetota* repertoires.

To evaluate how basin structures inferred from the assembly landscape framework relate to observed community composition, we performed principal coordinate analysis (PCoA) based on family-level dissimilarities (Supplementary Fig. S14). Differences in abundance-based community composition associated with basin identity were statistically detectable (PerMANOVA; Supplementary Table S1). However, the separations in ordination space were not visually pronounced (Supplementary Fig. S14a, c), likely because several basin-indicative families occurred at low relative abundances, including Glomeraceae (arbuscular mycorrhizal fungi; relative abundance = 2.01 × 10^−2^) and Nocardioidaceae (Actinomycetota bacteria; relative abundance = 5.87 × 10^−4^; see Figs. 2c and 3c), thereby contributing little to abundance-weighted dissimilarity patterns. In contrast, clearer separations were observed under Jaccard dissimilarities based on presence–absence profiles (Supplementary Fig. S14b, d and Supplementary Table S1), as expected from the presence–absence definition of community states in the assembly landscape analysis. Thus, this comparison helps clarify that the inferred basin structures primarily reflect differences in taxon membership, including low-abundance but ecologically informative families, rather than abundance variation among dominant taxa.

### Endophytic fungi emerge as candidate keystones

We then explored potential keystone taxa by calculating the Δtopography and Δevenness indices defined above (Fig. 1d–g). Because it was impractical to apply the computationally intensive approach to numerous microbial OTUs, we focused on genus-level ecological properties.

**Fig. 4:**
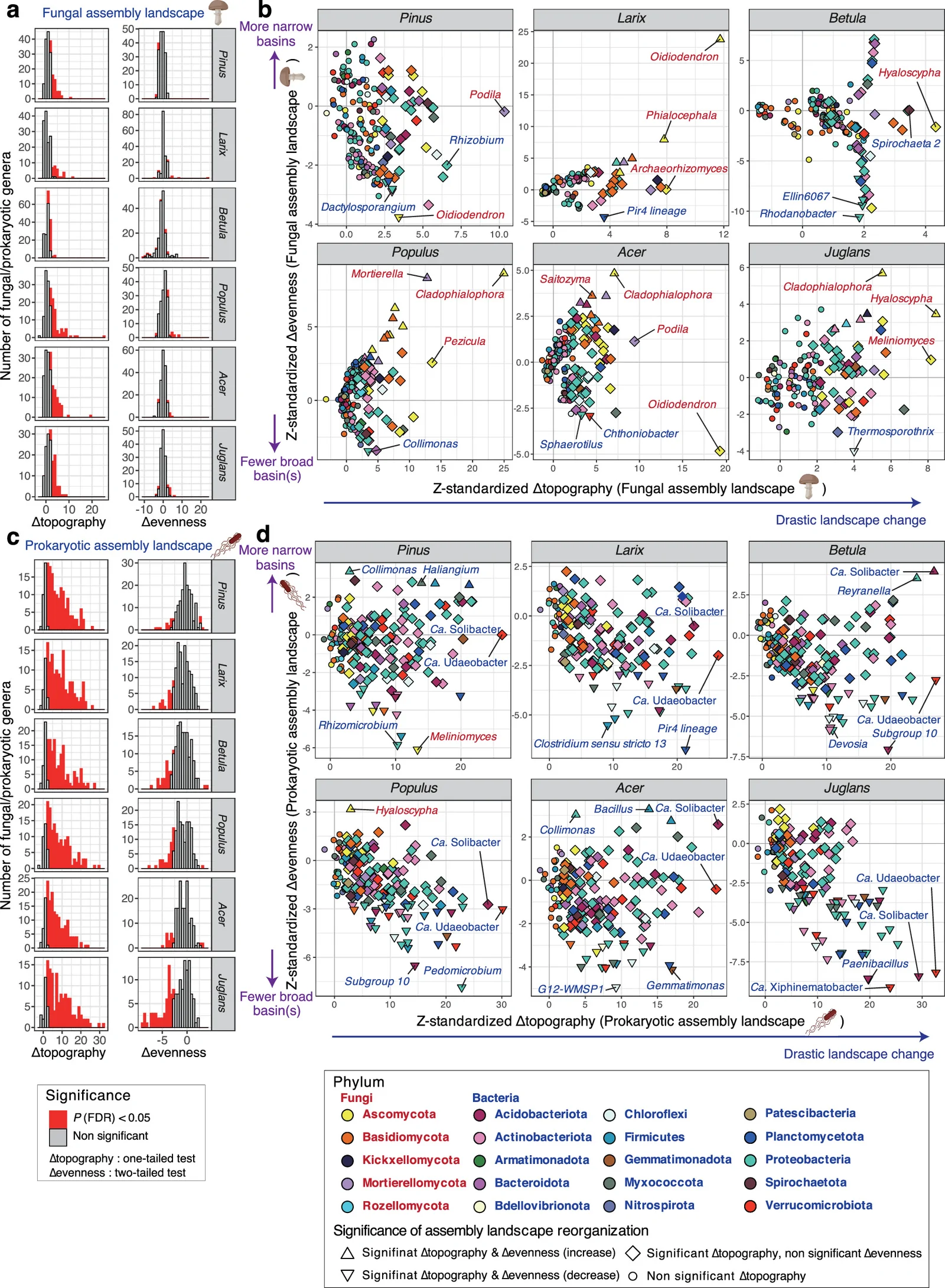
Highlighting candidate keystone taxa. **a** Histograms of potential impacts on fungal community assembly. The Δtopography and Δevenness indices respectively quantify changes in the overall topography and evenness of assembly landscape architecture along the abundance gradient of a focal genus (Fig. 1d–g). *Z*-standardized values and empirical *P* values of Δtopography and Δevenness were calculated based on randomization analyses in which the abundance of the focal genus was shuffled within root samples of the same host-plant background (10,000 permutations). *P* values were adjusted using the Benjamini–Hochberg procedure. Genera that had significant indices are shown in red (one-sided test for Δtopography; two-sided test for Δevenness). Results are shown separately for each host-plant background. **b** Taxa showing strong potential impacts on fungal assembly landscapes. On the two-dimensional surface defined by Δtopography and Δevenness, microbial genera whose abundance changes were inferred to substantially reorganize the assembly landscape architecture of root-associated fungal communities are indicated. **c** Histograms of potential impacts on prokaryotic community assembly. **d** Taxa showing strong potential impacts on prokaryotic assembly landscapes. Source data are provided as a Source data file. Fungal illustrations (**a**, **b**): Created in BioRender. Toju, H. (2026) https://BioRender.com/2rdd0th. Bacterial illustrations (**c**, **d**): Created in BioRender. Toju, H. (2026) https://BioRender.com/w6b4bi9.

We found that diverse fungal and bacterial genera were significantly associated with changes in the assembly landscape topography of fungal communities (Fig. 4a and Supplementary Table S2). In total, 30 of 33 fungal genera (90.9%) and 91 of 143 prokaryotic genera (63.6%) exhibited significant Δtopography values [false discovery rate (*q-*value) < 0.05; one-tailed test] in at least one host-plant background, indicating their potential to reshape the overall architecture of the assembly landscapes. Meanwhile, 19 fungal genera (57.6%) and 29 prokaryotic genera (20.3%) showed significant Δevenness (*q-*value < 0.05; two-tailed test), suggesting their influence on higher-order properties of fungal community assembly, such as the evenness of basin frequencies. Although taxon abundance and/or occupancy were significantly associated with a subset of landscape metrics across genera (Kendall’s rank correlation test; Supplementary Tables S3–S4), many genera highlighted as having large effects showed intermediate, rather than particularly high, abundance and occupancy (Supplementary Figs. S17–S20). These results indicate that abundance and occupancy alone do not determine which taxa contribute the largest effects.

Among the examined microbes, several genera of root endophytic fungi were significantly associated with both the overall architecture and higher-order properties of fungal assembly landscapes (Fig. 4b). For instance, *Oidiodendron* (Ascomycota; Helotiales), known as a dark septate endophyte^32,33^ taxon, exhibited the strongest influence on fungal community assembly within the root systems of *Larix* and *Acer*. This taxon showed patterns consistent with destabilization of community processes in *Larix* roots while exhibiting stabilizing tendencies in *Pinus* roots. Another dark septate endophytic genus, *Cladophialophora*^33^ (Ascomycota; Chaetothyriales), showed greatest impacts on fungal assembly landscapes within the root systems of the three dual-mycorrhizal plant species examined (*Acer*, *Juglans*, and *Populus*; Fig. 4b). This taxon consistently exhibited destabilizing influence on community assembly across the host-plant backgrounds examined. Some other endophytic genera in the Ascomycota order Helotiales, such as *Phialocephala*, *Hyaloscypha*, *Meliniomyces*, and *Pezicula*, were highlighted as major candidates organizing fungal assembly landscapes (Fig. 4b). The analysis also suggested that some Mortierellaceae (Mortierellomycota) fungi, which have been known for their saprotrophic and endophytic lifestyles^34^, showed significant Δtopography and/or Δevenness values (e.g., *Podila* and *Mortierella* on *Pinus* and *Populus* plants, respectively; Fig. 4b). Ectomycorrhizal fungi such as *Russula*, *Tomentella*, and *Amanita* showed significant influence on the assembly landscapes of fungal communities as well (Supplementary Table S5). However, their impacts were less pronounced than those of the endophytic taxa listed above despite their high abundance within the plant root systems (Fig. 2a and Supplementary Figs. S17–S20). Although the list of microbes with the strongest potential effects on fungal community assembly was dominated by endophytic fungi, some bacterial genera were highlighted as potential keystone taxa for fungal community assembly (e.g., Chloroflexota bacterial genus *Thermosporothrix* on *Juglans*) (Fig. 4b).

### Key bacteria consistently reshape prokaryotic landscapes

Whereas the root mycobiome processes were characterized by the predominant roles of endophytic fungi, the assembly landscape of the root-associated prokaryotic communities appeared to be organized by numerous microbial taxa (Fig. 4c and Supplementary Table S6). The histograms of Δtopography showed fat-tailed distributions; 132 prokaryotic (92.3%) and 25 fungal (75.8%) genera exhibited significant impacts on the entire architecture of prokaryotic assembly landscapes in at least one of the host-plant backgrounds (*q-*value < 0.05). This result suggests that the assembly landscapes of root-associated prokaryotic communities are much more labile than those of fungal communities. We also found that 74 prokaryotic (51.7%) and 7 fungal (21.2%) genera exhibited lower Δevenness than expected by chance (*q-*value < 0.05). This trend suggests that as their abundance increased, many microbial taxa promoted shifts from assembly landscapes characterized by numerous shallow basins to those with fewer but broader basins.

Among the microbial genera highlighted in the sensitivity analysis, the two bacterial genera *Candidatus* Solibacter (Acidobacteriota) and *Candidatus* Udaeobacter (Verrucomicrobiota) were consistently inferred to exert strong impacts on the entire assembly landscape architecture of prokaryotic communities across all the six host-plant backgrounds examined (Fig. 4d). Meanwhile, their impacts on the evenness of assembly landscapes were variable among host-plant backgrounds. We also found that endophytic fungi in the genera *Meliniomyces* and *Hyaloscypha* could have inter-kingdom impacts on prokaryotic community assembly (*Meliniomyces* in *Pinus* roots and *Hyaloscypha* in *Populus* roots; Fig. 4d).

### Candidate keystones are associated with basin turnover

**Fig. 5:**
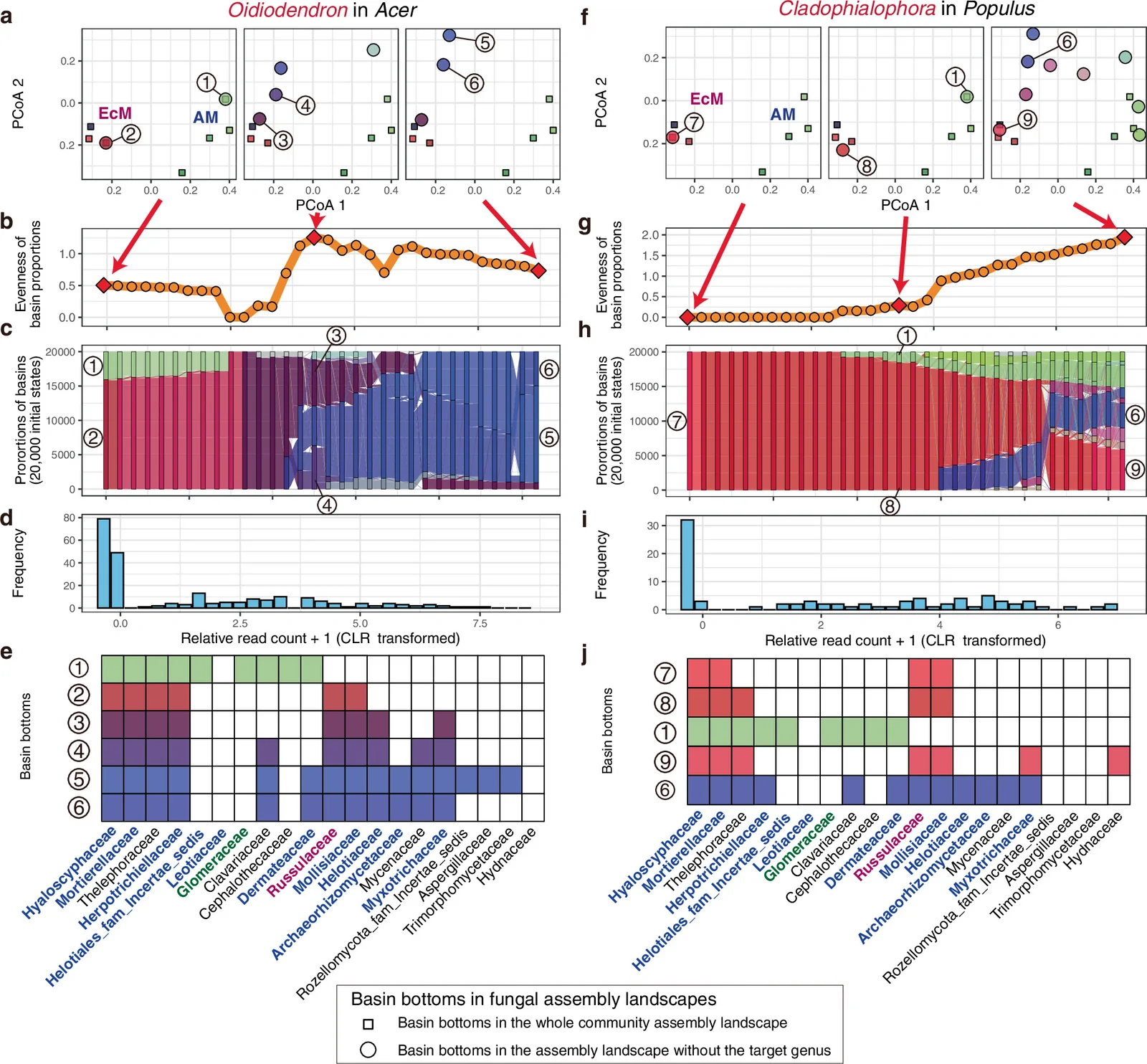
Fungal assembly landscape reorganization. Changes in the frequency distribution of fungal assembly landscape basins are shown along the abundance gradient of microbial genera highlighted in Fig. 4b [*Oidiodendron* in *Acer* roots (**a**–**e**) and *Cladophialophora* in *Populus* roots (**f**–**j**)]. Frequencies were evaluated through 20,000 simulations of community assembly from randomly generated initial states under 32 abundance conditions ranging from absence to the maximum observed abundance in each host background. (**a,f**) Shifts of basin bottoms along the abundance gradient. Basin bottoms detected at three representative abundance levels within the 32 equally spaced relative abundance steps—absence (step 1), intermediate abundance (step 16), and maximum abundance (step 32)—are shown as circles, together with the states detected in Figure 2c on the PCoA plane (see Supplementary Fig. S47a). The two most frequently observed states under each condition are labeled with their IDs. **b**, **g** Changes in basin evenness. Shannon entropy of basin frequencies is shown along the abundance axis [centered log ratio (CLR)-transformed relative read counts of the focal genus]. **c**, **h** Flow diagrams of community assembly destinations. Each bar plot depicts frequencies of basin bottoms under the corresponding abundance condition. Basin bottoms reached from the same initial community state across adjacent abundance steps are connected by bands. Colors represent community compositional similarity based on Jaccard dissimilarity, with more similar compositions shown in more similar colors (see Fig. 2c and Supplementary Fig. S48). **d**, **i** Histograms of CLR-transformed relative read counts of the focal genera in the respective host plants. **e**, **j** Family-level compositions of frequently detected basin bottoms. Fungal families are colored according to functional guilds: green, arbuscular mycorrhizal; magenta, ectomycorrhizal; blue, potentially endophytic; black, families with multiple or other functional attributes. Results for other highlighted fungal and prokaryotic genera are provided in Supplementary Figs. S21 and S22. Source data are provided as a Source data file.

**Fig. 6:**
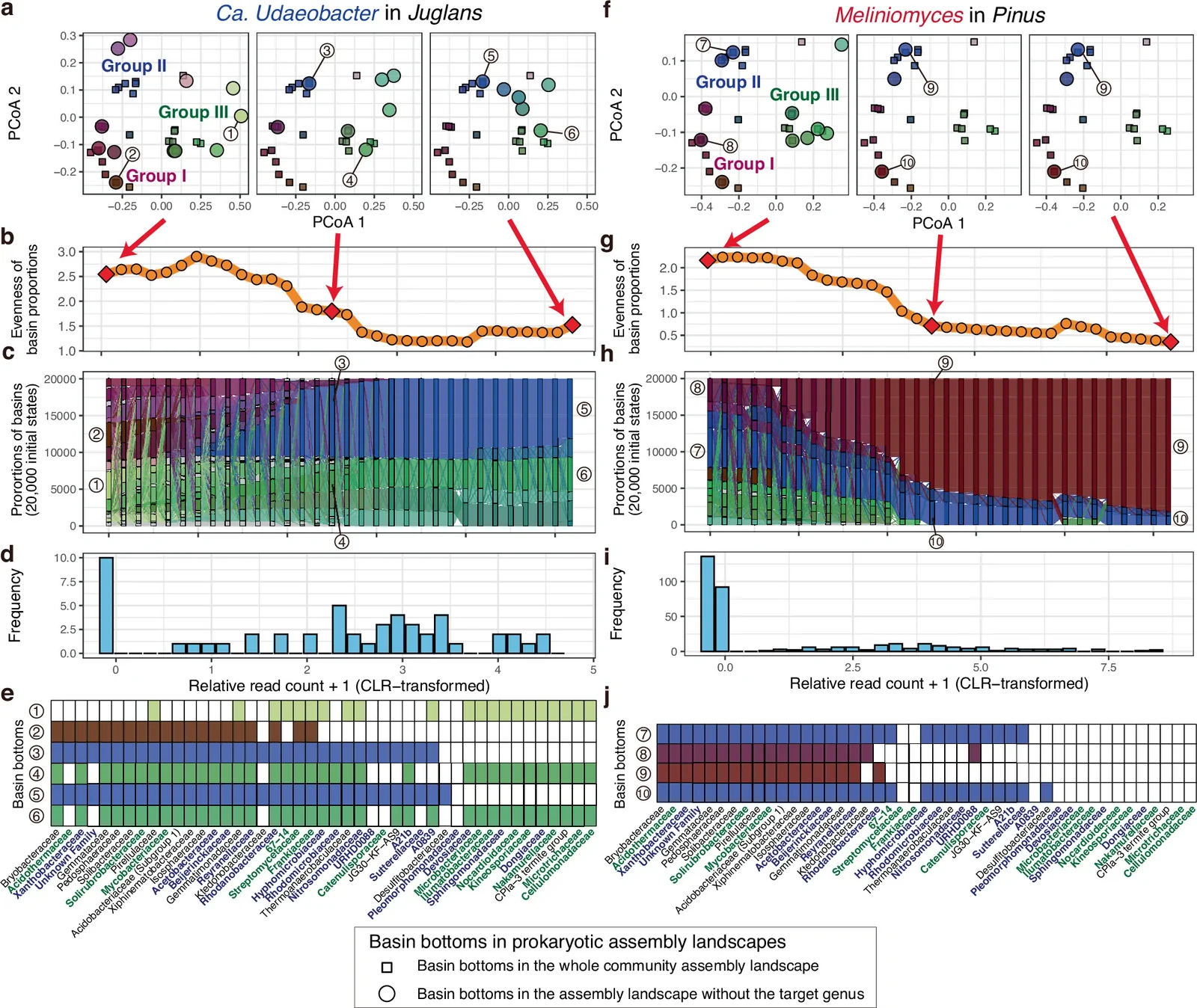
Prokaryotic assembly landscape reorganization. Changes in the frequency distribution of prokaryotic assembly landscape basins are shown along the abundance gradient of microbial genera highlighted in Fig. 4d [*Candidatus* Udaeobacter in *Juglans* roots (**a**–**e**) and *Meliniomyces* in *Pinus* roots (**f**–**j**)]. Frequencies were evaluated through 20,000 simulations of community assembly from randomly generated initial states under 32 abundance conditions ranging from absence to the maximum observed abundance in each host background. **a**, **f** Shifts of basin bottoms along the abundance gradient. Basin bottoms detected at three representative abundance levels within the 32 equally spaced relative abundance steps—absence (step 1), intermediate abundance (step 16), and maximum abundance (step 32)—are shown as circles, together with the states detected in Fig. 3c on the PCoA plane (see Supplementary Fig. S47b). The two most frequently observed states under each condition are labeled with their IDs. **b**, **g** Changes in basin evenness. Shannon entropy of basin frequencies is shown along the abundance axis [centered log ratio (CLR)-transformed relative read counts of the focal genus]. **c**, **h** Flow diagrams of community assembly destinations. Each bar plot depicts frequencies of basin bottoms under the corresponding abundance condition. Basin bottoms reached from the same initial community state across adjacent abundance steps are connected by bands. Colors represent community compositional similarity based on Jaccard dissimilarity, with more similar compositions shown in more similar colors (see Fig. 3c and Supplementary Fig. S49). **d**, **i** Histograms of CLR-transformed relative read counts of the focal genera in the respective host plants. **e**, **j** Family-level compositions of frequently detected basin bottoms. Prokaryotic families are colored according to phylum-level taxonomy: green, Actinomycetota; blue, Pseudomonadota; black, other phyla. Results for other highlighted fungal and prokaryotic genera are provided in Supplementary Figs. S23 and S24. Source data are provided as a Source data file.

We next conducted a detailed evaluation of how the number and distribution of basins could change along the abundance gradient of each genus highlighted in the Δtopography and Δevenness analyses (Figs. 5 and 6). For example, at low abundance of the root endophytic fungus *Oidiodendron*, the fungal assembly landscape on *Acer* was characterized by two distinct ectomycorrhizal and arbuscular mycorrhizal basins (Fig. 5a–e). As the relative abundance of *Oidiodendron* increased, the arbuscular-mycorrhizal basin was progressively replaced by the ectomycorrhizal basins. A similar loss of arbuscular-mycorrhizal basins within the fungal assembly landscape was observed along the abundance gradient of the mycophagous bacterial genus *Collimonas*^35^ on the dual-mycorrhizal host *Populus* (Supplementary Fig. S21). In contrast, a root endophytic fungus, *Cladophialophora*, was associated with the coexistence of narrower basins involving both ectomycorrhizal and arbuscular-mycorrhizal states along its abundance gradient on *Populus* (Fig. 5f–j). A similar pattern was observed for another endophytic fungal genus *Pezicula* (Supplementary Fig. S21). On ectomycorrhizal plants, endophytic fungi such as *Podila* and *Phialocephala* were associated with shifts between different ectomycorrhizal states (Supplementary Fig. S22).

In the analysis of prokaryotic communities, the number and distribution of basins changed markedly along the abundance gradients of potential keystone taxa (Fig. 6). These transitions involved dynamic shifts in the repertoire of basins. For instance, on the host *Juglans*, three basin groups of prokaryotic communities (Groups I–III; Fig. 3c) were present at low abundance of *Candidatus* Udaeobacte*r* (Fig. 6a–e). As *Candidatus* Udaeobacter abundance increased, Group I basins, mainly composed of core bacterial families, were replaced by Group II basins, which included additional families from Pseudomonadota (e.g., Sutterellaceae and A21b). Similar replacements of Group I basins by Group II basins, resulting in increased representation of Groups II and III basins, were also observed for other bacterial genera such as *Candidatus* Solibacter in *Juglans* roots and *Reyranella* in *Betula* roots (Supplementary Figs. S23–S24). In contrast, the endophytic fungus *Meliniomyces* was associated with the prevalence of community states composed of core bacterial families (Group I) in the roots of *Pinus* (Fig. 6f–j). Other potential keystone microbes were linked to directional changes in the assembly landscape architecture; for example, an increase in the abundance of the Chloroflexi genus *G12-WMSP1* was associated with the dominance of Group II basins in *Acer* roots (Supplementary Fig. S23).

### Candidate keystones reflect mycorrhizal colonization

To validate the results of the keystone exploration analysis, we examined the community-scale impacts of the highlighted taxa using an alternative statistical framework and complementary data. Although datasets that allow comprehensive statistical analyses of root microbiome assembly remain scarce, a recent study has provided microscopic observational data on root colonization by arbuscular mycorrhizal and ectomycorrhizal fungi across ecologically diverse woody plant species spanning a broad geographic range in Europe^36^ [from central Italy (42.08°N, 9.82°E) to northern Norway (69.46°N, 30.02°E)]. The dataset also included amplicon sequencing data targeting the fungal ITS region and the prokaryotic 16S rRNA gene, providing an opportunity to statistically evaluate the roles of diverse fungal and bacterial taxa in mycorrhizal symbiosis. Thus, we examined whether the candidate keystone taxa highlighted by the assembly landscape analysis of a cool-temperate forest dataset in Japan were significantly associated with variation in mycorrhizal community assembly across European forests.

For this analysis, we listed the top 10 genera with the highest Δtopography scores for each host-plant background in the fungal community dataset. As described above, this set consisted mainly of endophytic fungi in the orders Helotiales and Chaetothyriales and saprotrophic fungi spanning the saprotrophic to endophytic ecological spectrum (Fig. 4b). For genera occurring in both the Japan and European datasets, we constructed a series of generalized linear mixed models (GLMMs) to evaluate how variation in the target taxon’s abundance was associated with root colonization by arbuscular mycorrhizal or ectomycorrhizal fungi.

**Fig. 7:**
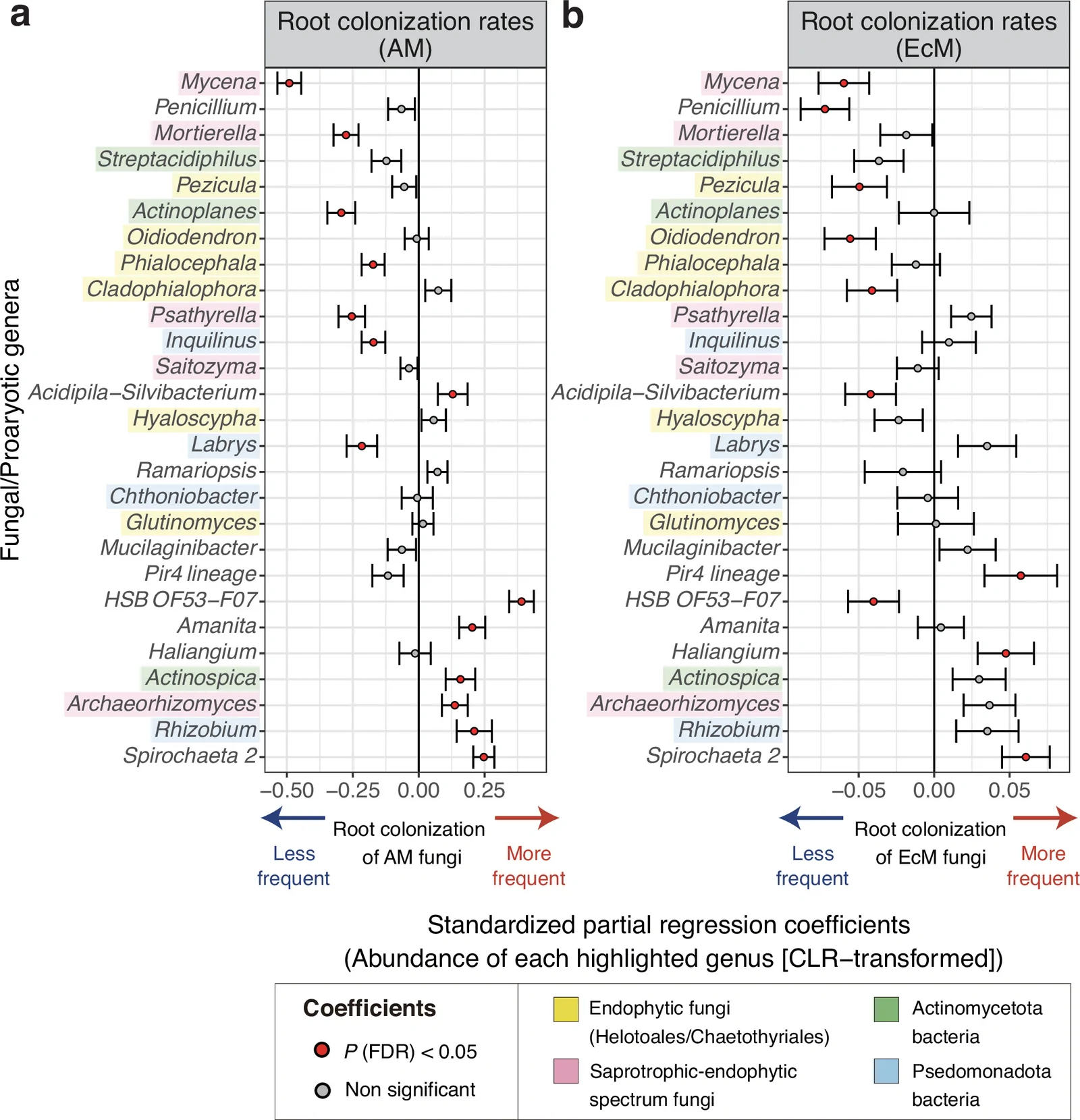
Ecological associations of candidate keystone genera across European forests. The ecological relevance of the candidate keystone genera highlighted in the assembly landscape analysis of the cool-temperate forest from Japan (the top 10 genera with the highest Δtopography values within each host-plant background in Fig. 4b) was evaluated by compiling a root microbiome dataset collected across Europe^36^ (see Supplementary Table S7 for statistics of each genus). **a** Associations with arbuscular mycorrhizal symbiosis. For each target genus, the relationship between its abundance [centered log ratio (CLR)-transformed relative read counts +1] and the root colonization rate of arbuscular mycorrhizal fungi was evaluated in a generalized linear mixed model (GLMM; *n* = 219 samples). Positive and negative values of the standardized regression coefficients (represented as circles) indicate positive and negative associations with arbuscular mycorrhizal colonization rate, respectively. The error bars represent standard errors of the coefficients. *P* values were adjusted using the Benjamini–Hochberg procedure. The significant coefficients (false discovery rate (*q-*value) < 0.05; two-sided test) are shown in red. **b** Associations with ectomycorrhizal symbiosis. Relationships between genus abundance and ectomycorrhizal colonization rate were evaluated using the same modeling framework (GLMM; *n* = 218 samples). Source data are provided as a Source data file.

We then found that 6 of 14 fungal genera and 8 of 13 bacterial genera examined were significantly associated with the root colonization rates of arbuscular mycorrhizal fungi depending on their abundance (*q-*value < 0.05; Fig. 7a and Supplementary Table S7). The taxa exhibiting significant associations included the endophytic fungus *Phialocephala* and saprotrophic–endophytic spectrum fungi such as *Mycena*, *Mortierella*, *Psathyrella*, and *Archaeorhizomyces,* as well as some Actinomycetota bacterial genera (*Streptacidiphilus*, *Actinoplanes*, and *Actinospica*) and the nitrogen-fixing bacterial genus *Rhizobium*.

We also found that 5 of 14 fungal genera and 5 of 13 bacterial genera examined were significantly associated with ectomycorrhizal colonization rates depending on their abundance (*q-*value < 0.05; Fig. 7b and Supplementary Table S7). Significant associations with ectomycorrhizal symbiosis were indicated for the endophytic fungal genera *Pezicula*, *Oidiodendron*, and *Cladophialophora* as well as for the saprotrophic–endophytic spectrum fungus *Mycena*.

Overall, these results indicate that the candidate keystone taxa identified in the forest microbiome dataset from Japan corresponded with variation in mycorrhizal fungal assembly across European forests. These cross-system patterns support the ecological relevance of the assembly landscape framework and suggest that the highlighted taxa could contribute to transitions between major mycorrhizal states.

## Discussion

Inspired by the concept of stability landscapes, we developed a statistical framework to identify species or taxa that play central roles in ecosystem-scale processes. Application of this keystone exploration framework to a forest root microbiome dataset from Japan (Figs. 4–6) identified candidate keystone microbes linked to major shifts in mycorrhizal symbiosis across geographically distant European forests (Fig. 7). A keystone species or taxon was originally defined as "one whose impact on its community or ecosystem is large, and disproportionately large relative to its abundance”^9^. Whereas this classic definition has primarily been applied in studies of macro-organisms (e.g., plants and animals), microbiome research has continued to propose modified operational definitions to address the complex and dynamic nature of microbial interactions^5,37–39^. Specifically, network hubs identified from microbial co-occurrence patterns are often regarded as candidate keystone taxa^5,40–42^. More recently, several in silico approaches have been proposed to evaluate how the presence or removal of particular taxa may affect overall community structure^37,38^. Building upon these advances, our approach extends such pattern-oriented frameworks by integrating theoretical perspectives on system-scale principles underlying community dynamics (see Supplementary Note 5). Based on the stability landscape concept, a keystone species (or taxon) can therefore be more explicitly conceptualized as one whose increase or decrease in abundance reshapes the rules governing community assembly.

The keystone exploration analysis suggested that endophytic fungi functioned as major drivers of microbial community assembly within plant root systems (Fig. 4b). Among them, those described as dark septate endophytes^32^ (e.g., *Cladophialophora*, *Phialocephala*, *Hyaloscypha*, and *Pezicula*) can support host plant physiology by, for example, enhancing the nitrogen and potassium uptake efficiency^43,44^. Moreover, an experimental study has shown that *Phialocephala* colonizes plant roots prior to ectomycorrhizal fungi, forming tripartite symbioses with ectomycorrhizal fungi and host plants^43^. Given that ectomycorrhizal fungi vary in their physiological impacts on host plants^45,46^, priority effects exerted by endophytic fungi could drive underappreciated dynamics in below-ground ecosystems^43,47^. In other words, endophytic fungi not only function as symbionts with direct beneficial effects but also indirectly influence host performance by regulating the assembly of ectomycorrhizal fungi within root systems. To understand the processes by which endophytic fungi regulate plant–fungus symbiosis, the mechanisms that allow these fungi to become pioneer colonizers need to be clarified. For example, melanin, which characterizes the appearance of dark septate endophytes, likely contributes to competitive superiority in chemical and physical interactions with other early-colonizing fungi in plant root systems^48,49^.

The hidden roles of endophytes were further illuminated in the analysis of plants associated with both ectomycorrhizal and arbuscular mycorrhizal fungi (Fig. 4b). Some plant species (e.g., *Populus deltoides*) have been known to build mutualistic interactions with the two groups of mycorrhizal fungi, potentially gaining distinct physiological benefits from such dual symbiosis^25^. In contrast, for plants conventionally classified as arbuscular mycorrhizal (e.g., *Acer* and *Juglans*), root-tip states dominated by ectomycorrhizal fungi can be disadvantageous. In fact, some ectomycorrhizal fungi are known to invade the roots of arbuscular mycorrhizal plants without forming specialized symbiotic structures, thereby impairing mutualistic interactions between the plants and their arbuscular mycorrhizal fungi^50–52^. Consequently, the observed shifts between arbuscular mycorrhizal states and ectomycorrhizal-fungus-dominated states are expected to entail substantial changes in plant physiological conditions. The insight that endophytic fungi can drive drastic transitions in mycorrhizal symbiosis underscores the need to deepen our understanding of the ecology and physiology of the underappreciated fungal guild. The endophytic fungi highlighted in our analysis (e.g., *Cladophialophora*, *Oidiodendron*, *Hyaloscypha*, and *Mortierella*; Figs. 4 and 5) often show high infection rates on diverse taxa of ectomycorrhizal and arbuscular mycorrhizal plants not only in cool-temperate but also in warm-temperate and subtropical regions^33,53^. Although these endophytes are often overlooked because of their lack of conspicuous morphological features within plant tissues^32,33^, they likely play fundamental roles in root-associated mycobiome dynamics across broad geographic ranges (Figs. 4 and 7).

Whereas the fungal community processes were characterized primarily by endophytic fungi, the assembly of root-associated prokaryotes appeared to be influenced by a broader range of both prokaryotic and fungal taxa (Fig. 4c, d). This result suggests that prokaryotic community structure shifts between alternative stable states more flexibly than fungal community structure within plant root systems. The reconstructed basins on the prokaryotic assembly landscapes were categorized into three types: communities consisting of commonly observed (core) taxa (Group I), and those further characterized by additional Pseudomonadota (Group II) or Actinomycetota (Group III) families (Fig. 3c). The core sets included bacterial taxa associated with cell wall degradation (e.g., Acidobacteriaceae, Acidothermus, and Isosphaeraceae) and plant growth promotion (e.g., Acetobacteraceae, Bryobacteraceae, and Solirubrobacteraceae) functions^54–59^, which may underlie their consistent presence in root microbiomes. The Actinomycetota families unique to Group III basins (e.g., Microbacteriaceae and Nocardioidaceae) are potentially recruited in response to environmental stresses (e.g., drought) or plant-derived stimuli^60–62^. In contrast, the Pseudomonadota taxa specific to Group II basins (e.g., Sutterellaceae and A21b) remain poorly characterized, both ecologically and physiologically. Intriguingly, transitions among the three community types (Groups I-III) were observed across all six plant species examined (Fig. 3c). Consequently, prokaryotic communities are likely to shift among representative states in response to subtle changes in biotic and abiotic conditions within roots.

Although numerous taxa were highlighted as potential drivers of prokaryotic community assembly, the Acidobacteriota genus *Candidatus Solibacter* exhibited the largest or second-largest impacts on the overall topography of prokaryotic assembly landscapes across all six host plants (Fig. 4d). Bacteria in this genus are known to degrade plant cell walls and produce diverse polysaccharides and secondary metabolites^63,64^, possibly modifying the microenvironments surrounding other prokaryotes within the endosphere and rhizosphere. Likewise, the Verrucomicrobiota genus *Candidatus* Udaeobacter showed the strongest impacts on assembly landscape topography in five of the six host-plant backgrounds (Figs. 4d and 6). Species in the genus adapt to nutrient-poor environments as oligotrophic bacteria, depending on metabolites emitted by other microbes in the soil^65^. However, once established, their populations persist as major components of soil microbiomes, potentially stabilizing overall prokaryotic community processes^65,66^. As their genomes are enriched with genes for protein and peptide degradation^65^, they likely increase amino acid availability for co-occurring bacteria with complementary physiological properties. From this perspective, prokaryotic community assembly may be disproportionately driven by metabolically versatile taxa that govern resource-mediated cooperation across taxonomically diverse members.

Although careful consideration of potential confounding factors is required, cross-kingdom interactions between fungi and prokaryotes likely act as key determinants of both fungal and prokaryotic community assembly^67,68^. Interestingly, the candidate keystone bacterium *Collimonas*, highlighted in the fungal assembly landscape analysis (Fig. 4b and Supplementary Fig. S21), has been reported to extract nutrients from living fungal hyphae, including both arbuscular and ectomycorrhizal ones, through mycoparasitic activity^35^. Conversely, certain endophytic fungi, such as *Meliniomyces* and *Hyaloscypha* (Figs. 4d, 6 and Supplementary Fig. S23), may also influence microbiome assembly dynamics by modulating host–prokaryote positive feedback via their diverse extracellular metabolites^69,70^ (e.g., amino acids, peptides, and lipids). Therefore, these fungi and prokaryotes could interact metabolically and jointly shape the overall dynamics of the root microbiome.

Toward a more comprehensive understanding of potential keystone taxa in complex microbial ecosystems, careful interpretation of the results is necessary. First, although the sensitivity analysis based on a statistical-physics framework provides a systematic means of estimating how the abundance of each microbe can alter whole-community processes, the roles of the highlighted taxa remain to be experimentally validated. For example, by inoculating potential keystone microbes to seedlings as pioneer symbionts, their priority effects on subsequent colonizers could be evaluated. In parallel, the introduction of aseptic seedlings into forest stands followed by subsequent sampling will reveal whether the focal microbes actually colonize plant roots as pioneer symbionts in natural ecosystems. Second, microbiome assembly within plant root systems can be determined not only by biotic but also by abiotic factors. The statistical model for inferring assembly landscapes is flexible in that it incorporates both implicit and explicit variables representing environmental conditions^14^ (Fig. 1b). While we included host plant identity as a background variable in the model, a more thorough estimation of assembly landscape architecture could be achieved by incorporating microenvironmental variables (e.g., rhizosphere soil pH or root exudate concentrations) as covariates. Third, due to its exhaustive computational approach, our current analysis targeted assembly landscapes defined with the binary-format (presence/absence) data of the family-level community structure. More detailed simulations of community dynamics will be achieved by extending computational capacities and applying emerging algorithms for summarizing high-dimensional microbiome data (e.g., natural language processing of the latent Dirichlet allocation model for quantitative microbiome data^22^). Fourth, although prokaryotes and fungi are often regarded as core components of root microbiomes, thorough knowledge of rhizosphere ecological processes is unavailable without evaluating the roles of other soil organisms such as protists^71^, nematodes^72^, and arthropods^68^. Once the limitation related to computational capacity is overcome, integrated assembly landscape analysis of whole soil communities will provide overarching insights into below-ground ecosystem dynamics.

In parallel with statistical analyses of ecological data, genomic and physiological approaches will deepen our understanding of the mechanisms that regulate below-ground plant–microbe symbiosis. Genome sequencing, for example, may reveal unique metabolic niches of potential keystone microbes^73^. Likewise, transcriptomic and metabolomic analyses targeting both symbionts and host plants will uncover direct physiological impacts of keystone microbes on other microbiome members (e.g., suppression via antibiotics^74^) as well as indirect control of root-associated microbiomes through the activation of plant immune systems^75^. Although intricate, the dynamics of root-associated microbiomes will be progressively elucidated through the integration of data-driven explorations of community-scale phenomena with reductionistic physiological approaches.

## Methods

### Root microbiome data

We sampled root tips (1 cm terminal roots) and leaves in the research forest of Sugadaira Research Station, Mountain Science Center, University of Tsukuba, Ueda, Nagano Prefecture, Japan (36.524 ºN; 138.349 ºE; 1340 m a.s.l.) in a previous study^26^. In the present study, we combined the previously published fungal community and host-plant dataset^26^ with newly obtained prokaryotic sequence data of root samples. Briefly, the root-tip samples were sonicated in 0.5% Tween 20 and surface sterilized subsequently with NaClO (Nacalai Tesque, effective chlorine concentration ca. 11%) diluted to 1% (v/v), sterile distilled water (three times), and 99.5% ethanol^26^. After DNA extraction with a cetyltrimethylammonium bromide (CTAB) method, the internal transcribed spacer 1 (ITS1) region of fungi^76^, the internal transcribed spacer 2 (ITS2) region of plant^77^ and the 16S rRNA region of prokaryotes^78^ were PCR-amplified, and the purified amplicons were sequenced using an Illumina MiSeq sequencer^26^ (primer information is provided in Supplementary Data 1). The resulting fungal ITS1 and plant ITS2 sequence data were identical to those reported in the previous study^26^, whereas the prokaryotic 16S rRNA sequence data (73,183,237 reads) were newly generated for the present study. The obtained sequencing reads of the ITS1, ITS2, and 16S rRNA regions were converted into FASTQ files using bcl2fastq (version 1.8.4) and demultiplexed with Claident v0.9.2022.01.26^79,80^. From these outputs, amplicon sequence variants (ASVs) were constructed by adaptor trimming by Cutadapt^81^ (version 3.7), quality filtering, and denoising by DADA2^82^ v.1.18.0 of R 4.2.2^83^. Contaminant ASVs were removed with the R package decontam^84^ (prevalence method). Taking into account duplication error in PCR, the ASVs were re-clustered into operational taxonomic units (OTUs) with a 97% similarity threshold using the program VSEARCH v2.21.1^85^. The taxonomic assignments of fungal and prokaryotic OTUs were performed based on the UNITE^86^ general FASTA release for eukaryotes 2. Version 18.07.2023 and the SILVA^87^ version 138 database using the naive Bayesian classifier method in DADA2^82,88^. The host-plant annotations were also performed using the plant OTU sequences with five-nearest-neighbor method^79^ against the BLAST nt database^89^ (version 2022-07-15) and neighbor-joining phylogenetic analysis^90^ with reference leaf samples^26^. Root samples with fewer than 1,000 fungal or prokaryotic reads were discarded. Then, obtained fungal/prokaryotic OTU matrices of the samples whose host plants were detected more than 50 times (*Acer*, *Betula*, *Juglans*, *Larix*, *Pinus* and *Populus*) were rarefied with coverage-based method using vegan package v.2.6-8^91^. In total, both fungal and prokaryotic community data were obtained for 1270 root samples collected from the 125 sampling points.

### Host preference of the fungal/prokaryotic families

To evaluate the host specificity of the fungal and prokaryotic families used in energy landscape estimation, we calculated the *d*’ metric of interaction specificity^92^ and two dimensional preference of plant–fungus associations (2DP)^93^ for the 34 fungal and 45 prokaryotic families (selected in Supplementary Figs. S11 and S13) following the same procedure as in our previous study^26^. In these analyses, the fungal/prokaryotic community data were binarized with the same criteria as energy landscape analysis (described in detail below). The standardized indices and their statistical significances were assessed by comparing the observed data with null model simulations. Specifically, the host plant information was shuffled among root samples collected at the same sampling positions (10,000 permutations), and the indices calculated from the randomized datasets were compared with those from the original dataset. For each fungal and prokaryotic family, a ${{\rm{standardized}}}\,{{d}^{\prime}}$ metric was calculated as follows:

1 $${{\rm{standardized}}}\,{d}^{{\prime}}=\frac{{d}_{{\rm{obs}}}^{\prime}-{{\rm{Mean}}}({d}_{{\rm{rand}}}^{\prime})}{{{\rm{SD}}}({d}_{{{\rm{rand}}}}^{\prime})}$$

where ${d}_{{{{\rm{obs}}}}}^{{\prime}}$ was the ${d}^{{\prime} }$ estimate of the original data and ${{{\rm{Mean}}}}({d}_{{{{\rm{rand}}}}}^{{\prime} })$ and ${{{\rm{SD}}}}({d}_{{{{\rm{rand}}}}}^{{\prime} })$ were the mean and standard deviation of the *d’* of randomized data matrices. For each pair of six host plants and fungal/prokaryotic family, standardized 2DP was also calculated as follows:

2 $${{{\rm{standardized}}}}\,2{{{\rm{DP}}}}=\frac{{N}_{{{{\rm{obs}}}}}\left(i,j\right)-{{{\rm{Mean}}}}({N}_{{{{\rm{rand}}}}}\left(i,j\right))}{{{{\rm{SD}}}}({N}_{{{{\rm{rand}}}}}\left(i,j\right))}$$

where ${N}_{{{{\rm{obs}}}}}\left(i,j\right)$ denoted the number of root samples from which a focal combination of a symbiont family and a host plant was observed in the original data, and the ${{{\rm{Mean}}}}({N}_{{{{\rm{rand}}}}}\left(i,j\right))$ and ${{{\rm{SD}}}}({N}_{{{{\rm{rand}}}}}\left(i,j\right))$ were the mean and standard deviation of the number of samples for the focal symbiont–plant pair across randomized matrices. False discovery rates [FDR] were also calculated to evaluate the statistical significance of the two indices^94^ (*d*’; one-tailed tests; 2DP, two-tailed tests).

### Energy landscape analysis

The assembly landscape architecture was estimated for each of the fungal and prokaryotic community datasets. We applied the statistical framework of energy landscape analysis, which has been widely used to characterize the relationship between multi-dimensional system states (e.g., brain activity patterns) and their potentials (observation probabilities), based on the Ising model in statistical physics^14,28^. In this framework, the probabilities of community states $p\left({\vec{{{{\boldsymbol{\sigma }}}}}}^{(k)}|{{{\boldsymbol{\varepsilon }}}}\right)$, under a given set of environmental conditions [${{{\boldsymbol{\varepsilon }}}}=({{{{\boldsymbol{\varepsilon }}}}}_{1},{{{{\boldsymbol{\varepsilon }}}}}_{2},\ldots,{{{{\boldsymbol{\varepsilon }}}}}_{M})$; *M* is the number of vectors representing environmental factors], are defined by the following equations^14^:

3 $$p\left({\vec{{{{\boldsymbol{\sigma }}}}}}^{(k)}|{{{\boldsymbol{\varepsilon }}}}\right)={e}^{-E\left({\vec{{{{\boldsymbol{\sigma }}}}}}^{(k)},{{{\boldsymbol{\varepsilon }}}}\right)}/Z$$

4 $$Z=\sum\limits_{k=0}^{{2}^{S-1}}{e}^{-E\left({\vec{{{{\boldsymbol{\sigma }}}}}}^{(k)},{{{\boldsymbol{\varepsilon }}}}\right)}$$

where ${\vec{{{{\boldsymbol{\sigma }}}}}}^{(k)}=({{\sigma }_{1}}^{\left(k\right)},{{\sigma }_{2}}^{\left(k\right)},\ldots,{{\sigma }_{S}}^{\left(k\right)})$ denotes the vector of the k-th community state, and *S* is the total number of species or taxa examined. Each element ${{\sigma }_{i}}^{\left(k\right)}$ is a binary variable indicating the presence (1) or absence (0) of taxon *i*, defined using optimized thresholds applied to relative-abundance data (described below; Supplementary Fig. S11a). Thus, there are a total of ${2}^{S}$ possible community states. The energy of the *k*-th community state, $E\left({\vec{{{{\boldsymbol{\sigma }}}}}}^{(k)},\,{{{\boldsymbol{\varepsilon }}}}\right)$, is given by:

5 $$E\left({\vec{{{{\boldsymbol{\sigma }}}}}}^{(k)},{{{\boldsymbol{\varepsilon }}}}\right)=-\sum\limits_{i=1}^{S}{h}_{i}{{\vec{\sigma }}_{i}}^{\left(k\right)}-\sum\limits_{i=1}^{S}\sum\limits_{j=1}^{M}{g}_{{ij}}{{{{\boldsymbol{\varepsilon }}}}}_{j}{{\vec{\sigma }}_{i}}^{(k)}-\sum\limits_{i=1}^{S}\sum\limits_{l=1,l\ne i}^{S}{J}_{{il}}{{\vec{\sigma }}_{i}}^{(k)}{{\vec{\sigma }}_{l}}^{(k)}/2$$

where ${h}_{i}$ is the net effect of implicit (unobserved) factors representing whether the *i-*th taxon is more likely to co-occur (${h}_{i}$ > 0) or not (${h}_{i}$ < 0); ${g}_{{ij}}$ denotes the effect of the *j-*th (observed) environmental variable on the occurrence of the *i-*th species/taxa; ${J}_{{il}}$ captures the pairwise co-occurrence relationship between *i-*th and *l-*th species/taxa. Since the logarithm of the probability of a community state is inversely proportional to $E\left({\vec{{{{\boldsymbol{\sigma }}}}}}^{(k)},{{{\boldsymbol{\varepsilon }}}}\right)$, a community state having lower $E\left({\vec{{{{\boldsymbol{\sigma }}}}}}^{(k)},{{{\boldsymbol{\varepsilon }}}}\right)$ is more frequently observed. This energy metric does not correspond in any way to physical energy; rather, it serves to distinguish between more probable and less probable community states. The parameters ${h}_{i}$, ${g}_{{ij}}$, and ${J}_{{il}}$ were estimated based on a stochastic approximation employing a persistent Markov Chain Monte Carlo method as detailed elsewhere^14^. Based on the inferred models, the states having lower energy than surrounding ones in the assembly graph^30,31^ were identified as basin bottoms using a steepest descent procedure^14^ (number of initial states = 150,000; Supplementary Fig. S25; see Supplementary Note 6 for details). The bottoms of basins on the reconstructed landscape (i.e., community states located at local energy minima) do not necessarily correspond to stable states (i.e., attractors)^20,21,47^. Nevertheless, energy landscape analysis for describing the probability distribution of community states provides a unified statistical framework for examining how community assembly rules depend on background conditions.

The statistical framework of energy landscape analysis was applied to the family-level compositional data of fungal and prokaryotic communities, respectively, after excluding five fungal samples and three prokaryotic samples in which more than 50% of sequence reads could not be annotated at the family level. The community data were filtered with optimized abundance-based binarization thresholds for this dataset defined as

6 $${{{\rm{Mean}}}}\left(\log \left({X}_{i}\right)\right)-2{{{\rm{SD}}}}\left(\log \left({X}_{i}\right)\right)$$

where ${{{\rm{Mean}}}}\left(\log \left({X}_{i}\right)\right)$ and ${{{\rm{SD}}}}(\log \left({X}_{i}\right))$ denote mean and standard deviation of the log-transformed read counts of taxon *i* across samples in which the taxon was detected (Supplementary Figs. S11–13, S26, and Supplementary Tables S8–S11; see Supplementary Note 4 for details).

Because exhaustive exploration of assembly graphs involves ${2}^{S}$ possible community states (where *S* is the number of taxa), the number of taxa included in the analysis was reduced by prioritizing and selecting informative families separately for fungal and prokaryotic datasets. To quantify the contribution of each family to overall community variation, a series of permutational analyses of variance (PerMANOVA)^95^ was performed on the relative abundance matrices, treating the presence or absence of each focal family as an explanatory variable (10,000 permutations). Bray–Curtis dissimilarity was used to quantify community differences among root samples, while host plant identity and sampling point were included as covariates. Families were then ranked according to their explanatory power (*R*²) in the PerMANOVA (Supplementary Tables S8–S9).

The optimal number of families to retain for energy landscape analysis was determined by evaluating how well binarized community matrices preserved information contained in the original quantitative data. Specifically, community dissimilarities calculated from relative abundance data (Bray–Curtis dissimilarity) were compared with those calculated from binarized data (Jaccard dissimilarity). The family set yielding the highest Kendall’s rank correlation coefficient between the two dissimilarity matrices was selected as the optimal binarized dataset (Supplementary Figs. S11a and S13).

The obtained presence/absence matrices comprising 34 fungal families and that comprising 45 prokaryotic families were respectively subjected to energy landscape analysis, in which host plants were included as categorical (dummy) explanatory variables, using the rELA package^14^ v0.60 of R 4.4.1^83^. The reproducibility and uncertainty in the assembly landscape reconstruction were evaluated based on 100-fold cross-validation and bootstrapping analyses (Supplementary Figs. S27–S35; see Supplementary Notes 7–8 for details). The reconstructed energy landscapes (assembly landscapes) were visualized on the two-dimensional surface of a principal coordinate analysis (PCoA) applied to the binary community matrices of basin bottoms and their intermediate states. Disconnectivity graphs representing the depth of basins are shown as well for each host plant background in the fungal and prokaryotic data, respectively.

In addition, to evaluate landscape architecture at a finer taxonomic resolution, energy landscape analyses of fungal and prokaryotic genus-level compositions were conducted separately using the same procedures (Supplementary Figs. S36–S37; Supplementary Tables S12–S13; see Supplementary Note 9 for details). The basin bottoms in each host plant background were compared with those identified in the family-level analyses to confirm correspondences in their taxonomic compositions (Supplementary Fig. S38).

### Community structures and assembly landscape architecture

To assess how basin structures inferred from fungal and prokaryotic assembly landscapes (Figs. 2b, c and 3b, c) relate to observed community composition, we performed principal coordinate analysis (PCoA) and permutation-based tests using the R package vegan^91^. For each dataset (fungi and prokaryotes), we computed family-level dissimilarities under two representations. First, to capture abundance-weighted community composition, we calculated Bray–Curtis dissimilarities from the full family-level community composition matrix. Second, to align with the community-state definition used in the assembly landscape analysis, we calculated Jaccard dissimilarities from presence–absence profiles corresponding to the community states used in the assembly landscape analysis.

We then evaluated whether the observed dissimilarity structure was explained by basin assignments using permutational multivariate analysis of variance^95^ (PerMANOVA) and tested the homogeneity of multivariate dispersions using PERMDISP^96^ . Both tests were conducted at two hierarchical levels: individual basins (basin identity) and basin groups defined by characteristic taxonomic profiles (fungi: ectomycorrhizal vs arbuscular-mycorrhizal basins; prokaryotes: Groups I–III; see Figs. 2c and 3c). To account for host-plant background and sampling location effects, permutations were restricted within strata defined by host plant–site combinations. *P* values from PerMANOVA and PERMDISP were adjusted separately for multiple testing using the Benjamini–Hochberg procedure^94^.

### Sensitivity analysis for exploring potential keystones

Based on the energy landscape analysis platform, we performed a series of sensitivity analyses for exploring microbial taxa whose changes in abundance potentially induce substantial reorganizations of assembly landscapes. For each fungal and prokaryotic genus detected in more than 100 root-tip samples, we constructed an energy landscape model (5) by incorporating the relative abundance of the focal genus as an additional explanatory variable [i.e., an added vector in the environmental condition matrix ${{{\boldsymbol{\varepsilon }}}}=({{{\boldsymbol{\varepsilon }}}}_{1},{{{\boldsymbol{\varepsilon }}}}_{2},\ldots,{{{\boldsymbol{\varepsilon }}}}_{M})$] (Fig. 1f). Prior to this analysis, we confirmed that each focal genus was absent in at least 40 samples. Within the sensitivity analysis framework, we sequentially re-defined the community data matrix by excluding each focal genus. Specifically, OTUs annotated to the focal genus were removed, and the remaining OTUs were aggregated at the family-level community compositions (Fig. 1f and Supplementary Fig. S11b). We then binarized the abundance of the taxa targeted in the above energy landscape analysis using the same criteria. During this procedure, taxa occurring in 30 or fewer samples were excluded.

After estimating the parameters of the energy landscape statistical model (5), we compared the landscape architecture reconstructed under the absence of the focal genus with that estimated under the assumption of an intermediate abundance level. The absence of each genus was defined as the condition in which its abundance was lower than the binarization threshold, and the mean relative abundance across samples classified as absent was used for the landscape reconstruction under the absence scenario. For the intermediate-abundance scenario, the abundance level was set, by default, to the median abundance (50% quantile) across samples in which the focal genus was detected. This choice was made because alternative settings, such as maximum values, could strongly depend on species-specific abundance distributions, whereas median values are least affected by the presence of outliers. For each energy (assembly) landscape reconstructed under different abundance levels of the focal genus [0% (absence) vs. 50% (median) quantiles], we performed a steepest-descent simulation of community succession, starting from the same set of 20,000 randomly generated initial community states (Fig. 1d, g and Supplementary Fig. S11b). The degree to which assembly landscape topography changed along the abundance gradient of the focal genus *i* was quantified as:

7 $${\Delta {{{\rm{topography}}}}}_{i,h}=\frac{1}{{N}_{{{\rm{init}}}}}\sum\limits_{j=1}^{{N}_{{{\rm{init}}}}}\left\{1-\frac{({{{{\bf{B}}}}}_{i,j,h,0}\cap {{{{\bf{B}}}}}_{i,j,h,50})}{({{{{\bf{B}}}}}_{i,j,h,0}\cup {{{{\bf{B}}}}}_{i,j,h,50})}\right\}$$

where ${{{{\bf{B}}}}}_{i,j,h,0}$ and ${{{{\bf{B}}}}}_{i,j,h,50}$ denoted the family-level taxonomic membership of the community under the 0% and 50% (median) abundance quantiles of the focal genus in the host plant background *h*, for each initial community state *j* ∈ $\left\{{{\mathrm{1,2}}},\ldots,{N}_{{\mbox{init}}}\right\}$ (${N}_{{\mbox{init}}}$ = 20,000; Fig. 1g and Supplementary Figs. S39a, b). Accordingly, $1-\frac{({{{{\bf{B}}}}}_{i,j,h,0}\cap {{{{\bf{B}}}}}_{i,j,h,50})}{({{{{\bf{B}}}}}_{i,j,h,0}\cup {{{{\bf{B}}}}}_{i,j,h,50})}$ corresponds to the Jaccard dissimilarity between the community destinations (i.e., the basin bottoms reached) under the two abundance assumptions.

In addition, we evaluated how the evenness of assembly landscapes changed along the abundance gradient of the focal genus (Fig. 1e and Supplementary Figs. S39c, d). Specifically, across the 20,000 steepest-descent simulations, changes in the evenness of destination frequencies were evaluated as follows:

8 $$\Delta {{{{\rm{evenness}}}}}_{i,h}={H}_{i,h,50}-{H}_{i,h,0} \\=-\sum\limits_{m=1}^{{K}_{i,h,50}}{p}_{i,m,h,50}\log {p}_{i,m,h,50} \\ -\left[-\sum\limits_{m=1}^{{K}_{i,h,0}}{p}_{i,m,h,0}\log {p}_{i,m,h,0}\right]$$

where ${H}_{i,h,50}$ and ${H}_{i,h,0}$ denote the Shannon entropy of the proportions of initial states attracted to specific basins under the 50% and 0% quantile abundance assumptions of focal genus *i*, respectively (Fig. 1g). The proportions of initial community states [*j* ∈ $\left\{{{\mathrm{1,2}}},\ldots,{N}_{{\mbox{init}}}\right\}$] attracted to each basin *m* in host plant background *h* under the two abundance conditions were denoted as ${p}_{i,m,h,50}$ and ${p}_{i,m,h,0}$. The values ${K}_{i,h,50}$ and ${K}_{i,h,0}$ indicate the numbers of basins under the respective conditions.

To standardize the estimated species’ effects on assembly landscape and evaluate their statistical significance, we performed null-model simulation tests. Specifically, we repeated the sensitivity analysis described above, but with randomly shuffled relative abundance of the focal genus using two different approaches (10,000 permutations each). In the randomization, shuffling was performed among samples sharing the same host plant. These indices $\Delta {{{{\rm{topography}}}}}_{i,h}$ and $\Delta {{{{\rm{evenness}}}}}_{i,h}$ were then standardized, respectively, based on the randomization as follows:

9 $${{Z-{\rm{standardized}}}}\,\Delta {{{{\rm{topography}}}}}_{i,h} \\=\frac{\Delta {{{{\rm{topography}}}}}_{i,h,{{{\rm{obs}}}}}-{{{\rm{Mean}}}}\left(\Delta {{{{\rm{topography}}}}}_{i,h,{{{\rm{rand}}}}}\right)}{{{{\rm{SD}}}}\left(\Delta {{{{\rm{topography}}}}}_{i,h,{{{\rm{rand}}}}}\right)}$$

10 $${{Z-{\rm{standardized}}}}\Delta {{{{\rm{evenness}}}}}_{i,h} \\=\frac{\Delta {{{{\rm{evenness}}}}}_{i,h,{{{\rm{obs}}}}}-{{{\rm{Mean}}}}\left(\Delta {{{{\rm{evenness}}}}}_{i,h,{{{\rm{rand}}}}}\right)}{{{{\rm{SD}}}}\left(\Delta {{{{\rm{evenness}}}}}_{i,h,{{{\rm{rand}}}}}\right)}$$

where $\Delta {{{{\rm{topography}}}}}_{i,h,{{{\rm{obs}}}}}$ was the value from the original dataset (observation), and ${{{\rm{Mean}}}}\left(\Delta {{{{\rm{topography}}}}}_{i,h,{{{\rm{rand}}}}}\right)$ and ${{{\rm{SD}}}}\left(\Delta {{{{\rm{topography}}}}}_{i,h,{{{\rm{rand}}}}}\right)$ were the mean and standard deviation, respectively, of the $\Delta {{{{\rm{topography}}}}}_{i,h}$ of the randomized datasets. Similarly, $\Delta {{{{\rm{evenness}}}}}_{i,h,{obs}}$, ${{{\rm{Mean}}}}\left(\Delta {{{{\rm{evenness}}}}}_{i,h,{{{\rm{rand}}}}}\right)$, and ${{{\rm{SD}}}}\left(\Delta {{{{\rm{evenness}}}}}_{i,h,{{{\rm{rand}}}}}\right)$ were obtained from the original and randomized datasets. Empirical *P* values for the two indices were separately corrected for multiple testing using the Benjamini–Hochberg method^94^. Corrections were applied separately to fungal and prokaryotic assembly landscapes by jointly considering all focal genera across the six host backgrounds.

We then examined the consistency of the highlighted genera across inferences obtained under a series of alternative analytical settings (see Supplementary Notes 10–13 for details). First, we performed a supplementary exploration of keystone microbes by compiling genus-level community data for the assembly landscape analysis (Supplementary Fig. S40 and Supplementary Tables S12–S13). Second, we calculated ${\Delta {{{\rm{topography}}}}}_{i,{h}}$ and ${\Delta {{{\rm{evenness}}}}}_{i,{h}}$ values for alternative combinations of abundance assumptions (e.g., 0% vs 25% and 0% vs 75% abundance quantiles; Supplementary Figs. S41–S43 and Supplementary Tables S14–S17). Third, a supplementary calculation of ${\Delta {{{\rm{topography}}}}}_{i,{h}}$ and ${\Delta {{{\rm{evenness}}}}}_{i,{h}}$ scores was performed based on an alternative randomization-block setting (shuffling among samples with both the same host plant identity and spatial sampling block; Supplementary Figs. S44–S45). We also evaluated uncertainty in the calculation of ${\Delta {{{\rm{topography}}}}}_{i,{h}}$ and ${\Delta {{{\rm{evenness}}}}}_{i,{h}}$ based on bootstrap resampling of the community data (Supplementary Fig. S46).

### Assembly landscape changes along abundance gradients

In addition to the analysis based on $\Delta {{{{\rm{topography}}}}}_{i,{h}}$ and $\Delta {{{{\rm{evenness}}}}}_{i,h}$ metrics, which represented assembly landscape changes between two abundance levels of focal genus *i*, we obtained detailed inferences of assembly landscape reorganization along the entire range of the abundance gradient. For each combination of genus *i* and host plant background *h*, a steepest-descent simulation was performed with the same 20,000 initial states at 32 equally spaced relative abundance levels, ranging from absence (0% quantile) to the maximum observed abundance (100% quantile) of genus *i*. At each of the first, 16th, and 32nd levels within the abundance gradient, reconstructed basins were characterized within the PCoA community space defined in Figs. 2 and 3. To illustrate the similarity of the inferred basins, the first three axes of the PCoA were mapped respectively onto an RGB color space (axis 1, green, axis 2, blue; axis 3, red; Supplementary Figs. S47–S49). In addition, to track changes in the evenness of assembly landscape architecture along the abundance gradient of the focal genus, the Shannon entropy of basin frequencies was calculated at each of the 32 abundance steps. As the analysis along the entire range of abundance gradient is computationally intensive, it was performed for fungal and prokaryotic genera with the strongest impacts on assembly landscape architecture. Specifically, we targeted the two genera with the highest significant *z*-standardized $\Delta {{{{\rm{topography}}}}}_{i,{h}}$, values and those with the largest absolute values of *z*-standardized $\Delta {{{{\rm{evenness}}}}}_{i,h}$ under each host-plant background.

### Benchmark analysis: assembly landscape reconstruction

To evaluate how community complexity and sample size affect the uncertainty and reliability of energy landscape reconstruction, we generated benchmark community datasets using generalized Lotka–Volterra (gLV) models, which are widely used to describe diverse ecological dynamics including oscillatory and chaotic dynamics as well as convergence to equilibria^97^. In these simulations, species richness (20–50 species) and sample size (20–1200 replicate community samples) were systematically varied. The population dynamics of benchmark communities were described as

11 $$\frac{d{N}_{i}}{{dt}}={N}_{i}\left({r}_{i}+\sum\limits_{j=1}^{S}{A}_{{ij}}{N}_{j}\right)+{\epsilon }_{i}\left(t\right)$$

where S (= 20, 30, 40, or 50) denotes the number of species, ${N}_{i}$ is the abundance of species i, ${r}_{i}$ is its intrinsic growth rate, and ${A}_{{ij}}$ represents the effect of species j on species i. Furthermore, to assess the performance of assembly landscape inference from observational data influenced by immigration, emigration, and external disturbances, stochastic perturbations were introduced at each Euler integration step as

12 $${\epsilon }_{i}\left(t\right)={\eta }_{i}\left(t\right){\xi }_{i}\left(t\right)$$

where ${\eta }_{i}\left(t\right){{{\mathscr{\sim }}}}{{{\mathscr{N}}}}\left({{\mathrm{0,0.01}}}\right)$ represents small symmetric fluctuations (mean = 0; standard deviation = 0.01), and ${\xi }_{i}(t)\sim {\mbox{LogNormal}}\left({{\mathrm{0,2}}}\right)$ introduces positively skewed multiplicative variability in perturbation magnitude. All random variables were independently sampled, producing stochastic perturbations with variable intensity^38^. When stochastic perturbations resulted in negative species abundances, the values were truncated to zero. Initial species abundances and intrinsic growth rates were drawn from a uniform distribution between 0 and 1. Interaction coefficients were defined as

13 $${A}_{{ij}}={Z}_{{ij}}{L}_{{ij}}$$

where ${Z}_{{ij}}{{{\mathscr{\sim }}}}{{{\mathscr{N}}}}(-{{\mathrm{0.1,0.3}}})$ and ${L}_{{ij}}\sim {\mbox{LogNormal}}({{\mathrm{0,1}}})$. The log-normal distribution was introduced to generate a heavy-tailed distribution of interaction strengths, allowing some species to exert disproportionately strong effects on others, as implemented in a previous gLV study^38^. This parameterization generated heterogeneous interaction strengths while slightly biasing interactions toward negative values, thereby suppressing unbounded population growth. The diagonal entries of **A** were fixed to –1. Simulations exhibiting unbounded population growth were excluded from subsequent analyses. Interaction sparsity was controlled by fixing connectance (the proportion of non-zero entries in ${A}_{{ij}}$) at 0.2. Specifically, ${A}_{{ij}}$ was set to ${Z}_{{ij}}{L}_{{ij}}$ with probability 0.2 and to zero with probability 0.8.

For each community size setting (*S* = 20, 30, 40, or 50), three independent parameter sets were generated. For each parameter set, we first evaluated the number of discrete terminal community states that could be reached from randomly initialized community compositions. To this end, we performed simulations from 100 independently generated initial abundance vectors and defined the terminal community composition of each simulation as the mean abundance vector over t=95–100. This procedure was repeated 15 times for each parameter set to account for uncertainty in the estimated number of clusters. Each simulation was run using the first-order Euler method ($\Delta t={10}^{-3}$) until t=100, which was sufficient to allow initial transient dynamics to decay in most simulations. The simulated abundance data were then converted into binarized community-state matrices required for energy landscape analysis. To determine an appropriate binarization threshold, we evaluated 30 candidate thresholds evenly spaced between the 10% and 90% abundance quantiles. For each threshold, Bray–Curtis dissimilarities calculated from abundance data and Jaccard dissimilarities calculated from binarized data were compared. The threshold maximizing Kendall’s rank correlation coefficient between Bray–Curtis and Jaccard dissimilarities was selected so that binarization preserved the underlying community structure (Supplementary Fig. S6a). The resulting binarized terminal community states were subjected to hierarchical clustering based on Jaccard dissimilarity using Ward’s method. The number of clusters varied from 2 to 10, and the optimal number of clusters was selected using the silhouette method (Supplementary Fig. S50a). These clusters were used as an approximation of the number of discrete terminal community states, or putative attractor-like outcomes, that could arise under each gLV parameter set.

Next, to evaluate how sample size affects the accuracy and stability of energy landscape reconstruction for these gLV-generated community dynamics, independent stochastic community dynamics were simulated repeatedly for each parameter set until the defined number of community samples ($20,\,50,\,100,\,150,\,200,\,300,\,500,\,800,$ or 1200) was obtained. This dataset-generation procedure was repeated ten times with different initial species-abundance conditions. Because natural communities are not always observed at a converged state, community compositions during transitional phases or other non-equilibrium dynamics may also appear in observational datasets. To reflect cross-sectional sampling and to reconstruct assembly landscapes over a broad state space, we drew one time point uniformly at random from each independent simulation replicate, yielding one community composition per replicate. Because basins in an assembly landscape are defined as high-probability regions of the state-occupancy distribution rather than strict dynamical attractors, this strategy captures both frequently visited compositions near stable regions and low-frequency compositions arising from transitional phases or other non-equilibrium dynamics. Collections of these sampled community states were used to generate datasets comprising 20–1200 replicate communities. The resulting abundance datasets were binarized using the same threshold-selection procedure as described above. These datasets allowed us to evaluate how increasing sample size affects the stability and uncertainty of reconstructed assembly landscapes. Because stochastic perturbations and heterogeneous interactions among species were incorporated in the gLV model, the simulated communities did not necessarily converge to a single equilibrium point, allowing diverse community states to be sampled across simulations.

Energy landscape analysis was subsequently applied to each binarized dataset, and basin bottoms were identified using a steepest-descent procedure initiated from 150,000 randomly generated community states (Supplementary Fig. S50b). To quantify uncertainty in reconstructed landscape architecture, basin-frequency distributions inferred from the ten replicate analyses (each corresponding to an independent gLV simulation) were compared using Jensen–Shannon divergence,

14 $${D}_{{ij}}=\sqrt{\frac{1}{2}{\sum }_{k=1}^{n}\left[{p}_{i}(k)\log \frac{{p}_{i}(k)}{{{{{\rm{\mu }}}}}_{{ij}}(k)}+{p}_{j}(k)\log \frac{{p}_{j}(k)}{{{{{\rm{\mu }}}}}_{{ij}}(k)}\right]}$$

where ${p}_{i}(k)$ and ${p}_{j}(k)$ denote the relative frequency of basin k in replicates i and j, respectively, and

15 $${{{{\rm{\mu }}}}}_{{ij}}\left(k\right)=\frac{1}{2}\left\{{p}_{i}\left(k\right)+{p}_{j}(k)\right\}$$

We then examined how the Jensen–Shannon divergence, which represents inconsistency among inferred landscapes, decreased with increasing numbers of community samples (Supplementary Fig. S50c).

### Benchmark analysis: keystone exploration

Using the gLV simulation platform, we conducted a benchmark analysis to evaluate whether the proposed keystone-exploration framework can prioritize species exerting strong community-level influences (Supplementary Fig. S6). Community datasets were generated from generalized Lotka–Volterra (gLV) models with 80 species under three levels of interaction sparsity (connectance = 0.2, 0.3, and 0.4). For each sparsity level, 50 independent parameter sets were generated, yielding a total of 150 candidate model systems representing diverse interaction structures. To prevent unstable population dynamics in relatively dense interaction networks, the variance of ${Z}_{{ij}}$, a component of ${A}_{{ij}}$ (13), was slightly reduced [${Z}_{{ij}}{{{\mathscr{\sim }}}}{{{\mathscr{N}}}}(-0.1,\,0.2)$]. Before running the benchmark simulations, we performed short preliminary simulations (until t=30) for each parameter set to verify that the population dynamics remained bounded.

For each parameter set, we performed 200 replicate simulations starting from independently generated initial abundance conditions and simulated community dynamics until t=100. One time point was randomly sampled from each replicate to construct a dataset of 200 community compositions (Supplementary Fig. S6a). These datasets were converted into binarized community-state matrices using the same thresholding procedure described above. Species with extremely high (>90%) or low (<10%) occurrence frequencies were removed because they provided limited information for energy landscape inference.

Because the number of informative species (i.e., species whose occurrence frequencies fell between 10% and 90%) varied among parameter sets, we next selected model systems suitable for benchmarking. Specifically, for each connectance level, five parameter sets (out of 50 tested) retaining the greatest number of species after filtering were selected for further analyses (15 model systems in total; Supplementary Fig. S6b). This selection ensured that assembly landscapes were inferred under conditions comparable to empirical datasets with sufficiently high community complexity.

Using the selected model systems, we applied the energy landscape framework sequentially to each species, treating the abundance of a focal species as an explanatory variable. *Z*-standardized Δtopography values were calculated for all 80 species based on comparisons between the 0% (absence) and 50% (median) abundance quantiles (Fig. 1g). Although simulated dynamics could reflect local properties of the assembly landscape (e.g., basin transitions along simulated trajectories), deriving landscape-level descriptors corresponding to Δevenness is less straightforward. Therefore, we focused on Δtopography in this benchmark analysis.

To obtain an independent measure of each species’ influence on community dynamics, we conducted additional simulations in which a focal species was either present in the initial community (initial abundance = 0.5) or removed (abundance fixed at zero), while keeping the initial abundances of all other species identical (200 replicate simulations per species). For both scenarios, mean abundances of all non-focal species were calculated over the time window *t* = 95–100. Community dissimilarity between the two scenarios was quantified using Bray–Curtis dissimilarity and Jaccard dissimilarity (after binarization with the selected threshold).

For each model system, the correspondence between Δtopography and the independently estimated community dissimilarity following species removal was evaluated using Kendall’s rank correlation tests (Supplementary Figs. S7–S8). *P* values across the 15 models were corrected for multiple comparisons within each dissimilarity metric using the Benjamini–Hochberg false discovery rate (FDR) procedure^94^.

In addition, to assess the robustness of Δtopography to alternative simulation settings, we performed two supplementary gLV-based validations (Supplementary Figs. S9–S10; see Supplementary Note 2 for details). First, to isolate the effect of stochastic perturbations, we repeated the entire benchmark pipeline for the same selected model systems after removing the stochastic perturbation term and re-evaluated the correspondence between *z*-standardized Δtopography (3,000 permutations) and the community dissimilarity induced by focal-species removal (Supplementary Fig. S9). Second, to test performance in systems that exhibit perturbation-driven switching among alternative community states after an initial transient, we randomly generated gLV parameter sets (connectance = 0.2) using a modified simulation scheme designed to promote multistable dynamics while preventing divergence. Specifically, the mean and variance of ${Z}_{{ij}}$ (a component of ${A}_{{ij}}$ (13)) were set to 0 and 0.1, respectively, and the diagonal entries of **A** were fixed to –6. In these simulations, stochastic perturbations were applied only to species with non-zero abundance, whereas immigration of absent species was modeled separately. At each integration step, each absent species immigrated with probability 0.002, in which case its abundance was set to 0.02. Species with abundances below 0.01 were treated as extinct and set to zero. Using this modified simulation scheme, we screened randomly generated parameter sets and selected three that displayed switching dynamics among distinct community states after the initial transient. For each system, we simulated trajectories until *t* = 3000 and constructed datasets by randomly sampling time points from *t* = 101–3000 to capture post-transient dynamics. We then analyzed these datasets using the same benchmark pipeline as in the primary evaluation (Supplementary Fig. S10).

### Benchmark analysis: interaction properties and basin number

To examine the relationship between interspecific interaction networks and the structure of the reconstructed assembly landscapes, we analyzed the 15 binarized community datasets generated from the gLV parameter sets used in the keystone-exploration benchmark analysis. For each dataset, we performed energy landscape analysis and estimated the number of basins using a steepest-descent procedure initiated from 150,000 randomly generated community states. For each corresponding gLV parameter set, we calculated two global properties of the interaction matrix: connectance levels (0.2, 0.3, and 0.4) and mean interaction strength, defined as the mean absolute value of nonzero off-diagonal interaction coefficients in the interaction matrix (Supplementary Fig. S51). We evaluated whether the number of inferred basins differed among connectance levels using Kruskal–Wallis test. The relationship between the number of inferred basins and mean interaction strength was assessed using Kendall’s rank correlation test.

### Candidate keystones and mycorrhizal states in Europe

To elucidate the ecological roles of the keystone taxa highlighted by the proposed framework, we analyzed their associations with variation in mycorrhizal symbiosis using a complementary root microbiome dataset collected across a broad geographic range in Europe spanning central Italy (42.08°N, 9.82°E) to northern Norway (69.46°N, 30.02°E)^36^. The dataset comprised root-associated fungal and bacterial communities from arbuscular mycorrhizal plants (*Sorbus aucuparia*, *Sorbus domestica*, and *Sorbus torminalis*), an ectomycorrhizal plant (*Betula pendula*), and dual-mycorrhizal plants (*Alnus glutinosa* and *Alnus incana*), together with measurements of root colonization rates by mycorrhizal fungi and environmental covariates^36^. Fungal and prokaryotic community compositions in roots were characterized by amplicon sequencing of the internal transcribed spacer (ITS) region and the 16S rRNA gene, respectively. In addition to the root microbiome data, measurements of root colonization by mycorrhizal fungi were obtained by microscopic observation. Because of fundamental differences in hyphal morphology between mycorrhizal types, colonization was quantified as the percentage of root length colonized for arbuscular mycorrhizal fungi and as the percentage of root tips colonized for ectomycorrhizal fungi, respectively^36^. Arbuscular mycorrhizal colonization rates were assessed for arbuscular mycorrhizal (*Sorbus*) and dual-mycorrhizal (*Alnus*) plants (219 plant individuals in total), whereas ectomycorrhizal colonization rates were obtained for ectomycorrhizal (*Betula*) and dual-mycorrhizal (*Alnus*) plants (218 plant individuals in total). Overall, this European dataset enabled statistical evaluation of each fungal or prokaryotic taxon in relation to mycorrhizal symbiosis.

We constructed a series of generalized linear mixed models (GLMMs), using the root colonization rate of either arbuscular or ectomycorrhizal fungi as the response variable and the abundance of each candidate keystone taxon (Fig. 4) as the main explanatory variable, together with environmental covariates selected based on the original study^36^. Specifically, models for arbuscular mycorrhizal colonization employed a logit link-function with binomial errors and included soil pH as an additional fixed effect and site as a random effect. For ectomycorrhizal colonization, gravimetric soil moisture was included instead of soil pH, with site again treated as a random effect. The Benjamini–Hochberg method for multiple testing^94^ was applied separately to the analyses on arbuscular mycorrhizal and ectomycorrhizal symbioses.

### Reporting summary

Further information on research design is available in the Nature Portfolio Reporting Summary linked to this article.

## Supplementary information

Supplementary Information

Description of Additional Supplementary Files

Supplementary Data 1

Reporting Summary

Transparent Peer Review file

## Source data

Source Data

## Data Availability

DNA sequence data in this study have been deposited in the DNA Data Bank of Japan database under accession numbers PRJDB17520 (Fungi and plants; https://ddbj.nig.ac.jp/search/entry/bioproject/PRJDB17520) and PRJDB37499 (Prokaryotes; https://ddbj.nig.ac.jp/search/entry/bioproject/PRJDB37499). This study also used a publicly available root microbiome dataset (Supplementary Data 1 and 2 in the original publication) collected across Europe^36^. These data are available in the original publication and the Zenodo open data repository under https://doi.org/10.5281/zenodo.10203817. Source data are provided with this paper.

## Code availability

The computer code is available from the GitHub repository (https://github.com/ngchngch/Assembly_landscape_of_the_forest_root_microbiome) and has been archived in Zenodo^98^ under https://doi.org/10.5281/zenodo.21888542.

## References

[1] Trivedi, P., Leach, J. E., Tringe, S. G., Sa, T. & Singh, B. K. Plant–microbiome interactions: from community assembly to plant health. *Nat. Rev. Microbiol.* **18**, 607–621 (2020). 10.1038/s41579-020-0412-1 32788714

[2] Liu, H., Brettell, L. E., Qiu, Z. & Singh, B. K. Microbiome-mediated stress resistance in plants. *Trends Plant Sci.* **25**, 733–743 (2020). 10.1016/j.tplants.2020.03.014 32345569

[3] Puginier, C., Keller, J. & Delaux, P.-M. Plant–microbe interactions that have impacted plant terrestrializations. *Plant Physiol.* **190**, 72–84 (2022). 10.1093/plphys/kiac258 PMC9434271 35642902

[4] Toju, H. et al. Core microbiomes for sustainable agroecosystems. *Nat. Plants* **4**, 247–257 (2018). 10.1038/s41477-018-0139-4 29725101

[5] Banerjee, S., Schlaeppi, K. & van der Heijden, M. G. A. Keystone taxa as drivers of microbiome structure and functioning. *Nat. Rev. Microbiol.* **16**, 567–576 (2018). 10.1038/s41579-018-0024-1 29789680

[6] Niu, B., Paulson, J. N., Zheng, X. & Kolter, R. Simplified and representative bacterial community of maize roots. *Proc. Natl. Acad. Sci. USA* **114**, E2450–E2459 (2017). 10.1073/pnas.1616148114 PMC5373366 28275097

[7] Yang, N. et al. Interspecific interactions facilitate keystone species in a multispecies biofilm that promotes plant growth. *ISME J.* **18**, wrae012 (2024). 10.1093/ismejo/wrae012 PMC10938371 38365935

[8] Ma, C.-Y. et al. Fungal endophyte promotes plant growth and disease resistance of *Arachis hypogaea* L. by reshaping the core root microbiome under monocropping conditions. *Microbiol. Res.* **277**, 127491 (2023). 10.1016/j.micres.2023.127491 37769598

[9] Power, M. E. et al. Challenges in the quest for keystones: identifying keystone species is difficult—but essential to understanding how loss of species will affect ecosystems. *BioScience* **46**, 609–620 (1996).

[10] Paine, R. T. A conversation on refining the concept of keystone species. *Conserv. Biol.* **9**, 962–964 (1995).

[11] Philippot, L., Raaijmakers, J. M., Lemanceau, P. & Van Der Putten, W. H. Going back to the roots: the microbial ecology of the rhizosphere. *Nat. Rev. Microbiol.* **11**, 789–799 (2013). 10.1038/nrmicro3109 24056930

[12] Scheffer, M., Carpenter, S., Foley, J. A., Folke, C. & Walker, B. Catastrophic shifts in ecosystems. *Nature* **413**, 591–596 (2001). 10.1038/35098000 11595939

[13] Shade, A. et al. Fundamentals of microbial community resistance and resilience. *Front. Microbiol.* **3**, 417 (2012). 10.3389/fmicb.2012.00417 PMC3525951 23267351

[14] Suzuki, K., Nakaoka, S., Fukuda, S. & Masuya, H. Energy landscape analysis elucidates the multistability of ecological communities across environmental gradients. *Ecol. Monogr.* **91**, e01469 (2021).

[15] Fujita, H. et al. Alternative stable states, nonlinear behavior, and predictability of microbiome dynamics. *Microbiome* **11**, 63 (2023). 10.1186/s40168-023-01474-5 PMC10052866 36978146

[16] Holling, C. S. Resilience and stability of ecological systems. *Annu. Rev. Ecol. Syst.* **4**, 1–23 (1973).

[17] Scheffer, M., Carpenter, S. R., Dakos, V. & Van Nes, E. H. Generic indicators of ecological resilience: inferring the chance of a critical transition. *Annu. Rev. Ecol. Evol. Syst.* **46**, 145–167 (2015).

[18] Beisner, B., Haydon, D. & Cuddington, K. Alternative stable states in ecology. *Front. Ecol. Environ.* **1**, 376–382 (2003).

[19] Scheffer, M. & Carpenter, S. R. Catastrophic regime shifts in ecosystems: linking theory to observation. *Trends Ecol. Evol.* **18**, 648–656 (2003).

[20] Hastings, A. et al. Transient phenomena in ecology. *Science* **361**, eaat6412 (2018). 10.1126/science.aat6412 30190378

[21] Morozov, A. et al. Long transients in ecology: theory and applications. *Phys. Life Rev.* **32**, 1–40 (2020). 10.1016/j.plrev.2019.09.004 31982327

[22] Zhang, K. & Nakaoka, S. An energy landscape approach reveals the potential key bacteria contributing to the development of inflammatory bowel disease. *PLoS ONE* **19**, e0302151 (2024). 10.1371/journal.pone.0302151 PMC11182530 38885178

[23] Kadoya, T., Suzuki, K. & Terui, A. Linking energetic instability to compositional changes in biological communities. *Proc. Natl. Acad. Sci. USA* **122**, e2422701122 (2025). 10.1073/pnas.2422701122 PMC12054833 40258153

[24] Fujita, H., Yoshida, S., Suzuki, K. & Toju, H. Alternative stable states of microbiome structure and soil ecosystem functions. *Environ. Microbiome* **20**, 28 (2025). 10.1186/s40793-025-00688-4 PMC11887376 40050988

[25] Teste, F. P., Jones, M. D. & Dickie, I. A. Dual-mycorrhizal plants: their ecology and relevance. *New Phytol.* **225**, 1835–1851 (2020). 10.1111/nph.16190 31514244

[26] Noguchi, M. & Toju, H. Mycorrhizal and endophytic fungi structure forest below-ground symbiosis through contrasting but interdependent assembly processes. *Environ. Microbiome* **19**, 84 (2024). 10.1186/s40793-024-00628-8 PMC11531145 39488693

[27] Watanabe, T. et al. Energy landscapes of resting-state brain networks. *Front. Neuroinform.* **8**, 22 (2014). 10.3389/fninf.2014.00012 PMC3933812 24611044

[28] Masuda, N., Islam, S., Aung, S. T. & Watanabe, T. Energy landscape analysis based on the Ising model: tutorial review. *PLoS Complex Syst.* **2**, e0000039 (2025). 10.1371/journal.pcsy.0000039 PMC13215579 42211873

[29] Coyte, K. Z., Schluter, J. & Foster, K. R. The ecology of the microbiome: networks, competition, and stability. *Science* **350**, 663–666 (2015). 10.1126/science.aad2602 26542567

[30] Serván, C. A. & Allesina, S. Tractable models of ecological assembly. *Ecol. Lett.* **24**, 1029–1037 (2021). 10.1111/ele.13702 33773006

[31] Capitán, J. A., Cuesta, J. A. & Bascompte, J. Statistical mechanics of ecosystem assembly. *Phys. Rev. Lett.* **103**, 168101 (2009). 10.1103/PhysRevLett.103.168101 19905727

[32] Jumpponen, A. & Trappe, J. M. Dark septate endophytes: a review of facultative biotrophic root-colonizing fungi. *New Phytol.* **140**, 295–310 (1998). 10.1046/j.1469-8137.1998.00265.x 33862835

[33] Grünig, C. R., Queloz, V. & Sieber, T. N. Structure of diversity in dark septate endophytes: from species to genes. in *Endophytes of Forest Trees: Biology and Applications* (eds. Pirttilä, A. M. & Frank, A. C.) 3–30 10.1007/978-94-007-1599-8_1 (Springer Netherlands, 2011).

[34] Ozimek, E. & Hanaka, A. *Mortierella* species as the plant growth-promoting fungi present in the agricultural soils. *Agriculture* **11**, 7 (2021).

[35] Leveau, J. H. J., Uroz, S. & De Boer, W. The bacterial genus *Collimonas*: mycophagy, weathering and other adaptive solutions to life in oligotrophic soil environments. *Environ. Microbiol.* **12**, 281–292 (2010). 10.1111/j.1462-2920.2009.02010.x 19638176

[36] Netherway, T. et al. Pervasive associations between dark septate endophytic fungi with tree root and soil microbiomes across Europe. *Nat. Commun.* **15**, 159 (2024). 10.1038/s41467-023-44172-4 PMC10761831 38167673

[37] Amit, G. & Bashan, A. Top-down identification of keystone taxa in the microbiome. *Nat. Commun.* **14**, 3951 (2023). 10.1038/s41467-023-39459-5 PMC10319726 37402745

[38] Wang, X.-W. et al. Identifying keystone species in microbial communities using deep learning. *Nat. Ecol. Evol.* **8**, 22–31 (2024). 10.1038/s41559-023-02250-2 PMC12125608 37974003

[39] Garza, D. R., Gonze, D. & Faust, K. Keystone concept revisited: insights into microbial community dynamics and control. *Nat. Rev. Microbiol*. 10.1038/s41579-025-01266-8 (2026). 41478866

[40] Agler, M. T. et al. Microbial hub taxa link host and abiotic factors to plant microbiome variation. *PLoS Biol.* **14**, e1002352 (2016). 10.1371/journal.pbio.1002352 PMC4720289 26788878

[41] Floc’h, J.-B. et al. Inter-kingdom networks of canola microbiome reveal *Bradyrhizobium* as keystone species and underline the importance of bulk soil in microbial studies to enhance canola production. *Microb. Ecol.* **84**, 1166–1181 (2022). 10.1007/s00248-021-01905-6 34727198

[42] Berry, D. & Widder, S. Deciphering microbial interactions and detecting keystone species with co-occurrence networks. *Front. Microbiol.* **5**, 219 (2014). 10.3389/fmicb.2014.00219 PMC4033041 24904535

[43] Sun, X., Zhao, Y. & Ding, G. Morphogenesis and metabolomics reveal the compatible relationship among *Suillus bovinus*, *Phialocephala fortinii*, and their co-host, *Pinus massoniana*. *Microbiol. Spectr.* **11**, e01453–23 (2023). 10.1128/spectrum.01453-23 PMC10580909 37676026

[44] Newsham, K. K. A meta-analysis of plant responses to dark septate root endophytes. *New Phytol.* **190**, 783–793 (2011). 10.1111/j.1469-8137.2010.03611.x 21244432

[45] Smith, S. E. & Read, D. J. *Mycorrhizal Symbiosis* (Academic Press, 2008).

[46] Tedersoo, L. & Bahram, M. Mycorrhizal types differ in ecophysiology and alter plant nutrition and soil processes. *Biol. Rev. Camb. Philos. Soc.* **94**, 1857–1880 (2019). 10.1111/brv.12538 31270944

[47] Fukami, T. Historical contingency in community assembly: integrating niches, species pools, and priority effects. *Annu. Rev. Ecol. Evol. Syst.* **46**, 1–23 (2015).

[48] Lopez, R. O., Chiocchio, V. M., Ruscitti, M. F., Taborda, C. P. & Saparrat, M. C. N. Towards a better understanding of melanins from dark septate endophytes (DSEs): their variability, synthesis pathways and biological roles. *J. Soil Sci. Plant Nutr.* **24**, 1650–1664 (2024).

[49] Netherway, T. & Bahram, M. Melanized root-associated fungi: key players in plant–soil systems. *Trends Microbiol.* **32**, 1190–1199 (2024). 10.1016/j.tim.2024.06.006 38987052

[50] Schneider-Maunoury, L. et al. Two ectomycorrhizal truffles, *Tuber melanosporum* and *T. aestivum*, endophytically colonise roots of non-ectomycorrhizal plants in natural environments. *New Phytol.* **225**, 2542–2556 (2020). 10.1111/nph.16321 31733103

[51] Laurent-Webb, L. et al. Endophytic and ectomycorrhizal, an overlooked dual ecological niche? Insights from natural environments and *Russula* species. *New Phytol*, **250**, 3946–3960 10.1111/nph.71175 (2026). PMC13193396 41992769

[52] Taschen, E. et al. Insight into the truffle brûlé: tripartite interactions between the black truffle (*Tuber melanosporum*), holm oak (*Quercus ilex*) and arbuscular mycorrhizal plants. *Plant Soil* **446**, 577–594 (2020).

[53] Toju, H., Tanabe, A. S. & Sato, H. Network hubs in root-associated fungal metacommunities. *Microbiome* **6**, 116 (2018). 10.1186/s40168-018-0497-1 PMC6015470 29935536

[54] Coluccia, M. & Besaury, L. Acidobacteria members harbour an abundant and diverse carbohydrate-active enzymes (cazyme) and secreted proteasome repertoire, key factors for potential efficient biomass degradation. *Mol. Genet. Genomics* **298**, 1135–1154 (2023). 10.1007/s00438-023-02045-x 37335345

[55] Gonçalves, O. S. et al. Insights into plant interactions and the biogeochemical role of the globally widespread Acidobacteriota phylum. *Soil Biol. Biochem.* **192**, 109369 (2024).

[56] Reis, V. M. & Teixeira, K. R. Nitrogen fixing bacteria in the family Acetobacteraceae and their role in agriculture. *J. Basic Microbiol.* **55**, 931–949 (2015). 10.1002/jobm.201400898 PMC7166518 25736602

[57] Jara-Servin, A., Mejia, G., Romero, M. F., Peimbert, M. & Alcaraz, L. D. Unravelling the genomic and environmental diversity of the ubiquitous. *Solirubrobacter. Environ. Microbiol.* **26**, e16685 (2024). 10.1111/1462-2920.16685 39147372

[58] Dedysh, S. N. & Ivanova, A. A. Planctomycetes in boreal and subarctic wetlands: diversity patterns and potential ecological functions. *FEMS Microbiol. Ecol.* **95**, fiy227 (2019). 10.1093/femsec/fiy227 30476049

[59] Anderson, I. et al. Genomics of aerobic cellulose utilization systems in actinobacteria. *PLoS ONE* **7**, e39331 (2012). 10.1371/journal.pone.0039331 PMC3377646 22723998

[60] Naylor, D., DeGraaf, S., Purdom, E. & Coleman-Derr, D. Drought and host selection influence bacterial community dynamics in the grass root microbiome. *ISME J.* **11**, 2691–2704 (2017). 10.1038/ismej.2017.118 PMC5702725 28753209

[61] Simmons, T. et al. Drought drives spatial variation in the millet root microbiome. *Front. Plant Sci.* **11**, 599 (2020). 10.3389/fpls.2020.00599 PMC7270290 32547572

[62] Zhang, F. et al. Root exudation of organic acid anions and recruitment of beneficial actinobacteria facilitate phosphorus uptake by maize in compacted silt loam soil. *Soil Biol. Biochem.* **184**, 109074 (2023).

[63] Challacombe, J. F. et al. Biological consequences of ancient gene acquisition and duplication in the large genome of *Candidatus* Solibacter usitatus Ellin6076. *PLoS ONE* **6**, e24882 (2011). 10.1371/journal.pone.0024882 PMC3174227 21949776

[64] Parsley, L. C. et al. Polyketide synthase pathways identified from a metagenomic library are derived from soil Acidobacteria. *FEMS Microbiol. Ecol.* **78**, 176–187 (2011). 10.1111/j.1574-6941.2011.01122.x 21539584

[65] Brewer, T. E., Handley, K. M., Carini, P., Gilbert, J. A. & Fierer, N. Genome reduction in an abundant and ubiquitous soil bacterium ‘*Candidatus* Udaeobacter copiosus’. *Nat. Microbiol.* **2**, 16198 (2016). 10.1038/nmicrobiol.2016.198 27798560

[66] Willms, I. M. et al. Globally abundant “*Candidatus* Udaeobacter” benefits from release of antibiotics in soil and potentially performs trace gas scavenging. *mSphere* **5**, e00186–20 (2020). 10.1128/mSphere.00186-20 PMC7343977 32641424

[67] Durán, P. et al. Microbial interkingdom interactions in roots promote *Arabidopsis* survival. *Cell* **175**, 973–983.e14 (2018). 10.1016/j.cell.2018.10.020 PMC6218654 30388454

[68] Liu, W. et al. Multikingdom interactions govern the microbiome in subterranean cultural heritage sites. *Proc. Natl. Acad. Sci. USA* **119**, e2121141119 (2022). 10.1073/pnas.2121141119 PMC9169738 35344401

[69] Wang, S., Bi, Y., Quan, W. & Christie, P. Growth and metabolism of dark septate endophytes and their stimulatory effects on plant growth. *Fungal Biol.* **126**, 674–686 (2022). 10.1016/j.funbio.2022.08.006 36116899

[70] Bi, Y., Wang, S., Song, Y., Tan, H. & Christie, P. Contributions of the dark septate endophyte extracellular metabolites to plant growth, metabolism and root-associated microbial community structure. *Plant Soil* **511**, 189–208 (2025).

[71] Gao, Z., Karlsson, I., Geisen, S., Kowalchuk, G. & Jousset, A. Protists: puppet masters of the rhizosphere microbiome. *Trends Plant Sci.* **24**, 165–176 (2019). 10.1016/j.tplants.2018.10.011 30446306

[72] Kudjordjie, E. N., Santos, S. S., Topalović, O. & Vestergård, M. Distinct changes in tomato-associated multi-kingdom microbiomes during Meloidogyne incognita parasitism. *Environ. Microbiome* **19**, 1–18 (2024). 10.1186/s40793-024-00597-y PMC11282865 39068487

[73] Malard, L. A. & Guisan, A. Into the microbial niche. *Trends Ecol. Evol.* **38**, 936–945 (2023). 10.1016/j.tree.2023.04.015 37236880

[74] Sun, T. et al. Interactions with native microbial keystone taxa enhance the biocontrol efficiency of *Streptomyces*. *Microbiome* **13**, 126 (2025). 10.1186/s40168-025-02120-y PMC12087250 40390122

[75] Finkel, O. M. et al. A single bacterial genus maintains root growth in a complex microbiome. *Nature* **587**, 103–108 (2020). 10.1038/s41586-020-2778-7 PMC10329457 32999461

[76] Toju, H., Tanabe, A. S., Yamamoto, S. & Sato, H. High-coverage ITS primers for the DNA-based identification of ascomycetes and basidiomycetes in environmental samples. *PLoS ONE* **7**, e40863 (2012). 10.1371/journal.pone.0040863 PMC3395698 22808280

[77] Kolter, A. & Gemeinholzer, B. Internal transcribed spacer primer evaluation for vascular plant metabarcoding. *Metabarcoding Metagenomics* **5**, e68155 (2021).

[78] Caporaso, J. G. et al. Global patterns of 16S rRNA diversity at a depth of millions of sequences per sample. *Proc. Natl. Acad. Sci. USA* **108**, 4516–4522 (2011). 10.1073/pnas.1000080107 PMC3063599 20534432

[79] Tanabe, A. S. & Toju, H. Two new computational methods for universal DNA barcoding: a benchmark using barcode sequences of bacteria, archaea, animals, fungi, and land plants. *PLoS ONE* **8**, e76910 (2013). 10.1371/journal.pone.0076910 PMC3799923 24204702

[80] Tanabe, A. S. Claident v0.9.2022.01.26: a software distributed by author: available online at: https://github.com/astanabe/Claident (2022).

[81] Martin, M. Cutadapt removes adapter sequences from high-throughput sequencing reads. *EMBnet. J.* **17**, 10–12 (2011).

[82] Callahan, B. J. et al. DADA2: High-resolution sample inference from Illumina amplicon data. *Nat. Methods* **13**, 581–583 (2016). 10.1038/nmeth.3869 PMC4927377 27214047

[83] R Core Team. *R: A Language and Environment for Statistical Computing* (R Foundation for Statistical Computing, 2024).

[84] Davis, N. M., Proctor, D. M., Holmes, S. P., Relman, D. A. & Callahan, B. J. Simple statistical identification and removal of contaminant sequences in marker-gene and metagenomics data. *Microbiome* **6**, 226 (2018). 10.1186/s40168-018-0605-2 PMC6298009 30558668

[85] Rognes, T., Flouri, T., Nichols, B., Quince, C. & Mahé, F. VSEARCH: a versatile open source tool for metagenomics. *PeerJ* **4**, e2584 (2016). 10.7717/peerj.2584 PMC5075697 27781170

[86] Abarenkov, K. et al. UNITE general FASTA release for eukaryotes 2. UNITE Community. 10.15156/BIO/2938070 (2023).

[87] Quast, C. et al. The SILVA ribosomal RNA gene database project: improved data processing and web-based tools. *Nucleic Acids Res.* **41**, D590–D596 (2013). 10.1093/nar/gks1219 PMC3531112 23193283

[88] Wang, Q., Garrity, G. M., Tiedje, J. M. & Cole, J. R. Naïve Bayesian classifier for rapid assignment of rRNA sequences into the new bacterial taxonomy. *Appl. Environ. Microbiol.* **73**, 5261–5267 (2007). 10.1128/AEM.00062-07 PMC1950982 17586664

[89] Sayers, E. W. et al. Database resources of the National Center for Biotechnology Information. *Nucleic Acids Res.* **50**, D20–D26 (2022). 10.1093/nar/gkab1112 PMC8728269 34850941

[90] Stecher, G., Tamura, K. & Kumar, S. Molecular Evolutionary Genetics Analysis (MEGA) for macOS. *Mol. Biol. Evol.* **37**, 1237–1239 (2020). 10.1093/molbev/msz312 PMC7086165 31904846

[91] Oksanen, J. et al. vegan: community ecology package. R package version 2.6-4 https://CRAN.R-project.org/package=vegan (2022).

[92] Blüthgen, N., Menzel, F., Hovestadt, T., Fiala, B. & Blüthgen, N. Specialization, constraints, and conflicting interests in mutualistic networks. *Curr. Biol.* **17**, 341–346 (2007). 10.1016/j.cub.2006.12.039 17275300

[93] Toju, H., Tanabe, A. S. & Ishii, H. S. Ericaceous plant–fungus network in a harsh alpine–subalpine environment. *Mol. Ecol.* **25**, 3242–3257 (2016). 10.1111/mec.13680 27136380

[94] Benjamini, Y. & Hochberg, Y. Controlling the false discovery rate: a practical and powerful approach to multiple testing. *J. R. Stat. Soc. Ser. B Methodol.* **57**, 289–300 (1995).

[95] Anderson, M. J. A new method for non-parametric multivariate analysis of variance. *Austral. Ecol.* **26**, 32–46 (2001).

[96] Anderson, M. J. Distance-based tests for homogeneity of multivariate dispersions. *Biometrics* **62**, 245–253 (2006). 10.1111/j.1541-0420.2005.00440.x 16542252

[97] Vano, J. A., Wildenberg, J. C., Anderson, M. B., Noel, J. K. & Sprott, J. C. Chaos in low-dimensional Lotka–Volterra models of competition. *Nonlinearity* **19**, 2391–2404 (2006).

[98] Noguchi, M., Suzuki, K., Fujita, H. & Toju, H. Code available online at Zenodo 10.5281/zenodo.21888542 (2026).

