# Supplementary material for: Statistical inference of keystone taxa reshaping the assembly rules of forest root microbiomes
Source: Nat Commun. 2026 Oct 28;17:10997. doi: 10.1038/s41467-026-78401-3 (PMC13716906; doi:10.1038/s41467-026-78401-3)
Supplement: Supplementary file 1 [file 41467_2026_78401_MOESM1_ESM.pdf]

Supplementary Information for

Statistical inference of keystone taxa reshaping the assembly rules of forest root microbiomes

Mikihito Noguchi[1-3]*, Kenta Suzuki[4,5], Hiroaki Fujita[2,6], and Hirokazu Toju[2,6]*

[1]Center for Ecological Research, Kyoto University, Otsu, Shiga 520-2133, Japan

[2]Laboratory of Ecosystems and Coevolution, Graduate School of Biostudies, Kyoto University, Kyoto 606-8501, Japan

[3]Environmental Management Research Institute, National Institute of Advanced Industrial Science and Technology (AIST), Tsukuba Center, 305-8569, Tsukuba, Japan

[4]Integrated Bioresource Information Division, BioResource Research Center, RIKEN, Tsukuba, Ibaraki 305-0074, Japan.

[5]Institute for Multidisciplinary Sciences, Yokohama National University, Yokohama, Kanagawa 240-8501, Japan.

[6]Center for Living Systems Information Science (CeLiSIS), Graduate School of Biostudies, Kyoto University, Kyoto 606-8501, Japan

*Corresponding authors:

Mikihito Noguchi: mikihito.noguchi@aist.go.jp

Hirokazu Toju: toju.hirokazu.4c@kyoto-u.ac.jp

Supplementary Note 1. Benchmark analysis: assembly landscape reconstruction

Based on datasets generated by generalized Lotka–Volterra (gLV) simulations, we examined how the results of energy landscape analyses varied among replicate community datasets arising from different initial species abundances. We also compared the number of basins inferred by energy landscape analysis with the number of distinct final community-composition groups observed in randomly initialized gLV simulations.

When the number of community samples was 300 or fewer, the number of detected basins varied among replicate datasets and, in some cases, differed from the number of terminal community-state clusters, depending on community complexity (i.e., species richness) and the gLV parameter settings (Supplementary Fig. S50a–b). In contrast, when the number of community samples exceeded 300, variation in the number of detected basins was reduced, and the detected number of basins often became closer to the number of distinct final community-composition groups observed in randomly initialized simulations (Supplementary Fig. S50a–b).

We further evaluated the consistency of energy landscape inference by focusing on the topography of reconstructed assembly landscapes. Differences in inferred basin occurrence frequencies among replicate datasets decreased with increasing sample size (Supplementary Fig. S50c). When the number of community samples reached 1,200, highly consistent basin frequencies (Jensen–Shannon divergence < 0.06) were obtained across combinations of community complexity and gLV parameter settings (Supplementary Fig. S50c).

These results indicate that, although the accuracy of energy landscape inference depends on species richness, interaction structure, and other model parameters, increasing sample size substantially improves the reliability of reconstructed landscape topography.

Supplementary Note 2. Benchmark analysis: keystone exploration

To benchmark the proposed keystone-exploration framework, we performed community dynamics simulations based on generalized Lotka–Volterra models comprising 80 species (Supplementary Fig. S6a; see Methods for details). Using the resulting community compositions, we calculated *z*-standardized Δtopography values. In addition, we evaluated both abundance-based and presence/absence-based community dissimilarities (Bray–Curtis and Jaccard dissimilarities) between simulations with and without each focal species, representing its influence on community destinations (i.e., basin bottoms reached) (Supplementary Fig. S6b).

Across the selected model systems, the *z*-standardized Δtopography was positively correlated with both dissimilarity metrics (Supplementary Figs. S7–S8). The proposed index captured not only changes in abundances but also changes in species membership, with significant correlations detected in at least three of five models at each level of interaction sparsity (Supplementary Figs. S7–S8).

These results indicate that the assembly landscape framework can recover species' influence on community destinations from observational community data.

To evaluate whether these results depend on the stochasticity and sampling scheme used in the primary benchmark, we performed two supplementary gLV-based validations (Supplementary Figs. S9–S10) while keeping the benchmark pipeline unchanged. First, we repeated the benchmark for the same selected model systems after removing the stochastic perturbation term. In the resulting datasets, some species that were present in all samples were excluded from the evaluation because the comparison between the situations with and without them in this pipeline was difficult. Under this deterministic setting, the correspondence between Δtopography and the dissimilarity induced by focal-species removal remained significant in 14 of the 15 parameter sets (Supplementary Fig. S9), indicating that the positive association is consistent with or without stochastic perturbations.

Second, we evaluated performance under post-transient switching dynamics, where stochasticity, extinction, and immigration can repeatedly move communities among alternative quasi-stable states. To construct such test systems, we randomly generated candidate gLV parameter sets and screened them using three criteria. First, to confirm multistability as a prerequisite for state shifts, we simulated 200 trajectories up to $t = 200$ with noise set to zero from distinct initial conditions (each species initialized from $U(0,1)$, with 30% randomly masked to zero). We assessed whether endpoint communities (mean composition over $t = 195–200$) formed discrete states by hierarchical clustering (Ward's method) on Bray–Curtis dissimilarities, choosing cluster numbers ($k = 2–10$) to maximize the mean silhouette width and retaining candidates with a maximum mean silhouette width > 0.30. Second, to ensure a sufficient number of species available for Δtopography*,* we ran an additional simulation to $t = 1000$ and retained parameter sets with more than 35 available species, where a species was considered available if it was present ($N > 0$) in > 5% of time points and absent ($N = 0$) in > 5% of time points during $t = 101–1000$ as the post-transient dynamics. Third, to select systems that switched among states after the transient, we computed Bray–Curtis dissimilarities between consecutive window-averaged community compositions (window size = 20) over $t = 101–1000$ and retained parameter sets with at least one event exceeding 0.25. We then selected three parameter sets that satisfied all criteria and simulated each system until $t = 3000$. To focus on post-transient dynamics, we constructed evaluation datasets by uniformly sampling 200 time points from $t = 101–3000$ and applied the same benchmark procedure as in the primary analysis (Supplementary Fig. S10).

In these switching systems, system-wide correlations between Δtopography and community dissimilarity were significant in only one of the three parameter sets, likely because pronounced community-level impacts were concentrated in a small subset of species (Supplementary Fig. S10c–d). Nevertheless, across all three systems, species whose removal induced the largest compositional changes consistently exhibited high Δtopography values. Together, these supplementary validations support that Δtopography can prioritize species whose removal induces relatively large community-

level compositional changes across dynamical regimes, including deterministic dynamics and post-transient switching driven by disturbance, extinction, and immigration.

Supplementary Note 3. Benchmark analysis: interaction properties and inferred basin number

Previous theoretical studies have suggested that properties of interspecific interaction networks can influence the number and stability of alternative attractors in ecological dynamical systems[1]. However, quantitatively predicting the number of attractors from the interaction matrix remains difficult for general gLV systems[2]. Moreover, basins inferred in our assembly landscape framework are operationally defined as high-probability regions in the reconstructed state-occupancy landscape, and therefore are related to, but not necessarily identical to, deterministic attractors of the underlying gLV model.

To examine how global properties of the interaction network are associated with basin structure in the reconstructed assembly landscapes, we analyzed the 15 gLV parameter sets used in Supplementary Figs. S7–S8. For each parameter set, we reconstructed the energy landscape from the corresponding binarized community matrix and estimated the number of basins using a steepest-descent procedure initiated from 150,000 randomly generated community states. We then tested whether the inferred number of basins was associated with interaction-network connectance or mean interaction strength.

We did not detect a significant difference in the number of inferred basins among the three connectance levels used in the benchmark analysis: $C = 0.2$, 0.3, and 0.4 (Kruskal-Wallis test: χ^2(degree of freedom = 2) = 2.77, P = 0.250; Supplementary Fig. S51a). In addition, mean interaction strength was not significantly correlated with the number of inferred basins (Kendall's rank correlation coefficient: $\tau = -9.66 \times 10^{-3}$, P = 0.960; Supplementary Fig. S51b). These results suggest that, under the parameter settings examined here, the number of basins inferred by the assembly landscape analysis was not explained by connectance or mean interaction strength alone. Instead, basin structure may depend on more detailed features of the interaction network, nonlinear community dynamics, stochastic perturbations, extinction/immigration processes, and the sampling scheme.

Supplementary Note 4. Optimization of data-matrix preparation for assembly landscape inference

To ensure that assembly landscape inference was robust to data-matrix preparation procedures, we compared two alternative strategies for binarization and taxon selection. In addition to the primary method based on each taxon's abundance distribution, we evaluated an alternative approach using uniform relative-read-count thresholds. Relative abundance thresholds ranging from 0 to 0.1 (increments of 0.01) were applied to binarize the datasets, after which taxa were prioritized and

selected using the same criterion as in the primary analysis. Among all combinations of abundance thresholds and retained taxon numbers, an optimal binarized matrix was selected based on how well binary community dissimilarities preserved the structure of the original abundance data.

Applying the abundance-distribution-based method to the family-level datasets resulted in the selection of 34 fungal families and 45 prokaryotic families (Supplementary Figs. S12–S13; Supplementary Tables S8–S9). Importantly, this dataset retained both Russulaceae, an ectomycorrhizal fungal family, and Glomeraceae, an arbuscular mycorrhizal fungal family, each detected in more than 100 samples (Russulaceae: 916 samples; Glomeraceae: 173 samples).

In contrast, the uniform-threshold approach selected 29 fungal families and 49 prokaryotic families (Supplementary Fig. S26; Supplementary Tables S10–S11). Under this procedure, Glomeraceae was detected in only 38 samples (Supplementary Table S10), indicating substantial loss of arbuscular mycorrhizal fungal signals. This discrepancy likely reflects the known underestimation of the presence of arbuscular mycorrhizal fungi in amplicon sequencing targeting broad fungal lineages[3].

Taken together, these results suggest that binarization based on uniform abundance thresholds may propagate methodological bias into downstream assembly landscape analyses. Therefore, all subsequent analyses were conducted using the binarization approach based on each taxon's abundance distribution.

Supplementary Note 5. Energy landscape inferences for complex microbiomes

In this study, we applied an energy landscape framework to reconstruct probabilistic assembly landscapes of complex root microbiomes from empirical data. In statistical physics, energy landscape formulations (e.g., free energy or Lyapunov potentials) are formally justified for systems with symmetric interactions or, more generally, for dynamics that admit a global Lyapunov function[4]. Microbial communities, however, are typically governed by strongly asymmetric interactions, including predator–prey relationships, antibiotic-mediated inhibition, and metabolic cross-feeding[5,6]. Such asymmetries violate detailed balance and can generate non-equilibrium dynamics such as sustained oscillations or limit cycles[7,8]. Consequently, the assumption that community dynamics follow monotonic descent along a global landscape gradient may not strictly hold for complex microbiomes.

Notably, despite these theoretical limitations, potential-like descriptions of system states have successfully characterized diverse complex systems, including protein folding, brain activity, and ecological communities[9–13]. In ecological applications, energy landscape approaches have captured compositional instability among community states and have successfully predicted observed transitions in community composition[13]. Consistent with this perspective, our simulation-based validation indicated that structural changes in inferred landscape topography correspond directly to

shifts in community destinations (Supplementary Figs. S7–S10). These results suggest that the inferred landscapes reflect meaningful statistical constraints governing community assembly outcomes. In this sense, the inferred landscape should be interpreted as a probabilistic summary of community-state distributions rather than a deterministic Lyapunov potential governing ecological dynamics.

Although broader experimental validation incorporating explicit biological mechanisms remains necessary, the present findings suggest that landscape-based frameworks can capture key statistical features of microbiome assembly even under non-equilibrium ecological dynamics. From this viewpoint, the assembly landscape inferred here can be interpreted as a statistical potential that summarizes the probabilistic distribution of microbiome states emerging from asymmetric ecological interactions.

Supplementary Note 6. Robustness of basin detection to the number of initial states

To evaluate whether the number of initial states used in steepest-descent simulations influences the detection of basin bottoms in energy landscape analysis, we examined how basin estimates changed across a wide range of initial-state counts. For each host-plant background in the fungal and prokaryotic community datasets, steepest-descent procedures were conducted using ten different numbers of initial states, ranging from 100 to 200,000 (100, 1,000, 5,000, 10,000, 20,000, 40,000, 70,000, 100,000, 150,000, and 200,000; Supplementary Fig. S25).

In fungal assembly landscapes, the number of detected basins remained consistent across all evaluated initial-state settings for each host-plant background (Supplementary Fig. S25a), indicating that basin detection was robust to the choice of initial-state number. In prokaryotic assembly landscapes, basin numbers converged when 5,000 or more initial states were used (Supplementary Fig. S25b), suggesting that sufficiently large initial-state sampling stabilizes basin detection.

These results confirm that the number of initial states adopted in the main analyses was sufficient to reliably identify basin bottoms in both fungal and prokaryotic assembly landscapes.

Supplementary Note 7. Cross-validation confirms predictive robustness of inferred assembly landscapes

To evaluate whether the inferred assembly landscapes possess predictive capability beyond the fitted data, we performed 100-fold cross-validation of basin assignments. Basin identities inferred from the model fitted to the full dataset were used as reference classifications (Supplementary Fig. S14). For each cross-validation fold, models were reconstructed using datasets excluding a subset of focal

community samples, and basin assignments for the excluded samples were predicted using the refitted models.

Predictive performance was evaluated in two ways: (i) agreement in basin assignments and (ii) similarity of basin-bottom community compositions. Assignment accuracy was quantified as the proportion of samples assigned to the same basins as in the full-dataset analysis, whereas deviations in basin identities were assessed using Jaccard dissimilarity between basin-bottom community memberships (Supplementary Fig. S27).

Across the fungal and prokaryotic datasets, 92.7% and 86.1% of community samples, respectively, were assigned to identical basins as those inferred from the full dataset (Supplementary Fig. S27a). Differences in basin-bottom compositions were also minimal. Across host-plant backgrounds, only a small fraction of samples showed noticeable deviations (Jaccard dissimilarity > 0.1: 7.17% in fungi and 4.74% in prokaryotes; Supplementary Fig. S27b).

Together, these results indicate that the energy landscape framework provides stable and predictive estimates of assembly landscapes for the present root microbiome datasets.

Supplementary Note 8. Bootstrap analysis on the consistency of inferred basin structures

To evaluate how sampling variability influences assembly landscape inference, we performed bootstrap resampling analyses (1,000 replicates) for both fungal and prokaryotic datasets. For each bootstrap replicate (i.e., generated by resampling the binarized community-states matrix), the energy landscape inference procedure was repeated. We evaluated robustness of the reconstructed landscape architecture from two complementary perspectives: the compositional consistency of basin bottoms (Supplementary Fig. S28) and the stability of energy differences between community states corresponding to basin bottoms in the original landscape and boundary states identified in bootstrap landscapes (Supplementary Fig. S29).

We first assessed uncertainty in the family-level composition of basin bottoms identified in the reconstructed landscapes (Figs. 2c and 3c). Steepest-descent simulations were performed to identify basin bottoms using an identical set of 150,000 initial states for the assembly landscape inferred from the original dataset and for each bootstrap replicate (Supplementary Fig. S28). Using the same initial states allowed direct comparison of the basin bottom reached from a given initial community state across the original and bootstrap landscapes. For each basin bottom identified in the original landscape, we collected the set of initial states that converged to that basin and then tracked the basin bottoms reached from those same initial states in each bootstrap replicate. For each family, we calculated the proportion of bootstrap outcomes in which the family was present in the corresponding bootstrap-inferred basin bottom. These proportions were summarized as family-wise basin representation scores for each original basin bottom.

In addition to compositional consistency, we evaluated robustness and uncertainty in landscape topography by examining energy differences between community states corresponding to basin bottoms in the original landscape and their corresponding lowest-energy boundary states (Supplementary Fig. S29). Because absolute energy values are comparable only among community states within a given energy landscape, for each bootstrap replicate we recalculated the energy of the community states corresponding to each basin bottom and its corresponding lowest-energy boundary state (defined in the original landscape) under the bootstrap-inferred landscape and calculated the energy difference between them as follows:

$$\text{energy difference} = E_{\text{boundary}} - E_{\text{basin}}, \quad \text{(S1)}$$

where E_{basin} and E_{boundary} denote the energy of the community states corresponding to the basin bottom and its corresponding boundary state, respectively, evaluated under the bootstrap-inferred landscape. A positive value of this energy difference indicates that the relative ordering between basin-bottom states (defined in the original landscape) and their corresponding boundary states is preserved (Supplementary Fig. S29a). This energy-difference index was calculated for each basin in the original energy landscape across bootstrap replicates, yielding a distribution summarized as a histogram. This framework was further used to examine the consistency of inferred compositional transitions between major basin groups (Supplementary Fig. S29b). Specifically, transitions between arbuscular mycorrhizal and ectomycorrhizal basins (Fig. 2c) were examined for fungal communities, whereas transitions between Group I, II, and III basins (Fig. 3c) were examined for prokaryotic communities.

In the fungal assembly landscapes, the ectomycorrhizal family Russulaceae was consistently detected in ectomycorrhizal basin bottoms across bootstrap replicates (Supplementary Figs. S30–S31; F_B1–B3), indicating high robustness of these basins. The arbuscular mycorrhizal family Glomeraceae was also frequently detected in arbuscular mycorrhizal basins inferred for dual-mycorrhizal host plants, although its mean basin representation score across bootstrap replicates was relatively low (Supplementary Fig. S31; F_B4–B5 and F_B7). Consistent with these compositional patterns, the relative ordering between basin-bottom states defined in the original landscape and their boundary states was largely preserved for ectomycorrhizal basins, with boundary states typically having higher energy than the corresponding community states identified as basin bottoms in the original dataset (Supplementary Fig. S32a). Further analysis focusing on transitions between mycorrhizal types indicated that boundary states separating ectomycorrhizal basin-bottom states in the original landscape from arbuscular mycorrhizal states were detected in all 1,000 bootstrap replicates (Supplementary Fig. S32b). For arbuscular mycorrhizal basins, the relationships between basin-bottom states and inter-basin-group boundaries observed in the original analysis were preserved in 69.1%, 73.2%, and 75.4% of bootstrap replicates for *Populus* (F_B4), *Acer* (F_B4), and *Juglans* (mean of F_B5 and F_B7), respectively (Supplementary Fig. S32b).

In the prokaryotic assembly landscapes, dominant basin-bottom families identified in Figure 3c (e.g., Gemmataceae, Pedosphaeraceae, and Solibacteraceae) were consistently recovered across host-plant backgrounds (Supplementary Figs. S33–S34). Families characterizing Group II and Group III basins, including members of Pseudomonadota (e.g., Sterellaceae) and Actinomycetota (e.g., Microbacteriaceae), also showed stable detection patterns (Supplementary Figs. S33–S34). Regarding the relative energy levels between basin-bottom states defined in the original landscape and boundary states inferred from bootstrap landscapes, some basins exhibited high consistency (e.g., P_B3 in *Populus* and P_B12 in *Juglans*; Supplementary Fig. S35a). For other basin-bottom states, the energy difference relative to the minimum-energy boundary state occasionally reversed compared with the original landscape (Supplementary Fig. S35a). Nevertheless, the relative ordering of basin bottoms and boundary states remained largely preserved across bootstrap replicates for transitions among basin groups (i.e., Group I, Group II, and Group III basins) (Supplementary Fig. S35b).

Overall, taxa with relatively sparse occurrence patterns (e.g., arbuscular mycorrhizal fungi) showed greater sensitivity to sampling variability, whereas topographic relationships between compositionally distinct basin types were comparatively stable across bootstrap replicates.

Supplementary Note 9. Basin structures inferred from genus-level assembly landscapes

To examine whether assembly landscape patterns were consistent across taxonomic resolutions, we conducted energy landscape analyses using genus-level community composition data for both fungal and prokaryotic communities. The same data-processing procedures applied in the family-level analyses were used. Samples in which more than 50% of sequence reads could not be annotated at the genus level were excluded, resulting in the removal of 98 fungal community samples and 187 prokaryotic community samples. The remaining samples were binarized, and genera were selected following the criteria described above (Supplementary Fig. S11). The resulting community matrices comprised 20 fungal genera and 49 prokaryotic genera, respectively (Supplementary Figs. S36–S38; Supplementary Tables S12–S13).

In the fungal genus-level assembly landscape, arbuscular mycorrhizal fungi were not retained in the selected community states (Supplementary Table S12). Nevertheless, ectomycorrhizal basins containing *Russula* (family Russulaceae) were clearly detected (FG_B3–B4). In addition, potential arbuscular-mycorrhizal-related basins composed of *Ramariopsis* (family Clavariaceae) were identified (FG_B1–B2), corresponding to basins that co-occurred with Glomeraceae in the family-level analysis (Supplementary Fig. S38a).

In the prokaryotic genus-level assembly landscape (Supplementary Table S13), basin structures corresponding to Group II were less clearly resolved, largely because genus-level annotation was limited for several Pseudomonadota families (e.g., Sutterellaceae and A21b) (Supplementary Fig. S38b). In contrast, Group I basins (PG_B10–B12), composed of commonly detected taxa such as

Candidatus Solibacter (family Solibacteraceae) and *Conexibacter* (family Solirubrobacteraceae), were consistently identified. Group III basins (PG_B1–B5), additionally characterized by Actinomycetota genera such as *Nocardioides* (family Nocardioidaceae) and *Actinoplanes* (family Micromonosporaceae), were also clearly recovered (Supplementary Fig. S38b).

Together, these results indicate that assembly landscape architectures inferred at the family level capture community assembly rules that remain detectable at finer taxonomic resolution, despite reduced annotation completeness at the genus level.

Supplementary Note 10. Sensitivity analysis based on genus-level assembly landscapes

To examine whether the identification of keystone taxa depended on taxonomic resolution, we evaluated the effects of fungal and prokaryotic genera on assembly landscape topography using the same analytical procedures applied in the family-level analysis.

In fungal assembly landscapes, several endophytic fungal genera exhibited strong and significant Δtopography and/or Δevenness values. For example, *Oidiodendron* in *Larix* and *Cladophialophora* in *Populus*, *Acer*, and *Juglans* were associated with pronounced changes in landscape architecture (Supplementary Fig. S40a), consistent with the family-level results. In addition, dominant ectomycorrhizal genera such as *Russula*, *Lactarius*, and *Tomentella*, together with several bacterial genera, were newly identified as influential taxa at the genus level (Supplementary Fig. S40a).

Similarly, in prokaryotic assembly landscapes, *Candidatus* Udaeobacter and *Candidatus* Solibacter consistently showed significant impacts across multiple host-plant backgrounds, in agreement with the family-level analysis (Supplementary Fig. S40b). Furthermore, the Actinomycetota genus *Mycobacterium* emerged as a potential keystone taxon in four of the six host-plant backgrounds examined (Supplementary Fig. S40b).

Together, these results indicate that analyses at finer taxonomic resolution highlight additional candidate keystone taxa while consistently recovering the central roles of endophytic fungi and specific bacterial lineages in shaping root fungal and prokaryotic community assembly.

Supplementary Note 11. Sensitivity analysis under alternative abundance assumptions

To evaluate how assumptions about focal taxon's abundance influence inference of community-scale effects, we conducted sensitivity analyses using alternative abundance conditions defined by the 25% and 75% abundance quantiles. Δtopography and Δevenness values were calculated using the same procedure applied to the comparison between the 0% and 50% abundance quantile conditions (Fig. 1g).

Across alternative abundance settings, several endophytic fungi and specific bacterial taxa were consistently associated with strong changes in fungal assembly landscapes (Supplementary Fig. S42). For example, the endophytic genera *Podila*, *Hyaloscypha*, and *Cladophialophora* repeatedly exhibited among the highest Δtopography values in one or two host-plant backgrounds across the three abundance conditions [comparison between 0% (absence) and 50% (median) abundance quantiles, Fig. 4b; comparison between 0% and 25 % quantiles, Supplementary Fig. S42a; comparison between 0% and 75% quantiles, Supplementary Fig. S42b].

In prokaryotic assembly landscapes, some genera highlighted in the comparison between 0% and 50% abundance quantiles (Fig. 4d) were not detected in the 0% versus 25% abundance quantile comparison. Nevertheless, many taxa were consistently identified as candidate keystones, including *Candidatus* Udaeobacter and *Candidatus* Solibacter (Supplementary Fig. S43).

Taken together, these results indicate that the results of the keystone-exploration analysis are broadly robust across a range of abundance assumptions.

Supplementary Note 12. Sensitivity analysis with alternative randomization-block settings

To evaluate whether the identification of candidate keystone taxa was robust to alternative randomization schemes accounting for spatial structure, we conducted additional permutation tests incorporating both host-plant identity and spatial configuration of sampling locations. Defining randomization blocks as unique combinations of host plants and the 125 sampling points resulted in excessively small sample sizes within blocks, preventing reliable permutation procedures. Instead, spatial sampling blocks were constructed based on the relative spatial arrangement of sampling points using *k*-means clustering. The number of clusters was varied from 2 to 15, and the optimal clustering was determined using the silhouette method implemented in the R cluster package[14]. Among the tested configurations, sampling points were grouped into seven spatial blocks (Supplementary Fig. S44).

Permutation tests conducted using these spatial blocks consistently identified several endophytic fungi and bacterial taxa as candidate keystone genera in fungal assembly landscapes, including *Oidiodendron* in *Pinus*, *Larix*, and *Acer*, *Cladophialophora* in *Populus*, *Acer*, and *Juglans*, and *Thermosporothrix* in *Juglans* (Supplementary Fig. S45a). In prokaryotic assembly landscapes, the bacterial genera *Candidatus* Udaeobacter and *Candidatus* Solibacter also showed significant effects on landscape topography (Supplementary Fig. S45b).

Supplementary Note 13. Uncertainty in the calculation of Δtopography and Δevenness

To assess uncertainty in the keystoneness metrics introduced in this study, we quantified the variability of *z*-standardized Δtopography and Δevenness values using bootstrap resampling (100 replicates). Because each bootstrap replicate required 10,000 permutations constrained within the six host-plant backgrounds, exhaustive evaluation across all host–genus combinations was computationally prohibitive. We therefore focused on representative focal taxa highlighted in the main text.

Among the examined genera, the inferred effects of *Oidiodendron* and *Cladophialophora* on fungal assembly landscapes showed moderate variation among bootstrap replicates. Mean Δtopography values were 7.36 ± 4.24 (mean ± standard deviation) for *Oidiodendron* and 8.56 ± 6.70 for *Cladophialophora* (Supplementary Fig. S46a). In addition, *Cladophialophora* exhibited consistent effects on higher-order landscape structure, with Δevenness values of 4.54 ± 2.51 (Supplementary Fig. S46b).

In prokaryotic assembly landscapes, *Candidatus* Udaeobacter and *Candidatus* Solibacter consistently showed high Δtopography values together with negative Δevenness across bootstrap replicates (Supplementary Fig. S46c–d).

Overall, although uncertainty was present in the absolute magnitudes of the indices, the bootstrap analyses demonstrated that the proposed metrics robustly captured consistent directional effects of focal genera on assembly landscape reorganization.

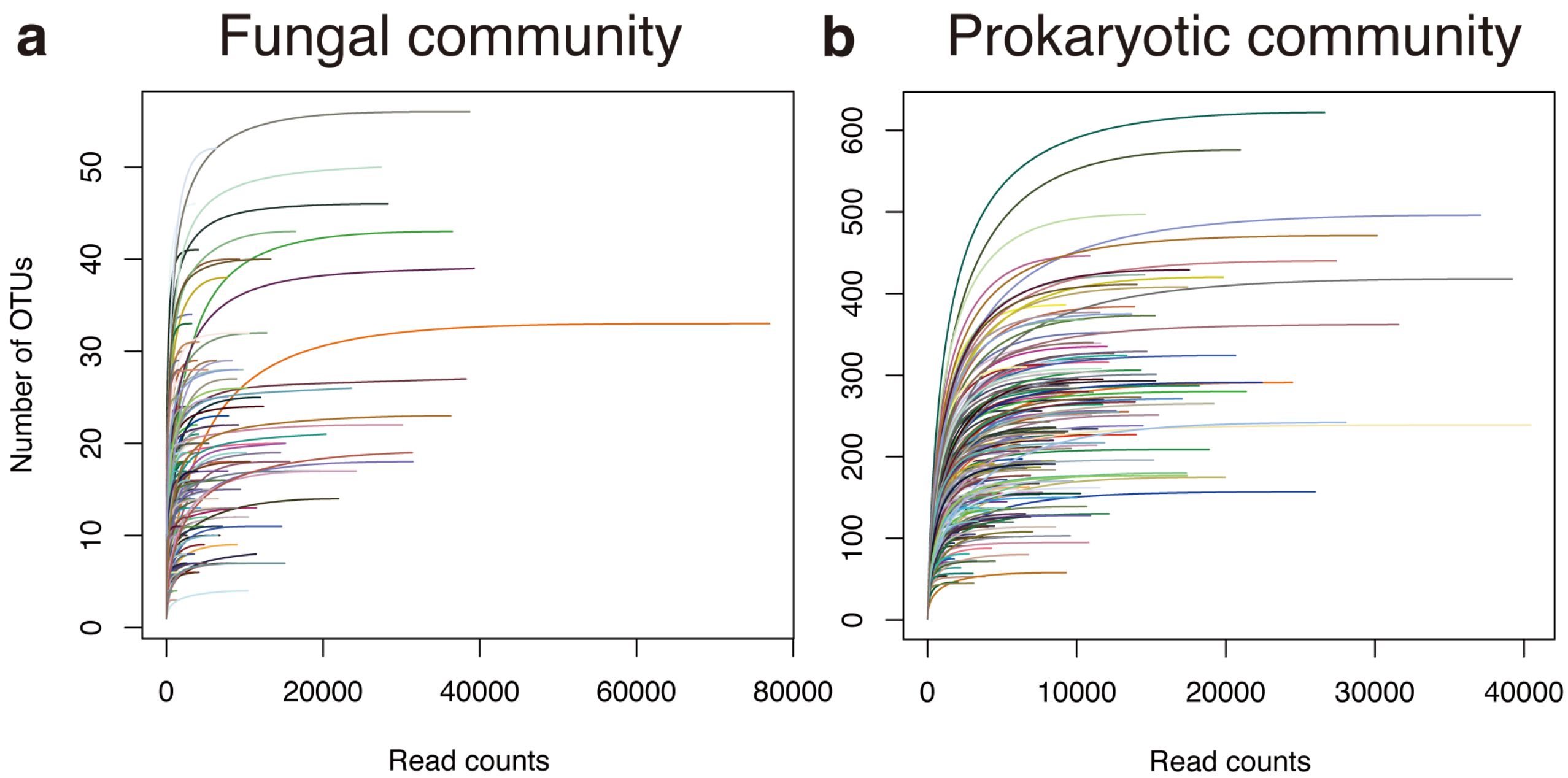

Supplementary Fig. S1 | Rarefaction curves. (**a**) Number of fungal OTUs detected in root samples. For visualization, the relationships between the number of sequencing reads and the number of fungal OTUs detected in 200 randomly selected samples are shown. (b) Number of prokaryotic OTUs. Source data are provided as a Source Data file.

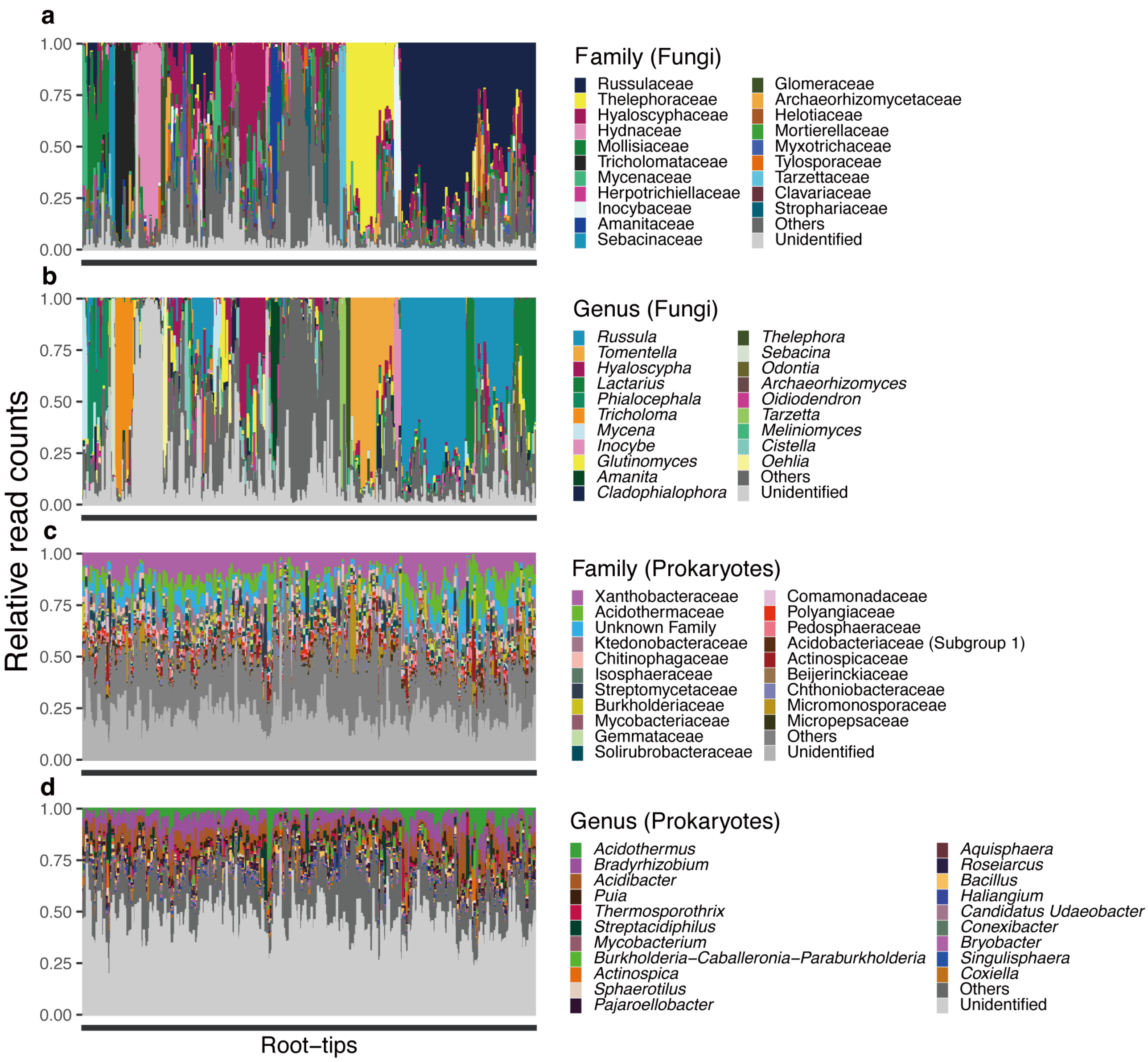

Supplementary Fig. S2 | Fungal and prokaryotic community compositions. (**a**) Family-level taxonomic compositions of fungal communities across 1,270 root-tip samples. (**b**) Genus-level taxonomic compositions of fungal communities. (**c**) Family-level taxonomic compositions of prokaryotic communities. (**d**) Genus-level taxonomic compositions of prokaryotic communities. Source data are provided as a Source Data file.

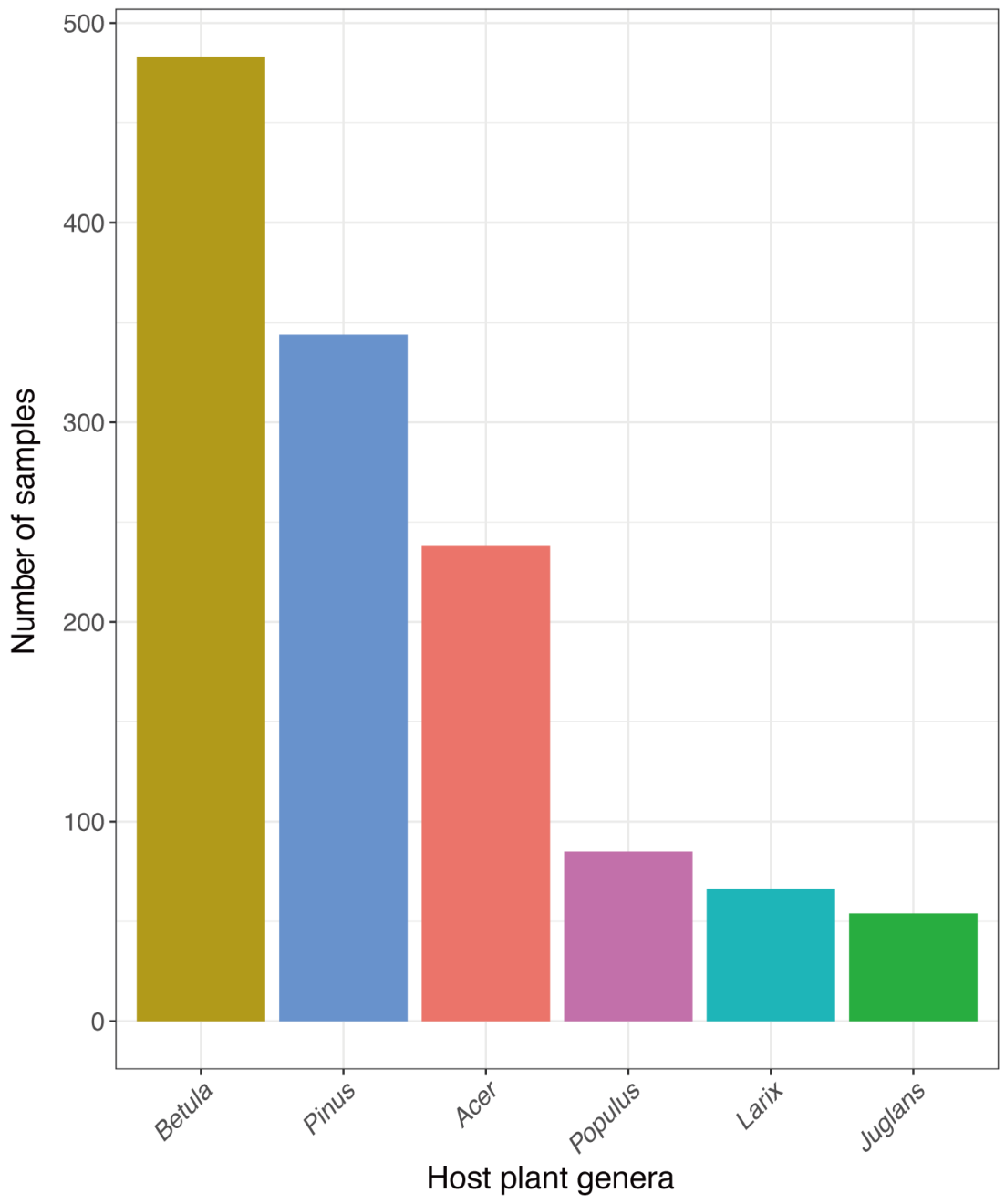

Supplementary Fig. S3 | Number of the samples from each host plant. The sample size of each host plant genera used in energy landscape analysis is shown. After a series of quality filtering, 1,270 root samples identified as those of major six plant genera (i.e., plant genera with more than 50 samples) were used in the statistical analysis. Source data are provided as a Source Data file.

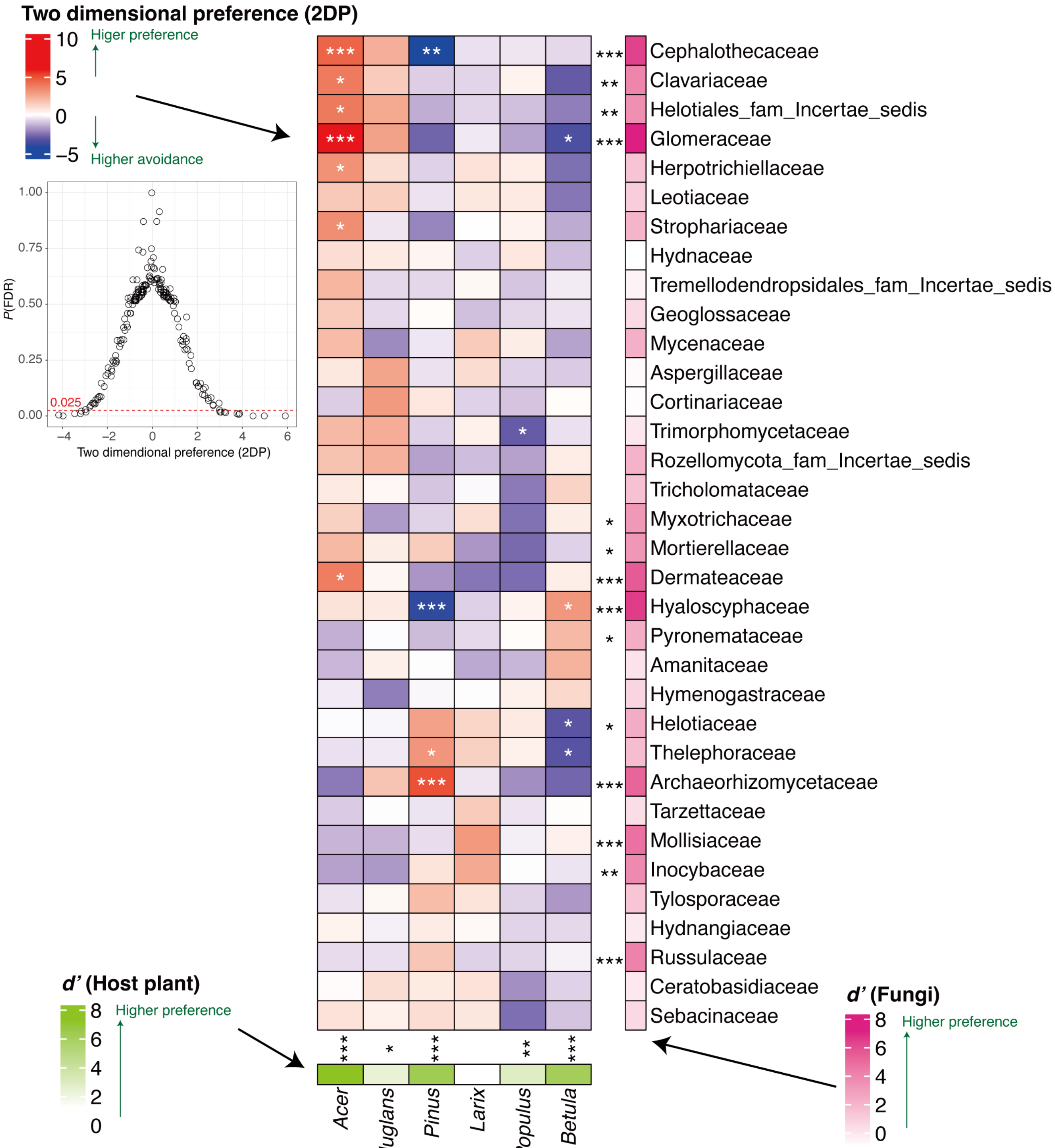

Supplementary Fig. S4

Supplementary Fig. S4 | Preferences in plant–fungus associations. Host preferences of the fungal families analyzed in the energy landscape analysis (rows) and symbiont preferences of the six host plant genera (column) are shown as *z*-standardized *d'* estimates. In addition, specificity of each host–symbiont pair is represented with a two-dimensional preference (2DP) estimate in the matrix heatmap, which indicates the extent to which the association of a target plant–fungal pair is observed more or less frequently than expected by chance. Significance of the *z*-standardized *d'* (one-tailed test) and 2DP estimates (two-tailed test) are evaluated with the null model simulations (See Methods; ***, *P (FDR)* < 0.001; **, *P (FDR)* < 0.01; *, *P (FDR)* < 0.05). The relationship between the 2DP estimates

and FDR-corrected *P* values are also shown in the left-side panel. Source data are provided as a Source Data file.

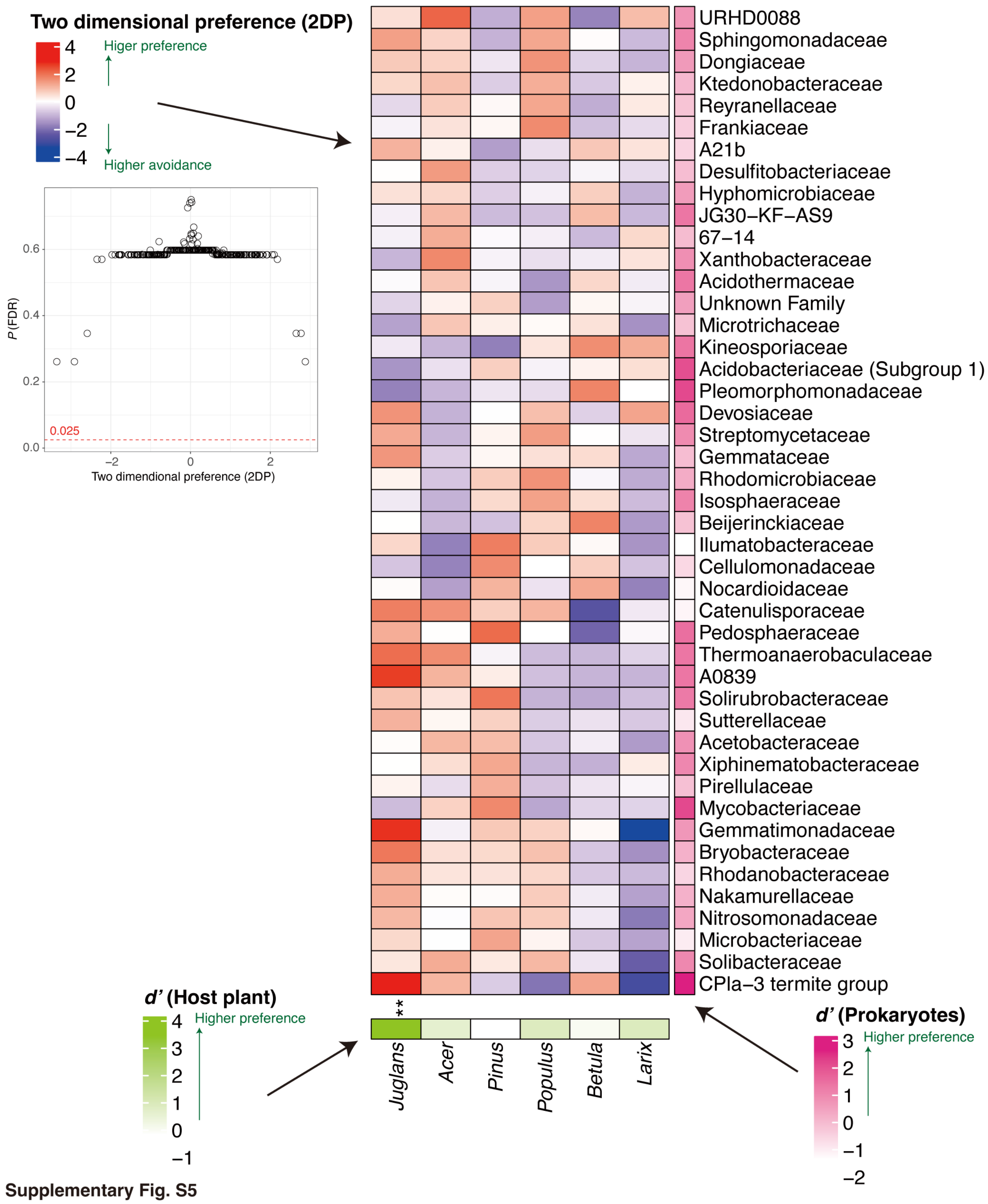

Supplementary Fig. S5 | Preferences in plant–prokaryote associations. Host preferences of the prokaryotic families analyzed in the energy landscape analysis (rows) and symbiont preferences of the six host plant genera (column) are shown with the *z*-standardized *d'* estimates. In addition, specificity of each host–symbiont pair is represented with a two-dimensional preference (2DP) estimate in the matrix heatmap, which indicates the extent to which the association of a target plant–prokaryote pair is observed more or less frequently than expected by chance. Significance of the z-standardized *d'* (one-tailed test) and 2DP estimates (two-tailed test) are evaluated with the null model simulations (See

Methods; ***, P (FDR) < 0.001; **, P (FDR) < 0.01; *, P (FDR) < 0.05). The relationship between the 2DP estimates and FDR-corrected P values are also shown in the left-side panel. Source data are provided as a Source Data file.

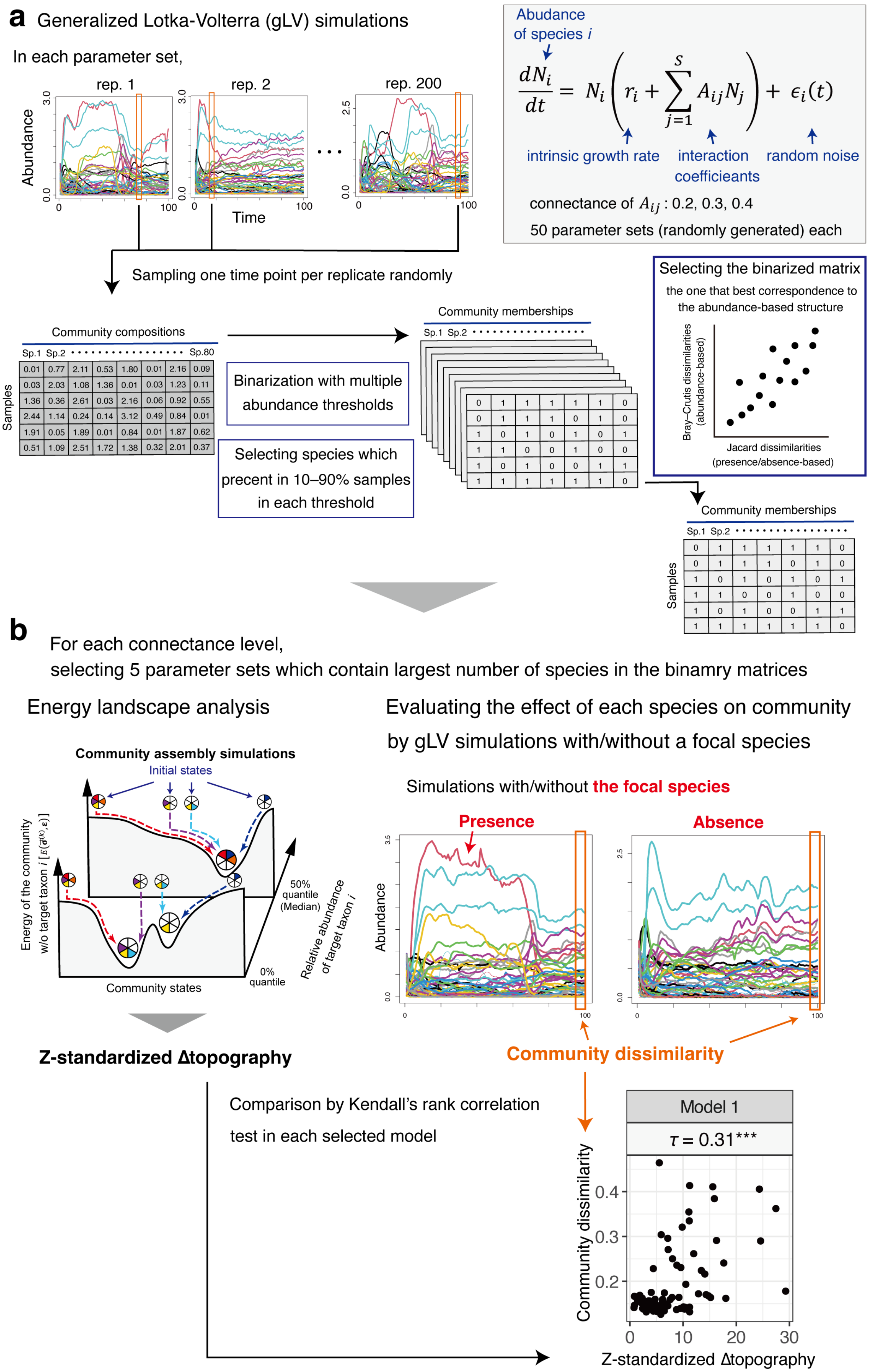

Supplementary Fig. S6

Supplementary Fig. S6 | Workflow for preparing simulated community datasets and validating candidate keystone inference using generalized Lotka–Volterra models. (**a**) Generation of simulated community datasets. Benchmark community datasets were generated using generalized Lotka–Volterra (gLV) models. Community dynamics were simulated under varying species richness and interaction structures defined by connectance (proportion of non-zero entries in A_{ij}). For each model, stochastic dynamics were simulated from independently generated initial species abundances until t = 100. One time point was randomly sampled from each simulation to represent an independent community composition, and sampled states were combined into datasets of multiple replicate communities. Simulated abundance data were converted into binarized community-state matrices using an optimal threshold selected from candidate values corresponding to evenly spaced abundance quantiles from 10% to 90% to maximize correspondence between Bray–Curtis (abundance-based) and Jaccard (binary data) dissimilarities. (**b**) Validation of candidate keystone inference. To evaluate whether the keystone-exploration framework prioritizes species with strong community-level influences, the workflow in (**a**) was applied to 80-species gLV models at three connectance levels (0.2, 0.3, and 0.4). For each connectance level, 50 independent parameter sets were generated, and a dataset of 200 community compositions was obtained for each parameter set from 200 simulations with independently generated initial abundances. After binarization, species occurring in fewer than 10% or more than 90% of samples were excluded as uninformative for energy landscape inference. Because the number of species remaining after filtering varied among parameter sets, the five sets retaining the most species at each connectance level were selected (15 model systems total). Energy landscape analysis was applied to each selected dataset, and z-standardized Δtopography was calculated for each species by comparing the 0% and 50% (median) abundance quantiles. Species influence was assessed in additional paired simulations in which a focal species was present (initial abundance = 0.5) or removed (abundance fixed at zero), with identical initial abundances for all other species (200 pairs per focal species). Bray–Curtis and Jaccard dissimilarities between the paired terminal communities quantified compositional impacts. Associations between Δtopography and these impacts were evaluated using Kendall's rank correlation tests.

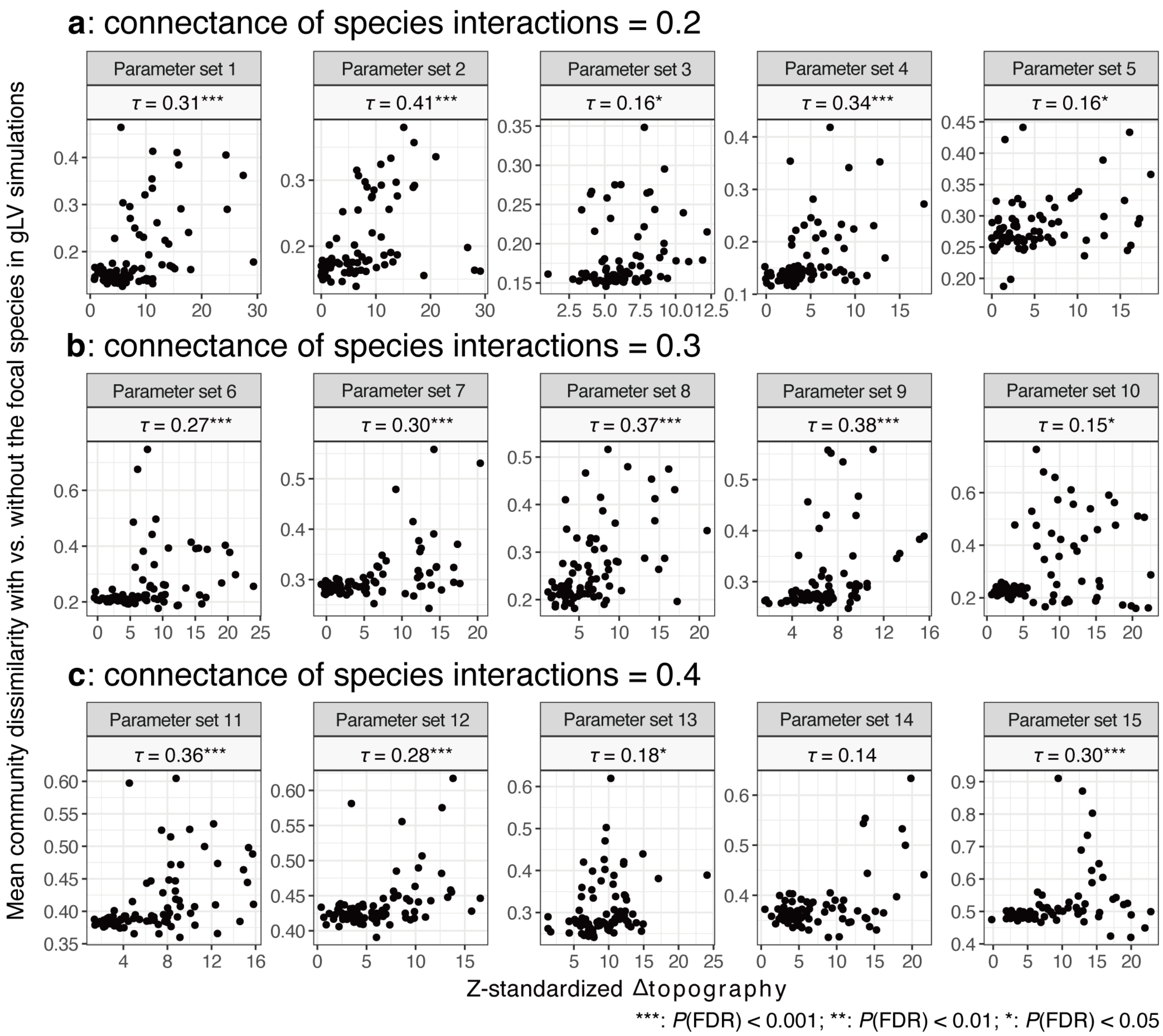

Supplementary Fig. S7 | Relationships between inferred Δtopography and species influences on community composition in generalized Lotka–Volterra simulations (evaluation using Bray–Curtis dissimilarity). Community dynamics were simulated using 15 parameter sets of generalized Lotka–Volterra models representing different interaction structures defined by connectance (sparse: parameter sets 1–5, **a**; intermediate: parameter sets 6–10, **b**; dense: parameter sets 11–15, **c**). These simulations were used to evaluate whether the proposed keystone-detection framework can recover species-level influences on community assembly (Fig. 1; see Supplementary Fig. S6 and Methods for details). To quantify the impact of a focal species on community dynamics, additional simulations were performed with and without the focal species while keeping all model parameters identical. Resulting community compositions were compared using Bray–Curtis dissimilarity. Independently, z-standardized Δtopography values were calculated from simulated community datasets using the energy landscape framework (Fig. 1). Relationships between species impacts on community composition and inferred keystoneness were evaluated using Kendall's rank correlation tests. *P* values were adjusted for multiple comparisons using the Benjamini–Hochberg false discovery rate (FDR) procedure. Source data are provided as a Source Data file.

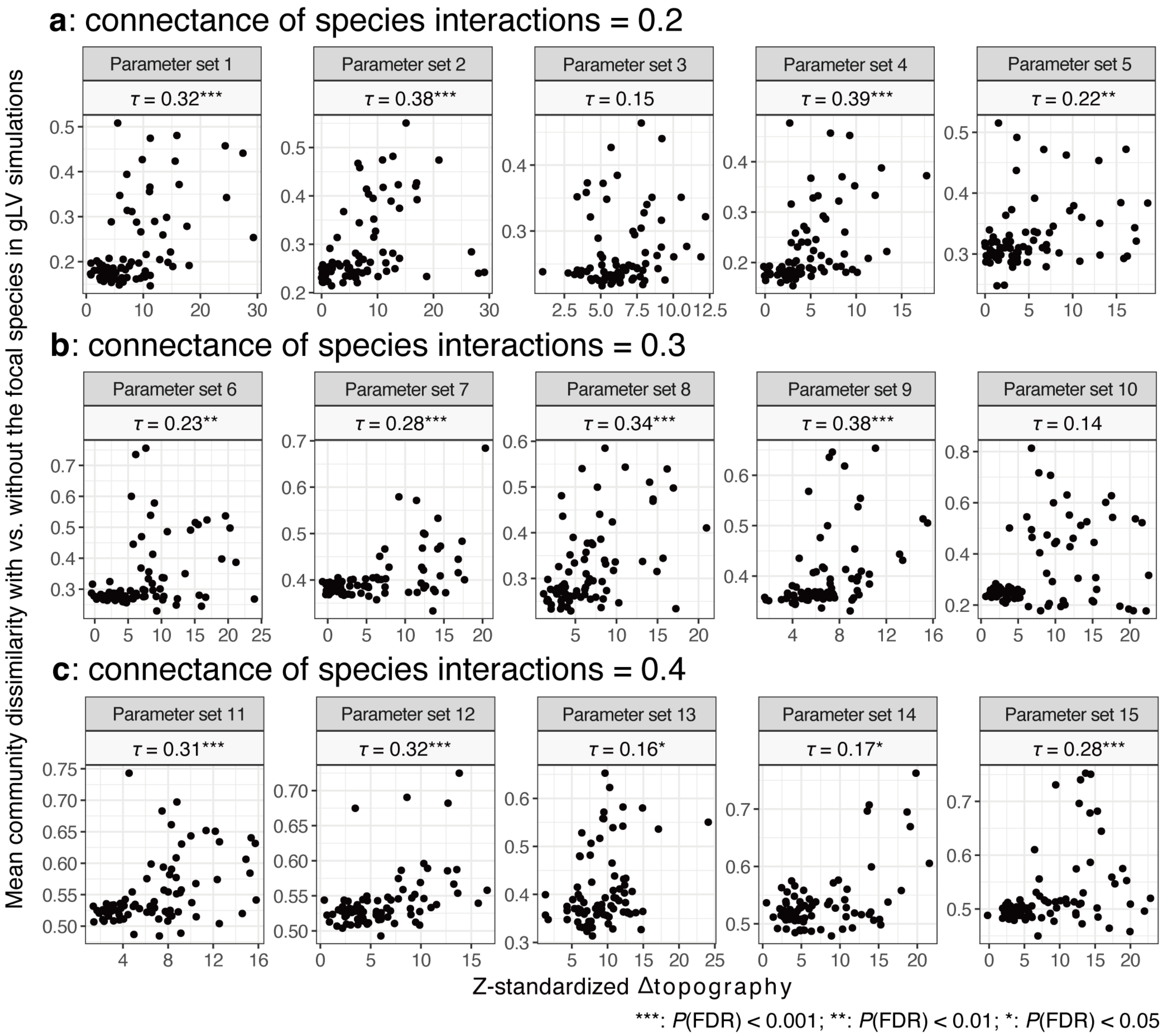

Supplementary Fig. S8 | Relationships between inferred Δtopography and species influences on community states in generalized Lotka–Volterra simulations (evaluation using Jaccard dissimilarity). Using the same simulation datasets as in Supplementary Figure S7, we evaluated whether inferred keystoneness (*z*-standardized Δtopography) reflects species-level influences on binarized community states across 15 parameter sets representing different interaction structures defined by connectance (sparse: parameter sets 1–5, **a**; intermediate: parameter sets 6–10, **b**; dense: parameter sets 11–15, **c**). To quantify the impact of a focal species on community states within each model, simulations were performed with and without the focal species while keeping model parameters identical. Resulting community compositions were binarized using the abundance thresholds defined for energy landscape analysis (see Methods), and species-level influences were quantified as the Jaccard dissimilarity between paired simulations with and without the focal species. Relationships between *z*-standardized Δtopography and species influences on community states were evaluated using Kendall's rank correlation tests. *P* values were adjusted for multiple comparisons using the Benjamini–Hochberg false discovery rate (FDR) procedure. Source data are provided as a Source Data file.

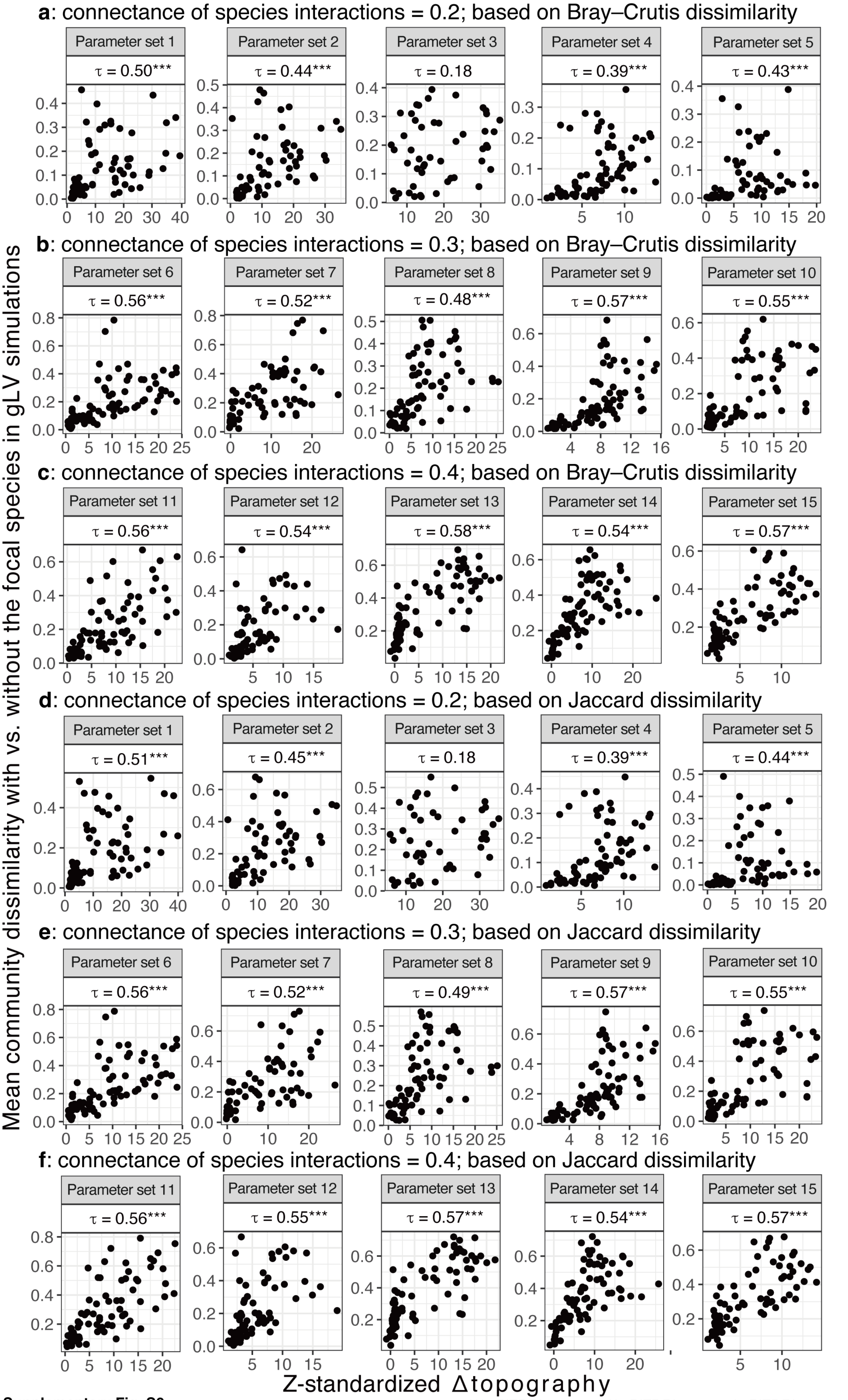

Supplementary Fig. S9

***: *P*(FDR) < 0.001; **: *P*(FDR) < 0.01; *: *P*(FDR) < 0.05

Supplementary Fig. S9 | Relationships between inferred Δtopography and species influences on community composition in deterministic generalized Lotka–Volterra simulations. To examine the robustness of the benchmark results shown in Supplementary Figures S7 and S8 to the inclusion of stochastic perturbations, we repeated the same validation analysis using deterministic generalized Lotka–Volterra (gLV) simulations in which the stochastic noise term was removed from the models described in Supplementary Figure S6. Community dynamics were simulated using 15 gLV parameter sets representing different interaction structures defined by connectance (sparse: parameter sets 1–5, **a,d**; intermediate: parameter sets 6–10, **b,e**; dense: parameter sets 11–15, **c,f**). To quantify the impact of each focal species on community dynamics, additional simulations were performed with and without the focal species while keeping all model parameters and initial abundances of the other species identical. Species-level influences were quantified using Bray–Curtis dissimilarity for abundance-based community compositions (**a–c**) and Jaccard dissimilarity for binarized community states (**d–f**). Independently, z-standardized Δtopography values were calculated from simulated community datasets using the energy landscape framework. Relationships between inferred keystoneness and simulated species influences on community composition or community states were evaluated using Kendall's rank correlation tests. *P* values were adjusted for multiple comparisons using the Benjamini–Hochberg false discovery rate (FDR) procedure. Source data are provided as a Source Data file.

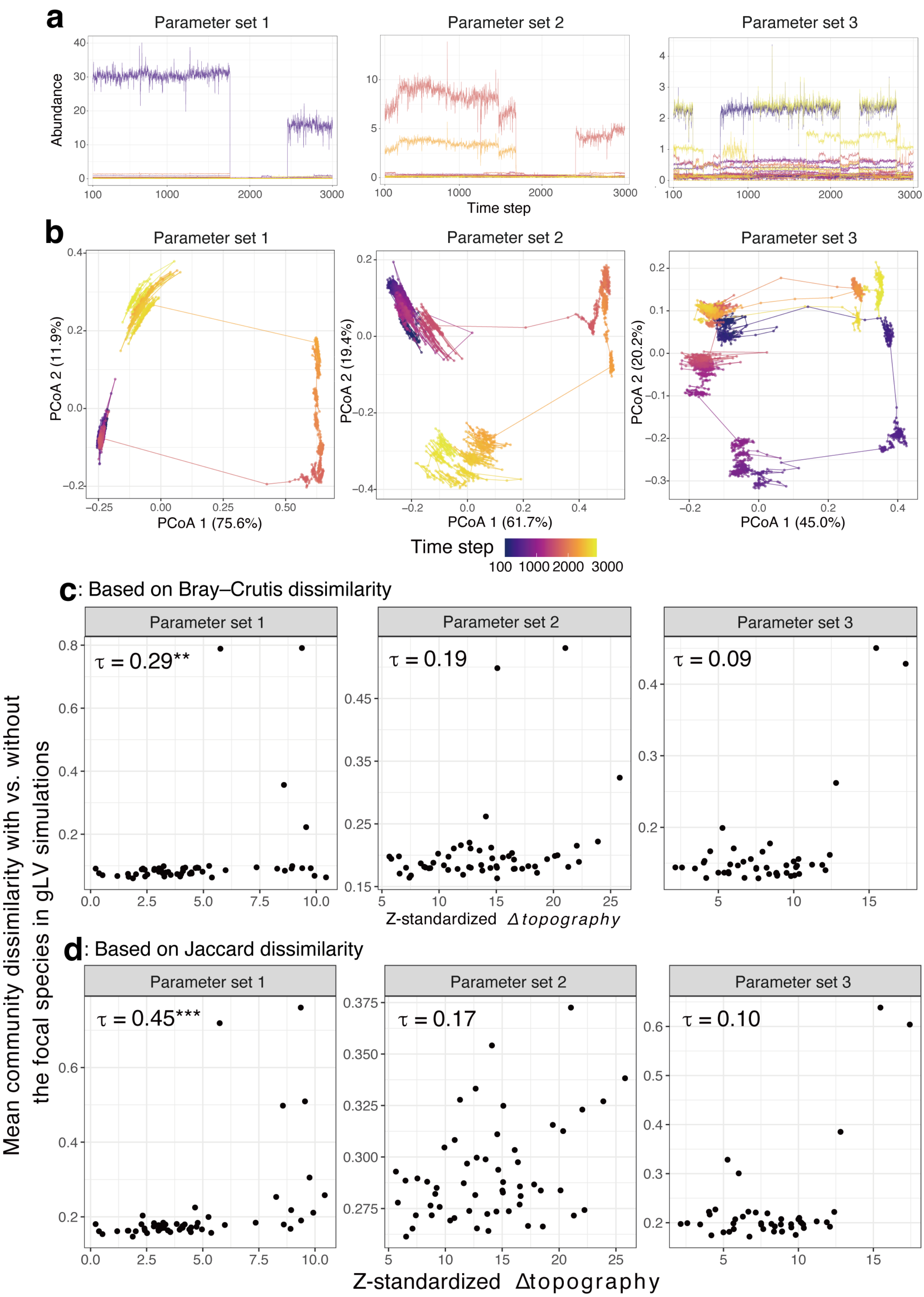

Supplementary Fig. S10

***: *P*(FDR) < 0.001; **: *P*(FDR) < 0.01; *: *P*(FDR) < 0.05

Supplementary Fig. S10 | Relationships between inferred keystoneness and species influences in perturbation-driven switching dynamics after initial transients. To examine the robustness of the benchmark results shown in Supplementary Figures S7 and S8 to the sampling scheme, we repeated the validation analysis using a stochastic gLV system in which random perturbations induced switching among discrete community compositions after the initial transient period. We first searched for a parameter set that generated initial-condition-dependent discrete community compositions, large compositional shifts within a single time series, and a sufficient number of informative species for keystoneness analysis (see Supplementary Note 2 for details). Using the resulting parameter set, we simulated community dynamics from random initial abundances up to t = 3,000, excluded the initial transient period (t = 1–100), and randomly sampled 200 time points from the remaining trajectory (t = 101–3,000). (**a**) Community dynamics after the initial transient period in the selected stochastic gLV system; different line colors in each panel indicate different species. (**b**) Principal coordinate analysis based on Bray–Curtis dissimilarity showing community trajectories after the initial transient period. Using the sampled dataset, z-standardized Δtopography values were calculated using the energy landscape framework. Independently, species-level influences were quantified by comparing simulations with and without each focal species while keeping all model parameters and initial abundances of the other species identical. Bray–Curtis dissimilarity was used for abundance-based community compositions (**c**), whereas Jaccard dissimilarity was used for binarized community states (**d**). Relationships between inferred keystoneness and simulated species-level influences were evaluated using Kendall's rank correlation tests. P values were adjusted using the Benjamini–Hochberg false discovery rate (FDR) procedure. Source data are provided as a Source Data file.

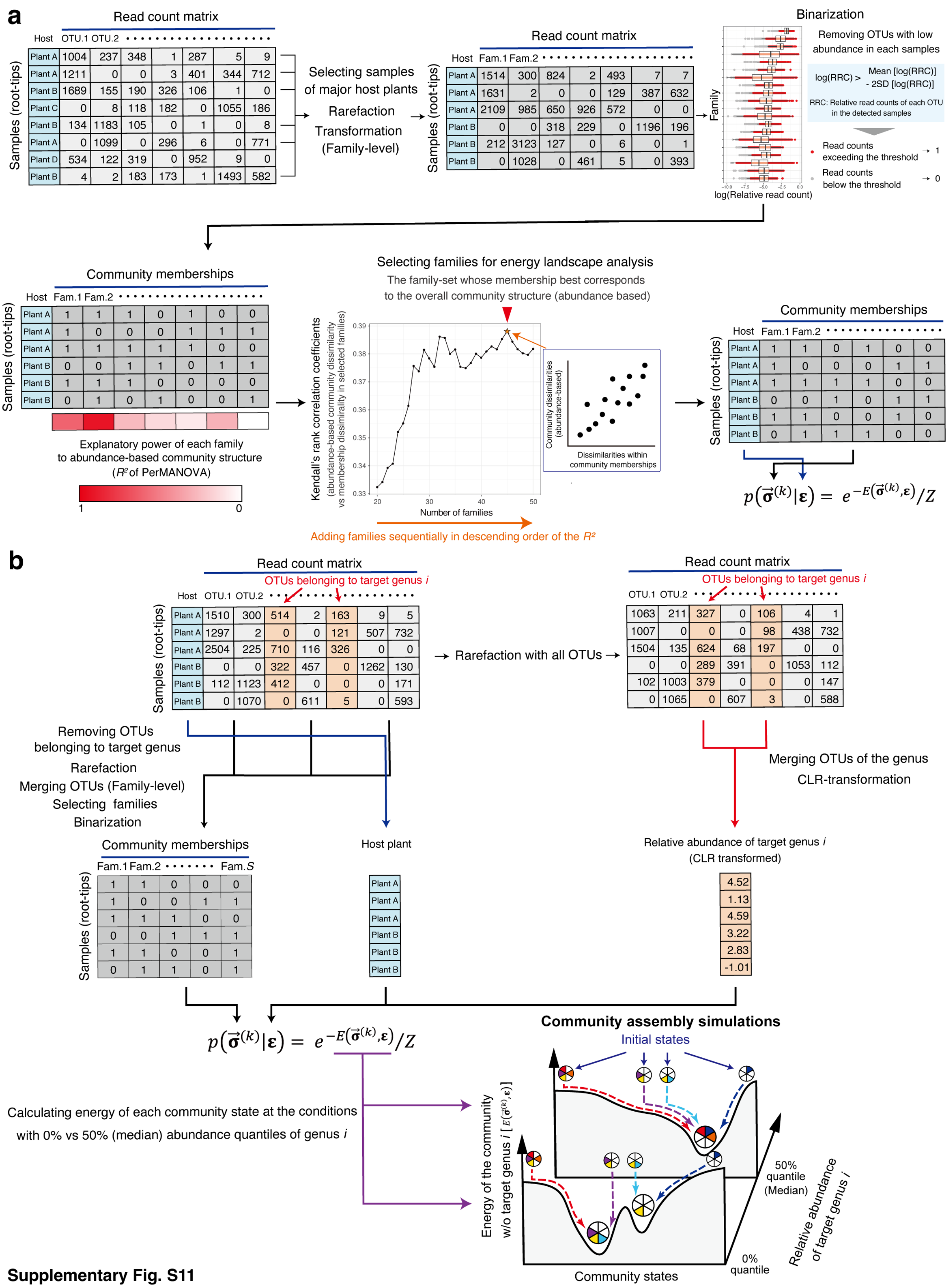

Supplementary Fig. S11

Supplementary Fig. S11 | Workflow for energy landscape analysis and evaluation of keystoneness (a) Input data for energy landscape analysis. We performed coverage-based rarefaction

for the root samples. In a matrix of family-level taxonomic compositions, relative read counts of each family were binarized with a threshold described in Methods and Supplementary Figure S12. To make the subsequent statistical analysis (energy landscape analysis) computationally feasible, we prioritized and selected families based on their contribution to overall community structure, evaluated by PerMANOVA (R^2; Supplementary Tables S8–S9). The family set showing the highest correspondence between the binarized pattern and abundance-based community structure was selected among candidate sets determined by their R^2 values. Energy landscape analysis was then conducted using the family-set with host plant genera (encoded as dummy variables) as explanatory variables. (**b**) Input data for the keystone exploration. From the original data matrix excluding OTUs annotated as a focal genus, coverage-based rarefaction was conducted. Binarization was applied to the same set of families as in (**a**). In parallel, the whole community matrix was rarefied, and the genus-level compositions were subjected to centered log-ratio (CLR) transformation. Energy landscape analysis was performed by including host plant genera (dummy variables) and CLR-transformed relative read counts (+1) of the focal genus as external factors. Two types of indices for identifying potential keystone taxa were calculated by comparing the landscape topography inferred in the absence of the focal species/taxon with that estimated under the assumption of an intermediate abundance level (25%, 50%, or 75% quantiles of abundance across samples in which the focal taxon was present) (Fig. 1g).

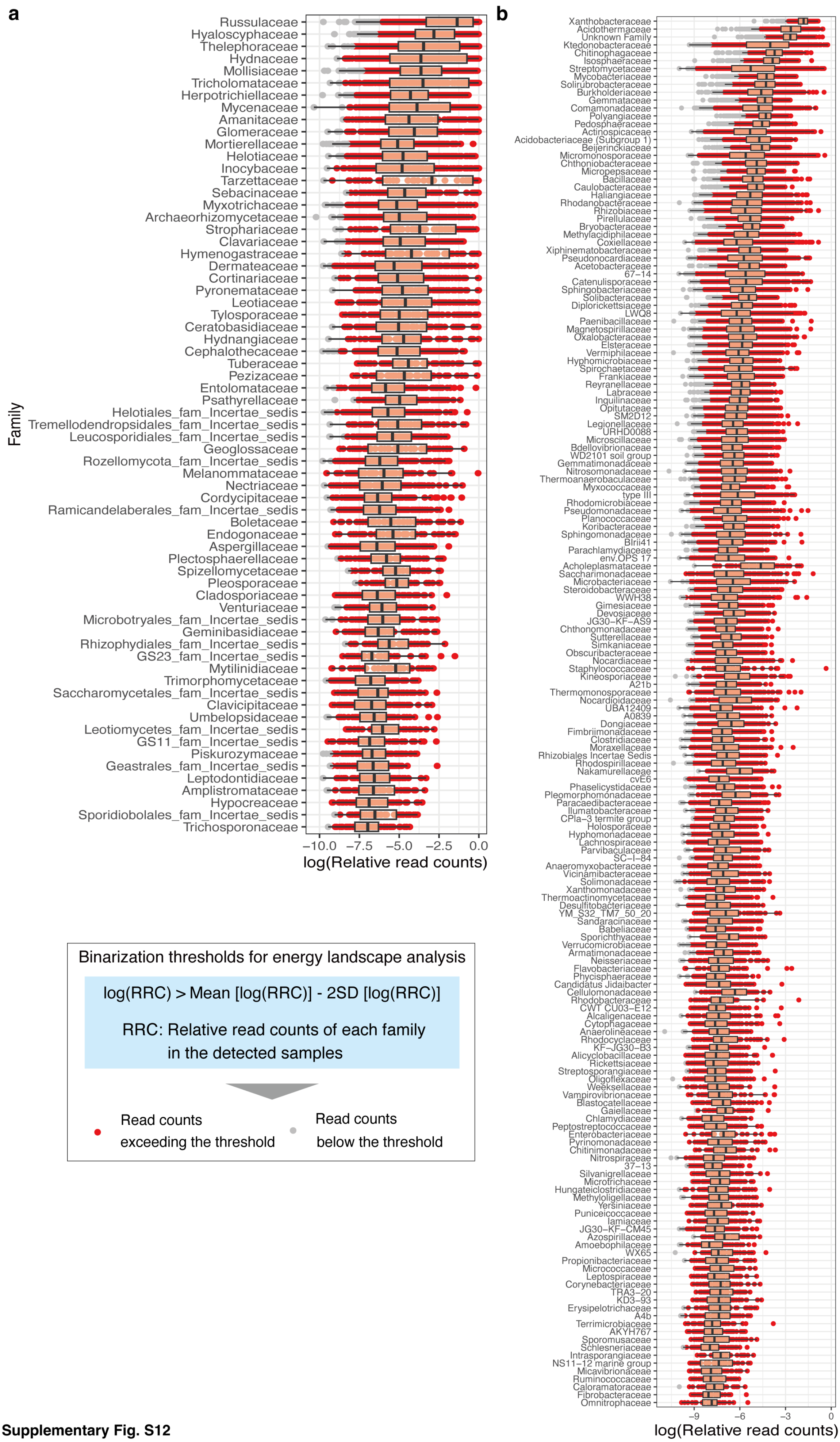

Supplementary Fig. S12

Supplementary Fig. S12 | Relative abundances of microbial families | (**a**) Distribution of relative read counts of each fungal family in samples from which it was detected. Each boxplot shows the distribution of relative read counts (RRC) of the focal family across these samples. The centre lines indicate the median (50% quantile), the lower and upper bounds of the boxes indicate the 25% and 75% quantiles, respectively, and the whiskers extend to the smallest and largest observations within 1.5 × the interquartile range (IQR) from the lower and upper quartiles, respectively. To remove potential sequencing contaminants, the threshold for binarization was defined for each family as Mean [log (RRC)] – 2SD [log (RRC)], where Mean [log (RRC)] and SD [log (RRC)] denote the mean and standard deviation of the log-transformed relative read counts of the focal family, respectively. (**b**) Distribution of relative read counts for each prokaryotic family. Source data are provided as a Source Data file.

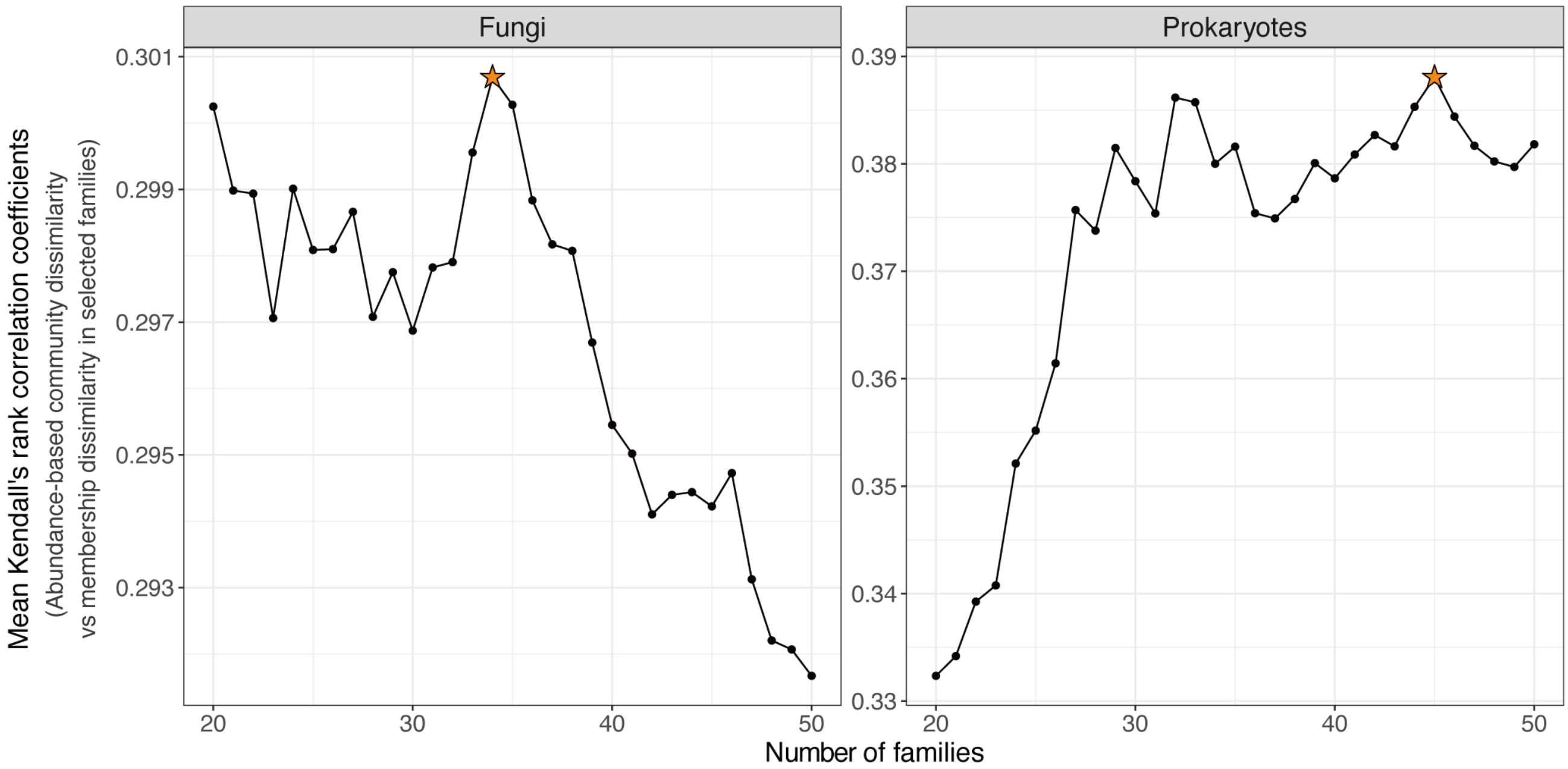

Supplementary Fig. S13 | Selecting optimal family sets for energy landscape analysis. The optimal sets (numbers) of fungal and prokaryotic families were determined to best preserve the information contained in the original community data during the filtering process for energy landscape analysis (see Supplementary Fig. S11 and Methods for details). Specifically, to make the exploration of optimal fungal and prokaryotic family sets computationally feasible, we first ordered 50 families based on their explanatory power for the overall community structure (R^2 in the PerMANOVA; see Supplementary Tables S8–S9). We then evaluated the correlations between community dissimilarity values calculated from the binarized data of each candidate family set (Jaccard dissimilarity) and those calculated from the original relative abundance data (Bray–Curtis dissimilarity). The selected fungal and prokaryotic family sets are indicated by star-shaped symbols. Source data are provided as a Source Data file.

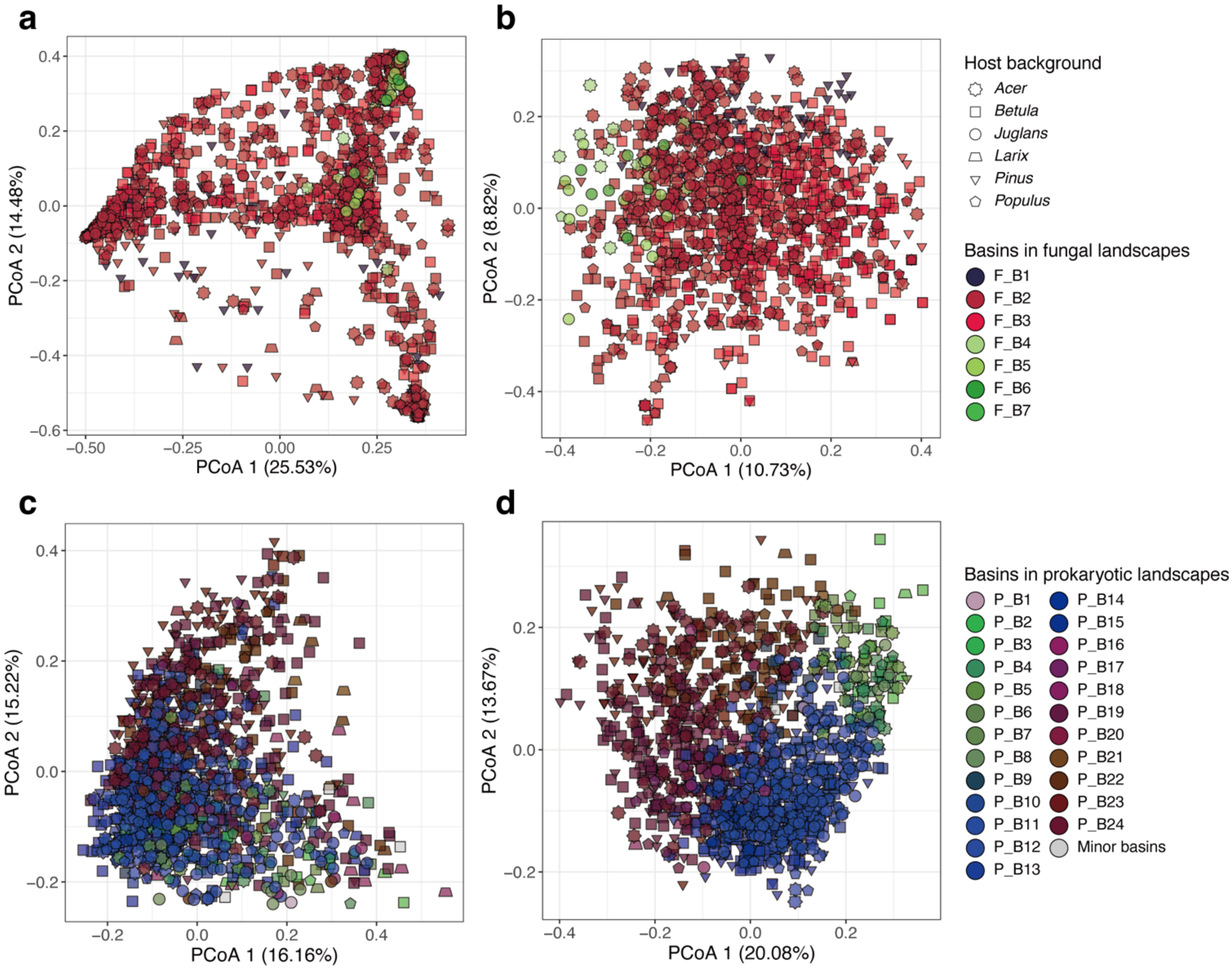

Supplementary Fig. S14 | Relationships between community structures and inferred basins. (**a, b**) Principal coordinate analysis (PCoA) based on Bray–Curtis dissimilarity of fungal community composition (**a**) and Jaccard dissimilarity of family memberships used in the energy landscape analysis (**b**). Basin assignments of individual root samples inferred by the energy landscape analysis are indicated by the corresponding colors (Fig. 2c). (**c, d**) PCoA of family-level prokaryotic community compositions (**c**) and family memberships (**d**). Basins to which more than 1% of samples were assigned are shown using the original colors (Fig. 3c), whereas the remaining basins are grouped and presented as Minor basins. Source data are provided as a Source Data file.

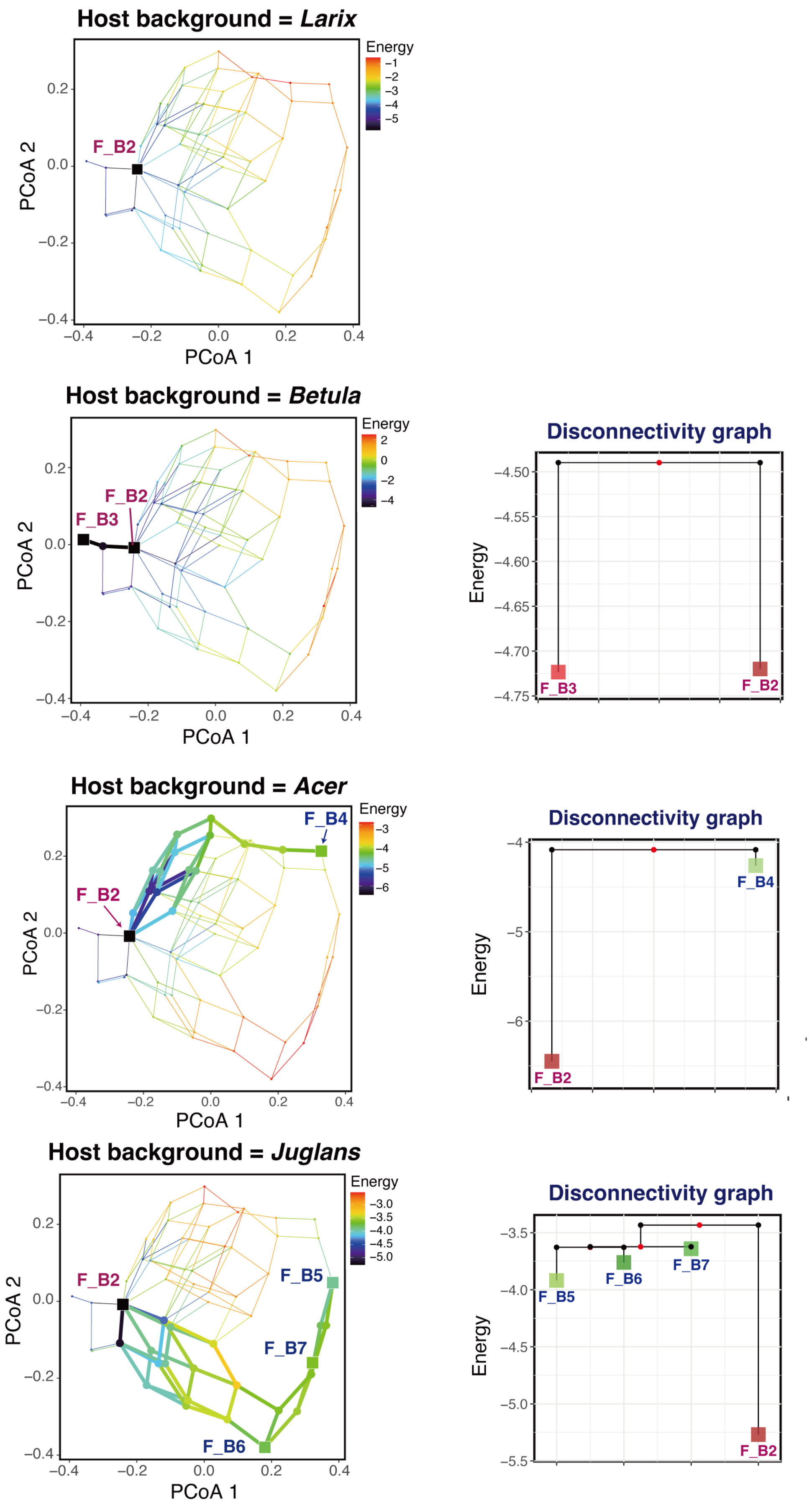

Supplementary Fig. S15

Supplementary Fig. S15 | Energy landscapes of the fungal community inferred by assuming *Larix, Betula, Acer* or *Juglans* host-plant backgrounds. The identity of host plants is included as an explicit factor in the statistical model (Fig. 1b). We calculate energy of each community state and reconstructed energy-weighted assembly graph in each host-plant background. The basin bottoms (squares), the intermediate states (circles) and the lowest-energy transition pathways between them (thick lines) are visualized on the PCoA surface representing community states. Pathways and intermediates states for the basin bottoms inferred in other host-plant backgrounds are shown by thin lines and small circles, respectively. The energy of basin bottoms and boundary states between them is detailed in the disconnectivity graphs. Source data are provided as a Source Data file.

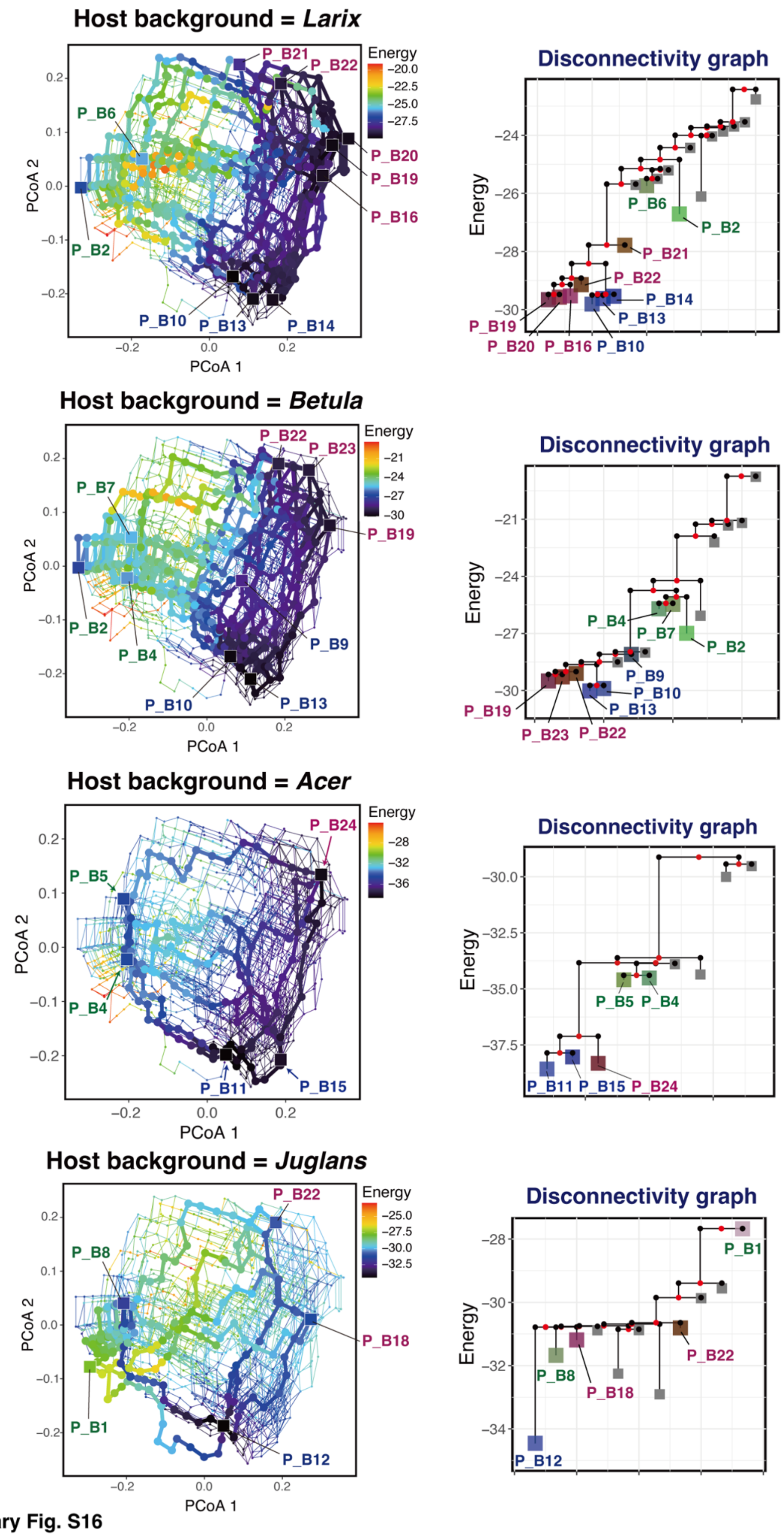

Supplementary Fig. S16 | Energy landscapes of the prokaryotic community inferred by assuming *Larix, Betula, Acer* or *Juglans* host-plant backgrounds. The identity of host plants is

included as an explicit factor in the statistical model (Fig. 1b). We calculate energy of each community state and reconstructed energy-weighted assembly graph in each host-plant background. The basin bottoms (squares), the intermediate states (circles) and the lowest-energy transition pathways between them (thick lines) are visualized on the PCoA surface representing community states. Pathways and intermediates states for the basin bottoms inferred in other host-plant backgrounds are shown by thin lines and small circles, respectively. The energy of basin bottoms and boundary states between them is detailed in the disconnectivity graphs. Source data are provided as a Source Data file.

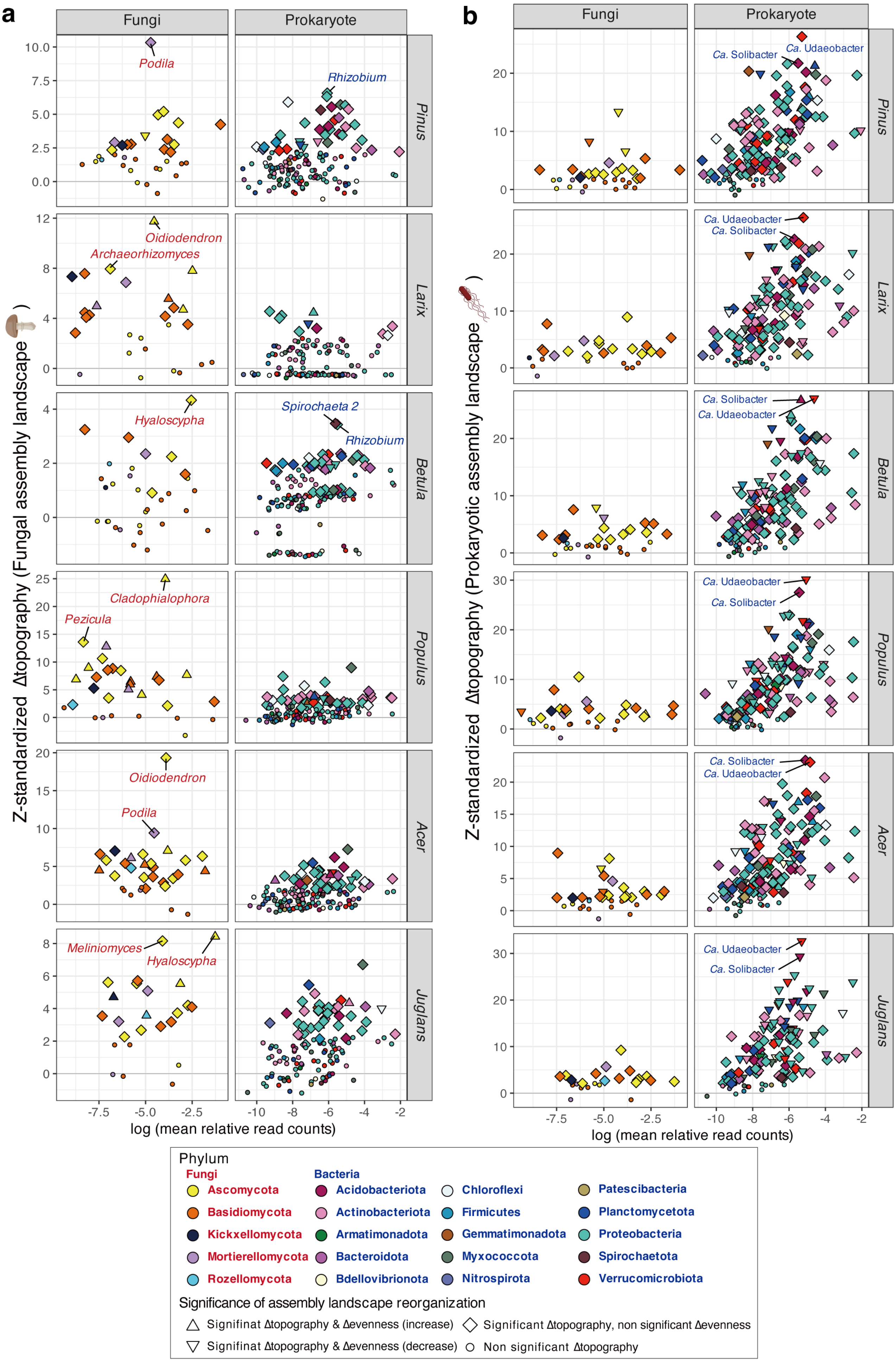

Supplementary Fig. S17

Supplementary Fig. S17 | Potential impacts on the overall energy landscape architecture and the abundance of each microbial genus. (a) Z-standardized Δtopography of individual microbial genera in fungal energy landscapes. Genera whose abundance changes are inferred to substantially reshape the fungal community destinations within the energy landscape are highlighted in each host-plant background. The effects of fungal and prokaryotic genera are presented separately (left-side panels: fungal genera, right-side panels: prokaryotic genera). (b) Z-standardized Δtopography of individual microbial genera in prokaryotic energy landscapes. Source data are provided as a Source Data file. Fungal illustration (a): Created in BioRender. Toju, H. (2026) https://BioRender.com/2rdd0th. Bacterial illustration (b): Created in BioRender. Toju, H. (2026) https://BioRender.com/w6b4bi9.

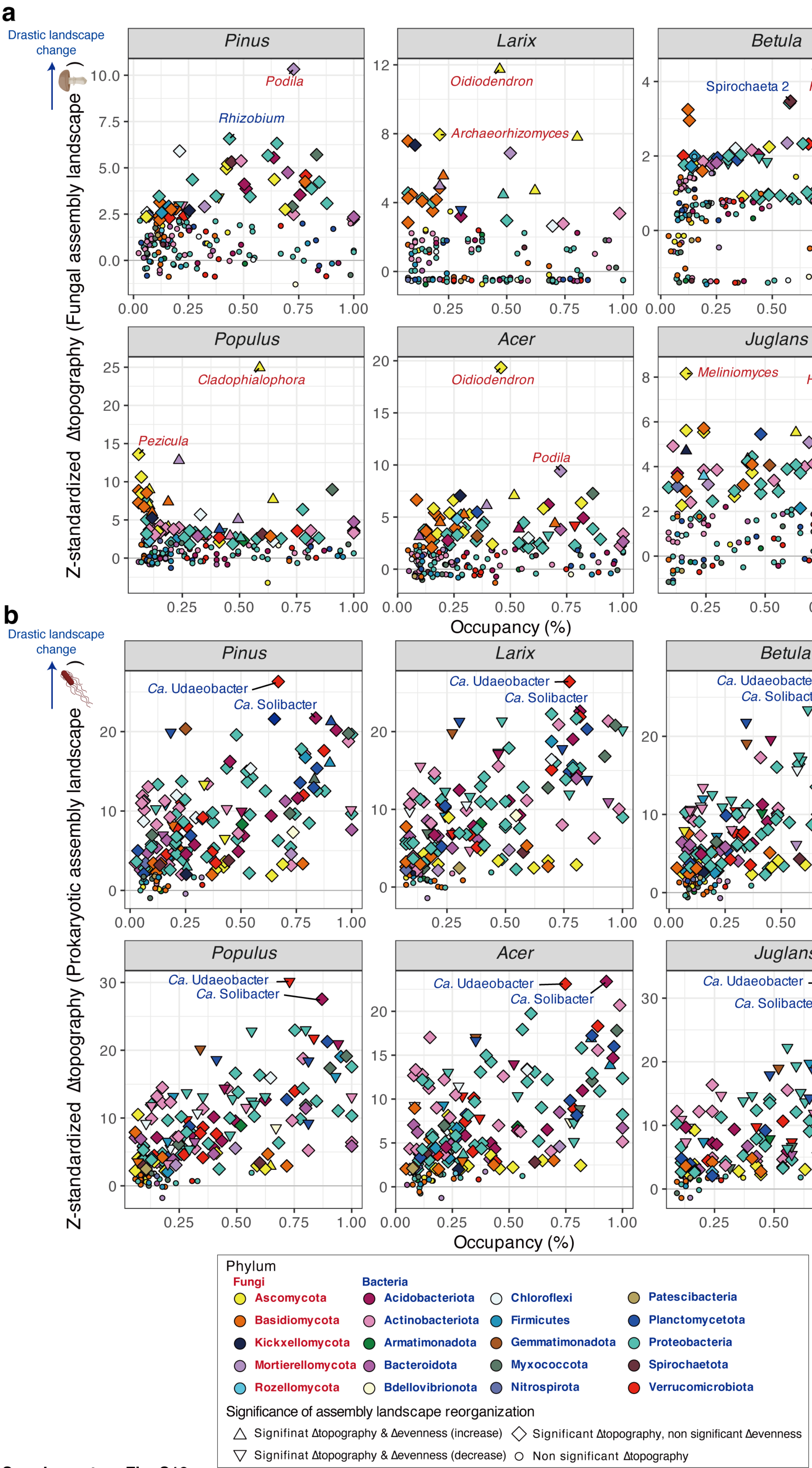

Supplementary Fig. S18

Supplementary Fig. S18 | Potential impacts on the overall energy landscape architecture and the occupancy of each microbial genus. (**a**) Z-standardized Δtopography of individual microbial genera in fungal energy landscapes. Genera whose abundance changes are inferred to substantially reshape the fungal community destinations within the energy landscape are highlighted in each host plant background. (**b**) Z-standardized Δtopography of individual microbial genera in prokaryotic energy landscapes. Source data are provided as a Source Data file. Fungal illustration (a): Created in BioRender. Toju, H. (2026) https://BioRender.com/2rdd0th. Bacterial illustration (b): Created in BioRender. Toju, H. (2026) https://BioRender.com/w6b4bi9.

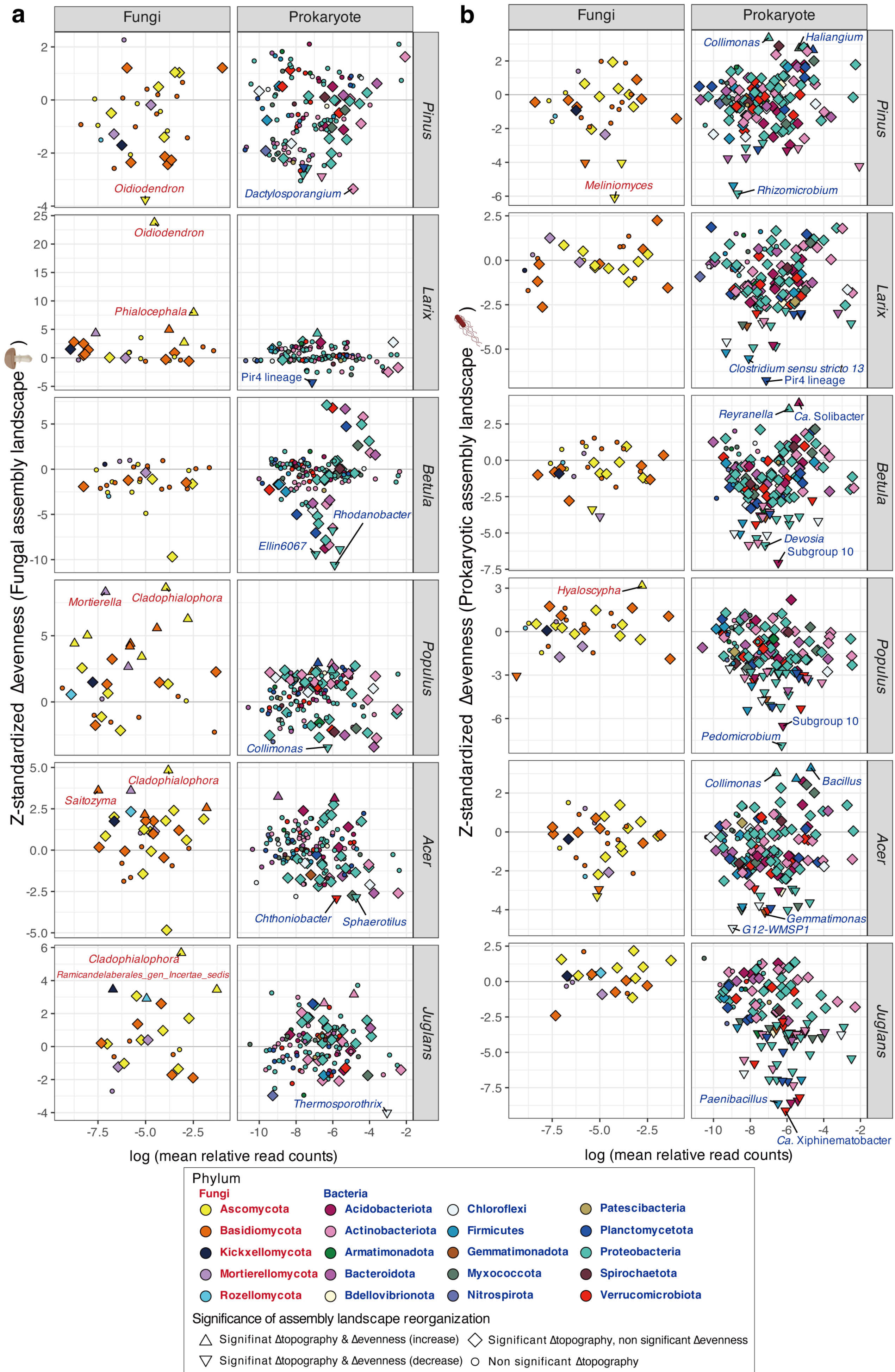

Supplementary Fig. S19

Supplementary Fig. S19 | Potential impacts on the evenness of the basin distributions and the relative abundance of each microbial genus. (**a**) Z-standardized Δevenness of individual microbial genera in fungal energy landscapes. Genera whose abundance changes are inferred to substantially alter the basin distributions and frequencies within the energy landscape are highlighted in each host plant background. The effects of fungal and prokaryotic genera are presented separately (left-side panels: fungal genera, right-side panels: prokaryotic genera). (**b**) Z-standardized Δevenness of individual microbial genera in prokaryotic energy landscapes. Source data are provided as a Source Data file. Fungal illustration (a): Created in BioRender. Toju, H. (2026) https://BioRender.com/2rdd0th. Bacterial illustration (b): Created in BioRender. Toju, H. (2026) https://BioRender.com/w6b4bi9.

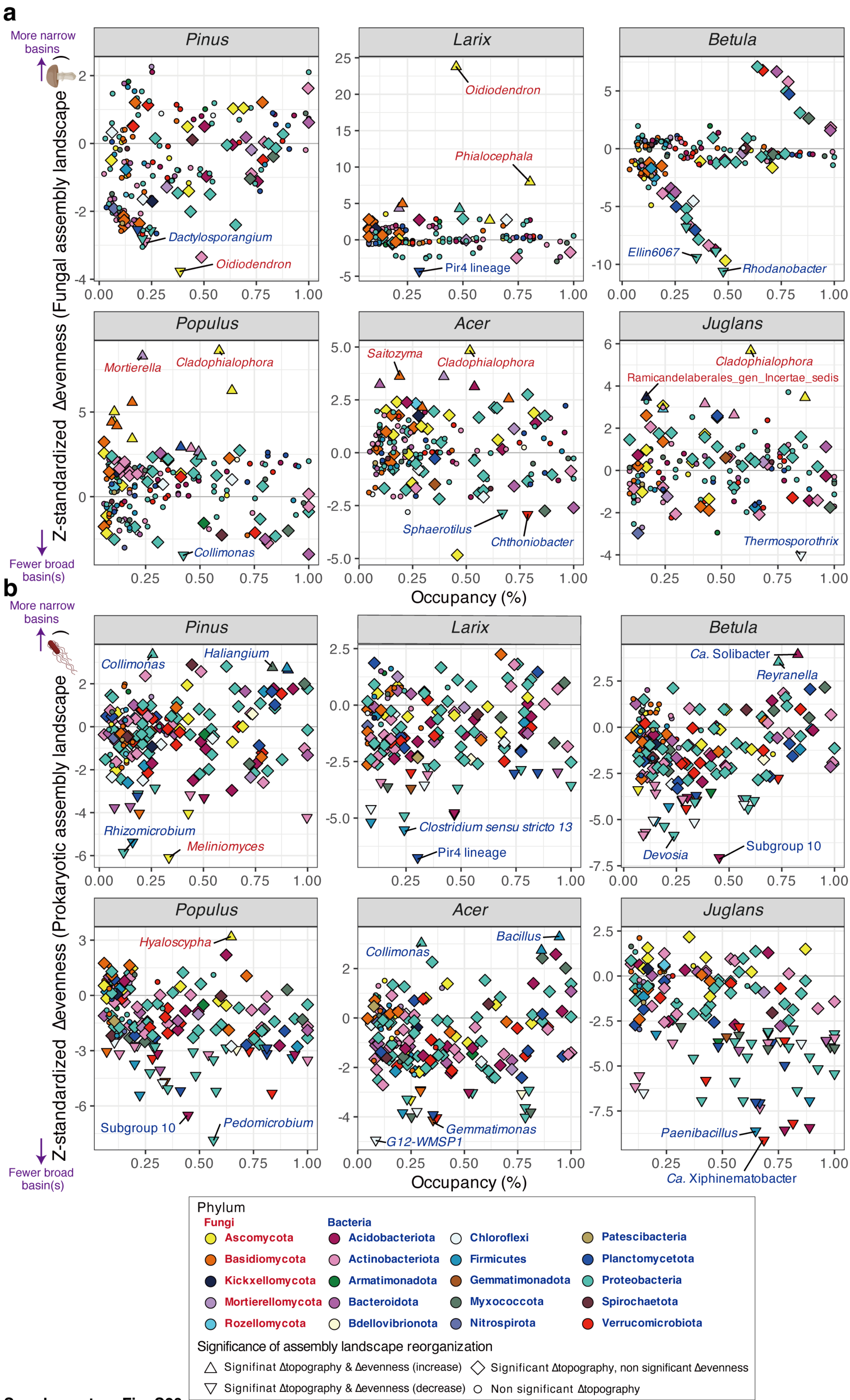

Supplementary Fig. S20

Supplementary Fig. S20 | Potential impacts on the evenness of the basin distributions and the occupancy of each microbial genus. (**a**) Z-standardized Δevenness of individual microbial genera in fungal energy landscapes. Genera whose abundance changes are inferred to substantially alter the basin distributions and frequencies within the energy landscape are highlighted in each host plant background. (**b**) Z-standardized Δevenness of individual microbial genera in prokaryotic energy landscapes. Source data are provided as a Source Data file. Fungal illustration (a): Created in BioRender. Toju, H. (2026) https://BioRender.com/2rdd0th. Bacterial illustration (b): Created in BioRender. Toju, H. (2026) https://BioRender.com/w6b4bi9.

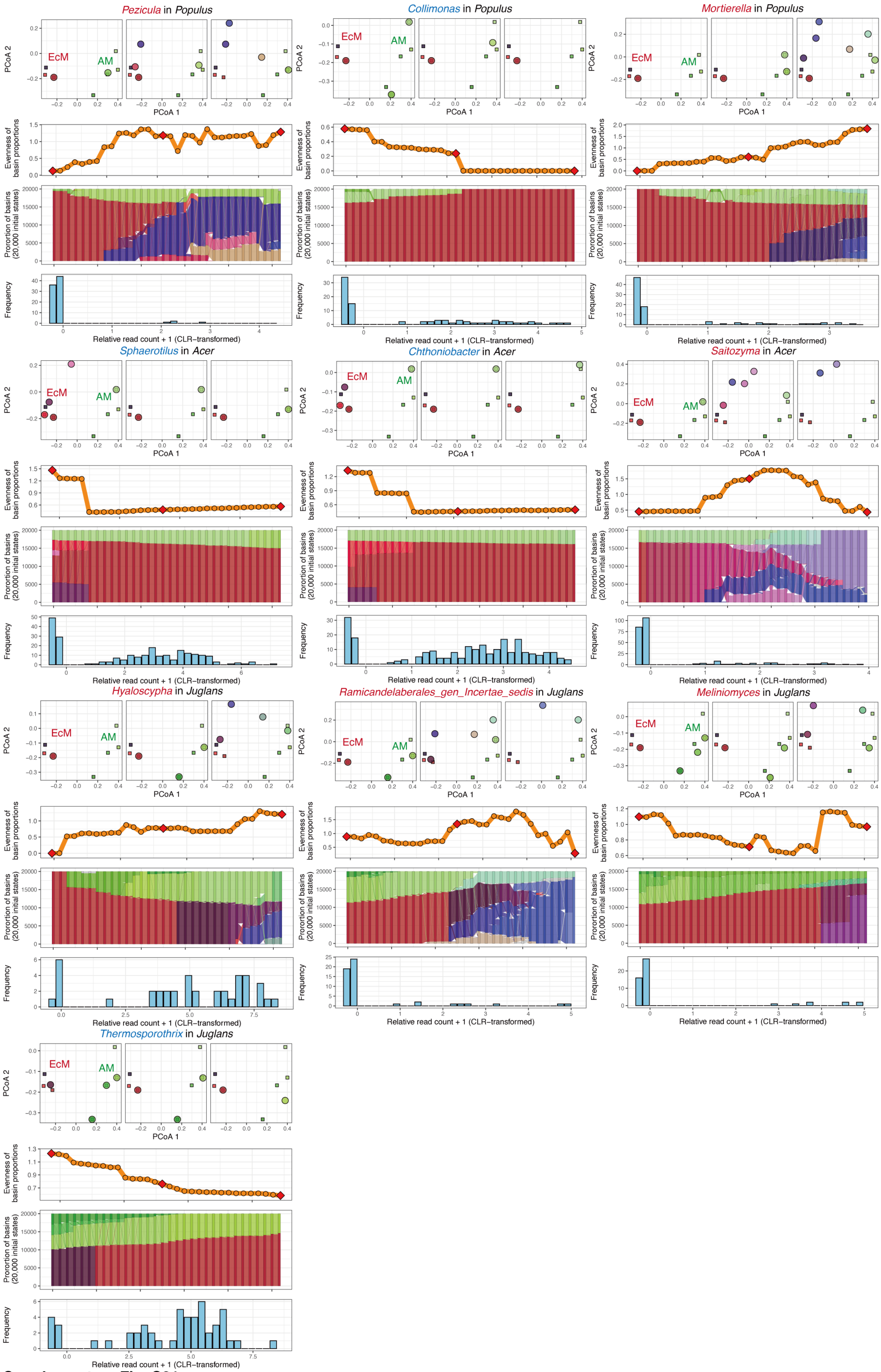

Supplementary Fig. S21

Supplementary Fig. S21 | Fungal energy landscape reorganization in the dual mycorrhizal host-plant backgrounds. Along the abundance gradient of each microbial genus highlighted in Figure 4b (*Populus*, *Acer* and *Juglans*), changes in the frequency distribution of fungal energy landscape basins are shown. Frequencies are evaluated through 20,000 simulations of community assemblies from randomly generated initial states, under 32 abundance conditions ranging from absence to the maximum observed abundance in the host. On the PCoA planes, the basin bottoms detected at three representative abundance levels within 32 equally spaced relative abundance steps—absence (step 1), intermediate abundance (step 16), and maximum abundance (step 32)—are shown as circles, together with the states detected in Figure 2c (see Supplementary Fig. S47a). The Shannon entropy of the basin frequencies is also shown along the abundance axis (centered log ratio (CLR) -transformed relative read counts of a focal genus). In addition, the destinations of the initial communities in each condition of the focal genus's abundance are indicated in the flow diagrams with the histograms of their relative abundances in the hosts. Each bar plot depicts the frequency of basin bottoms detected under the corresponding abundance condition. Basin bottoms reached from the same initial community state across adjacent abundance conditions (steps) are connected by bands. Colors represent community compositional similarity based on Jaccard dissimilarity, with more similar compositions rendered in more similar colors (see Fig. 2c; Supplementary Fig. S48). Source data are provided as a Source Data file.

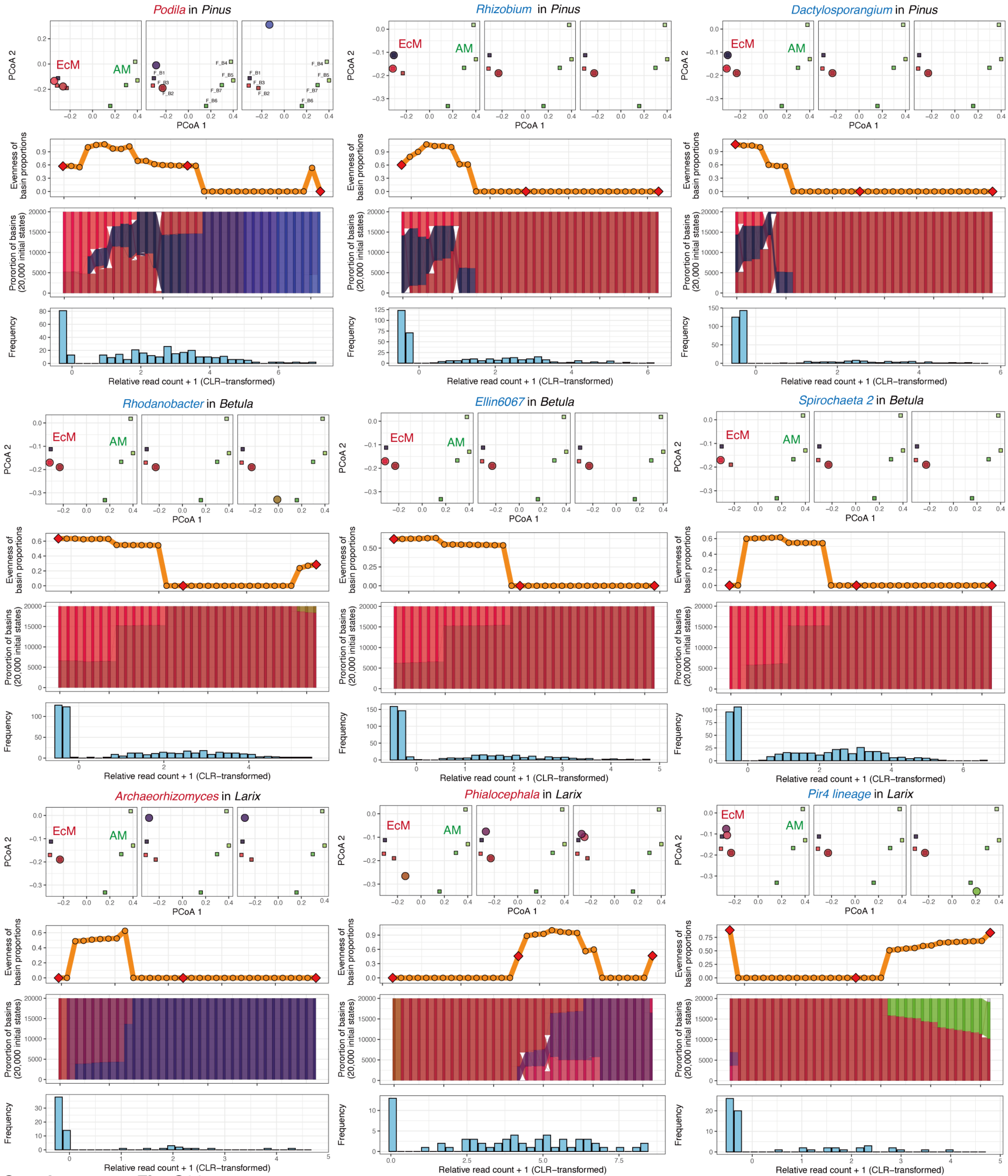

Supplementary Fig. S22

Supplementary Fig. S22 | Fungal energy landscape reorganization in the ectomycorrhizal host-plant backgrounds. Along the abundance gradient of each microbial genus highlighted in figure 4b (*Pinus*, *Betula* and *Larix*), changes in the frequency distribution of fungal energy landscape basins are shown. Frequencies are evaluated through 20,000 simulations of community assemblies from randomly generated initial states, under 32 abundance conditions ranging from absence to the maximum observed abundance in the host. On the PCoA planes, the basin bottoms detected at three representative abundance levels within 32 equally spaced relative abundance steps—absence (step 1),

intermediate abundance (step 16), and maximum abundance (step 32)—are shown as circles, together with the states detected in Figure 2c (see Supplementary Fig. S47a). The Shannon entropy of the basin frequencies is also shown along the abundance axis (centered log ratio (CLR) -transformed relative read counts of a focal genus). In addition, the destinations of the initial communities in each condition of the focal genus's abundance are indicated in the flow diagrams with the histograms of their relative abundances in the hosts. Each bar plot depicts the frequency of basin bottoms detected under the corresponding abundance condition. Basin bottoms reached from the same initial community state across adjacent abundance conditions (steps) are connected by bands. Colors represent community compositional similarity based on Jaccard dissimilarity, with more similar compositions rendered in more similar colors (see Fig. 2c; Supplementary Fig. S48). Source data are provided as a Source Data file.

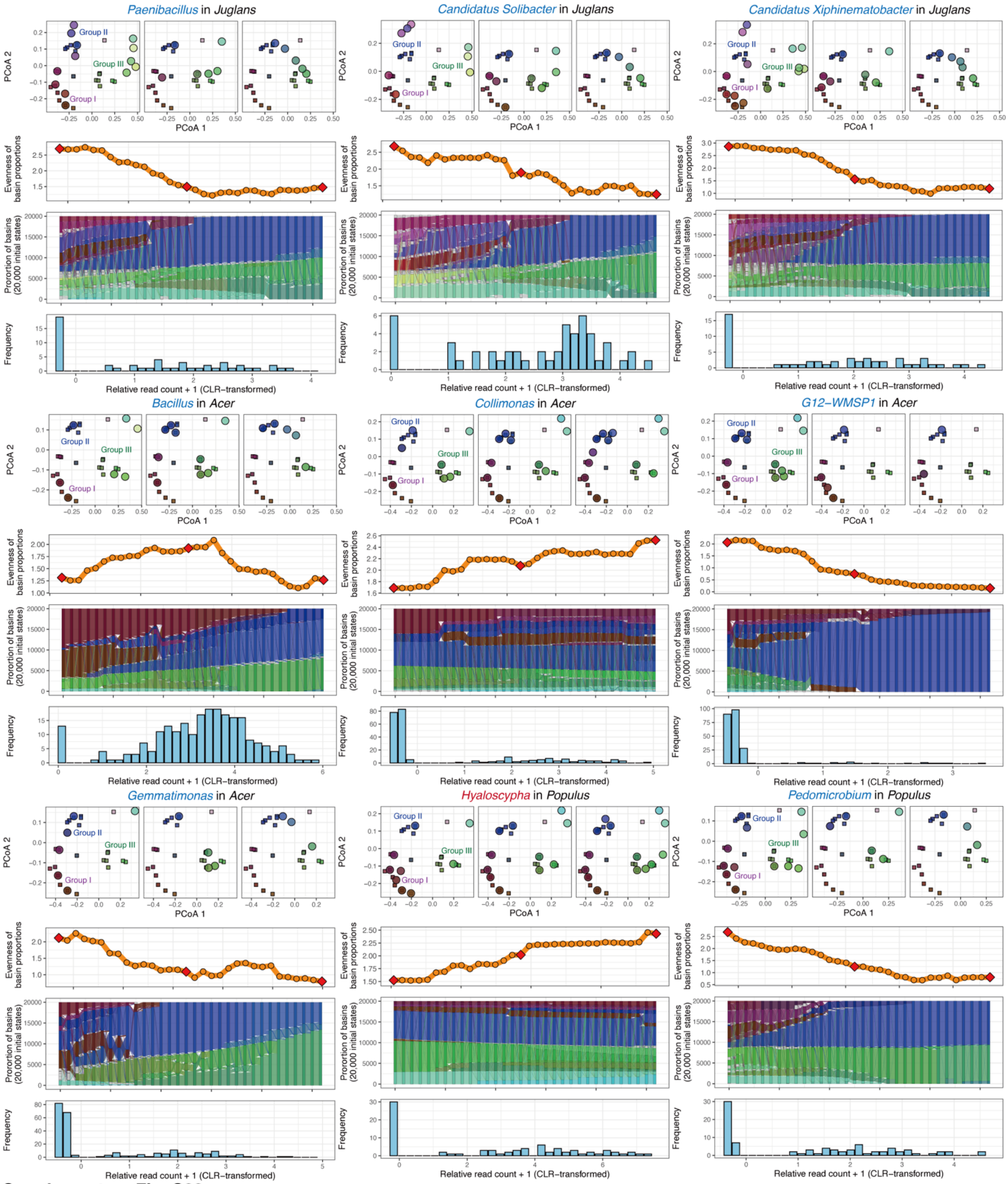

Supplementary Fig. S23

Supplementary Fig. S23 | Prokaryotic energy landscape reorganization in the dual mycorrhizal host-plant backgrounds. Along the abundance gradient of each microbial genus highlighted in Figure 4d (*Juglans*, *Acer* and *Populus*), changes in the frequency distribution of prokaryotic energy landscape basins are shown. Frequencies are evaluated through 20,000 simulations of community assemblies from randomly generated initial states, under 32 abundance conditions ranging from absence to the maximum observed abundance in the host. On the PCoA planes, the basin bottoms detected at three representative abundance levels within 32 equally spaced relative abundance steps—

absence (step 1), intermediate abundance (step 16), and maximum abundance (step 32)—are shown as circles, together with the states detected in Figure 3c (see Supplementary Fig. S47b). The Shannon entropy of the basin frequencies is also shown along the abundance axis (centered log ratio (CLR) -transformed relative read counts of a focal genus). In addition, the destinations of the initial communities in each condition of the focal genus's abundance are indicated in the flow diagrams with the histograms of their relative abundances in the hosts. Each bar plot depicts the frequency of basin bottoms detected under the corresponding abundance condition. Basin bottoms reached from the same initial community state across adjacent abundance conditions (steps) are connected by bands. Colors represent community compositional similarity based on Jaccard dissimilarity, with more similar compositions rendered in more similar colors (see Fig. 3c; Supplementary Fig. S49). Source data are provided as a Source Data file.

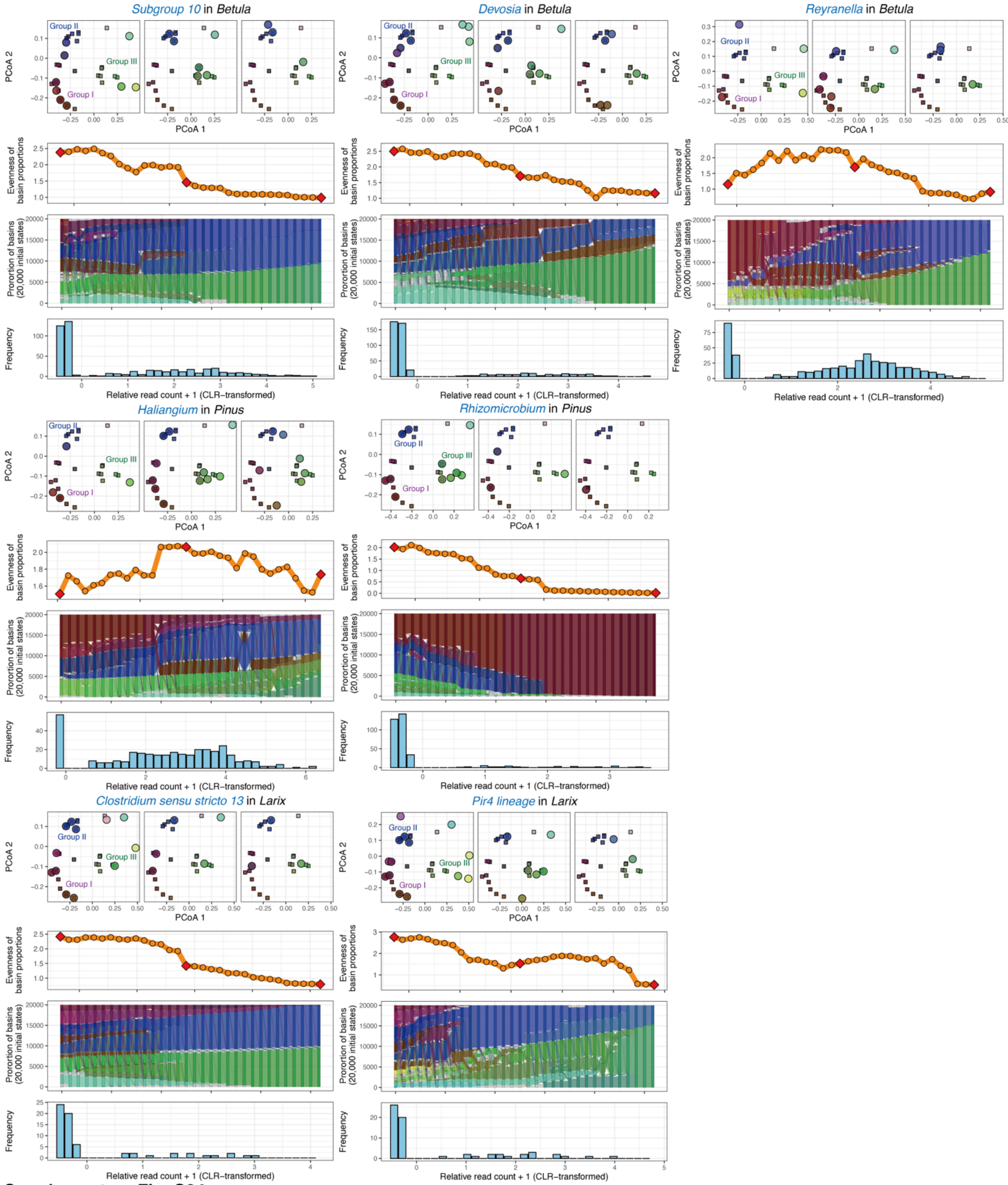

Supplementary Fig. S24

Supplementary Fig. S24 | Prokaryotic energy landscape reorganization in the ectomycorrhizal host-plant backgrounds. Along the abundance gradient of each microbial genus highlighted in Figure 4d (*Betula, Pinus*, and *Larix*), changes in the frequency distribution of prokaryotic energy landscape basins are shown. Frequencies are evaluated through 20,000 simulations of community assemblies from randomly generated initial states, under 32 abundance conditions ranging from absence to the maximum observed abundance in the host. On the PCoA planes, the basin bottoms detected at three representative abundance levels within 32 equally spaced relative abundance steps—

absence (step 1), intermediate abundance (step 16), and maximum abundance (step 32)—are shown as circles, together with the states detected in Figure 3c (see Supplementary Fig. S47b). The Shannon entropy of the basin frequencies is also shown along the abundance axis (centered log ratio (CLR) - transformed relative read counts of a focal genus). In addition, the destinations of the initial communities in each condition of the focal genus's abundance are indicated in the flow diagrams with the histograms of their relative abundances in the hosts. Each bar plot depicts the frequency of basin bottoms detected under the corresponding abundance condition. Basin bottoms reached from the same initial community state across adjacent abundance conditions (steps) are connected by bands. Colors represent community compositional similarity based on Jaccard dissimilarity, with more similar compositions rendered in more similar colors (see Fig. 3c; Supplementary Fig. S49). Source data are provided as a Source Data file.

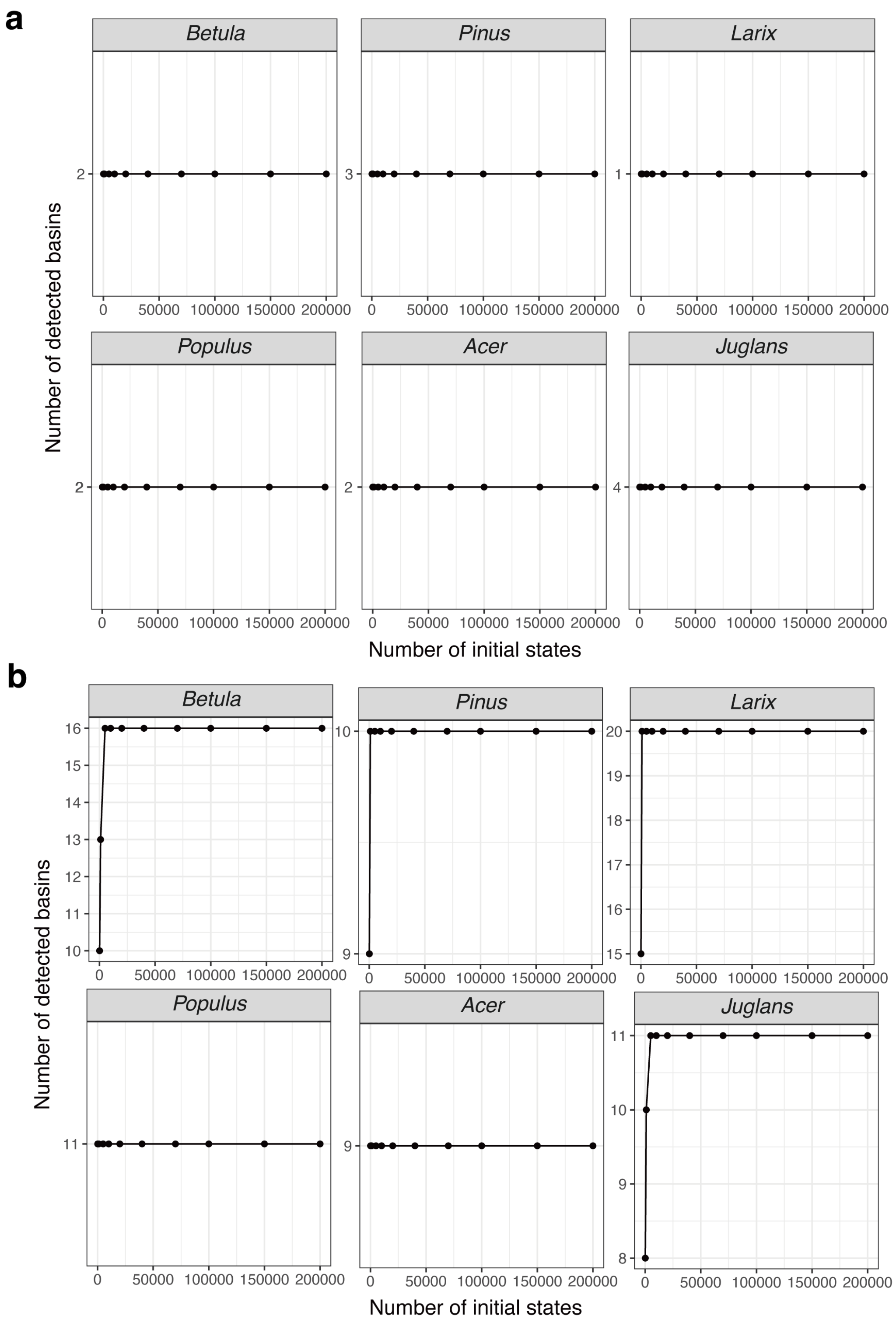

Supplementary Fig. S25 | Evaluation of the number of initial states required for robust basin detection in energy landscape analysis. To examine whether the number of initial community states used in steepest-descent simulations influences basin detection, we repeated basin searches using increasing numbers of initial states (100–200,000) for each host-plant background. (**a**) Numbers of detected basins in fungal assembly landscapes across six host-plant backgrounds. (**b**) Numbers of detected basins in prokaryotic assembly landscapes across the same host-plant backgrounds. Source data are provided as a Source Data file.

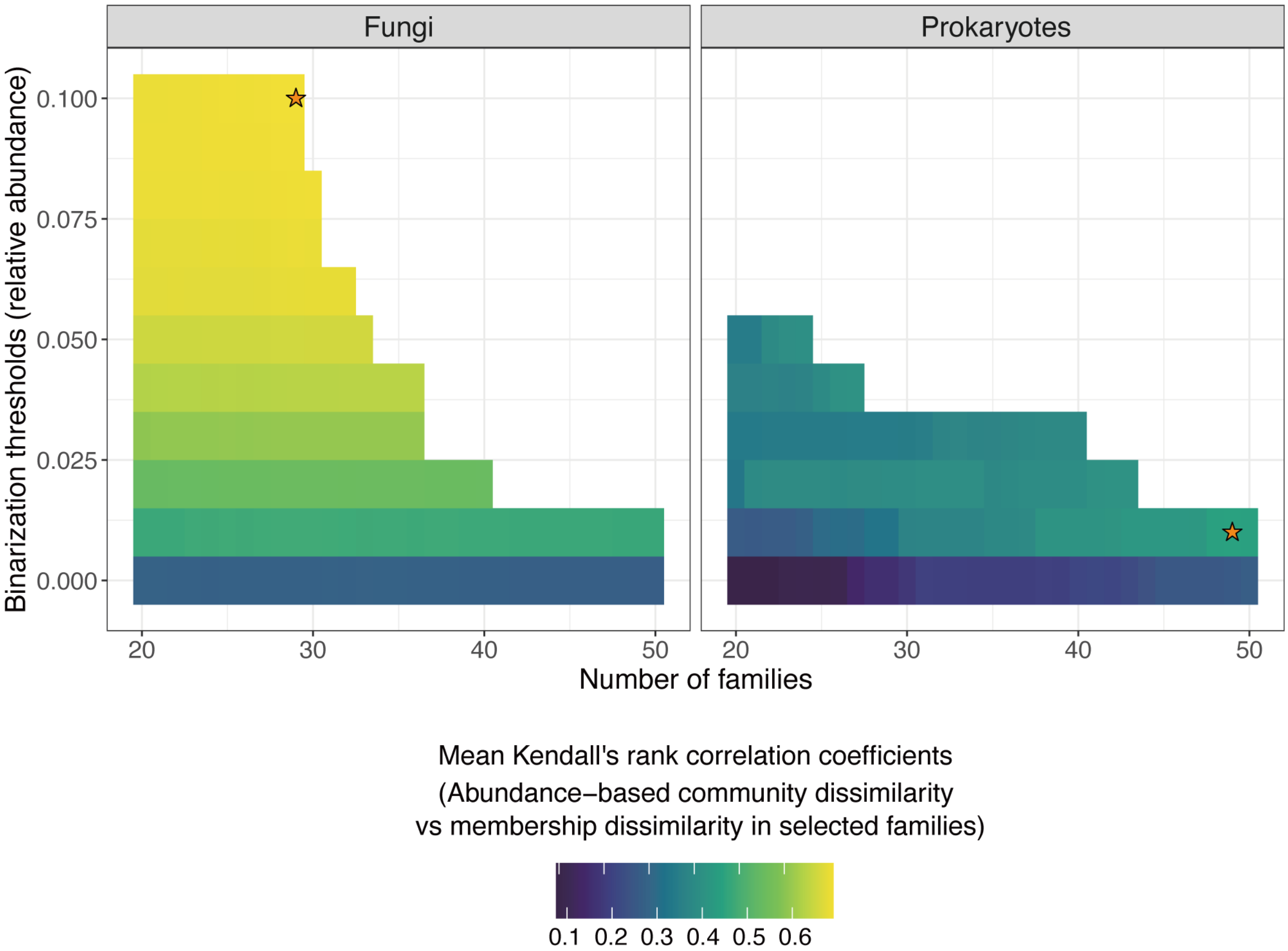

Supplementary Fig. S26 | Identification of optimal family sets under uniform relative-abundance binarization thresholds. To evaluate whether uniform relative-abundance thresholds can preserve community structure during binarization, we explored combinations of abundance thresholds and numbers of retained families for fungal and prokaryotic datasets (see Supplementary Note 4 for details). Community matrices were binarized using 30 candidate abundance thresholds, after which families were ranked by their explanatory power for overall community structure (PerMANOVA R^2; Supplementary Tables S10–S11). For each candidate family set, we quantified how well binary community dissimilarities (Jaccard dissimilarity) preserved the structure of the original abundance-based data (Bray–Curtis dissimilarity). Star symbols indicate the selected optimal combinations of abundance threshold and family number used for comparison with the primary binarization approach. Source data are provided as a Source Data file.

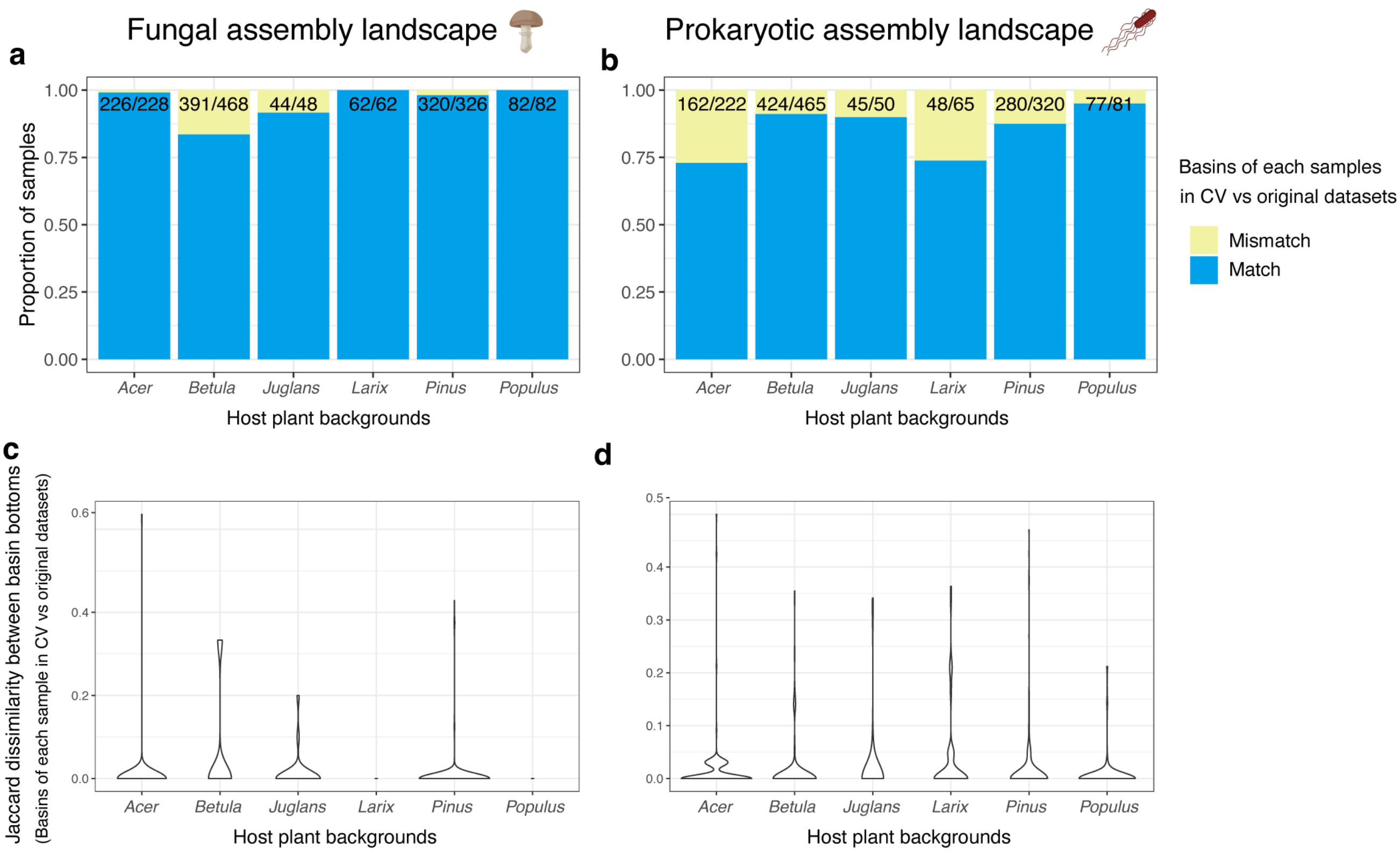

Supplementary Fig. S27 | Cross-validation assessment of predictive robustness in basin assignments. To evaluate the predictive robustness of basin assignments inferred by energy landscape analysis, we conducted 100-fold cross-validation (CV) of assembly landscape inference. For each fold, models were trained using datasets excluding focal samples, and basin assignments for the excluded samples were predicted using the fitted models. The proportions of samples assigned to identical basins in the full-dataset and cross-validated inferences are shown for fungal (**a**) and prokaryotic (**b**) assembly landscapes. To further assess consistency in inferred community states, we quantified differences in basin-bottom compositions between full-dataset and cross-validation analyses using Jaccard dissimilarity (fungal landscapes, **c**; prokaryotic landscapes, **d**). Source data are provided as a Source Data file. Fungal illustration (a): Created in BioRender. Toju, H. (2026) https://BioRender.com/2rdd0th. Bacterial illustration (b): Created in BioRender. Toju, H. (2026) https://BioRender.com/w6b4bi9.

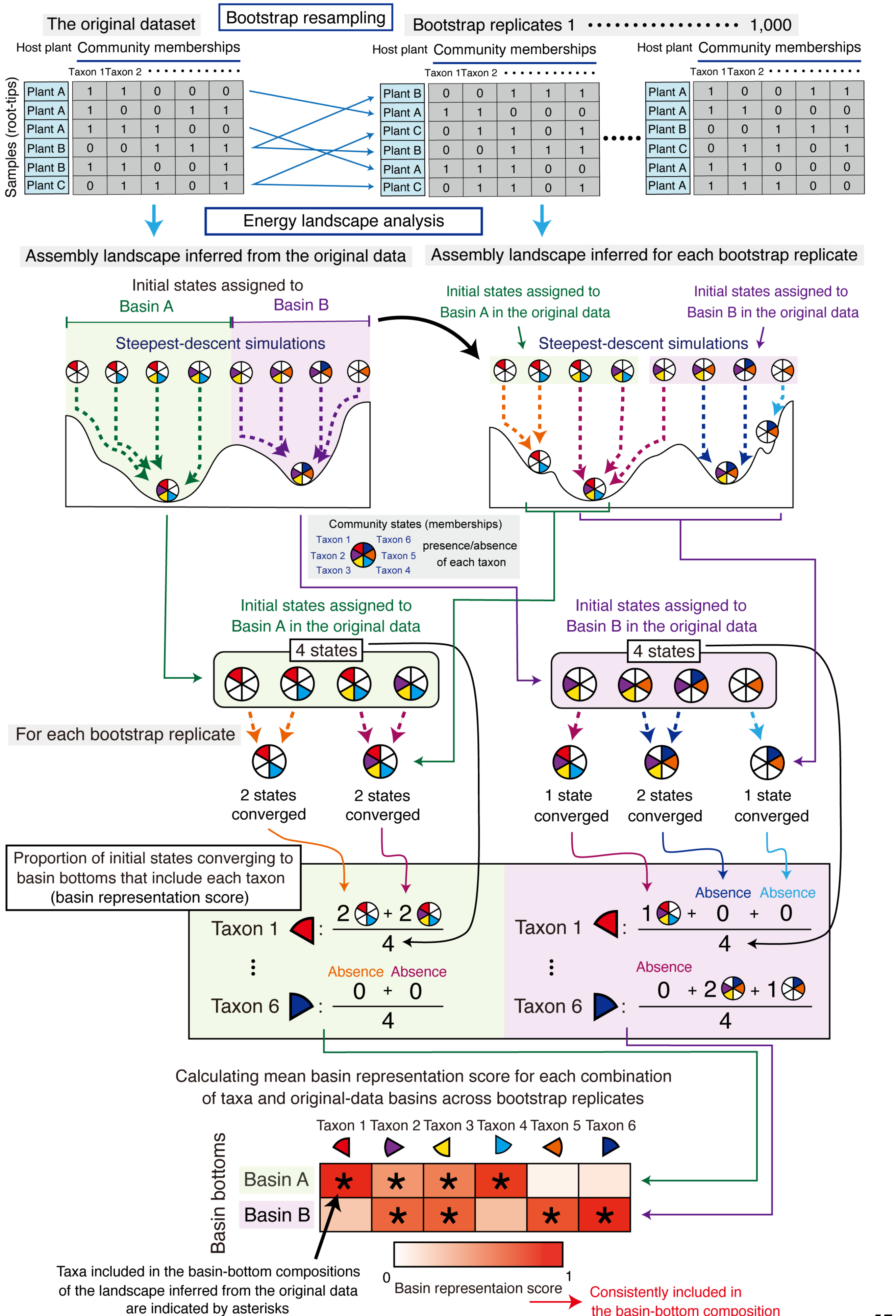

Supplementary Fig. S28

Supplementary Fig. S28 | Workflow for evaluating consistency in the estimation of basin bottoms. To assess how sampling variability influences inferred basin-bottom compositions, bootstrap resampling of community state matrices was performed for both root-tip fungal and prokaryotic datasets (1,000 replicates). For each bootstrap replicate, the full energy landscape inference procedure was repeated. Basin bottoms were identified using steepest-descent simulations initiated from an identical set of 150,000 initial community states for both the assembly landscape inferred from the original dataset and those inferred from bootstrap replicates. Using the same initial states allowed direct comparison of the basin bottom reached from a given initial community state across the original and bootstrap landscapes. For each basin bottom identified in the original landscape, we collected the set of initial states that converged to that basin in the original landscape and then tracked the basin bottoms reached from those same initial states in each bootstrap replicate. For each microbial taxon (family), we calculated the proportion of cases in which the basin bottom reached from those initial states contained the focal taxon (basin representation score). We then calculated mean basin representation scores for each microbial taxon and each original-data basin across bootstrap replicates.

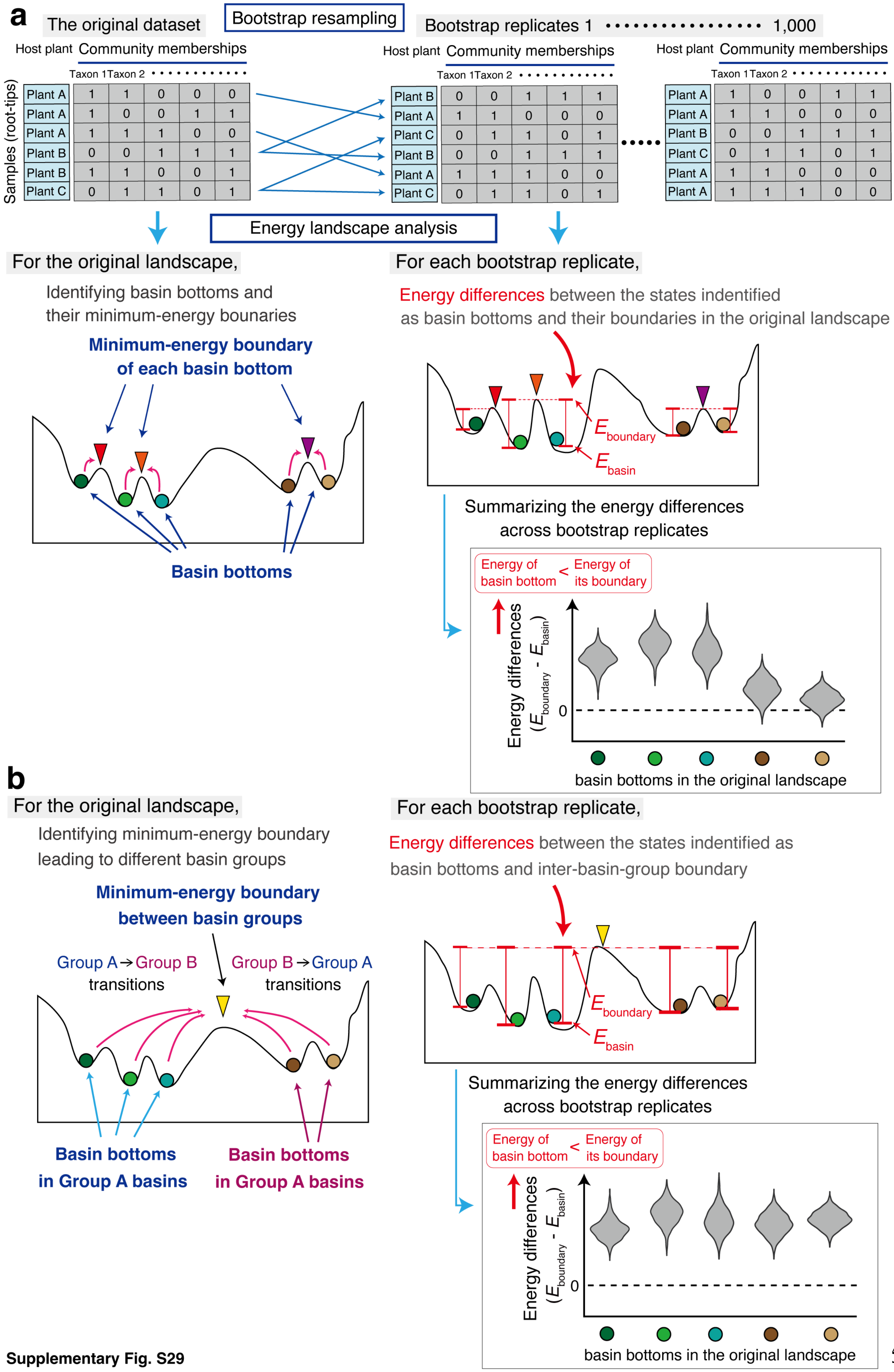

Supplementary Fig. S29

Supplementary Fig. S29 | Workflow for evaluating consistency in the estimation of assembly landscape topography. (**a**) Bootstrap procedure for evaluating the influence of sampling variability on landscape topography. In this bootstrap analysis, resampling of community-state matrices was performed for both root-tip fungal and prokaryotic datasets (1,000 replicates). For each bootstrap replicate, we evaluated energy differences between community states corresponding to basin bottoms and their corresponding lowest-energy boundary states defined in the original landscape. Because energy values are comparable only among community states within the same energy landscape, the energies of these community states were recalculated under each bootstrap-inferred landscape before comparison. A positive value of this energy-difference index indicates that the relative ordering between basin-bottom states and boundary states was preserved across bootstrap landscapes. (**b**) Bootstrap procedure for evaluating the influence of sampling variability on large compositional transitions between major basin groups. Energy differences between community states corresponding to basin bottoms and boundary states separating basin groups were evaluated using the same framework. Transitions between arbuscular mycorrhizal and ectomycorrhizal basins (Fig. 2c) were examined for fungal communities, whereas transitions between Group I, II, and III basins (Fig. 3c) were examined for prokaryotic communities.

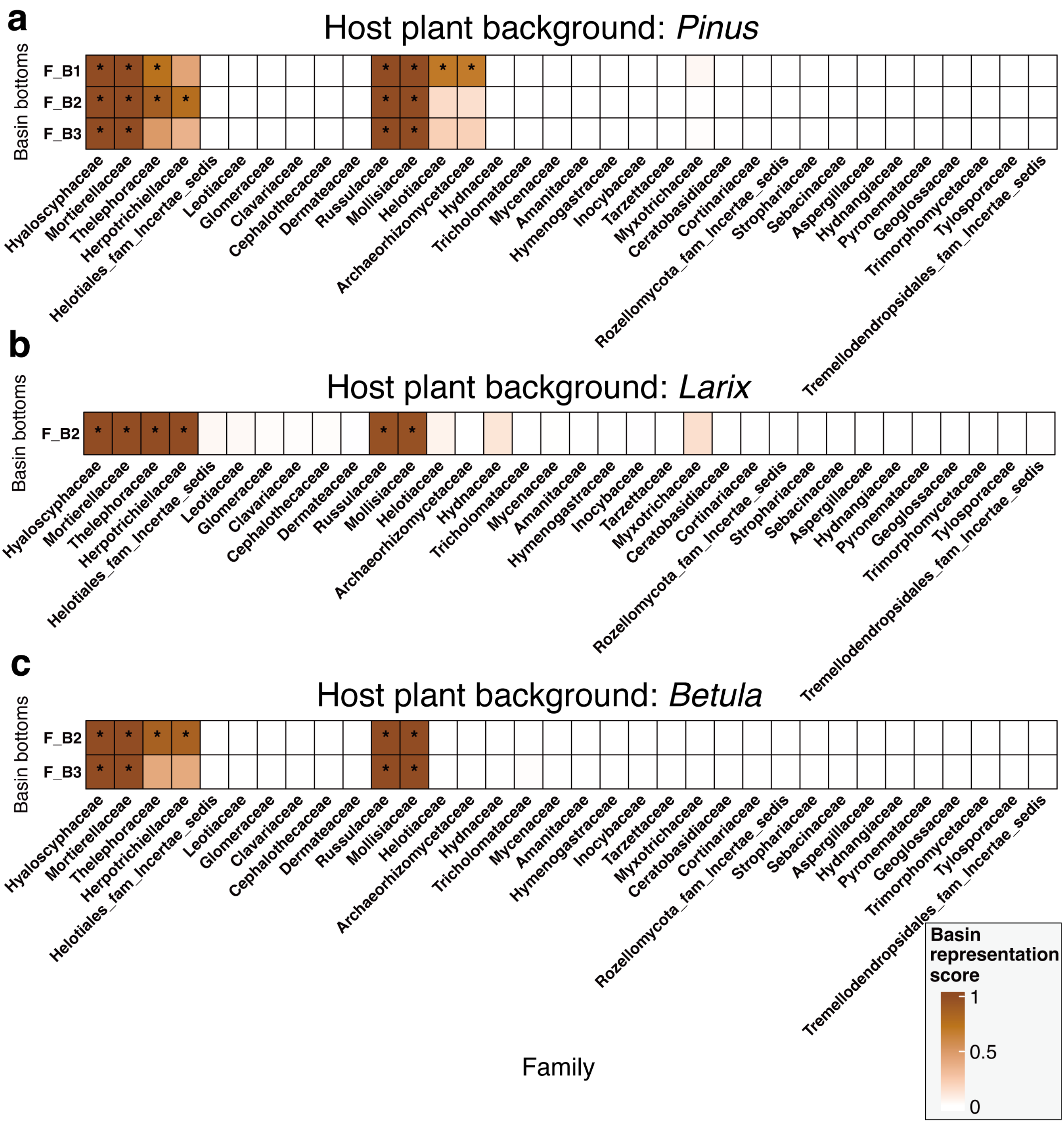

Supplementary Fig. S30 | Basin representation scores for family memberships of basin bottoms in fungal assembly landscapes under ectomycorrhizal host backgrounds. To evaluate the robustness of taxonomic compositions defining basin bottoms, we performed bootstrap resampling of samples (1,000 replicates) followed by repeated energy landscape inference. Analyses focused on assembly landscapes inferred for ectomycorrhizal host plants: *Pinus* (**a**), *Larix* (**b**), and *Betula* (**c**). Steepest-descent simulations were conducted using the same initial community states as those applied in the original analyses (Fig. 2c) to enable direct comparison among corresponding basins. Basin representation score for each family was quantified as the mean proportion of basin bottoms identified in bootstrap replicates that contained the focal family among states assigned to the corresponding basin in the original inference (Supplementary Fig. S28; see Supplementary Note 8 for details). The

families included in each basin in the original inference are indicated with asterisks. Source data are provided as a Source Data file.

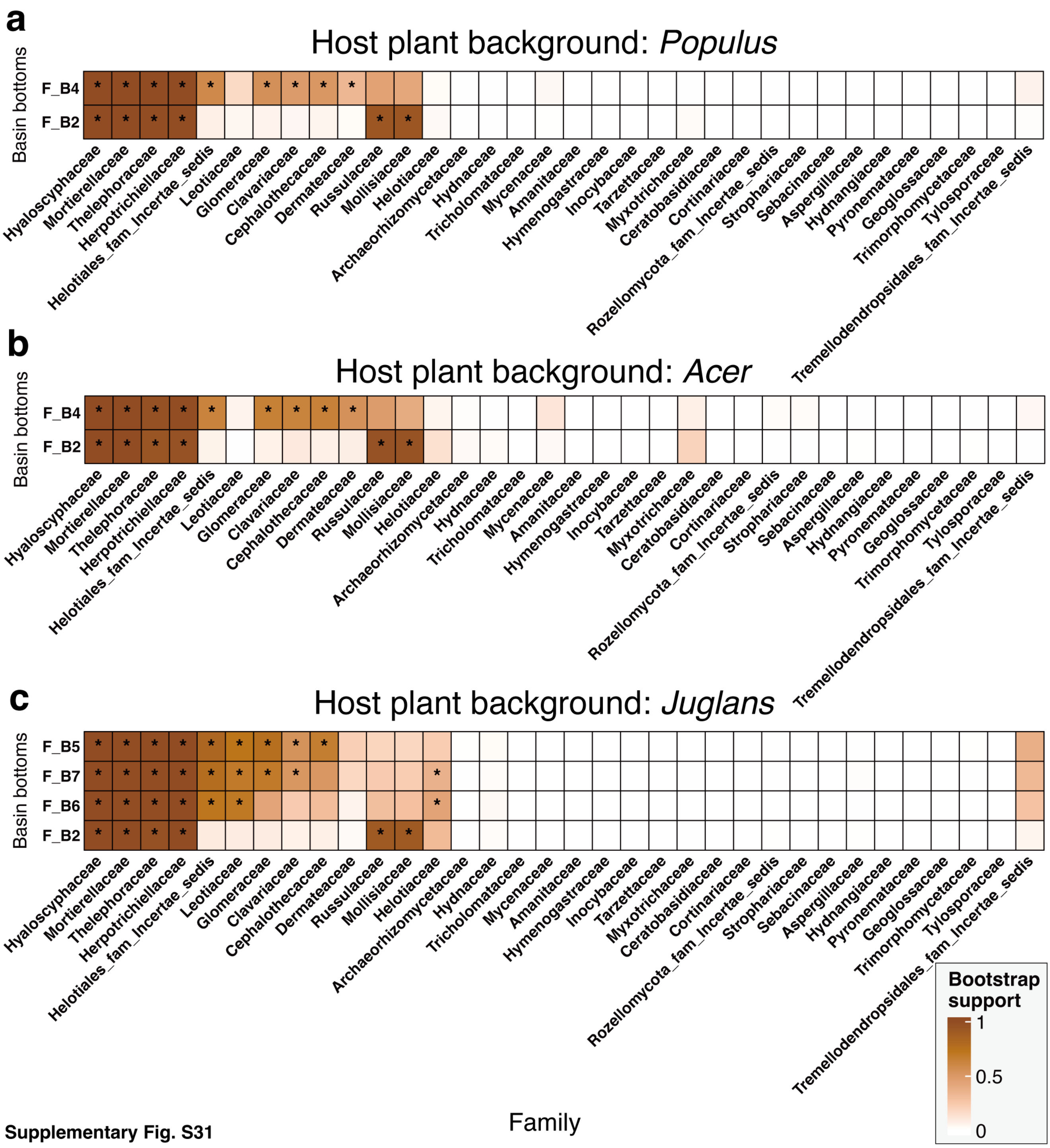

Supplementary Fig. S31 | Basin representation scores for family memberships of basin bottoms in fungal assembly landscapes under dual-mycorrhizal host backgrounds. To evaluate the robustness of taxonomic compositions defining basin bottoms, we performed bootstrap resampling of samples (1,000 replicates) followed by repeated energy landscape inference. Analyses focused on assembly landscapes inferred for dual-mycorrhizal host plants: *Populus* (**a**), *Acer* (**b**), and *Juglans* (**c**). Steepest-descent simulations were conducted using the same initial community states as those applied in the original analyses (Fig. 2c) to enable direct comparison among corresponding basins. Basin representation score for each family was quantified as the mean proportion of basin bottoms identified in bootstrap replicates that contained the focal family among states assigned to the corresponding basin in the original inference (Supplementary Fig. S28; see Supplementary Note 8 for details). The

families included in each basin in the original inference are indicated with asterisks. Source data are provided as a Source Data file.

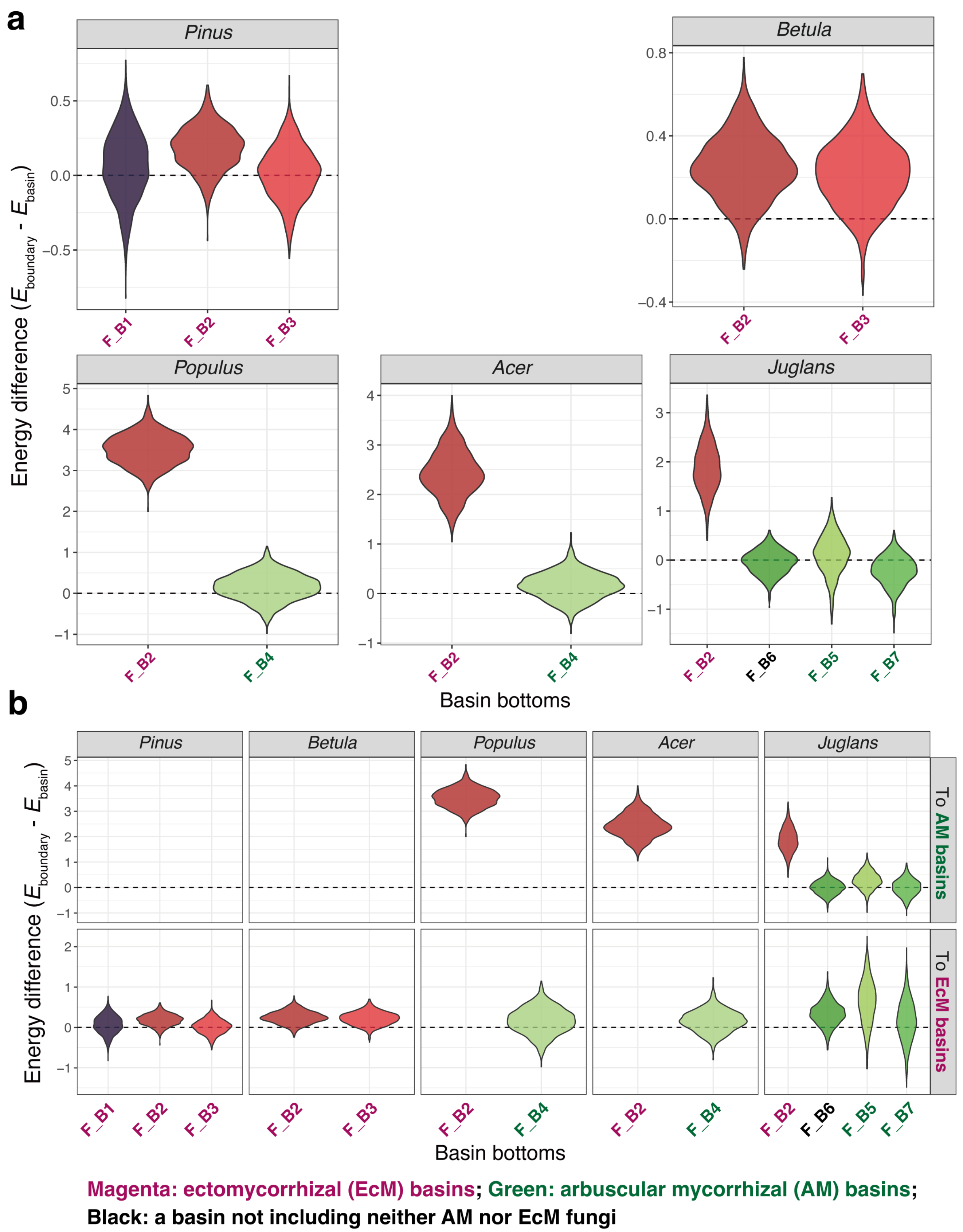

Supplementary Fig. S32

Supplementary Fig. S32 | Bootstrap analysis for evaluating uncertainty in the topography of fungal assembly landscapes. To evaluate the robustness of the relative ordering of basin bottoms and the corresponding boundary states to sampling variation, we performed bootstrap resampling of fungal community states (1,000 replicates) and repeated the energy landscape inference for each replicate

(Supplementary Fig. S29). (**a**) Energy difference between basin bottoms and the lowest-energy boundaries. For each bootstrap replicate, we evaluated energy differences between community states corresponding to basin bottoms and their corresponding lowest-energy boundary states defined in the original landscape. A positive value of this energy-difference index indicates that the relative ordering between basin-bottom states and boundary states was preserved across bootstrap landscapes. (**b**) Energy difference defined for transitions between arbuscular mycorrhizal and ectomycorrhizal basins. Arbuscular mycorrhizal basins (F_B4, F_B5, and F_B7) and ectomycorrhizal basins (F_B1– B3) were defined in Figure 2c. Source data are provided as a Source Data file.

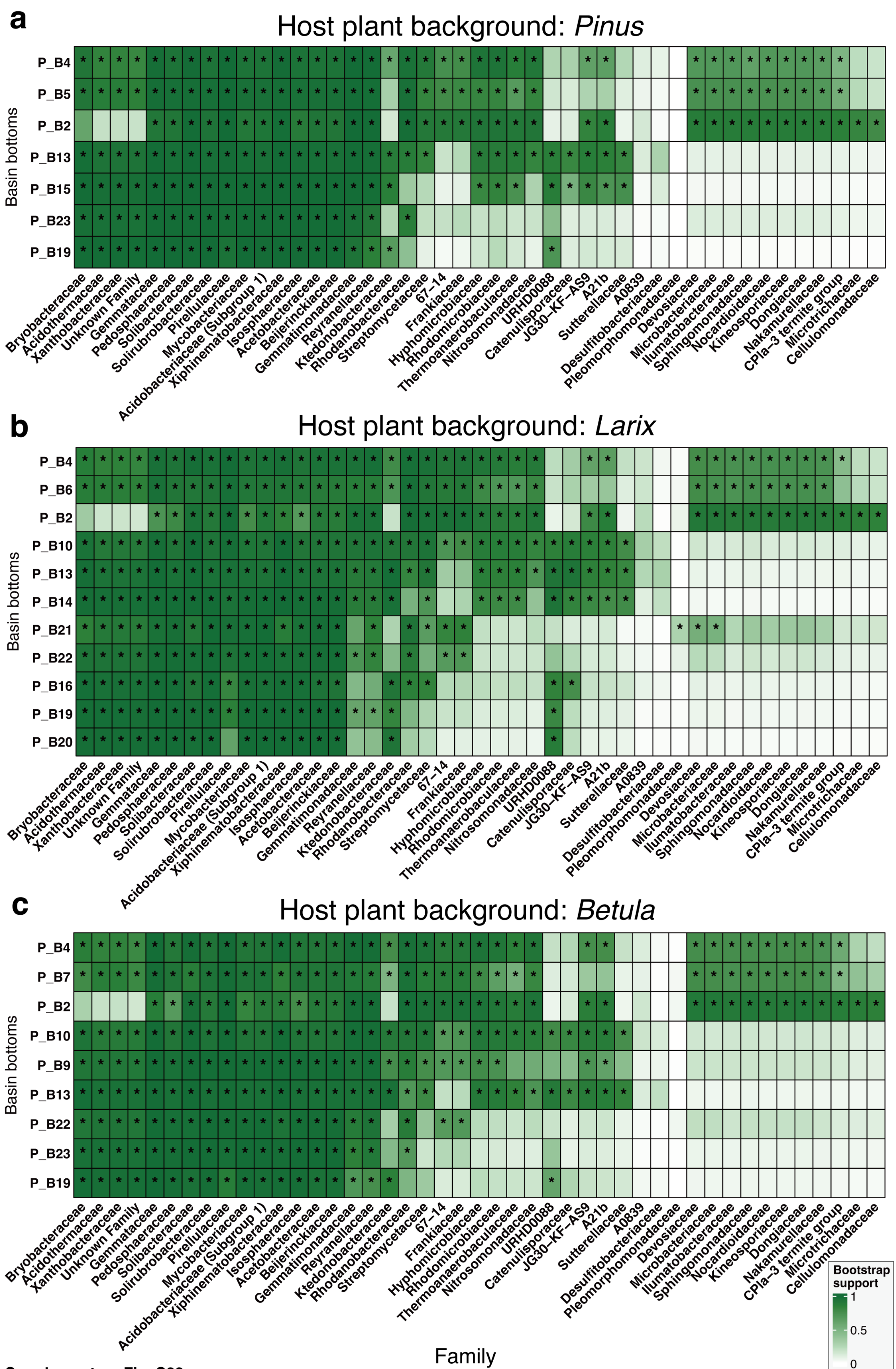

Supplementary Fig. S33

Supplementary Fig. S33 | Basin representation scores for family memberships of basin bottoms in prokaryotic assembly landscapes under ectomycorrhizal host backgrounds. To evaluate the robustness of basin-bottom community compositions to sampling variation, we performed bootstrap resampling of samples (1,000 replicates) followed by repeated energy landscape inference. For ectomycorrhizal host plants (*Pinus* (**a**), *Larix* (**b**), and *Betula* (**c**)), steepest-descent simulations were conducted using the same initial states as those applied in the original analyses (Fig. 3c). Basin representation score for each family was quantified as the mean proportion of basin bottoms identified in bootstrap replicates that contained the focal family among states assigned to the corresponding basin in the original inference (Supplementary Fig. S28; see Supplementary Note 8 for details). The families included in each basin in the original inference are indicated with asterisks. Source data are provided as a Source Data file.

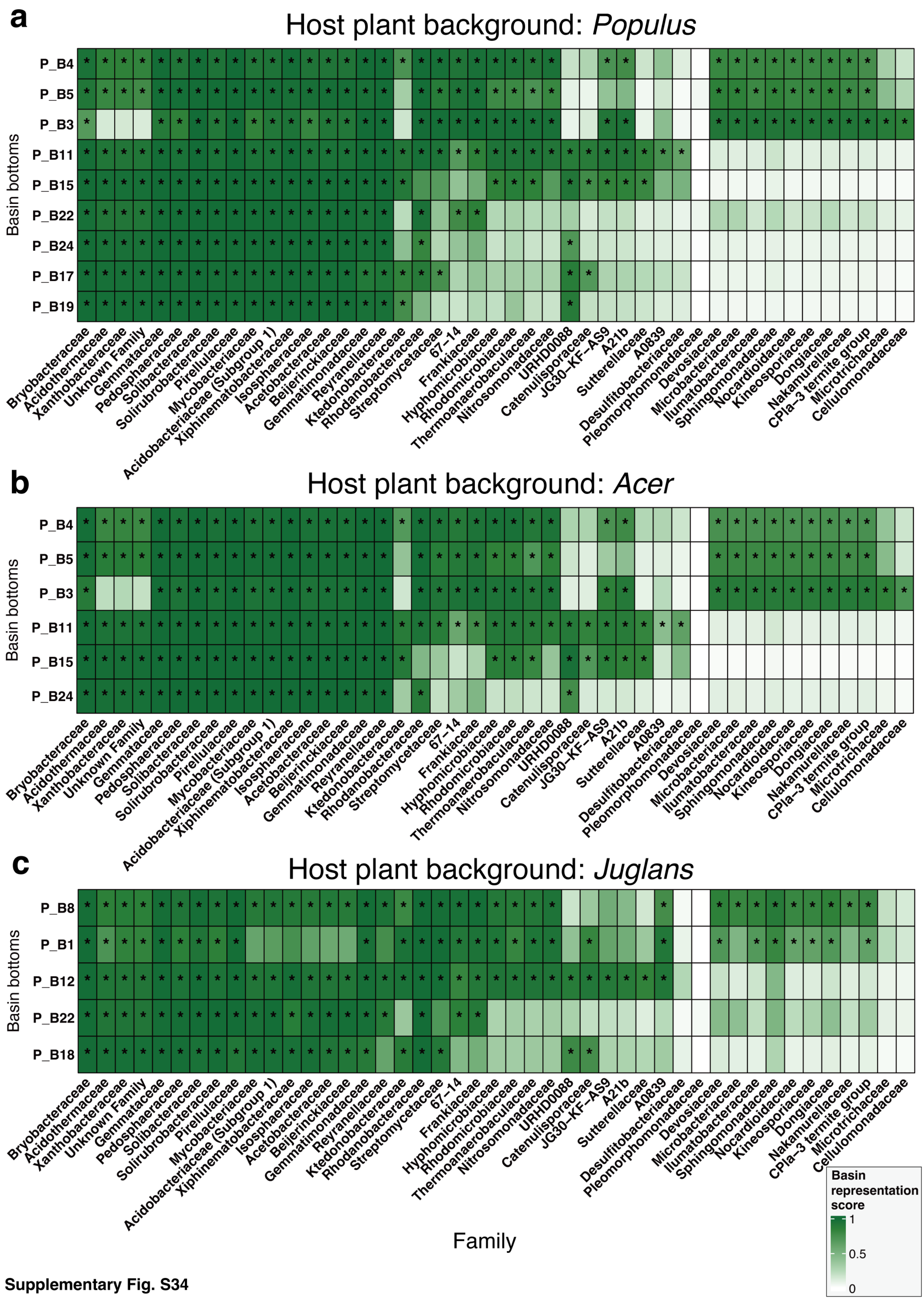

Supplementary Fig. S34

Supplementary Fig. S34 | Basin representation scores for family memberships of basin bottoms in prokaryotic assembly landscapes under dual-mycorrhizal host backgrounds. To evaluate the robustness of basin-bottom community compositions to sampling variation, we performed bootstrap resampling of samples (1,000 replicates) followed by repeated energy landscape inference. For dual-mycorrhizal host plants (*Populus* (**a**), *Acer* (**b**), and *Juglans* (**c**)), steepest-descent simulations were conducted using the same initial states as those applied in the original analyses (Fig. 3c). Basin representation score for each family was quantified as the mean proportion of basin bottoms identified in bootstrap replicates that contained the focal family among states assigned to the corresponding basin in the original inference (Supplementary Fig. S28; see Supplementary Note 8 for details). The families included in each basin in the original inference are indicated with asterisks. Source data are provided as a Source Data file.

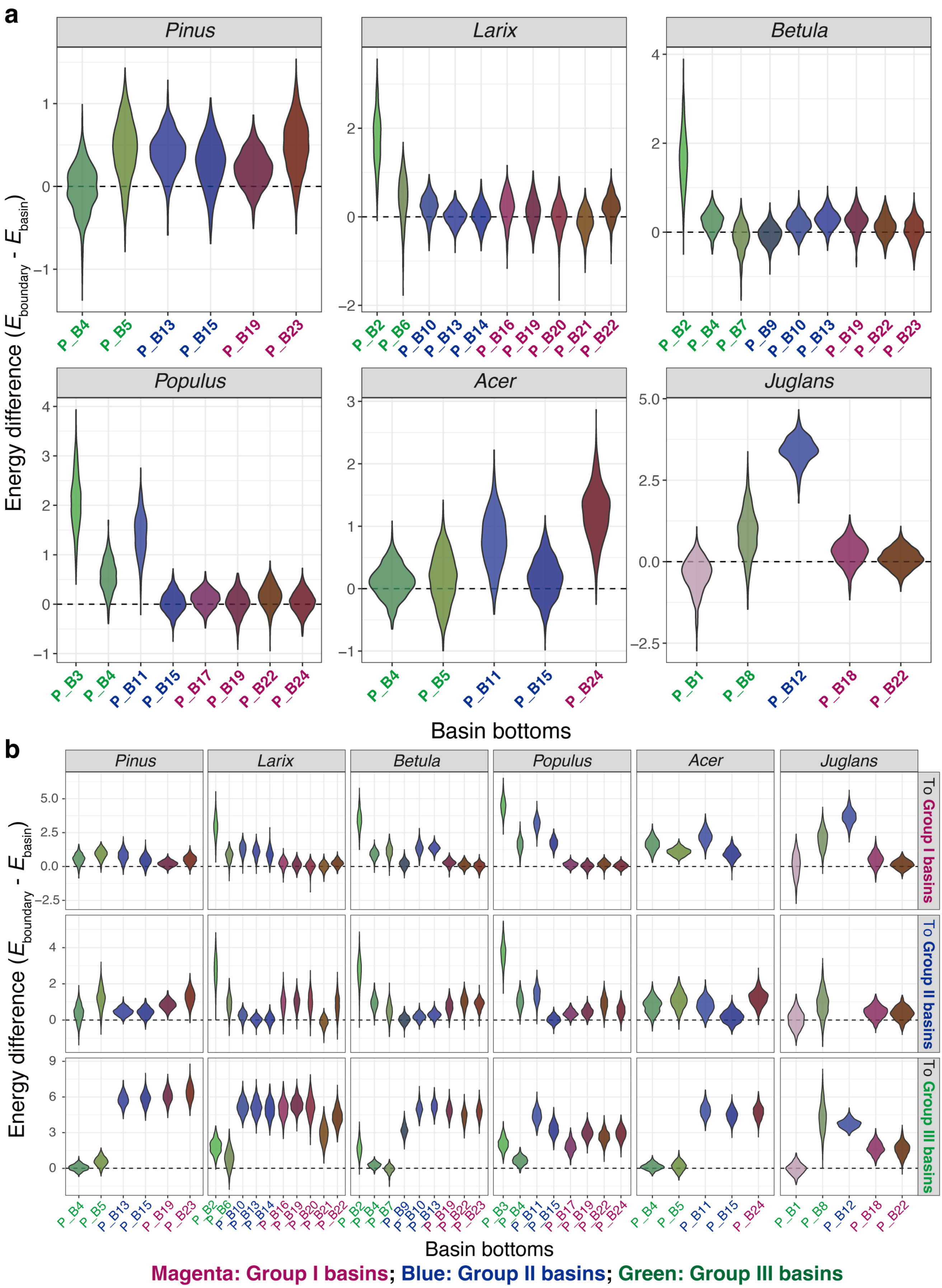

Supplementary Fig. S35 | Bootstrap analysis for evaluating uncertainty in the topography of prokaryotic assembly landscapes. To evaluate the robustness of the relative ordering of basin

bottoms and the corresponding boundary states to sampling variation, we performed bootstrap resampling of prokaryotic community states (1,000 replicates) and repeated the energy landscape inference for each replicate (Supplementary Fig. S29). (**a**) Energy difference between basin bottoms and the lowest-energy boundaries. For each bootstrap replicate, we evaluated energy differences between community states corresponding to basin bottoms and their corresponding lowest-energy boundary states defined in the original landscape. A positive value of this energy-difference index indicates that the relative ordering between basin-bottom states and boundary states was preserved across bootstrap landscapes. (**b**) Energy difference defined for transitions among basin groups. The basins groups of prokaryotic communities (Group I basins: P_B16– B24; Group II basins: P_B9– B15; Group III basins: P_B1– B8) were defined in Figure 3c. Source data are provided as a Source Data file.

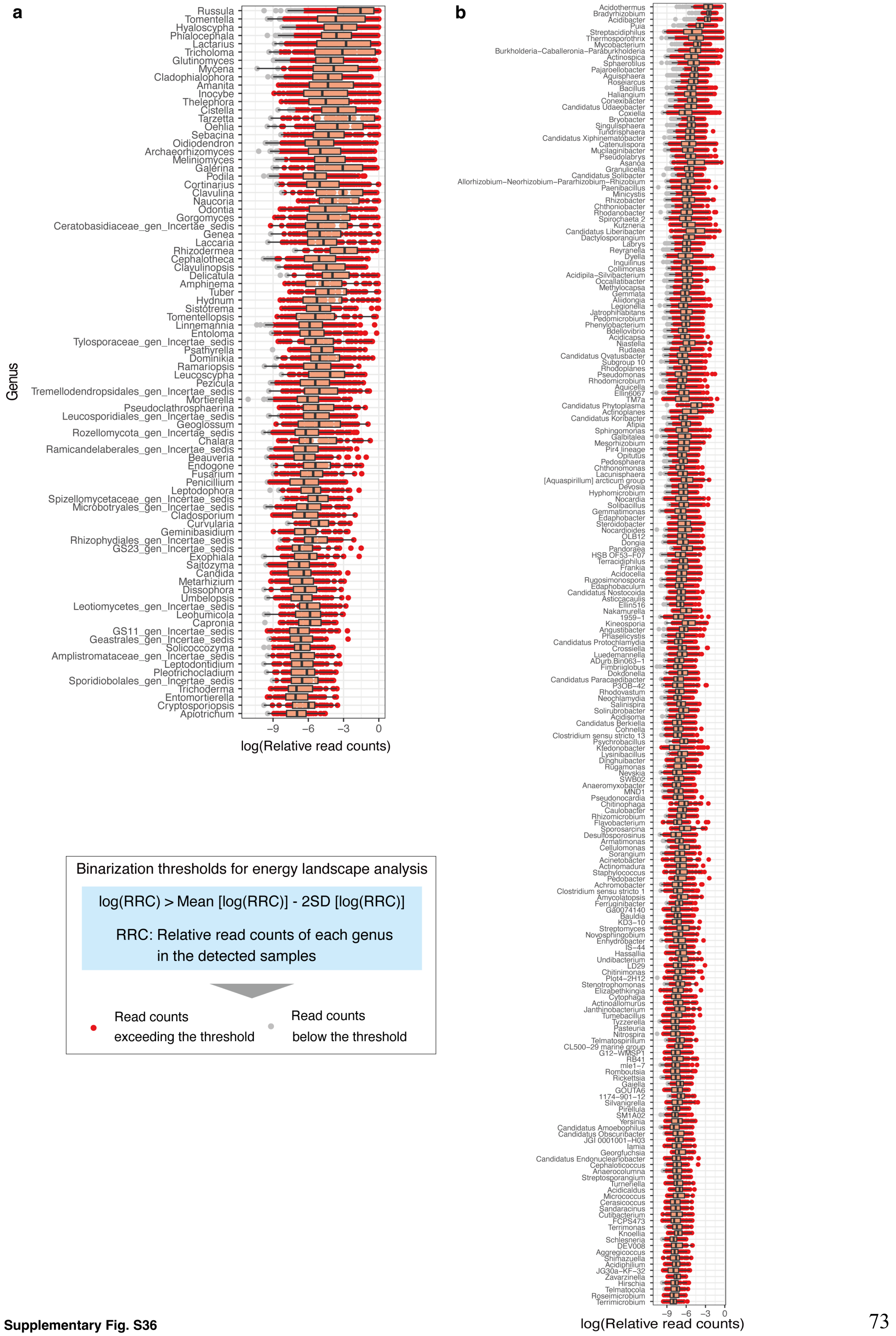

Supplementary Fig. S36

Supplementary Fig. S36 | Relative abundances of microbial genera | (**a**) Distribution of relative read counts of each fungal genus in samples in which it was detected. Each boxplot shows the distribution of relative read counts (RRC) of the focal genus across these samples. The centre lines indicate the median (50% quantile), the lower and upper bounds of the boxes indicate the 25% and 75% quantiles, respectively, and the whiskers extend to the smallest and largest observations within 1.5 × the interquartile range (IQR) from the lower and upper quartiles, respectively. To remove potential sequencing contaminants, the threshold for binarization was defined in the same way as binarization of families (Supplementary Figs. S11–S12). Briefly, the threshold for each genus was defined as Mean [log (RRC)] – 2SD [log (RRC)], where Mean [log (RRC)] and SD [log (RRC)] denote the mean and standard deviation of the log-transformed relative read counts of the focal genus, respectively. (**b**) Distribution of relative read counts for each prokaryotic genus. Source data are provided as a Source Data file.

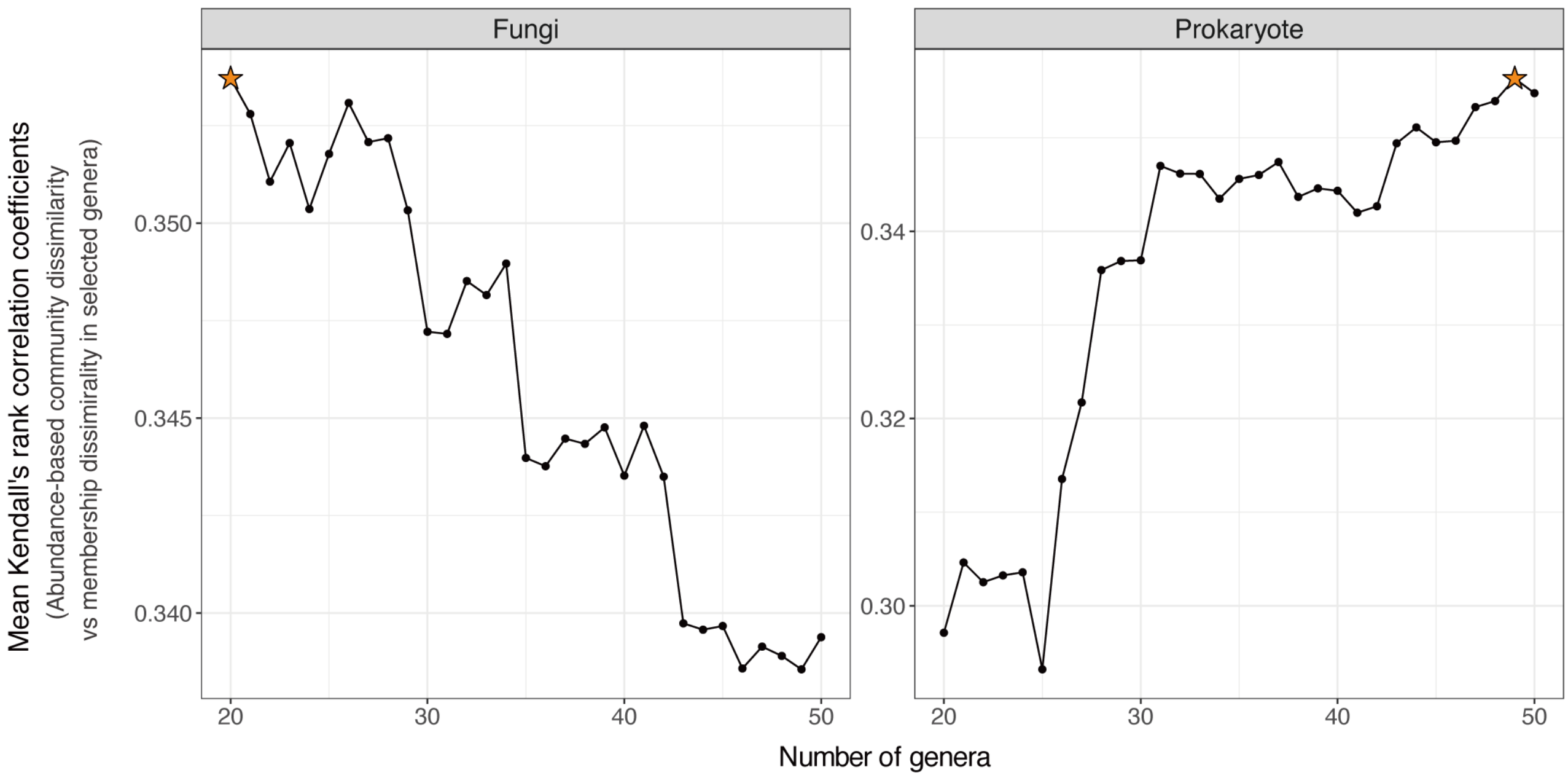

Supplementary Fig. S37 | Selecting optimal genus sets for energy landscape analysis. The optimal sets (numbers) of fungal and prokaryotic genera were determined in the same way as selecting optimal families (Supplementary Figs. S11– S13). Specifically, to make the exploration of optimal fungal and prokaryotic genus sets computationally feasible, we first ordered 50 genera based on their explanatory power for the overall community structure (R^2 in the PerMANOVA). We then evaluated the correlations between community dissimilarity values calculated from the binarized data of each candidate genus set (Jaccard dissimilarity) and those calculated from the original relative abundance data (Bray–Curtis dissimilarity). The selected fungal and prokaryotic genus sets are indicated by star-shaped symbols (See Supplementary Tables S12–S13). Source data are provided as a Source Data file.

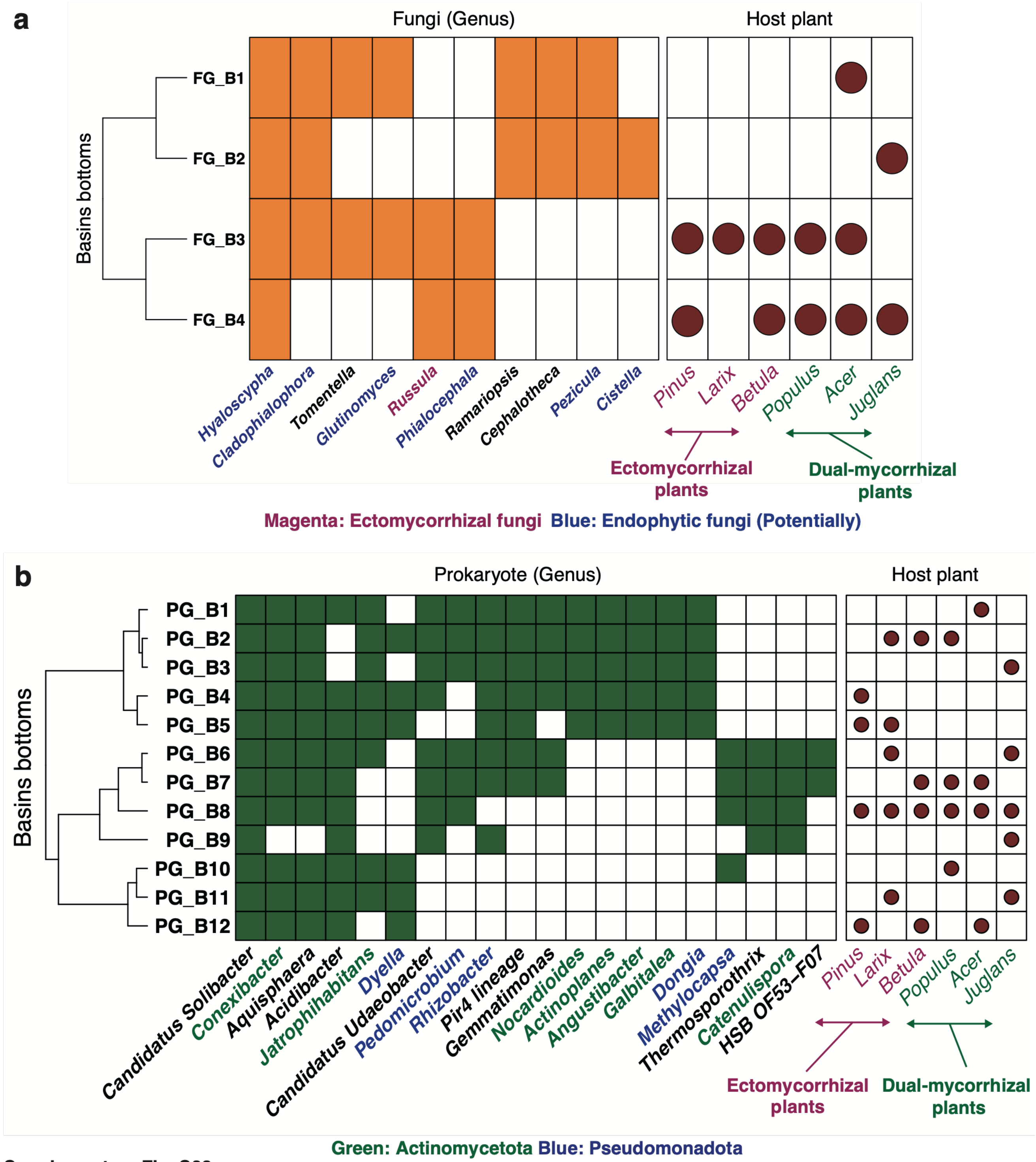

Supplementary Fig. S38

Supplementary Fig. S38 | Community states of the basin bottoms in genus-level energy landscape analysis. (**a**) Genus memberships of the basin bottoms in fungal assembly landscapes. The four bottoms inferred across the six host plant backgrounds are shown with a dendrogram representing taxonomic membership similarity. While arbuscular mycorrhizal fungi were not included in community states for this genus-level analysis (Supplementary Table S12), arbuscular mycorrhizal and ectomycorrhizal basins are tentatively defined depending on ectomycorrhizal genus *Russula* (family: Russulaceae) and saprotrophic genus *Ramariopsis* (family: Clavariaceae) detected in arbuscular mycorrhizal basins in family-level analysis (Fig. 2c). The repertoires of basin bottoms

detected in each host-plant background are shown in the right panel. (**b**) Genus memberships in prokaryotic assembly landscapes. While the basins corresponding to Group II in family-level analysis are unclear because genus-level annotation was difficult for several families within Pseudomonadota (e.g. Sutterellaceae and A21b), Group I basins (PG_B10–B12), composed of commonly detected taxa such as *Candidatus* Solibacter (family: Solibacteraceae) and *Conexibacter* (family: Solirubrobacteraceae), were clearly identified, as were Group III basins (PG_B1–B5) that additionally included Actinomycetota taxa (e.g., *Nocardioides* [family: Nocardioidaceae] and *Actinoplanes* [family: Micromonosporaceae]). Source data are provided as a Source Data file.

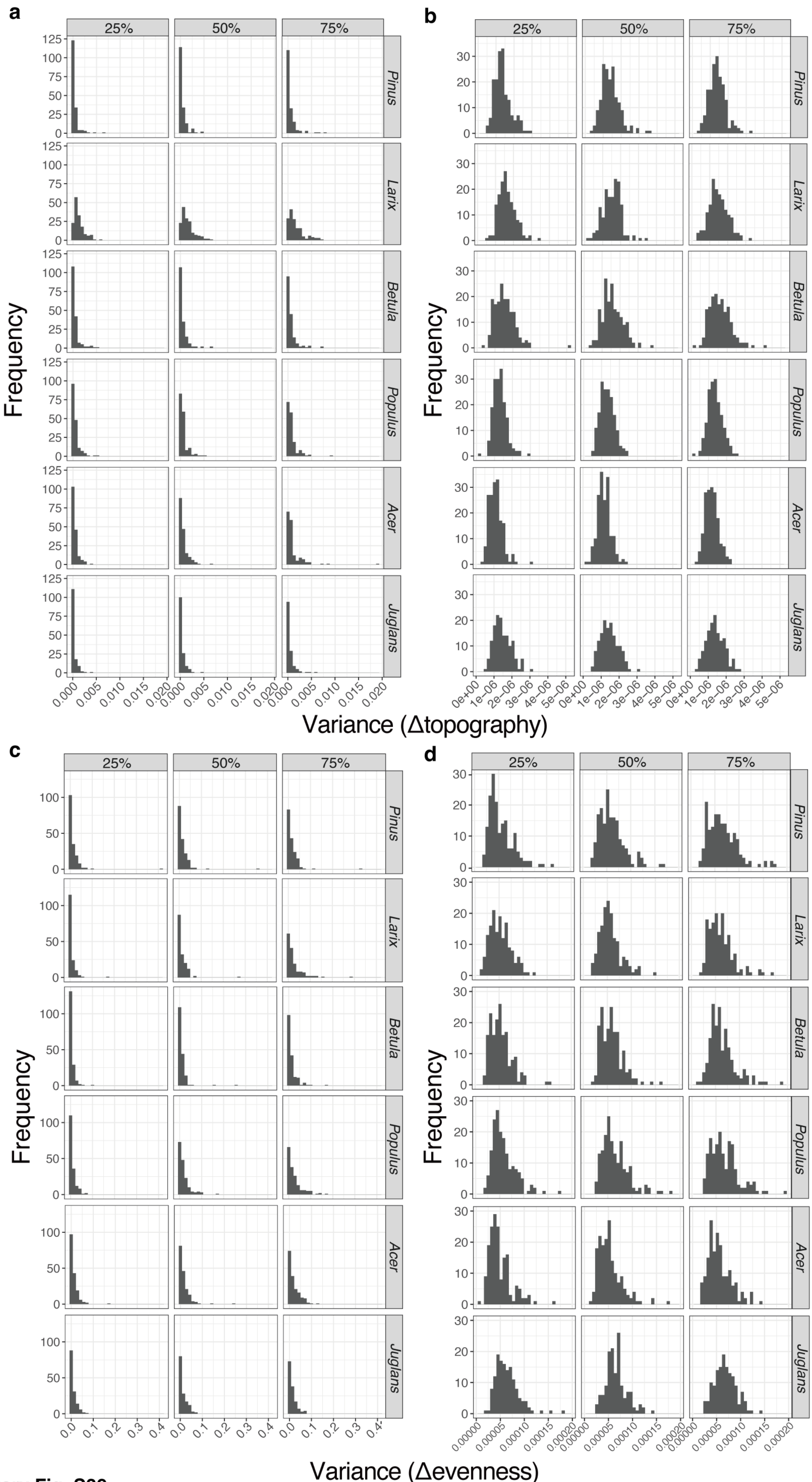

Supplementary Fig. S39

Supplementary Fig. S39 | Histograms of the estimation variances in keystoneness metrics. The

Δtopography and Δevenness values of each genus analyzed in Figure 4 were recalculated 30 times using different sets of 20,000 randomly initialized community states. Calculations were performed for each of the six host plants (rows), assuming different abundance quantile levels (columns; 25%, 50%, and 75% quantiles of relative read counts in samples in which the focal microbial taxon was present). (**a**) Δtopography for the fungal assembly landscapes. (**b**) Δtopography for the prokaryotic assembly landscapes. (**c**) Δevenness for the fungal assembly landscapes. (**d**) Δevenness for the prokaryotic assembly landscapes. Source data are provided as a Source Data file.

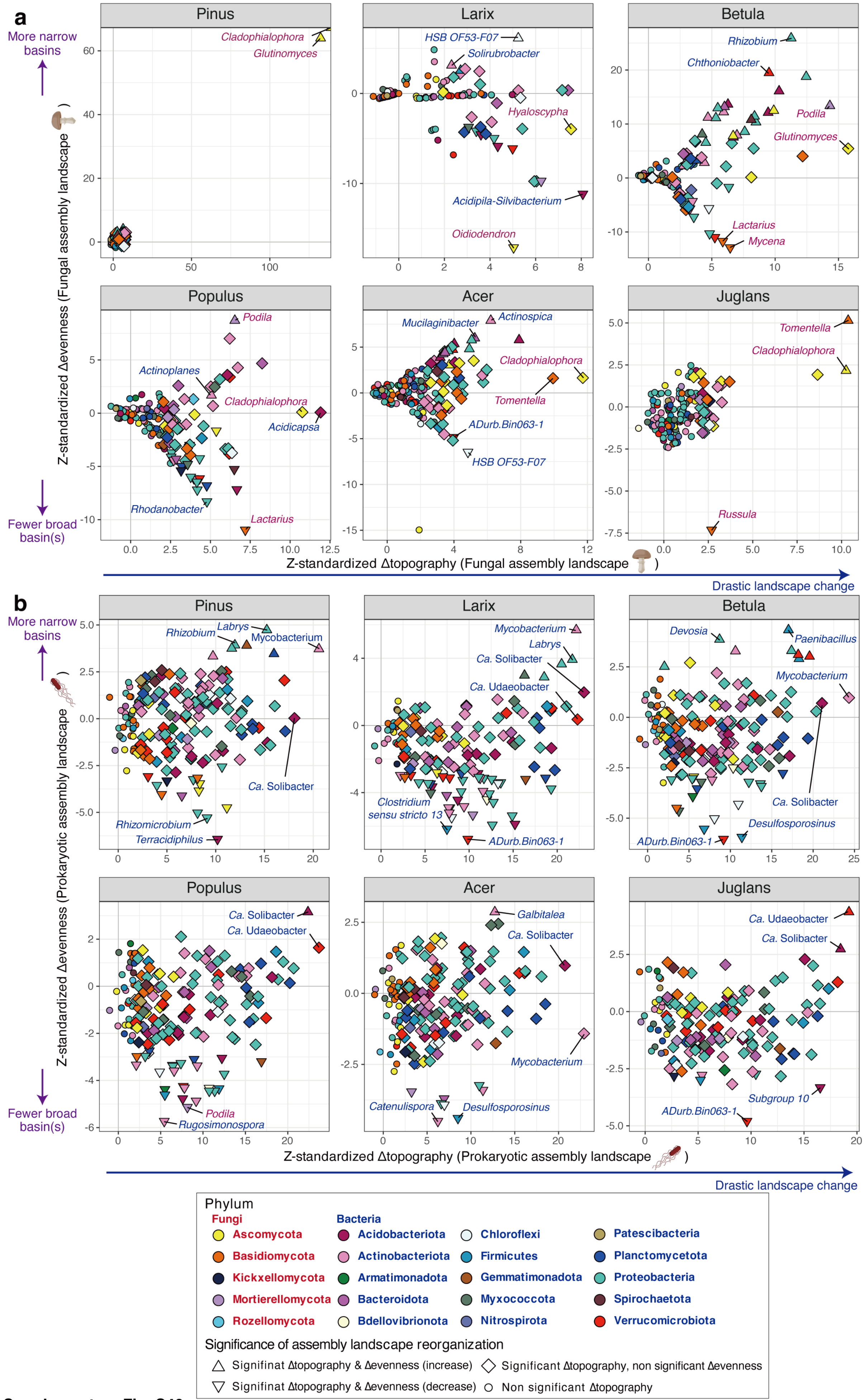

Supplementary Fig. S40

Supplementary Fig. S40 | Potential impacts on the fungal and prokaryotic community inferred in genus-level assembly landscapes. (**a**) Taxa strongly associated with the reorganizations of genus-level fungal assembly landscapes along their abundance gradients from 0% (absence) to 50% (median) abundance quantile. On two-dimensional planes defined by Δtopography and Δevenness, microbial genera whose abundance changes were inferred to substantially reshape the landscape architecture of root-associated fungal communities are highlighted. (**b**) Taxa strongly associated with the reorganizations of genus-level prokaryotic assembly landscapes. Source data are provided as a Source Data file. Fungal illustrations (a): Created in BioRender. Toju, H. (2026) https://BioRender.com/2rdd0th. Bacterial illustrations (b): Created in BioRender. Toju, H. (2026) https://BioRender.com/w6b4bi9.

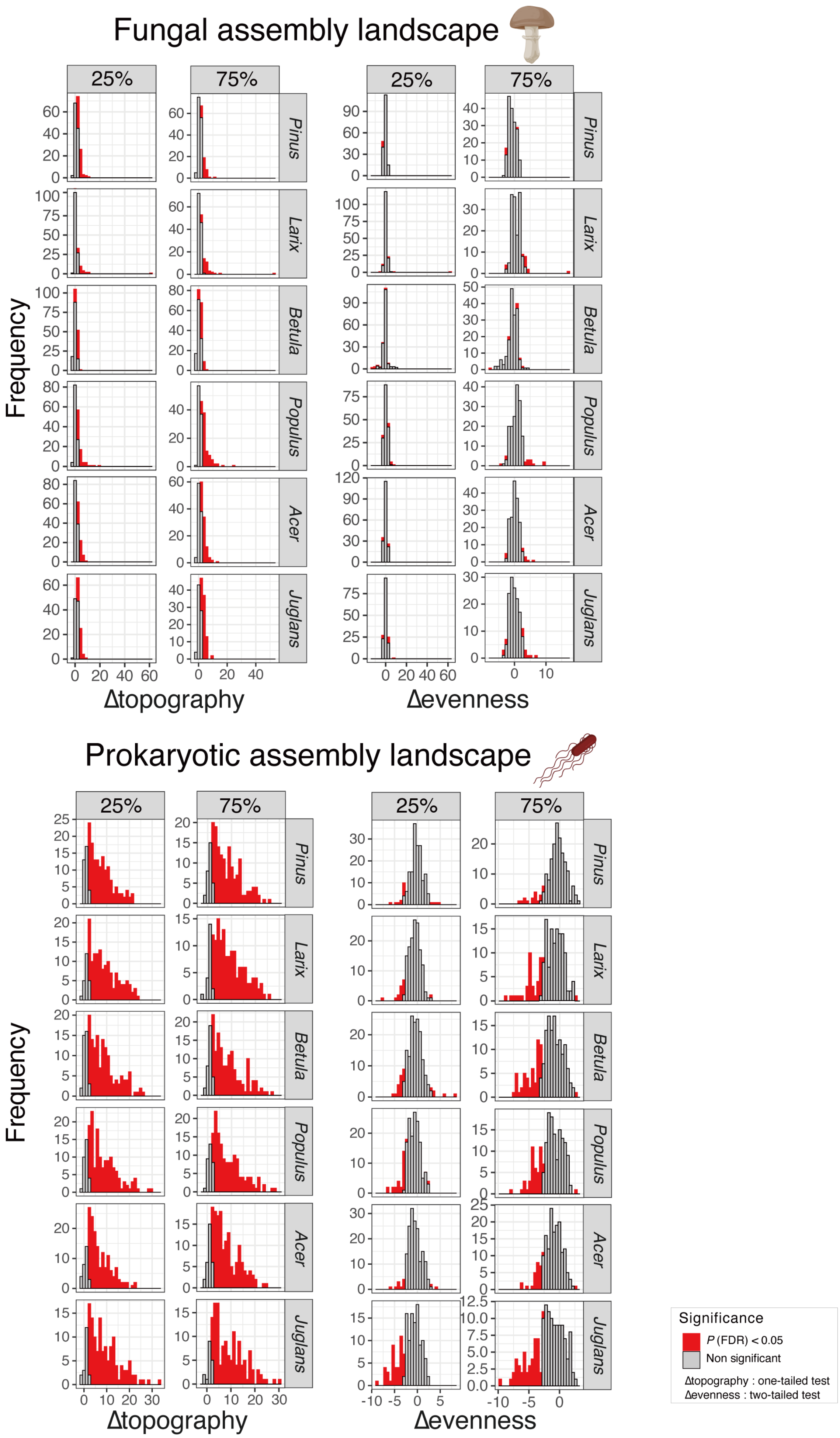

Supplementary Fig. S41

Supplementary Fig. S41 | Sensitivity of keystoneness metrics to alternative abundance quantile assumptions. To evaluate whether inferred keystoneness depends on the choice of abundance conditions used in the comparison, we recalculated keystoneness metrics using alternative presence conditions defined by the 25% and 75% quantiles of the relative abundance of each microbial genus. Histograms show the distributions of *z*-standardized Δtopography and Δevenness values, which quantify changes in the overall landscape topography and the evenness of basin-frequency distributions, respectively, along abundance gradients of focal genera (see Fig. 1d–g). *P* values were adjusted using the Benjamini–Hochberg procedure. Genera that had significant Δtopography or Δevenness values are shown in red (one-sided test for Δtopography; two-sided test for Δevenness). Results are presented separately for each host-plant background for fungal and prokaryotic assembly landscapes. Source data are provided as a Source Data file. Fungal illustration: Created in BioRender. Toju, H. (2026) https://BioRender.com/2rdd0th. Bacterial illustration: Created in BioRender. Toju, H. (2026) https://BioRender.com/w6b4bi9.

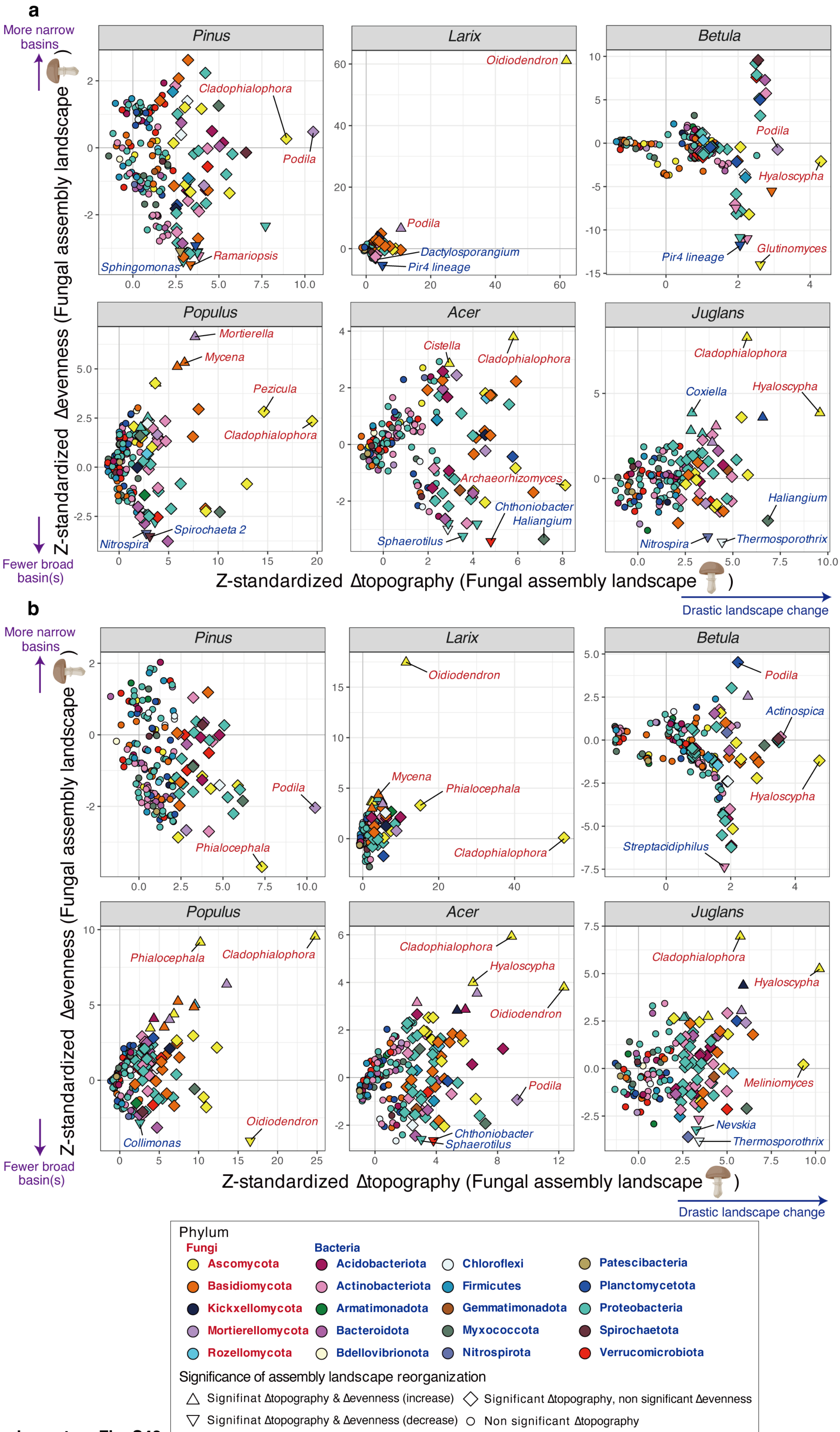

Supplementary Fig. S42

Supplementary Fig. S42 | Sensitivity of inferred keystone impacts to alternative abundance-gradient definitions in fungal assembly landscapes. To examine how inferred community-scale impacts depend on abundance assumptions, we evaluated potential keystone effects along alternative abundance gradients of each microbial genus. Genera whose abundance changes were inferred to substantially reorganize fungal assembly landscapes are shown on two-dimensional planes defined by z-standardized Δtopography and Δevenness, which represent changes in landscape topography and basin-frequency evenness, respectively. (**a**) Results based on abundance gradients defined between absence (0%) and the 25% abundance quantile. (**b**) Results based on abundance gradients defined between absence (0%) and the 75% abundance quantile. Source data are provided as a Source Data file. Fungal illustrations: Created in BioRender. Toju, H. (2026) https://BioRender.com/2rdd0th.

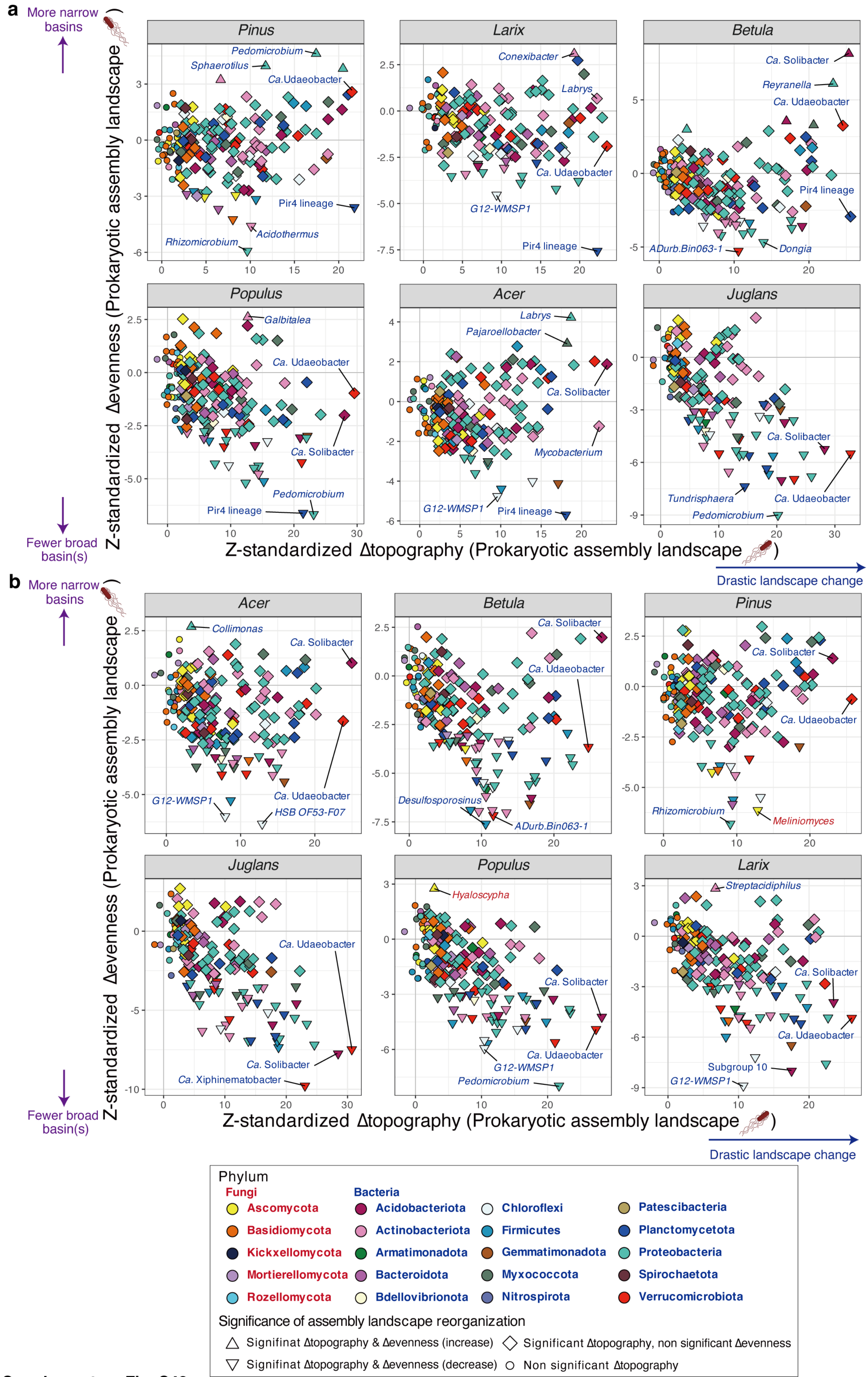

Supplementary Fig. S43

Supplementary Fig. S43 | Sensitivity of inferred keystone impacts to alternative abundance-gradient definitions in prokaryotic assembly landscapes. To examine how inferred community-scale impacts depend on abundance assumptions, we evaluated potential keystone effects along alternative abundance gradients of each microbial genus. Genera whose abundance changes were inferred to substantially reorganize prokaryotic assembly landscapes are shown on two-dimensional planes defined by z-standardized Δtopography and Δevenness, which represent changes in landscape topography and basin-frequency evenness, respectively. (**a**) Results based on abundance gradients defined between absence (0%) and the 25% abundance quantile. (**b**) Results based on abundance gradients defined between absence (0%) and the 75% abundance quantile. Source data are provided as a Source Data file. Bacterial illustrations: Created in BioRender. Toju, H. (2026) https://BioRender.com/w6b4bi9.

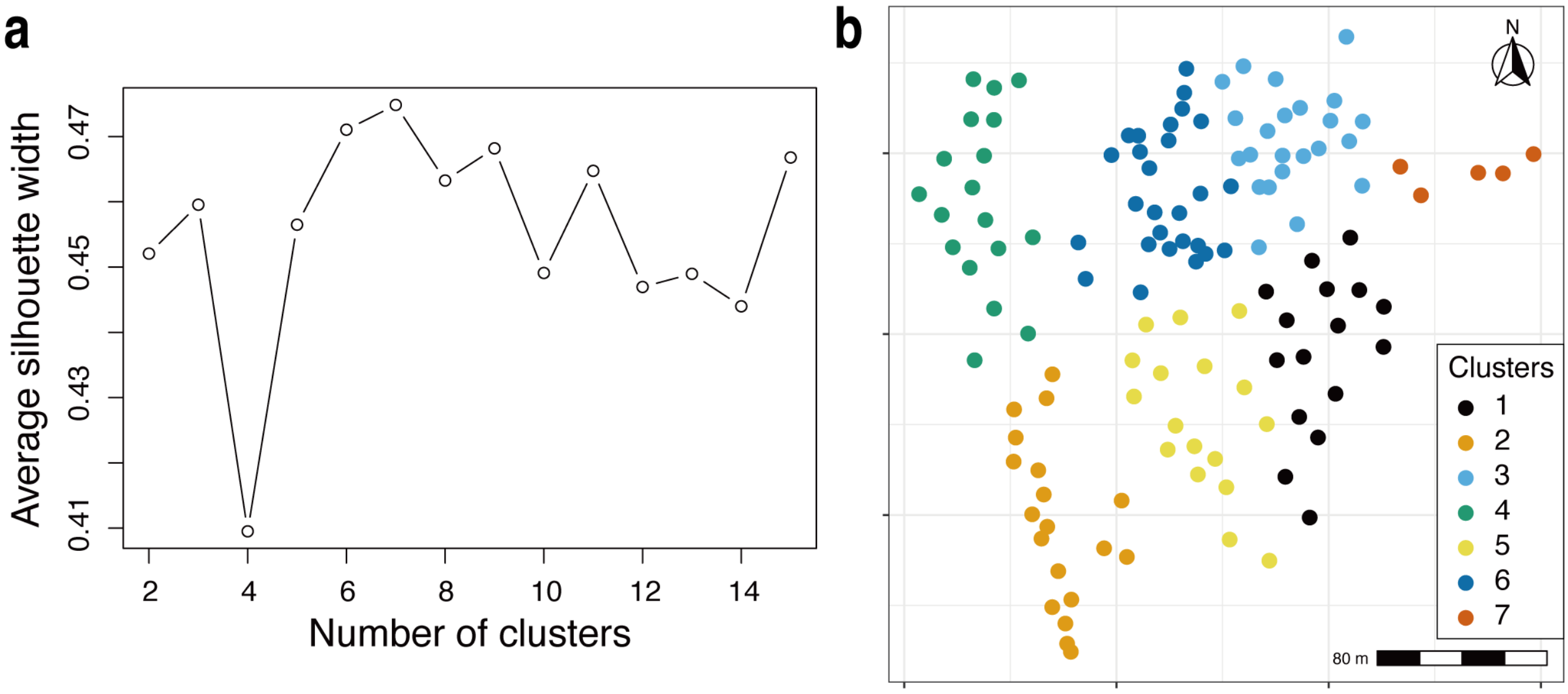

Supplementary Fig. S44 | Definition of spatial blocks used for constrained randomization analyses. To account for spatial structure in permutation-based sensitivity analyses, sampling locations were grouped into spatial blocks using *k*-means clustering. (**a**) Determination of the optimal number of spatial blocks. Clustering was performed sequentially with cluster numbers ranging from *k* = 2 to 15, and the optimal number of clusters was identified using the silhouette method. (**b**) Spatial configuration of sampling blocks. Based on the optimal clustering result, the 125 sampling points in the study forest were classified into seven spatial blocks. Source data are provided as a Source Data file.

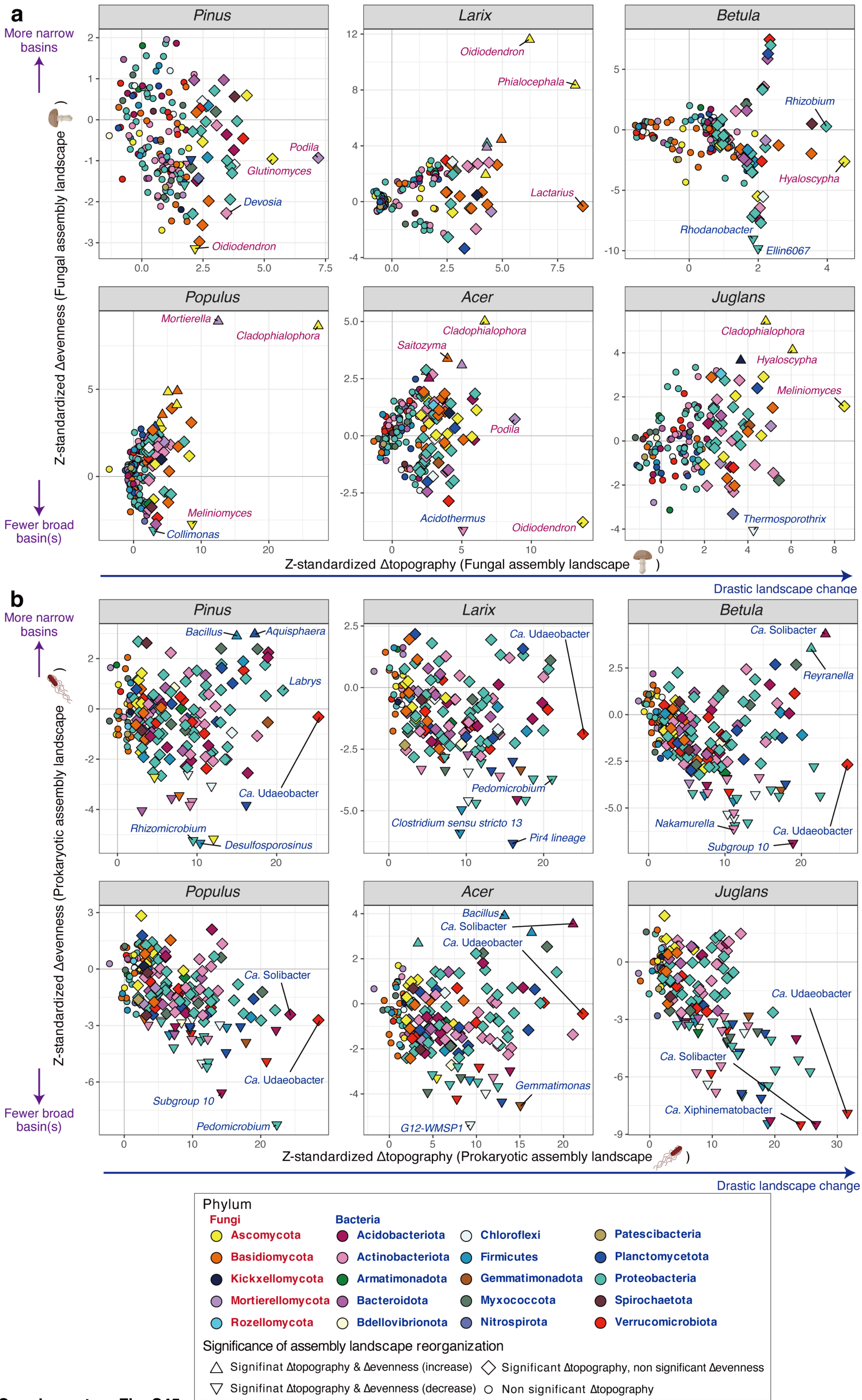

Supplementary Fig. S45

Supplementary Fig. S45 | Sensitivity of inferred keystone impacts to spatially constrained permutation tests. To evaluate whether spatial structure influences the estimation of keystoneness metrics (Δtopography and Δevenness), permutation tests were performed with randomization constrained by combinations of host identity and spatial sampling blocks (Supplementary Fig. S44; see Supplementary Note 12 for details). Genera whose abundance changes were inferred to substantially reorganize assembly landscapes are shown on two-dimensional planes defined by z-standardized Δtopography and Δevenness. (**a**) Results for fungal assembly landscapes based on abundance gradients defined between absence (0%) and the 50% abundance quantile (median). (**b**) Results for prokaryotic assembly landscapes under the same permutation constraints. Source data are provided as a Source Data file. Fungal illustrations (a): Created in BioRender. Toju, H. (2026) https://BioRender.com/2rdd0th. Bacterial illustrations (b): Created in BioRender. Toju, H. (2026) https://BioRender.com/w6b4bi9.

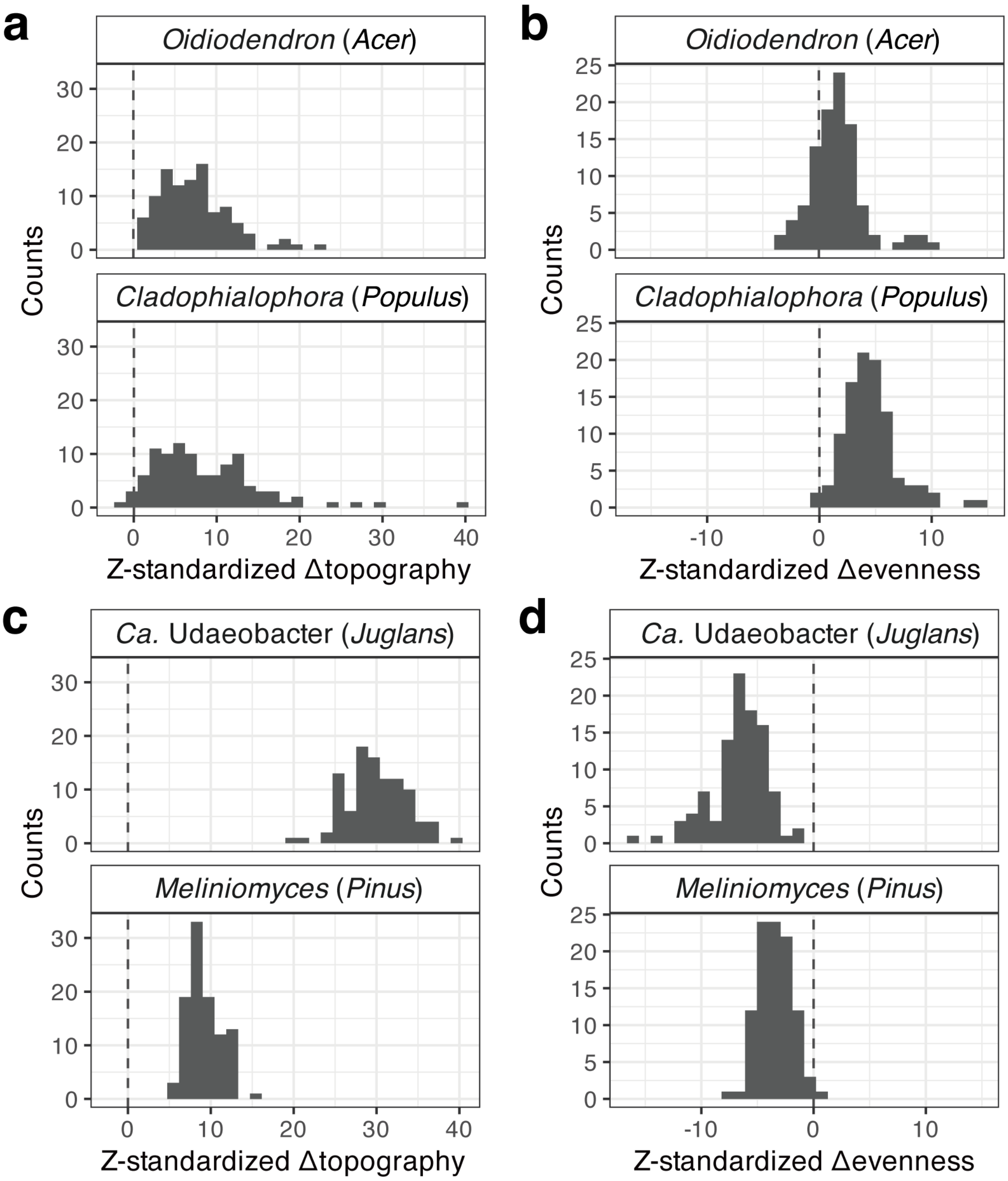

Supplementary Fig. S46 | Bootstrap uncertainty of inferred keystoneness metrics for representative taxa. To quantify uncertainty in the keystoneness metrics (Δtopography and Δevenness), we performed bootstrap resampling of sample-level abundance data for focal genera and recalculated the metrics across 100 bootstrap replicates. Because this procedure is computationally intensive, analyses were restricted to representative genus–host plant combinations highlighted in Figures 5 and 6. Distributions of z-standardized Δtopography and Δevenness are shown for fungal assembly landscapes of *Oidiodendron* (host plant background: *Acer*) and *Cladophialophora* (host plant background: *Populus*) (**a**,**b**), and for prokaryotic assembly landscapes of *Candidatus* Udaeobacter (host plant background: *Juglans*) and *Meliniomyces* (host plant background: *Pinus*) (**c**,**d**). Source data are provided as a Source Data file.

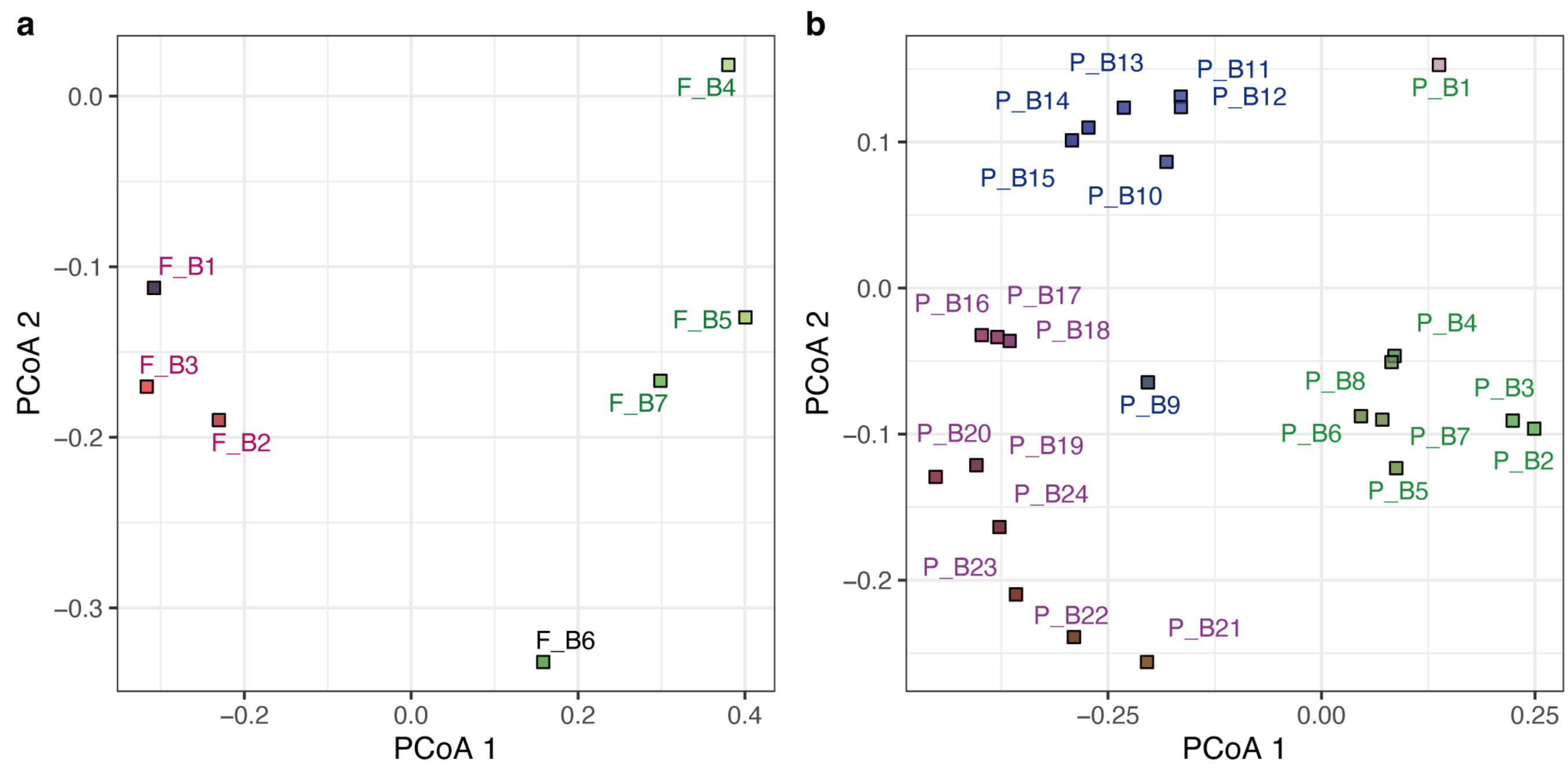

Supplementary Fig. S47 | Ordination of basin-bottom community compositions. (**a**) Principal coordinate analysis (PCoA) of fungal basin-bottom community states shown in Figure 2c. Dissimilarities among basin bottoms were calculated using Jaccard dissimilarity. Colors correspond to those used in Figure 2c and Supplementary Figure S48. (**b**) PCoA of prokaryotic basin-bottom community states shown in Figure 3c. Colors correspond to those used in Figure 3c and Supplementary Figure S49. Source data are provided as a Source Data file.

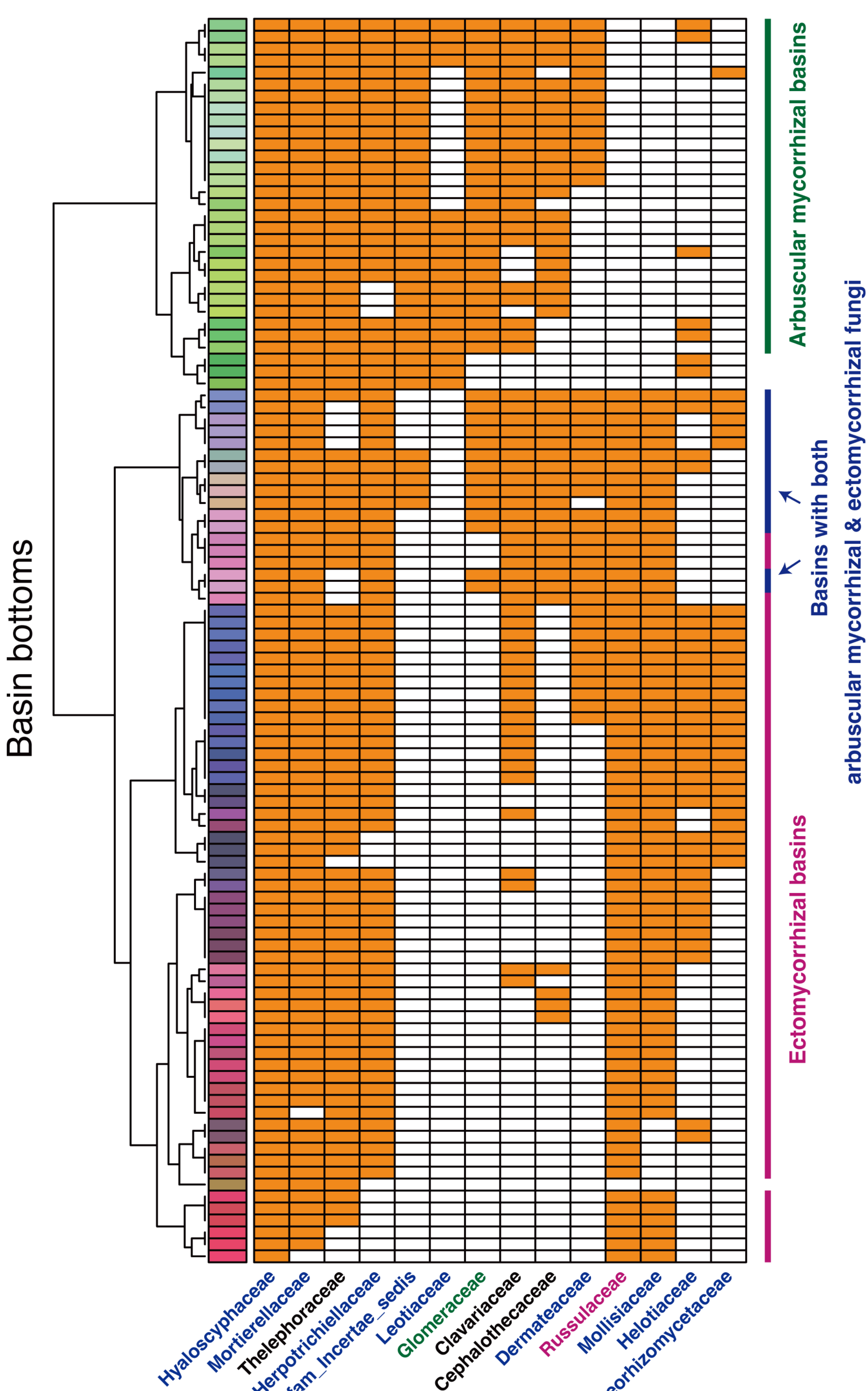

Supplementary Fig. S48

Supplementary Fig. S48 | Community states at basin bottoms of fungal energy landscapes. Basin-bottom community states inferred across six host-plant backgrounds (Figs 2c and 5; Supplementary Figs. S21–S22) are shown together with a dendrogram representing taxonomic membership similarity. The color gradient in the left panel indicates compositional similarity among community states and corresponds to the relationships illustrated in the flow diagrams (Fig. 5; Supplementary Figs. S21–S22). Based on the presence or absence of *Glomeraceae* and *Russulaceae*, basins are tentatively classified as arbuscular mycorrhizal or ectomycorrhizal types. In addition to the basins shown in Figure 2c, several basins containing both *Glomeraceae* and *Russulaceae* were detected. Fungal families are colored according to functional guilds: green, arbuscular mycorrhizal; magenta, ectomycorrhizal; blue, potentially endophytic; black, families with multiple or other functions. Source data are provided as a Source Data file.

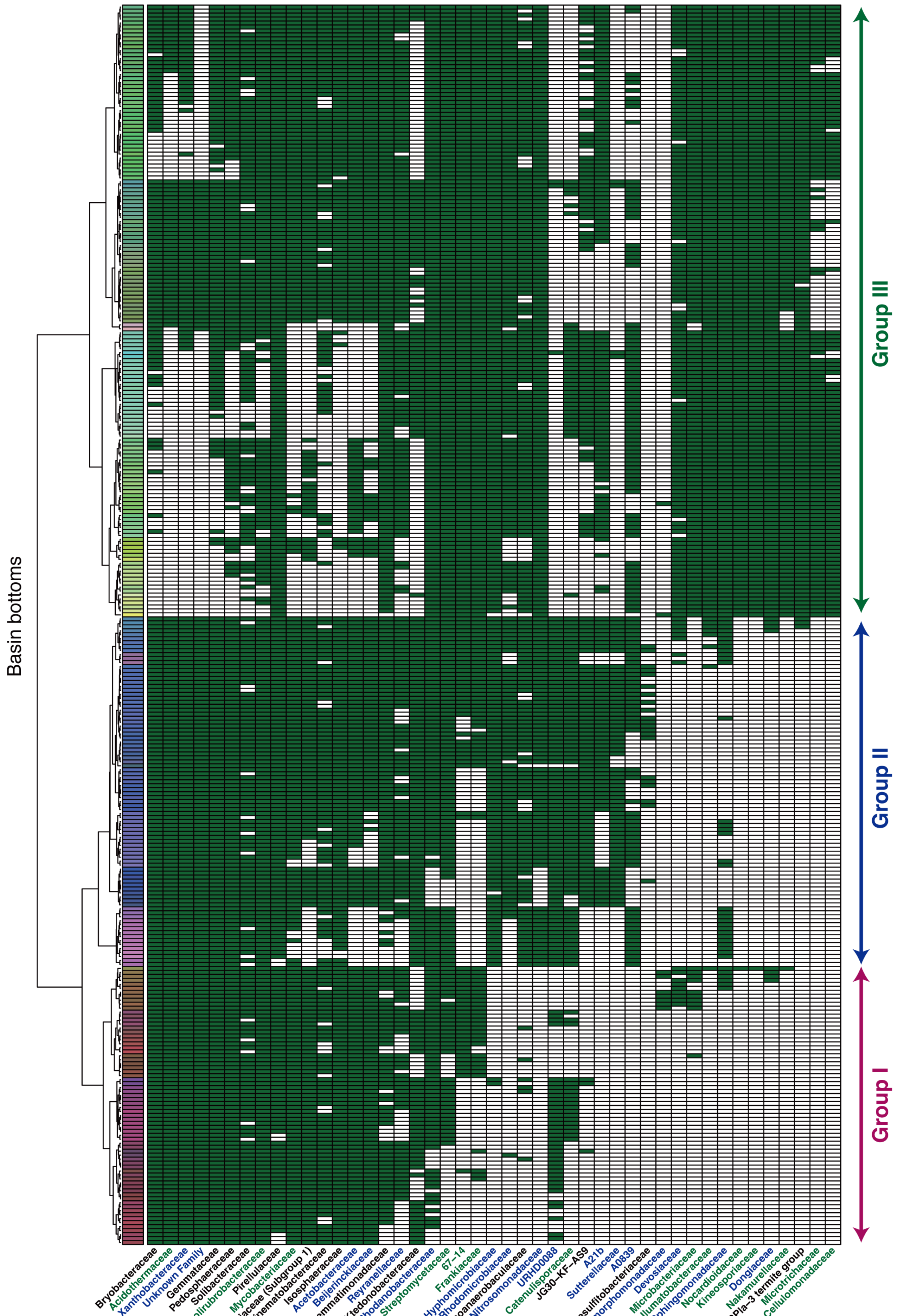

Supplementary Fig. S49

Supplementary Fig. S49 | Community states at basin bottoms of prokaryotic energy landscapes. Basin-bottom community states inferred across six host-plant backgrounds (Figs 3c and 6; Supplementary Figs. S23–S24) are shown together with a dendrogram representing taxonomic membership similarity. The color gradient in the left panel indicates compositional similarity among community states and corresponds to the relationships illustrated in the flow diagrams (Fig. 6; Supplementary Figs. S23–S24). Basins are tentatively classified into three categories: Group I, consisting primarily of commonly observed families; Group II, characterized by the presence of additional Pseudomonadota families; and Group III, characterized by the presence of additional Actinomycetota families. Prokaryotic families are colored according to phylum-level taxonomy: blue, Pseudomonadota; green, Actinomycetota; black, other phyla. Source data are provided as a Source Data file.

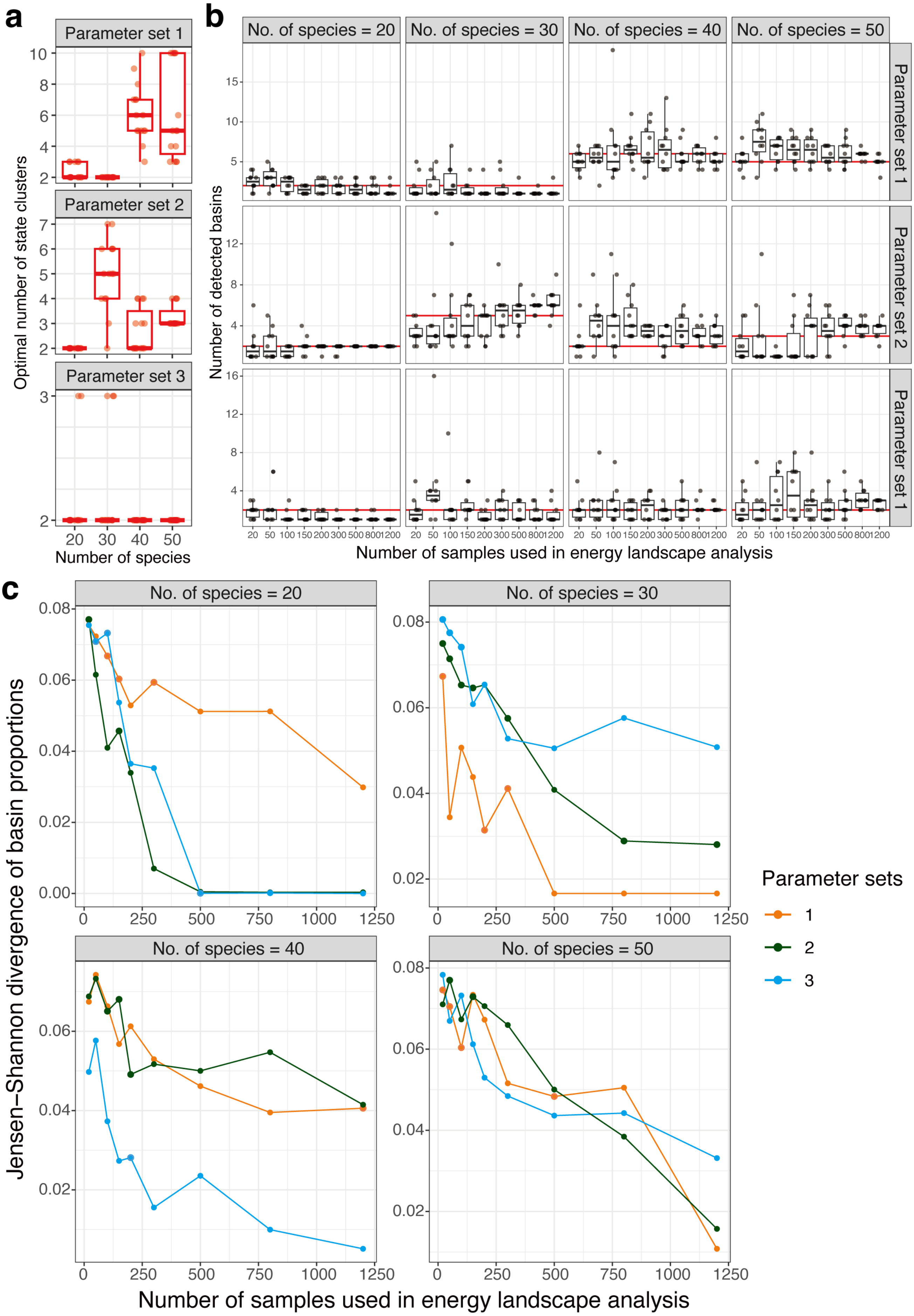

Supplementary Fig. S50

Supplementary Fig. S50 | Influences of sample size and species richness on assembly landscape reconstruction in generalized Lotka–Volterra simulations. Benchmark datasets were generated using generalized Lotka–Volterra (gLV) models to evaluate how species richness and sample size influence assembly landscape inference. Community dynamics were simulated for systems comprising 20, 30, 40, or 50 species, with three independently generated parameter sets for each richness level. For each parameter set, we estimated the number of discrete terminal community states that could arise from randomly initialized community compositions. Specifically, 100 simulations were run from independently generated initial abundance vectors until $t = 100$, and terminal community compositions were defined as the mean abundance vectors over $t = 95 - 100$. These terminal community compositions were binarized using thresholds selected to maximize the correspondence between Bray–Curtis (abundance data) and Jaccard (binarized data) dissimilarities. Binarized terminal community states were then subjected to hierarchical clustering based on Jaccard dissimilarity to determine the optimal number of clusters by varying the number of clusters from 2 to 10 (Ward's method and silhouette method). This procedure was repeated 15 times for each parameter set to account for uncertainty in cluster-number estimation. Using the same parameter sets, benchmark datasets containing 20–1,200 community compositions were generated by running stochastic gLV simulations from different initial species abundances until $t = 100$ and randomly sampling one time point from each simulation. Energy landscape analysis was then performed on binarized datasets, and basin bottoms were identified using a steepest-descent procedure initiated from 150,000 randomly generated community states. (**a**) Estimated optimal numbers of clusters for terminal community states. Boxplot centre lines indicate medians; boxes, the 25%–75% quantiles; and whiskers, the smallest and largest observations within 1.5 × the interquartile range (IQR). (**b**) Numbers of basins detected on inferred assembly landscapes for each combination of sample size and species richness. Red lines indicate the median cluster number estimated for each parameter set in (**a**). (**c**) Variability among replicate landscape inferences, each corresponding to independent gLV simulations with different initial conditions, quantified using Jensen–Shannon divergence of basin-frequency distributions. Source data are provided as a Source Data file.

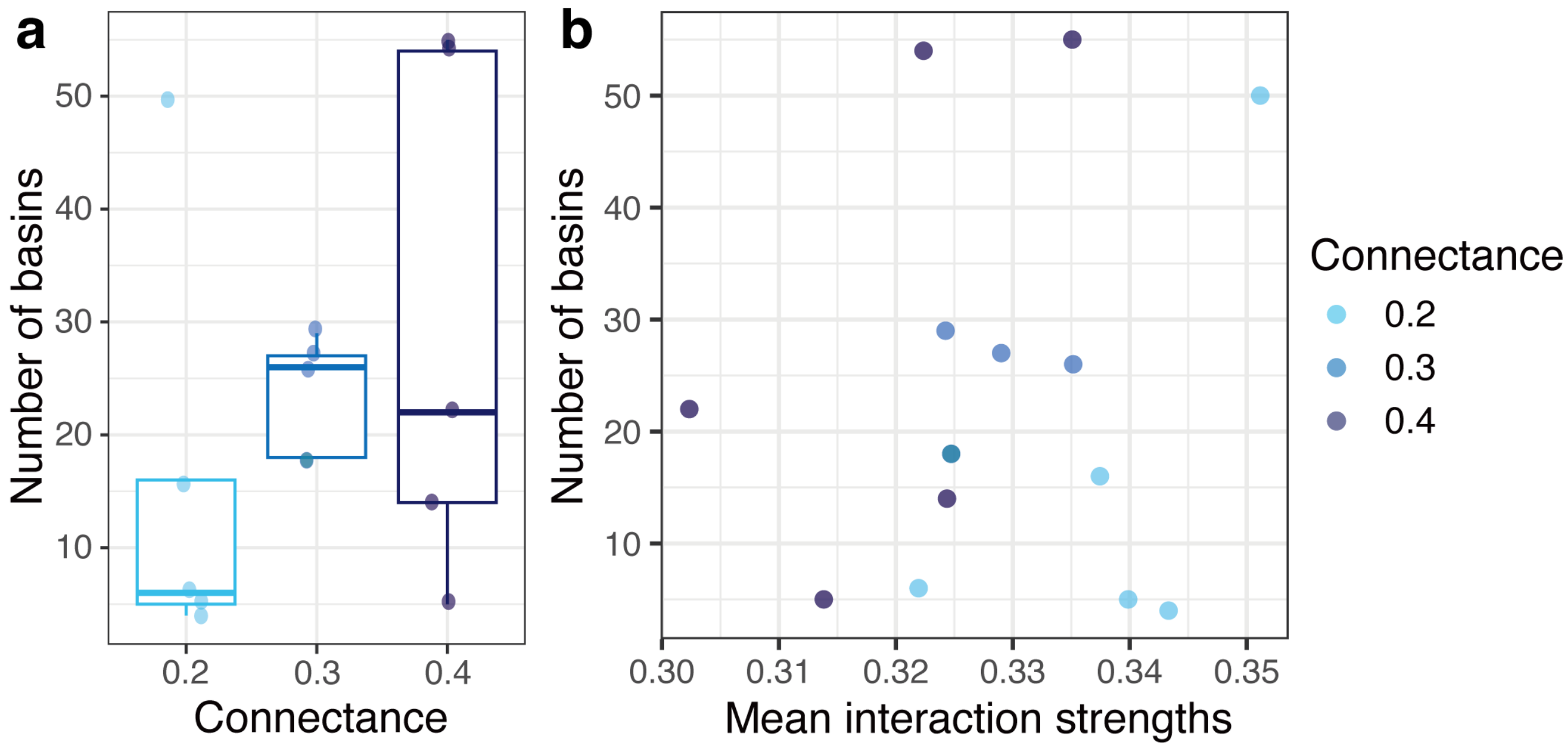

Supplementary Fig. S51 | Relationships between interaction-network properties and basin structure in reconstructed assembly landscapes. To examine whether global properties of the gLV interaction network were associated with the number of basins inferred by energy landscape analysis, we analyzed the 15 binarized community datasets generated from the gLV parameter sets used in the benchmark analyses shown in Supplementary Figures S6–S8. For each dataset, we reconstructed the energy landscape and estimated the number of basins using a steepest-descent procedure initiated from 150,000 randomly generated community states. We then examined the relationships between the number of inferred basins and two global properties of the corresponding interaction matrix: connectance (the proportion of nonzero off-diagonal interaction coefficients; Kruskal-Wallis test: n = 15 independent datasets, χ^2(degree of freedom = 2) = 2.77, P = 0.250) and mean interaction strength (the mean absolute value of nonzero off-diagonal interaction coefficients; Kendall's rank correlation coefficient: $\tau = -9.66 \times 10^{-3}$, P = 0.960). (**a**) Relationship between connectance and the number of inferred basins. Boxplot centre lines indicate medians; boxes, the 25%–75% quantiles; and whiskers, the smallest and largest observations within 1.5 × the interquartile range (IQR). (**b**) Relationship between mean interaction strength and the number of inferred basins. Source data are provided as a Source Data file.

Supplementary Table S1 | Correspondences between observed community structures and inferred basin identity. PerMANOVA and PERMDISP were used to evaluate whether basin identities inferred from the assembly landscape framework corresponded to observed family-level community structures. Fungal and prokaryotic communities were analyzed as abundance-based compositions, based on family-level relative abundance profiles, and as presence/absence-based community states, based on binarized occurrence patterns of the selected families used in the assembly landscape analysis. Bray–Curtis and Jaccard dissimilarities were used for the abundance-based compositions and presence/absence-based states, respectively. PerMANOVA tested differences in community structure among inferred basin identities (10,000 permutations), whereas PERMDISP tested differences in multivariate dispersion among basin identities. The table lists degrees of freedom, R^2 values, and FDR-adjusted P values from PERMANOVA, as well as F values and FDR-adjusted P values from PERMDISP. P values were adjusted using the Benjamini–Hochberg procedure. The exact P values are provided in Source Data file.

			PerMANOVA		PERMDISP	
	Community structure	Degree of freedom	*R2*	*P* (FDR)	*F* value	*P* (FDR)
Fungi	Abundance	6	3.74×10^{-2}	< 0.001	14.4	< 0.001
Fungi	Presence/Absence	6	7.15×10^{-2}	< 0.001	30.9	< 0.001
Prokaryotes	Abundance	24	1.72×10^{-1}	< 0.001	8.85	< 0.001
Prokaryotes	Presence/Absence	24	2.80×10^{-1}	< 0.001	21.5	< 0.001

Supplementary Table S2 | Genera that exhibited the greatest potential impacts on the fungal assembly landscape along their abundance gradients. The Δtopography and Δevenness indices respectively represented changes in the overall topography and evenness of energy landscape architecture along the abundance gradients of a focal genus (from absence to their median abundance condition; Fig. 1d–g). The *z*-standardized metrics of Δtopography and Δevenness are obtained based on the randomization analysis in which the abundance of a focal genus was shuffled within the root samples of the same host plant (10,000 iterations). For each host plant background, the two fungal/prokaryotic genera with the highest and significant Δtopography are listed. *P* values of Δtopography (one-sided test) and Δevenness (two-sided test) were separately adjusted using the Benjamini–Hochberg procedure. The exact *P* values are provided in Source Data file.

Genus	Host plant	Z-standardized Δtopography	*P* (FDR) [Δtopography]	Z-standardized Δevenness	*P* (FDR) [Δevenness]
Hyaloscypha	*Betula*	4.33	< **0.001**	-1.63	0.170
Spirochaeta 2	*Betula*	3.47	< **0.001**	0.05	0.517
Podila	*Pinus*	1.03 ×10	< **0.001**	-0.19	0.482
Rhizobium	*Pinus*	6.57	< **0.001**	-2.01	0.063
Oidiodendron	*Acer*	1.93 ×10	< **0.001**	-4.84	0.030
Podila	*Acer*	9.42	< **0.001**	1.13	0.252
Cladophialophora	*Populus*	2.50 ×10	< **0.001**	8.63	**0.002**
Pezicula	*Populus*	1.36 ×10	< **0.001**	2.56	0.129
Oidiodendron	*Larix*	1.18 ×10	**0.001**	2.38 ×10	**0.005**
Archaeorhizomyces	*Larix*	7.94	< **0.001**	0.06	0.703
Hyaloscypha	*Juglans*	8.43	< **0.001**	3.45	**0.002**
Meliniomyces	*Juglans*	8.16	< **0.001**	0.97	0.290

Supplementary Table S3 | Relationships between Δtopography and abundance or occupancy of microbial genera. Correlations between genus-level abundance (log-transformed relative read counts) or occupancy and z-standardized Δtopography values were evaluated to examine whether microbial genera with large effects on assembly landscape topography were simply explained by their dominance in the root microbiome dataset. For each host plant and each reconstructed assembly landscape, Kendall's rank correlation tests were performed between the z-standardized Δtopography values of microbial genera and their abundance or occupancy. The table lists Kendall's rank correlation coefficients (Kendall's τ), and false discovery rate-adjusted P values. P values were adjusted using the Benjamini–Hochberg procedure. The exact P values are provided in a Source Data file.

		Abundance (log (relative read counts))				Occupancy	
		Fungal genera		Prokaryotic genera		Fungal & Prokaryotic genera	
Landscapes	Host plant	Kendall's τ	P (FDR)	Kendall's τ	P (FDR)	Kendall's τ	P (FDR)
Fungal landscape	*Acer*	-1.14×10^{-2}	0.939	2.34×10^{-1}	< 0.001	1.21×10^{-1}	0.021
Fungal landscape	*Betula*	-2.42×10^{-2}	0.939	2.44×10^{-1}	< 0.001	2.30×10^{-1}	< 0.001
Fungal landscape	*Pinus*	8.71×10^{-2}	0.733	1.44×10^{-1}	0.013	1.41×10^{-1}	0.017
Fungal landscape	*Populus*	-1.68×10^{-1}	0.681	2.02×10^{-1}	< 0.001	-2.51×10^{-2}	0.628
Fungal landscape	*Larix*	-1.34×10^{-1}	0.682	-5.32×10^{-1}	0.367	-1.34×10^{-1}	0.021
Fungal landscape	*Juglans*	1.81×10^{-1}	0.681	2.67×10^{-1}	< 0.001	1.38×10^{-1}	0.021
Prokaryotic landscape	*Acer*	1.86×10^{-1}	0.435	4.18×10^{-1}	< 0.001	3.71×10^{-1}	< 0.001
Prokaryotic landscape	*Betula*	8.71×10^{-2}	0.489	4.63×10^{-1}	< 0.001	4.51×10^{-1}	< 0.001
Prokaryotic landscape	*Pinus*	1.52×10^{-1}	0.435	4.17×10^{-1}	< 0.001	3.88×10^{-1}	< 0.001
Prokaryotic landscape	*Populus*	1.22×10^{-1}	0.435	4.88×10^{-1}	< 0.001	4.51×10^{-1}	< 0.001
Prokaryotic landscape	*Larix*	1.28×10^{-1}	0.435	4.02×10^{-1}	< 0.001	4.11×10^{-1}	< 0.001
Prokaryotic landscape	*Juglans*	1.67×10^{-1}	0.435	3.92×10^{-1}	< 0.001	4.41×10^{-1}	< 0.001

Supplementary Table S4 | Relationships between Δevenness and abundance or occupancy of microbial genera. Correlations between genus-level abundance (log-transformed relative read counts) or occupancy and z-standardized Δevenness values were evaluated to examine whether microbial genera with large effects on basin evenness in reconstructed assembly landscapes were associated with their dominance in the root microbiome dataset. For each host plant and each reconstructed assembly landscape, Kendall's rank correlation tests were performed between the z-standardized Δevenness values of microbial genera and their abundance or occupancy. The table lists Kendall's rank correlation coefficients (Kendall's τ) and false discovery rate-adjusted P values. P values were adjusted using the Benjamini–Hochberg procedure. The exact P values are provided in a Source Data file.

		Abundance (relative read counts)				Occupancy	
		Fungal genera		Prokaryotic genera		Fungal & Prokaryotic genera	
Landscapes	Host plant	Kendall's τ	P (FDR)	Kendall's τ	P (FDR)	Kendall's τ	P (FDR)
Fungal landscape	*Acer*	6.82×10^{-2}	0.804	-1.22×10^{-1}	0.121	-4.17×10^{-2}	0.715
Fungal landscape	*Betula*	5.24×10^{-2}	0.804	-2.95×10^{-4}	0.996	6.30×10^{-2}	0.649
Fungal landscape	*Pinus*	1.14×10^{-1}	0.804	1.12×10^{-1}	0.121	2.18×10^{-1}	< 0.001
Fungal landscape	*Populus*	3.91×10^{-2}	0.804	1.06×10^{-1}	0.121	2.75×10^{-2}	0.715
Fungal landscape	*Larix*	-3.70×10^{-2}	0.804	-3.41×10^{-3}	0.996	-4.16×10^{-3}	0.939
Fungal landscape	*Juglans*	1.30×10^{-1}	0.804	4.38×10^{-2}	0.712	-3.78×10^{-2}	0.715
Prokaryotic landscape	*Acer*	-3.79×10^{-2}	0.924	4.82×10^{-2}	0.472	-5.42×10^{-2}	0.478
Prokaryotic landscape	*Betula*	-1.14×10^{-2}	0.939	7.53×10^{-2}	0.273	-4.55×10^{-4}	0.237
Prokaryotic landscape	*Pinus*	5.68×10^{-2}	0.924	1.20×10^{-1}	0.068	8.25×10^{-2}	0.237
Prokaryotic landscape	*Populus*	1.13×10^{-1}	0.892	-1.82×10^{-1}	0.004	-2.80×10^{-1}	< 0.001
Prokaryotic landscape	*Larix*	1.11×10^{-1}	0.892	2.08×10^{-2}	0.725	-2.85×10^{-3}	0.982
Prokaryotic landscape	*Juglans*	1.16×10^{-1}	0.892	-2.41×10^{-1}	< 0.001	-3.14×10^{-1}	< 0.001

Supplementary Table S5 | Ectomycorrhizal fungal genera for which significant impacts on the fungal assembly landscape were inferred. The Δtopography and Δevenness indices respectively represented changes in the overall topography and evenness of energy landscape architecture along the abundance gradients of a focal genus (from absence to their median abundance condition; Fig. 1d–g). The *z*-standardized metrics of Δtopography and Δevenness are obtained based on the randomization analysis in which the abundance of a focal genus was shuffled within the root samples of the same host plant (10,000 iterations). The ectomycorrhizal genera exhibited significant Δtopography are listed. *P* values of Δtopography (one-sided test) and Δevenness (two-sided test) were separately adjusted using the Benjamini–Hochberg procedure. The exact *P* values are provided in a Source Data file.

Genus	Host plant	z-standardized Δtopography	*P* (FDR) [Δtopography]	z-standardized Δevenness	*P* (FDR) [Δevenness]
Amanita	*Populus*	8.83	**< 0.001**	3.23	0.081
Russula	*Acer*	4.38	**< 0.001**	2.55	**0.020**
Russula	*Juglans*	4.10	**< 0.001**	-1.89	0.086
Tomentella	*Populus*	2.87	**< 0.001**	2.27	0.081
Russula	*Pinus*	4.24	**0.001**	1.21	0.155
Thelephora	*Populus*	6.73	**0.001**	1.47	0.192
Tomentella	*Juglans*	3.19	**0.001**	-1.72	0.123
Sebacina	*Acer*	3.64	**0.002**	0.99	0.311
Amanita	*Larix*	4.85	**0.006**	0.89	0.311
Amanita	*Acer*	2.85	**0.007**	-0.45	0.372
Amanita	*Pinus*	3.14	**0.009**	-2.43	0.098
Sebacina	*Larix*	2.84	**0.012**	2.79	**0.025**
Tricholoma	*Betula*	1.60	**0.027**	-1.49	0.365
Thelephora	*Larix*	4.17	**0.029**	-0.28	0.710
Lactarius	*Acer*	2.09	**0.030**	1.77	0.151
Sebacina	*Pinus*	2.39	**0.035**	-2.14	0.148
Thelephora	*Pinus*	2.19	**0.042**	-2.26	0.157
Lactarius	*Larix*	3.52	**0.048**	-0.59	0.687

Supplementary Table S6 | Genera that exhibited the greatest potential impacts on the prokaryotic assembly landscape along their abundance gradients. The Δtopography and Δevenness indices respectively represented changes in the overall topography and evenness of energy landscape architecture along the abundance gradients of a focal genus (from absence to their median abundance condition; Fig. 1d–g). The *z*-standardized metrics of Δtopography and Δevenness are obtained based on the randomization analysis in which the abundance of a focal genus was shuffled within the root samples of the same host plant (10,000 iterations). For each host plant background, the two fungal/prokaryotic genera with the highest and significant Δtopography are listed. *P* values of Δtopography (one-sided test) and Δevenness (two-sided test) were separately adjusted using the Benjamini–Hochberg procedure. The exact *P* values are provided in a Source Data file.

Genus	Host plant	z-standardized Δtopography	*P* (FDR) [Δtopography]	z-standardized Δevenness	*P* (FDR) [Δevenness]
Candidatus Udaeobacter	*Betula*	2.70×10	**< 0.001**	-2.76	**0.020**
Candidatus Solibacter	*Betula*	2.67×10	**< 0.001**	3.94	**0.005**
Candidatus Udaeobacter	*Pinus*	2.63×10	**< 0.001**	4.49×10^{-5}	0.532
Candidatus Solibacter	*Pinus*	2.17×10	**< 0.001**	1.77	0.124
Candidatus Solibacter	*Acer*	2.34×10	**< 0.001**	2.57	0.039
Candidatus Udaeobacter	*Acer*	2.31×10	**< 0.001**	-0.42	0.434
Candidatus Udaeobacter	*Populus*	3.02×10	**< 0.001**	-3.02	**0.012**
Candidatus Solibacter	*Populus*	2.75×10	**< 0.001**	-2.73	0.029
Candidatus Udaeobacter	*Larix*	2.64×10	**< 0.001**	-2.00	0.090
Candidatus Solibacter	*Larix*	2.26×10	**< 0.001**	-0.51	0.407
Candidatus Udaeobacter	*Juglans*	3.26×10	**< 0.001**	-8.18	**0.002**
Candidatus Solibacter	*Juglans*	2.93×10	**< 0.001**	-8.42	**0.002**

Supplementary Table S7 | Ecological associations between candidate keystone genera and root mycorrhizal status. To independently evaluate the ecological associations of candidate keystone genera inferred in Figure 4b, we fitted generalized linear mixed models (GLMMs) using an independent root microbiome dataset collected across Europe (See Fig. 7). Separate models were fitted for root colonization rates by arbuscular mycorrhizal (AM; n = 219 samples) and ectomycorrhizal (EcM; n = 218 samples) fungi. For each response variable, the abundance of each focal genus was included as an explanatory variable of interest while accounting for environmental covariates. Focal genera were defined as the top 10 genera with the highest Δtopography values within each host-plant background in Figure 4b. Standardized partial regression coefficients are reported together with their standard errors, 95% confidence interval (CI; lower and upper limits), Wald z statistics and two-sided P values. P values were adjusted for multiple testing using the Benjamini–Hochberg procedure. Exact P values are provided in the Source Data file.

Root colonization	Focal genera	Standardized partial regression coefficients	standard error	95% CI (lower)	95% CI (upper)	Wald z	P (FDR; q-value)
AM	*Phialocephala*	-1.73×10^{-1}	4.37×10^{-2}	-2.59×10^{-1}	-8.74×10^{-2}	-3.96	< 0.001
AM	*Hyaloscypha*	5.67×10^{-2}	4.64×10^{-2}	-3.42×10^{-2}	1.48×10^{-1}	1.22	0.289
AM	*Mycena*	-4.91×10^{-1}	4.52×10^{-2}	-5.80×10^{-1}	-4.02×10^{-1}	-1.09×10	< 0.001
AM	*Oidiodendron*	-7.61×10^{-3}	4.56×10^{-2}	-9.70×10^{-2}	8.18×10^{-2}	-1.67×10^{-1}	0.901
AM	*Cladophialophora*	7.42×10^{-2}	4.94×10^{-2}	-2.26×10^{-2}	1.71×10^{-1}	1.50	0.199
AM	*Archaeorhizomyces*	1.37×10^{-1}	4.90×10^{-2}	4.09×10^{-2}	2.33×10^{-1}	2.79	0.011
AM	*Mortierella*	-2.76×10^{-1}	4.73×10^{-2}	-3.69×10^{-1}	-1.83×10^{-1}	-5.83	< 0.001
AM	*Penicillium*	-6.55×10^{-2}	5.06×10^{-2}	-1.65×10^{-1}	3.38×10^{-2}	-1.29	0.278
AM	*Pezicula*	-5.50×10^{-2}	4.57×10^{-2}	-1.45×10^{-1}	3.45×10^{-2}	-1.20	0.289
AM	*Amanita*	2.03×10^{-1}	4.94×10^{-2}	1.06×10^{-1}	3.00×10^{-1}	4.11	< 0.001
AM	*Ramariopsis*	7.12×10^{-2}	3.81×10^{-2}	-3.41×10^{-3}	1.46×10^{-1}	1.87	0.098
AM	*Glutinomyces*	1.60×10^{-2}	3.99×10^{-2}	-6.23×10^{-2}	9.43×10^{-2}	4.01×10^{-1}	0.775
AM	*Psathyrella*	-2.54×10^{-1}	5.02×10^{-2}	-3.52×10^{-1}	-1.56×10^{-1}	-5.06	< 0.001
AM	*Saitozyma*	-3.69×10^{-2}	3.23×10^{-2}	-1.00×10^{-1}	2.65×10^{-2}	-1.14	0.298
AM	*Rhizobium*	2.11×10^{-1}	6.68×10^{-2}	7.98×10^{-1}	3.42×10^{-1}	3.15	0.004
AM	*Pir4 lineage*	-1.17×10^{-1}	5.94×10^{-2}	-2.33×10^{-1}	-1.34×10^{-4}	-1.96	0.084
AM	*Acidipila-Silvibacterium*	1.29×10^{-1}	5.65×10^{-2}	1.82×10^{-1}	2.40×10^{-1}	2.28	0.043
AM	*Labrys*	-2.16×10^{-1}	5.80×10^{-2}	-3.29×10^{-1}	-1.02×10^{-1}	-3.72	< 0.001
AM	*Haliangium*	-1.37×10^{-2}	5.96×10^{-2}	-1.31×10^{-1}	1.03×10^{-1}	-2.30×10^{-1}	0.884
AM	*Chthoniobacter*	-5.49×10^{-3}	5.90×10^{-2}	-1.21×10^{-1}	1.10×10^{-1}	-9.30×10^{-2}	0.926

AM	*Actinospica*	1.58×10^{-1}	5.61×10^{-2}	4.84×10^{-2}	2.68×10^{-1}	2.82	0.011
AM	*Mucilaginibacter*	-6.39×10^{-2}	5.38×10^{-2}	-1.69×10^{-1}	4.16×10^{-2}	-1.19	0.289
AM	*Actinoplanes*	-2.94×10^{-1}	5.28×10^{-2}	-3.97×10^{-1}	-1.90×10^{-1}	-5.56	< 0.001
AM	*Inquilinus*	-1.72×10^{-1}	4.49×10^{-2}	-2.60×10^{-1}	-8.37×10^{-2}	-3.83	< 0.001
AM	*Spirochaeta 2*	2.47×10^{-1}	4.01×10^{-2}	1.68×10^{-1}	3.25×10^{-1}	6.16	< 0.001
AM	*HSB OF53-F07*	3.90×10^{-1}	4.67×10^{-2}	2.99×10^{-1}	4.82×10^{-1}	8.35	< 0.001
AM	*Streptacidiphilus*	-1.23×10^{-1}	5.65×10^{-2}	-2.33×10^{-1}	-1.19×10^{-2}	-2.17	0.054
EcM	*Phialocephala*	-1.21×10^{-2}	1.59×10^{-2}	-4.34×10^{-2}	1.92×10^{-2}	-7.59×10^{-1}	0.550
EcM	*Hyaloscypha*	-2.35×10^{-2}	1.60×10^{-2}	-5.48×10^{-2}	7.77×10^{-3}	-1.47	0.223
EcM	*Mycena*	-5.98×10^{-2}	1.68×10^{-2}	-9.27×10^{-2}	-2.70×10^{-2}	-3.57	0.003
EcM	*Oidiodendron*	-5.56×10^{-2}	1.70×10^{-2}	-8.89×10^{-2}	-2.23×10^{-2}	-3.28	0.007
EcM	*Cladophialophora*	-4.12×10^{-2}	1.67×10^{-2}	-7.39×10^{-2}	-8.47×10^{-3}	-2.47	0.046
EcM	*Archaeorhizomyces*	3.67×10^{-2}	1.71×10^{-2}	3.09×10^{-3}	7.03×10^{-2}	2.14	0.073
EcM	*Mortierella*	-1.85×10^{-2}	1.72×10^{-2}	-5.23×10^{-2}	1.53×10^{-2}	-1.07	0.404
EcM	*Penicillium*	-7.24×10^{-2}	1.61×10^{-2}	-1.04×10^{-1}	-4.08×10^{-2}	-4.49	< 0.001
EcM	*Pezicula*	-4.95×10^{-2}	1.82×10^{-2}	-8.51×10^{-2}	-1.38×10^{-2}	-2.72	0.035
EcM	*Amanita*	4.47×10^{-3}	1.53×10^{-2}	-2.55×10^{-2}	3.45×10^{-2}	2.92×10^{-1}	0.866
EcM	*Ramariopsis*	-2.07×10^{-2}	2.53×10^{-2}	-7.03×10^{-2}	2.89×10^{-2}	-8.18×10^{-1}	0.550
EcM	*Glutinomyces*	1.18×10^{-3}	2.52×10^{-2}	-4.81×10^{-2}	5.05×10^{-2}	4.69×10^{-2}	0.998
EcM	*Psathyrella*	2.46×10^{-2}	1.34×10^{-2}	-1.60×10^{-3}	5.09×10^{-2}	1.84	0.133
EcM	*Saitozyma*	-1.09×10^{-2}	1.38×10^{-2}	-3.80×10^{-2}	1.63×10^{-2}	-7.84×10^{-1}	0.550
EcM	*Rhizobium*	3.53×10^{-2}	2.07×10^{-2}	-5.24×10^{-3}	7.57×10^{-2}	1.71	0.153
EcM	*Pir4 lineage*	5.74×10^{-2}	2.41×10^{-2}	1.02×10^{-2}	1.05×10^{-1}	2.38	0.046
EcM	*Acidipila-Silvibacterium*	-4.21×10^{-2}	1.68×10^{-2}	-7.50×10^{-2}	-9.15×10^{-3}	-2.50	0.046
EcM	*Labrys*	3.51×10^{-2}	1.93×10^{-2}	-2.75×10^{-3}	7.29×10^{-2}	1.82	0.133
EcM	*Haliangium*	4.75×10^{-2}	1.86×10^{-2}	1.10×10^{-2}	8.40×10^{-2}	2.55	0.046
EcM	*Chthoniobacter*	-4.24×10^{-3}	2.01×10^{-2}	-4.37×10^{-2}	3.52×10^{-2}	-2.11×10^{-1}	0.900
EcM	*Actinospica*	2.98×10^{-2}	1.76×10^{-2}	-4.70×10^{-3}	6.43×10^{-2}	1.69	0.153
EcM	*Mucilaginibacter*	2.21×10^{-2}	1.86×10^{-2}	-1.44×10^{-2}	5.87×10^{-2}	1.19	0.352
EcM	*Actinoplanes*	-5.80×10^{-5}	2.34×10^{-2}	-4.58×10^{-2}	4.57×10^{-2}	-2.48×10^{-3}	0.998
EcM	*Inquilinus*	9.82×10^{-3}	1.78×10^{-2}	-2.51×10^{-2}	4.47×10^{-2}	5.51×10^{-1}	0.682
EcM	*Spirochaeta 2*	6.08×10^{-2}	1.58×10^{-2}	2.97×10^{-2}	9.18×10^{-2}	3.84	0.002
EcM	*HSB OF53-F07*	-4.02×10^{-2}	1.68×10^{-2}	-7.32×10^{-2}	-7.17×10^{-3}	-2.39	0.046
EcM	*Streptacidiphilus*	-3.66×10^{-2}	1.64×10^{-2}	-6.87×10^{-2}	-4.51×10^{-3}	-2.24	0.062

Supplementary Table S8 | Prioritization of fungal families based on their contributions to overall community structure. To select candidate family sets for subsequent analyses (see Supplementary Figs. S11–S13), individual fungal families were prioritized according to their explanatory power for the abundance-based fungal community structure. Specifically, a PerMANOVA was performed on the relative abundance matrix of fungal data using the presence/absence of each family as an explanatory variable (10,000 iterations). In the PerMANOVA, Bray–Curtis dissimilarity was used to quantify community dissimilarity between root samples, and the host plant backgrounds and sampling points were included as additional explanatory variables (i.e., covariates). The exact *P* values are provided in a Source Data file.

Family	R^2	*P* (FDR)
Russulaceae	3.76×10^{-2}	< 0.001
Thelephoraceae	1.50×10^{-2}	< 0.001
Hydnaceae	1.30×10^{-2}	< 0.001
Tricholomataceae	1.26×10^{-2}	< 0.001
Hyaloscyphaceae	1.12×10^{-2}	< 0.001
Glomeraceae	1.11×10^{-2}	< 0.001
Mollisiaceae	1.08×10^{-2}	< 0.001
Mycenaceae	9.92×10^{-3}	< 0.001
Herpotrichiellaceae	8.50×10^{-3}	< 0.001
Clavariaceae	6.55×10^{-3}	< 0.001
Dermateaceae	5.53×10^{-3}	< 0.001
Cephalothecaceae	5.24×10^{-3}	< 0.001
Amanitaceae	4.74×10^{-3}	< 0.001
Helotiaceae	4.49×10^{-3}	< 0.001
Mortierellaceae	4.44×10^{-3}	< 0.001
Hymenogastraceae	4.32×10^{-3}	< 0.001
Helotiales_fam_Incertae_sedis	4.31×10^{-3}	< 0.001
Inocybaceae	3.84×10^{-3}	< 0.001
Tarzettaceae	3.47×10^{-3}	< 0.001
Leotiaceae	3.42×10^{-3}	< 0.001
Myxotrichaceae	3.42×10^{-3}	< 0.001
Ceratobasidiaceae	3.40×10^{-3}	< 0.001
Archaeorhizomycetaceae	2.76×10^{-3}	< 0.001
Cortinariaceae	2.71×10^{-3}	< 0.001
Rozellomycota_fam_Incertae_sedis	2.70×10^{-3}	< 0.001
Strophariaceae	2.64×10^{-3}	< 0.001
Sebacinaceae	2.61×10^{-3}	< 0.001
Aspergillaceae	2.27×10^{-3}	< 0.001
Hydnangiaceae	2.25×10^{-3}	0.001

Pyronemataceae	2.24×10^{-3}	< 0.001
Geoglossaceae	2.21×10^{-3}	< 0.001
Trimorphomycetaceae	2.20×10^{-3}	0.002
Tylosporaceae	2.20×10^{-3}	< 0.001
Tremellodendropsidales_fam_Incertae_sedis	2.12×10^{-3}	< 0.001
Ramicandelaberales_fam_Incertae_sedis	2.05×10^{-3}	0.002
Entolomataceae	1.92×10^{-3}	0.001
Leucosporidiales_fam_Incertae_sedis	1.91×10^{-3}	< 0.001
Pezizaceae	1.85×10^{-3}	0.003
Tuberaceae	1.83×10^{-3}	< 0.001
Venturiaceae	1.76×10^{-3}	0.007
Trichosporonaceae	1.72×10^{-3}	0.004
Leptodontidiaceae	1.69×10^{-3}	0.010
Melanommataceae	1.58×10^{-3}	0.008
Psathyrellaceae	1.57×10^{-3}	0.009
Endogonaceae	1.51×10^{-3}	0.006
Cladosporiaceae	1.46×10^{-3}	0.015
Clavicipitaceae	1.43×10^{-3}	0.005
Hypocreaceae	1.43×10^{-3}	0.016
Nectriaceae	1.39×10^{-3}	0.008
Umbelopsidaceae	1.38×10^{-3}	0.014
Microbotryales_fam_Incertae_sedis	1.27×10^{-3}	0.017
Cordycipitaceae	1.24×10^{-3}	0.017
Piskurozymaceae	1.23×10^{-3}	0.016
GS11_fam_Incertae_sedis	1.20×10^{-3}	0.013
Pleosporaceae	1.19×10^{-3}	0.042
Mytilinidiaceae	1.06×10^{-3}	0.048

Supplementary Table S9 | Prioritization of prokaryotic families based on their contributions to overall community structure. To select candidate family sets for subsequent analyses (see Supplementary Figs. S11–S13), individual prokaryotic families were prioritized according to their explanatory power for the abundance-based prokaryotic community structure. Specifically, a PerMANOVA was performed on the relative abundance matrix of prokaryotic data, using the presence/absence of each family as an explanatory variable (10,000 iterations). In the PerMANOVA, Bray–Curtis dissimilarity was used to quantify community dissimilarity between root samples, and the host plant backgrounds and sampling points were included as additional explanatory variables (i.e., covariates). The exact *P* values are provided in a Source Data file.

Family	R^2	*P* (FDR)
Microbacteriaceae	2.10×10^{-2}	< 0.001
Kineosporiaceae	1.97×10^{-2}	< 0.001
Dongiaceae	1.97×10^{-2}	< 0.001
67-14	1.89×10^{-2}	< 0.001
Catenulisporaceae	1.79×10^{-2}	< 0.001
Ktedonobacteraceae	1.75×10^{-2}	< 0.001
Nocardioidaceae	1.70×10^{-2}	< 0.001
Xanthobacteraceae	1.56×10^{-2}	< 0.001
JG30-KF-AS9	1.48×10^{-2}	< 0.001
Hyphomicrobiaceae	1.42×10^{-2}	< 0.001
Sutterellaceae	1.39×10^{-2}	< 0.001
Frankiaceae	1.36×10^{-2}	< 0.001
Solibacteraceae	1.32×10^{-2}	< 0.001
Unknown Family	1.29×10^{-2}	< 0.001
Acetobacteraceae	1.27×10^{-2}	< 0.001
Nakamurellaceae	1.27×10^{-2}	< 0.001
Devosiaceae	1.23×10^{-2}	< 0.001
Thermoanaerobaculaceae	1.15×10^{-2}	< 0.001
URHD0088	1.13×10^{-2}	< 0.001
Beijerinckiaceae	1.12×10^{-2}	< 0.001
Microtrichaceae	1.12×10^{-2}	< 0.001
Solirubrobacteraceae	1.11×10^{-2}	< 0.001
Cellulomonadaceae	1.08×10^{-2}	< 0.001
Isosphaeraceae	1.05×10^{-2}	< 0.001
Pirellulaceae	1.05×10^{-2}	< 0.001
Xiphinematobacteraceae	1.03×10^{-2}	< 0.001
Acidothermaceae	1.03×10^{-2}	< 0.001
Nitrosomonadaceae	1.02×10^{-2}	< 0.001
Pedosphaeraceae	1.02×10^{-2}	< 0.001

Desulfitobacteriaceae	9.98×10^{-3}	< 0.001
A21b	9.65×10^{-3}	< 0.001
Bryobacteraceae	9.62×10^{-3}	< 0.001
Rhodomicrobiaceae	9.47×10^{-3}	< 0.001
Streptomycetaceae	9.37×10^{-3}	< 0.001
Acidobacteriaceae (Subgroup 1)	9.35×10^{-3}	< 0.001
Rhodanobacteraceae	9.28×10^{-3}	< 0.001
Sphingomonadaceae	9.24×10^{-3}	< 0.001
Pleomorphomonadaceae	9.22×10^{-3}	< 0.001
A0839	9.19×10^{-3}	< 0.001
CPla-3 termite group	9.13×10^{-3}	< 0.001
Ilumatobacteraceae	9.13×10^{-3}	< 0.001
Gemmatimonadaceae	9.07×10^{-3}	< 0.001
Reyranellaceae	8.88×10^{-3}	< 0.001
Gemmataceae	8.82×10^{-3}	< 0.001
Mycobacteriaceae	8.80×10^{-3}	< 0.001
Rhizobiaceae	8.32×10^{-3}	< 0.001
Micromonosporaceae	8.16×10^{-3}	< 0.001
Paenibacillaceae	8.03×10^{-3}	< 0.001
Burkholderiaceae	8.03×10^{-3}	< 0.001
Vermiphilaceae	7.94×10^{-3}	< 0.001
SC-I-84	7.90×10^{-3}	< 0.001
Legionellaceae	7.86×10^{-3}	< 0.001
Steroidobacteraceae	7.78×10^{-3}	< 0.001
WD2101 soil group	7.66×10^{-3}	< 0.001
Labraceae	7.65×10^{-3}	< 0.001
Microscillaceae	7.62×10^{-3}	< 0.001
Gimesiaceae	7.45×10^{-3}	< 0.001
Sphingobacteriaceae	7.42×10^{-3}	< 0.001
Myxococcaceae	7.42×10^{-3}	< 0.001
Pseudonocardiaceae	7.41×10^{-3}	< 0.001
Comamonadaceae	7.37×10^{-3}	< 0.001
LWQ8	7.32×10^{-3}	< 0.001
Rhizobiales Incertae Sedis	7.27×10^{-3}	< 0.001
Anaerolineaceae	7.03×10^{-3}	< 0.001
Polyangiaceae	7.02×10^{-3}	< 0.001
Oxalobacteraceae	6.71×10^{-3}	< 0.001
Magnetospirillaceae	6.71×10^{-3}	< 0.001
Spirochaetaceae	6.69×10^{-3}	< 0.001

Hyphomonadaceae	6.64×10^{-3}	< 0.001
Sporichthyaceae	6.40×10^{-3}	< 0.001
Blastocatellaceae	6.03×10^{-3}	< 0.001
Methylacidiphilaceae	6.02×10^{-3}	< 0.001
env.OPS 17	6.01×10^{-3}	< 0.001
WWH38	5.91×10^{-3}	< 0.001
Saccharimonadaceae	5.71×10^{-3}	< 0.001
Anaeromyxobacteraceae	5.55×10^{-3}	< 0.001
Chthoniobacteraceae	5.52×10^{-3}	< 0.001
Bacillaceae	5.42×10^{-3}	< 0.001
Lachnospiraceae	5.34×10^{-3}	< 0.001
Micropepsaceae	5.21×10^{-3}	< 0.001
Opitutaceae	5.08×10^{-3}	< 0.001
Clostridiaceae	5.07×10^{-3}	< 0.001
Actinospicaceae	5.07×10^{-3}	< 0.001
Caulobacteraceae	4.96×10^{-3}	< 0.001
KF-JG30-B3	4.81×10^{-3}	< 0.001
Phycisphaeraceae	4.79×10^{-3}	< 0.001
JG30-KF-CM45	4.78×10^{-3}	< 0.001
Chthonomonadaceae	4.76×10^{-3}	< 0.001
Koribacteraceae	4.70×10^{-3}	< 0.001
Haliangiaceae	4.54×10^{-3}	< 0.001
Sandaracinaceae	4.44×10^{-3}	< 0.001
Parvibaculaceae	4.35×10^{-3}	< 0.001
Pyrinomonadaceae	4.34×10^{-3}	< 0.001
Xanthomonadaceae	4.27×10^{-3}	< 0.001
Bdellovibrionaceae	4.22×10^{-3}	< 0.001
Simkaniaceae	4.15×10^{-3}	< 0.001
Parachlamydiaceae	4.13×10^{-3}	< 0.001
BIrii41	4.04×10^{-3}	< 0.001
A4b	3.99×10^{-3}	< 0.001
Pseudomonadaceae	3.76×10^{-3}	< 0.001
Phaselicystidaceae	3.74×10^{-3}	< 0.001
Chitinophagaceae	3.72×10^{-3}	< 0.001
SM2D12	3.67×10^{-3}	< 0.001
Thermomonosporaceae	3.65×10^{-3}	< 0.001
Rhodospirillaceae	3.63×10^{-3}	< 0.001
Amoebophilaceae	3.60×10^{-3}	< 0.001
CWT CU03-E12	3.60×10^{-3}	< 0.001
Nitrospiraceae	3.57×10^{-3}	< 0.001

Alicyclobacillaceae	3.54×10^{-3}	< 0.001
Inquilinaceae	3.53×10^{-3}	< 0.001
cvE6	3.53×10^{-3}	< 0.001
Coxiellaceae	3.50×10^{-3}	< 0.001
Verrucomicrobiaceae	3.45×10^{-3}	< 0.001
Elsteraceae	3.38×10^{-3}	< 0.001
UBA12409	3.37×10^{-3}	< 0.001
Chlamydiaceae	3.29×10^{-3}	< 0.001
Flavobacteriaceae	3.26×10^{-3}	< 0.001
Obscuribacteraceae	3.21×10^{-3}	< 0.001
Gaiellaceae	3.19×10^{-3}	< 0.001
Vicinamibacteraceae	3.10×10^{-3}	< 0.001
Propionibacteriaceae	3.03×10^{-3}	< 0.001
WX65	3.02×10^{-3}	< 0.001
Thermoactinomycetaceae	3.02×10^{-3}	< 0.001
Babeliaceae	2.99×10^{-3}	< 0.001
Fimbriimonadaceae	2.87×10^{-3}	< 0.001
Diplorickettsiaceae	2.82×10^{-3}	< 0.001
TRA3-20	2.75×10^{-3}	< 0.001
AKYH767	2.74×10^{-3}	< 0.001
Holosporaceae	2.71×10^{-3}	< 0.001
Schlesneriaceae	2.62×10^{-3}	< 0.001
37-13	2.59×10^{-3}	< 0.001
Methyloligellaceae	2.56×10^{-3}	< 0.001
Peptostreptococcaceae	2.49×10^{-3}	< 0.001
Moraxellaceae	2.42×10^{-3}	< 0.001
Terrimicrobiaceae	2.28×10^{-3}	< 0.001
Iamiaceae	2.27×10^{-3}	< 0.001
Leptospiraceae	2.27×10^{-3}	0.002
Azospirillaceae	2.24×10^{-3}	0.002
Paracaedibacteraceae	2.14×10^{-3}	< 0.001
Rickettsiaceae	2.12×10^{-3}	< 0.001
Planococcaceae	2.12×10^{-3}	0.001
Candidatus Jidaibacter	2.10×10^{-3}	0.001
Streptosporangiaceae	1.95×10^{-3}	0.002
Armatimonadaceae	1.91×10^{-3}	< 0.001
Rhodobacteraceae	1.88×10^{-3}	0.008
Yersiniaceae	1.87×10^{-3}	0.008
Erysipelotrichaceae	1.86×10^{-3}	0.001
KD3-93	1.81×10^{-3}	0.005

Acholeplasmataceae	1.68×10^{-3}	0.002
Rhodocyclaceae	1.66×10^{-3}	0.003
Oligoflexaceae	1.62×10^{-3}	0.002
YM_S32_TM7_50_20	1.60×10^{-3}	0.002
Cytophagaceae	1.60×10^{-3}	0.003
Vampirovibrionaceae	1.48×10^{-3}	0.006
Nocardiaceae	1.47×10^{-3}	0.011
Chitinimonadaceae	1.41×10^{-3}	0.011
type III	1.40×10^{-3}	0.006
Intrasporangiaceae	1.39×10^{-3}	0.029
NS11-12 marine group	1.30×10^{-3}	0.034
Ruminococcaceae	1.28×10^{-3}	0.007
Solimonadaceae	1.21×10^{-3}	0.016
Neisseriaceae	1.15×10^{-3}	0.021
Micavibrionaceae	1.06×10^{-3}	0.029
Puniceicoccaceae	1.02×10^{-3}	0.032

Supplementary Table S10 | Fungal family list selected using the uniform abundance threshold. After binarization using the optimal combination of the abundance threshold and number of families (the threshold of relative read counts = 0.1; Supplementary Fig. S26), the community-states matrix for energy landscape analysis consisted of 29 fungal families. Specifically, PerMANOVA was performed for each fungal family to evaluate the correspondence of its presence/absence pattern with community structure (Bray–Curtis dissimilarities among community compositions of root samples). Based on R^2 in the PerMANOVA, the optimal family set showing highest correspondence with candidate community memberships was selected (See Supplementary Note 4 for details). The exact *P* values are provided in a Source Data file.

Family	Occurrence	*R2*	*P* (FDR)
Russulaceae	610	1.05×10^{-1}	< 0.001
Thelephoraceae	251	6.64×10^{-2}	< 0.001
Hyaloscyphaceae	437	6.10×10^{-2}	< 0.001
Hydnaceae	133	4.35×10^{-2}	< 0.001
Tricholomataceae	83	4.28×10^{-2}	< 0.001
Mollisiaceae	193	3.64×10^{-2}	< 0.001
Mycenaceae	67	2.43×10^{-2}	< 0.001
Amanitaceae	44	1.83×10^{-2}	< 0.001
Glomeraceae	38	1.57×10^{-2}	< 0.001
Herpotrichiellaceae	80	1.49×10^{-2}	< 0.001
Inocybaceae	29	1.26×10^{-2}	< 0.001
Tarzettaceae	22	1.10×10^{-2}	< 0.001
Sebacinaceae	20	9.14×10^{-3}	< 0.001
Helotiaceae	40	8.13×10^{-3}	< 0.001
Tylosporaceae	18	7.72×10^{-3}	< 0.001
Hymenogastraceae	19	7.06×10^{-3}	< 0.001
Myxotrichaceae	33	6.48×10^{-3}	< 0.001
Ceratobasidiaceae	13	6.41×10^{-3}	< 0.001
Archaeorhizomycetaceae	31	6.27×10^{-3}	< 0.001
Pyronemataceae	19	6.09×10^{-3}	< 0.001
Cortinariaceae	19	6.07×10^{-3}	< 0.001
Leotiaceae	19	5.80×10^{-3}	< 0.001
Hydnangiaceae	11	5.62×10^{-3}	< 0.001
Dermateaceae	23	5.46×10^{-3}	< 0.001
Mortierellaceae	23	5.11×10^{-3}	< 0.001
Clavariaceae	28	4.67×10^{-3}	< 0.001

Cephalothecaceae	14	4.50×10^{-3}	< 0.001
Strophariaceae	26	4.10×10^{-3}	< 0.001
Tuberaceae	11	3.34×10^{-3}	< 0.001

Supplementary Table S11 | Prokaryotic family list selected in the uniform abundance threshold. After binarization using the optimal combination of the abundance threshold (the threshold of relative read counts = 0.01; Supplementary Fig. S26), the community-states matrix for energy landscape analysis consisted of 49 prokaryotic families. Specifically, PerMANOVA was performed for each prokaryotic family to evaluate the correspondence of its presence/absence pattern with community structure (Bray–Curtis dissimilarities among community compositions of root samples). Based on R^2 in the PerMANOVA, the optimal family set showing highest correspondence with candidate community memberships was selected (See Supplementary Note 4 for details). The exact *P* values are provided in a Source Data file.

Family	Occurrence	*R2*	*P* (FDR)
Ktedonobacteraceae	641	3.91×10^{-3}	0.003
67-14	195	3.20×10^{-3}	0.003
Sphingobacteriaceae	156	3.20×10^{-3}	0.003
Microbacteriaceae	29	2.88×10^{-3}	0.003
Acidobacteriaceae (Subgroup 1)	536	2.52×10^{-3}	0.003
Methylacidiphilaceae	210	2.32×10^{-3}	0.003
Frankiaceae	95	2.29×10^{-3}	0.007
Unknown Family	1232	2.18×10^{-3}	0.005
Pirellulaceae	279	2.11×10^{-3}	0.005
Beijerinckiaceae	591	2.10×10^{-3}	0.003
Rhodanobacteraceae	248	1.95×10^{-3}	0.003
Rhizobiaceae	203	1.90×10^{-3}	0.013
Burkholderiaceae	554	1.85×10^{-3}	0.005
Pseudonocardiaceae	161	1.81×10^{-3}	0.003
Comamonadaceae	466	1.78×10^{-3}	0.025
Thermomonosporaceae	12	1.65×10^{-3}	0.015
Sphingomonadaceae	22	1.63×10^{-3}	0.015
Legionellaceae	37	1.56×10^{-3}	0.017
Bacillaceae	330	1.52×10^{-3}	0.024
Solibacteraceae	155	1.47×10^{-3}	0.031
Acetobacteraceae	238	1.39×10^{-3}	0.050
Micromonosporaceae	204	1.33×10^{-3}	0.028
Xiphinematobacteraceae	253	1.32×10^{-3}	0.026
Chthoniobacteraceae	449	1.28×10^{-3}	0.026
Solirubrobacteraceae	753	1.28×10^{-3}	0.041
Oxalobacteraceae	109	1.25×10^{-3}	0.073

Bryobacteraceae	245	1.23×10^{-3}	0.041
Mycobacteriaceae	839	1.23×10^{-3}	0.026
Vermiphilaceae	86	1.20×10^{-3}	0.041
Isosphaeraceae	1002	1.11×10^{-3}	0.085
Haliangiaceae	307	1.09×10^{-3}	0.081
Myxococcaceae	15	1.05×10^{-3}	0.127
type III	40	1.02×10^{-3}	0.071
SM2D12	43	1.01×10^{-3}	0.176
Pedosphaeraceae	658	9.80×10^{-4}	0.091
Spirochaetaceae	93	9.75×10^{-4}	0.151
Actinospicaceae	309	9.72×10^{-4}	0.089
Gemmataceae	791	9.67×10^{-4}	0.124
Gemmatimonadaceae	21	9.44×10^{-4}	0.091
Pseudomonadaceae	20	9.37×10^{-4}	0.075
Micropepsaceae	439	8.59×10^{-4}	0.175
LWQ8	100	8.32×10^{-4}	0.299
Polyangiaceae	841	8.03×10^{-4}	0.208
Hyphomicrobiaceae	81	7.62×10^{-4}	0.246
Inquilinaceae	33	7.60×10^{-4}	0.264
Microscillaceae	55	7.58×10^{-4}	0.367
Streptomycetaceae	307	7.55×10^{-4}	0.367
Acidothermaceae	1210	7.44×10^{-4}	0.545
Magnetospirillaceae	121	7.31×10^{-4}	0.289

Supplementary Table S12 | Fungal genus set selected in genus-level energy landscape analysis. To perform energy landscape analysis for the genus-level fungal community states, the 20 genera were selected in the same way as the family-level analysis (Supplementary Figs. S11–S13, S36–S37). Specifically, PerMANOVA was performed for each fungal genus to evaluate the correspondence of its presence/absence pattern with community structure (Bray–Curtis dissimilarities among genus-level community compositions of root samples). Based on R^2 in the PerMANOVA, the optimal genus set showing highest correspondence with candidate community memberships was selected (See Supplementary Note 9 for details). The exact *P* values are provided in a Source Data file.

Genus	Occurrence	R^2	*P* (FDR)
Russula	801	3.51×10^{-2}	< 0.001
Lactarius	232	2.01×10^{-2}	< 0.001
Tomentella	525	1.39×10^{-2}	< 0.001
Leucoscypha	53	1.33×10^{-2}	< 0.001
Phialocephala	737	1.24×10^{-2}	< 0.001
Tricholoma	154	1.22×10^{-2}	< 0.001
Hyaloscypha	843	1.16×10^{-2}	< 0.001
Mycena	202	1.07×10^{-2}	< 0.001
Glutinomyces	515	1.01×10^{-2}	< 0.001
Cladophialophora	557	8.35×10^{-3}	< 0.001
Oehlia	80	7.34×10^{-3}	< 0.001
Cephalotheca	211	5.75×10^{-3}	< 0.001
Pezicula	148	5.54×10^{-3}	< 0.001
Cistella	129	5.34×10^{-3}	< 0.001
Inocybe	139	4.83×10^{-3}	< 0.001
Amanita	169	4.67×10^{-3}	< 0.001
Meliniomyces	224	4.57×10^{-3}	< 0.001
Ramariopsis	170	4.43×10^{-3}	< 0.001
Gorgomyces	69	4.38×10^{-3}	< 0.001
Tarzetta	41	3.68×10^{-3}	< 0.001

Supplementary Table S13 | Prokaryotic genus set selected in genus-level energy landscape analysis. To perform energy landscape analysis for the genus-level prokaryotic community states, the 49 genera were selected in the same way as the family-level analysis (Supplementary Figs. S11–S13, S36–S37). Specifically, PerMANOVA was performed for each prokaryotic genus to evaluate the correspondence of its presence/absence pattern with community structure (Bray–Curtis dissimilarities among genus-level community compositions of root samples). Based on R^2 in the PerMANOVA, the optimal genus showing highest correspondence with candidate community memberships was selected (See Supplementary Note 9 for details). The exact *P* values are provided in a Source Data file.

Genus	Occurrence	*R2*	*P* (FDR)
Thermosporothrix	614	2.42×10^{-2}	< 0.001
Galbitalea	197	1.70×10^{-2}	< 0.001
Conexibacter	846	1.67×10^{-2}	< 0.001
Candidatus Solibacter	905	1.62×10^{-2}	< 0.001
Catenulispora	526	1.62×10^{-2}	< 0.001
Dongia	227	1.53×10^{-2}	< 0.001
Angustibacter	129	1.44×10^{-2}	< 0.001
Gemmatimonas	357	1.41×10^{-2}	< 0.001
Actinoplanes	94	1.35×10^{-2}	< 0.001
Dyella	364	1.30×10^{-2}	< 0.001
Acidibacter	1060	1.29×10^{-2}	< 0.001
Jatrophihabitans	464	1.28×10^{-2}	< 0.001
Nocardioides	155	1.26×10^{-2}	< 0.001
Rhizobacter	435	1.25×10^{-2}	< 0.001
Pedomicrobium	599	1.20×10^{-2}	< 0.001
Aquisphaera	954	1.17×10^{-2}	< 0.001
Methylocapsa	600	1.16×10^{-2}	< 0.001
Candidatus Udaeobacter	768	1.14×10^{-2}	< 0.001
HSB OF53-F07	278	1.08×10^{-2}	< 0.001
Pir4 lineage	345	1.07×10^{-2}	< 0.001
Roseiarcus	872	1.06×10^{-2}	< 0.001
Devosia	288	1.05×10^{-2}	< 0.001
Occallatibacter	493	1.03×10^{-2}	< 0.001
Pandoraea	110	1.03×10^{-2}	< 0.001
Streptacidiphilus	687	1.02×10^{-2}	< 0.001
Bryobacter	971	1.02×10^{-2}	< 0.001
Candidatus Xiphinematobacter	906	1.01×10^{-2}	< 0.001

Acidothermus	1063	1.00×10^{-2}	< 0.001
Nakamurella	120	9.96×10^{-3}	< 0.001
Subgroup 10	508	9.80×10^{-3}	< 0.001
Bradyrhizobium	1073	9.68×10^{-3}	< 0.001
Rhodomicrobium	554	9.37×10^{-3}	< 0.001
Dinghuibacter	130	9.32×10^{-3}	< 0.001
Sphaerotilus	800	9.19×10^{-3}	< 0.001
Solirubrobacter	103	9.02×10^{-3}	< 0.001
Reyranella	800	8.99×10^{-3}	< 0.001
Cellulomonas	57	8.97×10^{-3}	< 0.001
Rhizobium	597	8.64×10^{-3}	< 0.001
Granulicella	813	8.43×10^{-3}	< 0.001
Acidicapsa	523	8.17×10^{-3}	< 0.001
Paenibacillus	891	8.11×10^{-3}	< 0.001
Kutzneria	242	7.87×10^{-3}	< 0.001
Singulisphaera	869	7.77×10^{-3}	< 0.001
Dokdonella	144	7.66×10^{-3}	< 0.001
Hyphomicrobium	314	7.51×10^{-3}	< 0.001
Sphingomonas	325	7.50×10^{-3}	< 0.001
Mycobacterium	1047	7.48×10^{-3}	< 0.001
Acidipila-Silvibacterium	614	7.45×10^{-3}	< 0.001
CL500-29 marine group	76	7.44×10^{-3}	< 0.001

Supplementary Table S14 | Genera that exhibited the greatest potential impacts on the fungal assembly landscape at the 25% quantiles of their abundance. The z-standardized Δtopography and Δevenness indices were calculated along the abundance gradients of a focal genus (from absence to the 25% abundance quantile within detected samples; Fig. 1d–g). For each host plant background, the two fungal/prokaryotic genera with the highest and significant Δtopography are listed. *P* values of Δtopography (one-sided test) and Δevenness (two-sided test) were separately adjusted using the Benjamini–Hochberg procedure. The exact *P* values are provided in a Source Data file.

Genus	Host plant	z-standardized Δtopography	*P* (FDR) [Δtopography]	z-standardized Δevenness	*P* (FDR) [Δevenness]
Archaeorhizomyces	*Acer*	8.12	**< 0.001**	-1.44	0.091
Haliangium	*Acer*	7.16	**< 0.001**	-3.35	0.026
Hyaloscypha	*Betula*	4.30	**< 0.001**	-2.09	0.107
Podila	*Betula*	3.10	**< 0.001**	-0.74	0.287
Hyaloscypha	*Juglans*	9.58	**< 0.001**	3.86	**0.002**
Haliangium	*Juglans*	6.85	**< 0.001**	-2.48	0.035
Oidiodendron	*Larix*	6.20	**< 0.001**	6.11×10	**0.002**
Podila	*Larix*	1.08	**0.001**	6.58	**0.002**
Podila	*Pinus*	1.05	**< 0.001**	0.48	0.301
Cladophialophora	*Pinus*	8.92	**< 0.001**	0.27	0.112
Cladophialophora	*Populus*	1.95×10	**< 0.001**	2.34	0.105
Pezicula	*Populus*	1.46	**< 0.001**	2.82	0.112

Supplementary Table S15 | Genera that exhibited the greatest potential impacts on the prokaryotic assembly landscape at the 25% quantiles of their abundance. The z-standardized Δtopography and Δevenness indices were calculated along the abundance gradients of a focal genus (from absence to the 25% abundance quantile within detected samples; Fig. 1d–g). For each host plant background, the two fungal/prokaryotic genera with the highest and significant Δtopography are listed. *P* values of Δtopography (one-sided test) and Δevenness (two-sided test) were separately adjusted using the Benjamini–Hochberg procedure. The exact *P* values are provided in a Source Data file.

Genus	Host plant	z-standardized Δtopography	*P* (FDR) [Δtopography]	z-standardized Δevenness	*P* (FDR) [Δevenness]
Candidatus Solibacter	*Acer*	2.31×10	< **0.001**	1.88	0.120
Mycobacterium	*Acer*	2.22×10	< **0.001**	-1.24	0.225
Pir4 lineage	*Betula*	2.56×10	< **0.001**	-2.93	0.034
Candidatus Solibacter	*Betula*	2.53×10	< **0.001**	8.14	**0.002**
Candidatus Udaeobacter	*Juglans*	3.27×10	< **0.001**	-5.50	**0.002**
Candidatus Solibacter	*Juglans*	2.82×10	< **0.001**	-5.24	**0.002**
Candidatus Udaeobacter	*Larix*	2.35×10	< **0.001**	-1.90	0.106
Labrys	*Larix*	2.29×10	< **0.001**	-0.36	0.447
Pir4 lineage	*Pinus*	2.18×10	< **0.001**	-3.59	**0.007**
Candidatus Udaeobacter	*Pinus*	2.15×10	< **0.001**	2.58	0.043
Candidatus Udaeobacter	*Populus*	2.96×10	< **0.001**	-0.97	0.285
Candidatus Solibacter	*Populus*	2.80×10	< **0.001**	-2.01	0.092

Supplementary Table S16 | Genera that exhibited the greatest potential impacts on the fungal assembly landscape at the 75% quantiles of their abundance. The z-standardized Δtopography and Δevenness indices were calculated along the abundance gradients of a focal genus (from absence to the 75% abundance quantile within detected samples; Fig. 1d–g). For each host plant background, the two fungal/prokaryotic genera with the highest and significant Δtopography are listed. *P* values of Δtopography (one-sided test) and Δevenness (two-sided test) were separately adjusted using the Benjamini–Hochberg procedure. The exact *P* values are provided in a Source Data file.

Genus	Host plant	z-standardized Δtopography	*P* (FDR) [Δtopography]	z-standardized Δevenness	*P* (FDR) [Δevenness]
Hyaloscypha	*Betula*	4.75	< **0.001**	-1.19	0.387
Actinospica	*Betula*	3.55	< **0.001**	0.19	0.419
Podila	*Pinus*	1.05×10	< **0.001**	-2.04	0.045
Phialocephala	*Pinus*	7.32	< **0.001**	-3.69	0.039
Oidiodendron	*Acer*	1.23×10	< **0.001**	3.80	**0.016**
Podila	*Acer*	9.28	< **0.001**	-0.94	0.246
Cladophialophora	*Populus*	2.48×10	< **0.001**	9.56	**0.002**
Oidiodendron	*Populus*	1.65×10	< **0.001**	-4.03	**0.020**
Cladophialophora	*Larix*	5.30×10	< **0.001**	0.10	0.944
Phialocephala	*Larix*	1.52×10	< **0.001**	3.30	0.049
Hyaloscypha	*Juglans*	1.02×10	< **0.001**	5.25	**0.002**
Meliniomyces	*Juglans*	9.31	< **0.001**	0.20	0.487

Supplementary Table S17 | Genera that exhibited the greatest potential impacts on the prokaryotic assembly landscape at the 75% quantiles of their abundance. The z-standardized Δtopography and Δevenness indices were calculated along the abundance gradients of a focal genus (from absence to the 75% abundance quantile within detected samples; Fig. 1d–g). For each host plant background, the two fungal/prokaryotic genera with the highest and significant Δtopography are listed. *P* values of Δtopography (one-sided test) and Δevenness (two-sided test) were separately adjusted using the Benjamini–Hochberg procedure. The exact *P* values are provided in a Source Data file.

Genus	Host plant	z-standardized Δtopography	P (FDR) [Δtopography]	z-standardized Δevenness	P (FDR) [Δevenness]
Candidatus Solibacter	*Betula*	2.66×10	**< 0.001**	1.97	0.115
Candidatus Udaeobacter	*Betula*	2.48×10	**< 0.001**	-3.66	**0.004**
Candidatus Udaeobacter	*Pinus*	2.57×10	**< 0.001**	-0.62	0.375
Candidatus Solibacter	*Pinus*	2.31×10	**< 0.001**	1.39	0.196
Candidatus Solibacter	*Acer*	2.50×10	**< 0.001**	1.02	0.285
Candidatus Udaeobacter	*Acer*	2.38×10	**< 0.001**	-1.63	0.151
Candidatus Solibacter	*Populus*	2.82×10	**< 0.001**	-4.23	**0.002**
Candidatus Udaeobacter	*Populus*	2.73×10	**< 0.001**	-4.90	**0.002**
Candidatus Udaeobacter	*Larix*	2.61×10	**< 0.001**	-4.86	**0.002**
Candidatus Solibacter	*Larix*	2.35×10	**< 0.001**	-3.93	**0.002**
Candidatus Udaeobacter	*Juglans*	3.07×10	**< 0.001**	-7.48	**0.002**
Candidatus Solibacter	*Juglans*	2.85×10	**< 0.001**	-7.74	**0.002**

Supplementary References

1. van Nes, E. H. & Scheffer, M. Large species shifts triggered by small forces. *Am. Nat.* **164**, 255–266 (2004).
2. Akjouj, I. et al. Complex systems in ecology: a guided tour with large Lotka–Volterra models and random matrices. Proc. R. Soc. A 480, 20230284 (2024).
3. Suzuki, K., Takahashi, K. & Harada, N. Evaluation of primer pairs for studying arbuscular mycorrhizal fungal community compositions using a MiSeq platform. *Biol. Fertil. Soils* **56**, 853–858 (2020).
4. Cohen, M. A. & Grossberg, S. Absolute stability of global pattern formation and parallel memory storage by competitive neural networks. *IEEE Trans. Syst. Man Cybern.* **SMC-13**, 815–826 (1983).
5. Faust, K. & Raes, J. Microbial interactions: from networks to models. *Nat. Rev. Microbiol.* **10**, 538–550 (2012).
6. Coyte, K. Z., Schluter, J. & Foster, K. R. The ecology of the microbiome: networks, competition, and stability. *Science* **350**, 663–666 (2015).
7. May, R. M. Limit cycles in predator-prey communities. *Science* **177**, 900–902 (1972).
8. Xu, L., Zhang, F., Zhang, K., Wang, E. & Wang, J. The potential and flux landscape theory of ecology. *PLOS ONE* **9**, e86746 (2014).
9. Onuchic, J. N., Luthey-Schulten, Z. & Wolynes, P. G. Theory of protein folding: the energy landscape perspective. Annu. Rev. Phys. Chem. 48, 545–600 (1997).
10. Tapia-Rojo, R., Alonso-Caballero, A., Badilla, C. L. & Fernandez, J. M. Identical sequences, different behaviors: protein diversity captured at the single-molecule level. *Biophys. J.* **123**, 814–823 (2024).
11. Benozzo, D. *et al.* Analyzing asymmetry in brain hierarchies with a linear state-space model of resting-state fMRI data. *Netw. Neurosci.* **8**, 965–988 (2024).
12. Masuda, N., Islam, S., Aung, S. T. & Watanabe, T. Energy landscape analysis based on the Ising model: tutorial review. *PLOS Complex Syst.* **2**, e0000039 (2025).
13. Kadoya, T., Suzuki, K. & Terui, A. Linking energetic instability to compositional changes in biological communities. Proc. Natl Acad. Sci. USA 122, e2422701122 (2025).
14. Maechler, M., Rousseeuw, P. J., Struyf, A., Hubert, M. & Hornik, K. cluster: Cluster Analysis Basics and Extensions. R package version 2.1.6 https://CRAN.R-project.org/package=cluster (2025).
