# Supplementary material for: Statistical inference of keystone taxa reshaping the assembly rules of forest root microbiomes
Source: Nat Commun. 2026 Oct 28;17:10997. doi: 10.1038/s41467-026-78401-3 (PMC13716906; doi:10.1038/s41467-026-78401-3)
Supplement: Supplementary file 2 [file 41467_2026_78401_MOESM2_ESM.pdf]

Description of Additional Supplementary Files

File name: Supplementary Data 1
Description: PCR primer sequences used in the study.
