# Supplementary material for: Statistical inference of keystone taxa reshaping the assembly rules of forest root microbiomes
Source: Nat Commun. 2026 Oct 28;17:10997. doi: 10.1038/s41467-026-78401-3 (PMC13716906; doi:10.1038/s41467-026-78401-3)
Supplement: Supplementary file 3 [file 41467_2026_78401_MOESM4_ESM.pdf]

nature portfolio

Corresponding author(s): Mikihito Noguchi
Hirokazu Toju

Last updated by author(s): Aug 12, 2026

Reporting Summary

Nature Portfolio wishes to improve the reproducibility of the work that we publish. This form provides structure for consistency and transparency in reporting. For further information on Nature Portfolio policies, see our Editorial Policies and the Editorial Policy Checklist.

Statistics

For all statistical analyses, confirm that the following items are present in the figure legend, table legend, main text, or Methods section.

n/a	Confirmed	
☐	☒	The exact sample size (*n*) for each experimental group/condition, given as a discrete number and unit of measurement
☐	☒	A statement on whether measurements were taken from distinct samples or whether the same sample was measured repeatedly
☐	☒	The statistical test(s) used AND whether they are one- or two-sided<br>*Only common tests should be described solely by name; describe more complex techniques in the Methods section.*
☐	☒	A description of all covariates tested
☐	☒	A description of any assumptions or corrections, such as tests of normality and adjustment for multiple comparisons
☐	☒	A full description of the statistical parameters including central tendency (e.g. means) or other basic estimates (e.g. regression coefficient) AND variation (e.g. standard deviation) or associated estimates of uncertainty (e.g. confidence intervals)
☐	☒	For null hypothesis testing, the test statistic (e.g. *F*, *t*, *r*) with confidence intervals, effect sizes, degrees of freedom and *P* value noted<br>*Give P values as exact values whenever suitable.*
☒	☐	For Bayesian analysis, information on the choice of priors and Markov chain Monte Carlo settings
☒	☐	For hierarchical and complex designs, identification of the appropriate level for tests and full reporting of outcomes
☐	☒	Estimates of effect sizes (e.g. Cohen's *d*, Pearson's *r*), indicating how they were calculated

Our web collection on statistics for biologists contains articles on many of the points above.

Software and code

Policy information about availability of computer code

Data collection	The computer code is available from the GitHub repository (https://github.com/ngchngch/Assembly_landscape_of_the_forest_root_microbiome) and has been archived in Zenodo99 under DOI: https://doi.org/10.5281/zenodo.21888541.<br>Sequencing data of root microbiome in Japan were obtained with the Illumina MiSeq platform. Reads were processed using bcl2fastq (version 1.8.4), Claident (version 0.9.2022.01.26), Cutadapt (version. 3.7), R (version 4.2.2), DADA2 (version 1.18.0), decontam (version 1.1.1) and VSEARCH (version 2.21.1).
Data analysis	The computer code is available from the GitHub repository (https://github.com/ngchngch/Assembly_landscape_of_the_forest_root_microbiome) and has been archived in Zenodo99 under DOI: https://doi.org/10.5281/zenodo.21888541.<br>Analyses were conducted in R (version 4.4.1) using vegan (version 2.6-8), rELA (version 0.60), compositions (version 2.0.8), purr (version 1.0.4), stringdist (version 0.9.15), parallel (version 4.4.1), foreach (version 1.5.2), doParallell (version 1.0.17), dplyr (version 1.1.4), ComplexHeatmap (version 2.22.0), circlize (version 0.4.16), cluster (version 2.1.8), tidyverse (version 2.0.0), renv (version 1.1.4), patchwork (version 1.3.0), ggrepel (version 0.9.6), ggalluvial (version 0.12.5), Rcpp (version 1.0.14), RcppArmadillo (version 14.4.3.1), gtools (version 3.9.5), ggplot2 (version 3.5.2), ggtext (version 0.1.2) , ggstar (version 1.0.4), ggnetwork (version 0.5.13), glmmTMB (version 1.9.19), igraph (version 2.1.4), bipartite (version 2.20), RColorBrewer (version 1.1.3) and philentropy (version 0.10.0) packages.

For manuscripts utilizing custom algorithms or software that are central to the research but not yet described in published literature, software must be made available to editors and reviewers. We strongly encourage code deposition in a community repository (e.g. GitHub). See the Nature Portfolio guidelines for submitting code & software for further information.

Data

Policy information about availability of data

All manuscripts must include a data availability statement. This statement should provide the following information, where applicable:

- Accession codes, unique identifiers, or web links for publicly available datasets
- A description of any restrictions on data availability
- For clinical datasets or third party data, please ensure that the statement adheres to our policy

DNA sequence data in this study have been deposited in the DNA Data Bank of Japan database under accession numbers PRJDB17520 (Fungi and plant; https://ddbj.nig.ac.jp/search/entry/bioproject/PRJDB17520) and PRJDB37499 (Prokaryote; https://ddbj.nig.ac.jp/search/entry/bioproject/PRJDB37499) . This study also used a publicly root microbiome dataset (Supplementary Data 1 and Supplementary Data 2 in the original publication) collected across Europe. These data are available in the original publication and the Zenodo open data repository under DOI: 10.5281/zenodo.10203817. Taxonomy assignment was performed using the UNITE general FASTA release for eukaryotes 2. Version 18.07.2023 and the SILVA version 138 databases. Source are provided with this paper.

Research involving human participants, their data, or biological material

Policy information about studies with human participants or human data. See also policy information about sex, gender (identity/presentation), and sexual orientation and race, ethnicity and racism.

Reporting on sex and gender	This study did not involve human participants, their data, or biological materials.
Reporting on race, ethnicity, or other socially relevant groupings	This study did not involve human participants, their data, or biological materials.
Population characteristics	This study did not involve human participants, their data, or biological materials.
Recruitment	This study did not involve human participants, their data, or biological materials.
Ethics oversight	This study did not involve human participants, their data, or biological materials.

Note that full information on the approval of the study protocol must also be provided in the manuscript.

Field-specific reporting

Please select the one below that is the best fit for your research. If you are not sure, read the appropriate sections before making your selection.

☐ Life sciences ☐ Behavioural & social sciences ☒ Ecological, evolutionary & environmental sciences

For a reference copy of the document with all sections, see nature.com/documents/nr-reporting-summary-flat.pdf

Ecological, evolutionary & environmental sciences study design

All studies must disclose on these points even when the disclosure is negative.

Study description	This study aimed to characterize the assembly landscapes of root-associated microbiomes in a cool-temperate forest in central Japan. An observational, non-manipulative, cross-sectional study design was used. Root-tip samples were destructively collected once at 125 sampling points within a natural forest stand. The analyses focused on major six host-plants (Acer, Betula, Juglans, Larix, Pinus, and Populus). No experimental treatments were applied. Fungal ITS and prokaryotic 16S rRNA amplicon data obtained from the root-tip samples were integrated with host-plant information. Energy landscape analysis was then used to infer the assembly landscapes of fungal and prokaryotic communities and to identify potential keystone taxa associated with community assembly.
Research sample	Root-tip samples (1-cm terminal roots) were collected for six dominant tree genera (Acer, Betula, Juglans, Larix, Pinus, and Populus) in a natural cool-temperate forest at the Sugadaira Research Station, University of Tsukuba, Nagano, Japan (36.524°N, 138.349°E, 1,340 m a.s.l.). Both fungal and prokaryotic communities were analyzed using amplicon sequencing of the ITS and 16S rRNA regions. The fungal dataset was previously described in our earlier publication (https://doi.org/10.1186/s40793-024-00628-8).
Sampling strategy	Sampling points (n = 125) were randomly established across the forest, with a minimum distance of 4 m between adjacent points, to capture spatial variation in root-associated microbial communities across coexisting tree species. A total of 1,270 root-tip samples were collected from these sampling points, with more than 50 samples per focal host species, to capture variation within sampling points and among host species.<br><br>No a priori statistical power analysis was performed, as no standard power-analysis framework exists for assembly landscape reconstruction. Instead, sample-size sufficiency was evaluated using benchmark simulations (Supplementary Fig. S50c; Supplementary Note 1), which showed that the reconstructed assembly landscape topography, assessed based on basin frequencies, stabilized (Jensen–Shannon divergence < 0.06) once the number of community samples reached approximately 1,200 across a range of community complexities and interaction structures. The final dataset of 1,270 root-tip samples therefore exceeded this

	benchmark. Root samples yielding fewer than 1,000 reads were excluded. Sampling coverage was standardized using a coverage-based rarefaction method implemented in the R package vegan.
Data collection	MN and HT collected root samples at 125 points in a natural cool-temperate forest at the Sugadaira Research Station, University of Tsukuba, Nagano, Japan (36.524°N, 138.349°E, 1,340 m a.s.l.). MN conducted DNA metabarcoding for the fungal ITS and prokaryotic 16S rRNA regions. One-centimeter root tips were collected, surface sterilized, and subjected to DNA extraction and purification using the CTAB method, followed by PCR amplification of target regions. MiSeq sequencing data were quality-filtered, and operational taxonomic units (OTUs; 97% similarity threshold) were assigned to taxonomic groups.
Timing and spatial scale	The study covered an approximately 8.5-hectare forest (125 sampling points). Sampling was conducted as a single campaign from July 27 to August 5, 2022.
Data exclusions	Root samples with fewer than 1,000 sequencing reads were excluded from both fungal and prokaryotic datasets. Plant genera with fewer than 50 qualified root samples were not included. In the energy landscape analysis, samples in which more than 50% of reads could not be annotated at the family level were excluded.
Reproducibility	The outputs of the energy landscape analysis and the sensitivity analyses for identifying keystone taxa were highly reproducible, showing minimal variation when simulations with randomly generated initial community states were repeated 30 times. We also confirmed their robustness using bootstrap resampling procedures.
Randomization	In the energy landscape analysis, host plant identity was included as a covariate. To z-standardize the Δtopography and Δevenness indices, null distributions were generated using two distinct randomization schemes (10,000 permutations each). In the first scheme (host-restricted randomization), relative abundance values were randomly shuffled within each host-plant category across all samples. In the second scheme (host- and block-restricted randomization), relative abundance values were randomly shuffled only among samples belonging to the same host species and collected within the same spatial block.
Blinding	Blinding was not applicable, as the study involved no experimental manipulation or treatment groups. All analyses were based on computational simulations or modeling of observational microbial community data.

Did the study involve field work? ☒ Yes ☐ No

Field work, collection and transport

Field conditions	Fieldwork was conducted under natural environmental conditions without any experimental manipulation. For example, a previous study reported the soil nitrogen concentrations (Urakawa et al., 2015; measurements at 0–10 cm soil depth for five sampling points: NH4+, = 2.426 mgN per kg soil; NO3− = 0.439 mgN per kg soil). Root samples were collected from July 27 to August 5, 2022. Sampling was performed under non-rainy conditions to minimize variation in soil moisture and contamination risk.
Location	Field sampling was carried out in the research forest of Sugadaira Research Station, Mountain Science Center, University of Tsukuba, Sugadaira, Ueda, Nagano Prefecture, Japan (36.524 ºN; 138.349 ºE; 1340 m a.s.l.)
Access & import/export	All field collections were conducted with permission from Sugadaira Research Station, Mountain Science Center, University of Tsukuba.<br>No international transport of biological materials occurred; all samples were collected and processed domestically in Japan.
Disturbance	Sampling was designed to minimize ecological disturbance. At each sampling point, ca. 30 cm2 soil was excavated from a depth of 0 to 20 cm in order to collect woody plant roots. All sampling sites were restored to their original state immediately after collection.

Reporting for specific materials, systems and methods

We require information from authors about some types of materials, experimental systems and methods used in many studies. Here, indicate whether each material, system or method listed is relevant to your study. If you are not sure if a list item applies to your research, read the appropriate section before selecting a response.

Materials & experimental systems

n/a	Involved in the study	
☒	☐	Antibodies
☒	☐	Eukaryotic cell lines
☒	☐	Palaeontology and archaeology
☒	☐	Animals and other organisms
☒	☐	Clinical data
☒	☐	Dual use research of concern
☒	☐	Plants

Methods

n/a	Involved in the study	
☒	☐	ChIP-seq
☒	☐	Flow cytometry
☒	☐	MRI-based neuroimaging

Plants

Seed stocks	We did not use any seed stocks.
Novel plant genotypes	We did not produce any new plant genotypes.
Authentication	We did not use any seed stocks. We did not produce any new plant genotypes.
