# Supplementary material for: Statistical inference of keystone taxa reshaping the assembly rules of forest root microbiomes
Source: Nat Commun. 2026 Oct 28;17:10997. doi: 10.1038/s41467-026-78401-3 (PMC13716906; doi:10.1038/s41467-026-78401-3)
Supplement: Supplementary file 4 [file 41467_2026_78401_MOESM5_ESM.pdf]

nature portfolio

Peer Review File

Statistical inference of keystone taxa reshaping the assembly rules of forest root microbiomes

Corresponding Author: Professor Hirokazu Toju

This file contains all reviewer reports in order by version, followed by all author rebuttals in order by version.

Version 0:

Reviewer comments:

Reviewer #1

(Remarks to the Author)
Summary significance

This study applies a maximum-entropy, Ising-type model to infer assembly landscapes for root fungal prokaryotic communities from 1,270 root-tip samples across multiple host tree species. Steepest-descent simulations on the inferred energy function are used to define basins basin bottoms. You then introduce two indices, Δtopography (changes in which basins community states flow into) Δevenness (changes in the entropy of basin frequencies), to quantify how changes in the abundance of a focal genus reorganize the assembly landscape. This framework highlights several endophytic fungi some bacterial genera as putative "organizers" of community structure suggests possible cross-kingdom influences. The work is timely, the presentation is generally clear, the idea of an "assembly landscape" is likely to attract interest beyond the specific system studied here.

Major comments

1) Taxonomic-level mismatch information loss
The assembly landscapes are built from family-level presence/absence matrices, whereas the sensitivity analysis manipulates genus-level relative abundances removes the focal genus from the community matrix. This creates a conceptual statistical mismatch between the representation of the community state (family-level binary) the perturbation (genus-level abundance). Please clarify in detail how you implement the exclusion of a focal genus when multiple genera belong to the same family: how is family presence defined after genus removal, how sensitive are the inferred basins to alternative rules? In addition, please justify the family-level aggregation binarization by providing robustness checks, for example by:
(i) constructing landscapes at a finer taxonomic resolution (e.g. genus level) for a reduced subset of taxa,
(ii) comparing pure presence/absence landscapes to those built from simple abundance-thresholded or compositional encodings.

2) Confounding by microenvironment, spatial structure, technical factors
Host-plant identity is included as a covariate, but other potential confounders (soil chemistry, microclimate, spatial coordinates, sampling plot, sequencing batch) are not explicitly modeled. Because your main conclusions attribute landscape reorganization to particular taxa, it is important to ensure that these effects are not simply capturing unmodeled environmental or technical gradients. Please report which environmental technical metadata are available and, where possible, include them as explicit variables in the energy model or show that your results are robust to their inclusion. At minimum, consider spatial blocking or within-plot shuffle null models to address spatial autocorrelation beyond host-stratified permutations.

3) Model fit out-of-sample performance
The manuscript would benefit from more explicit evaluation of model fit predictive performance. In particular, it would be useful to:
(i) report held-out or cross-validated likelihoods, or accuracy of predicting basin assignments for unseen samples,
(ii) demonstrate that basin bottoms disconnectivity graphs are stable under resampling (bootstrapping of samples) under different numbers of initial states used in the steepest-descent simulations.

This would help readers judge how robust the inferred assembly landscapes are to sampling noise the choice of simulation settings.

4) Definition statistical testing of Δtopography Δevenness
The Δ indices are defined by comparing landscapes constructed under a “low” a “high” abundance setting for the focal genus (e.g. 0% vs 50% quantile, with some additional analyses using 25%/75%). The choice of quantiles thresholds should be justified more fully, effect sizes should be accompanied by measures of uncertainty, for example bootstrap confidence intervals for Δtopography Δevenness. Please also clarify the scope of multiple-testing correction (e.g. whether FDR is controlled across all genera hosts jointly, or within each host). Given the large number of tests, a careful definition of the FDR control procedure is important.

5) Quantifying uncertainty in basin energies topography
Figures showing basin bottoms disconnectivity graphs provide an appealing qualitative view of the assembly landscapes, but they do not convey uncertainty. Please consider summarizing uncertainty in basin energies in the heights of disconnectivity barriers, for example by bootstrap resampling of samples or by perturbing fitted parameters within their estimated uncertainty range. Even if only approximate, such uncertainty quantification would help readers gauge the robustness of the inferred landscape structure.

6) Cross-kingdom effects
Some of the most interesting claims concern fungal genera that appear to reorganize prokaryotic assembly landscapes, vice versa. These cross-kingdom effects are provocative biologically plausible, but difficult to distinguish from shared responses to underlying environmental gradients. Where feasible, please provide additional analyses to probe directionality, for instance by including fungal abundance alongside environmental covariates in models of the prokaryotic landscape, or by performing partial correlation / partial regression analyses. If such analyses are not possible with the available data, I would suggest softening the language to emphasize that the observed patterns are consistent with, but do not prove, cross-kingdom causal influence.

7) Fundamental limitations of the energy-landscape framework under asymmetric interactions
A more fundamental theoretical issue concerns the applicability of an energy-landscape (free-energy / Lyapunov potential) framework to real microbial communities. The statistical-physics concept of an energy landscape is strictly compatible with systems whose effective interactions are symmetric (or more generally whose dynamics admit a global Lyapunov function), in which case the system can be viewed as moving downhill on a potential surface. However, microbial interaction networks are in general asymmetric: competitive, exploitative, inhibitory facilitative interactions need not satisfy J_ij = J_ji, generalized Lotka–Volterra–type models with asymmetric interaction matrices typically do not possess a global Lyapunov function. In such systems, the dynamics can exhibit non-equilibrium attractors, including limit cycles, quasiperiodic or chaotic dynamics, noise-sustained oscillations, rather than convergence to fixed-point attractors that can be neatly organized into basins of a scalar potential.
I think the manuscript would benefit from an explicit discussion of how your “assembly landscapes” should be interpreted in this more general, non-gradient setting. Are they best viewed as effective probabilistic summaries of occupancy patterns, valid even when the microscopic dynamics are non-gradient support non-equilibrium attractors? Have you explored, for example in simulations, how your Δtopography Δevenness metrics behave when applied to data generated by systems with strongly asymmetric interactions non-equilibrium dynamics? At minimum, I suggest adding a dedicated paragraph in the Discussion that acknowledges this limitation of the energy-landscape framework, clarifies that the inferred “energy” is an effective statistical potential rather than a proven Lyapunov function of the underlying ecological dynamics, explains what aspects of real community dynamics can cannot be captured within this framework.

Minor comments
- Clarify the exact definition of “presence” in the presence/absence matrices (e.g. any non-zero read count after rarefaction, or a specific minimum read threshold).
- Provide more detail on the permutation/null-model schemes used to assess the significance of Δtopography Δevenness, including which properties of the data they preserve (host identity, community richness, library size, etc.).

(Remarks on code availability)

Reviewer #2

(Remarks to the Author)
This is a very well written manuscript that describes a novel approach to identify keystone taxa using an approach inspired by statistical physics. The approach is tested on real sequencing data from microbial communities associated with ectomycorrhizal dual-mycorihizal plants. Importantly, capabilities potential ecological roles of identified keystone taxa are discussed in detail, revealing that the their inferred status as keystone taxa is biologically meaningful, thereby validating the approach. The study provides new insights is highly relevant to the understanding of characteristics identification of keystone taxa. The manuscript comprises careful detailed analyses, all conclusions are clearly derived from the presented data. However, I think that the structure the clariyt of the manuscript can be improved (see comments below).
I struggle with understanding the theoretical framework. This is not necessarily due to a weakness of the paper, but may rather reflect that statistical physics is not within my area of expertise.

General comments:
i) The authors make use of the term 'assembly rules' numerous places in the text (including the title). But it is not clearly explained what an assembly rule is or can be. what is the relationship between assembly rules assembly landscapes? Is the stability of a community only dependent on the assembly landscape or a combination of the assembly landscape assembly rules?

ii) I think the 'flow' of the paper can be improved by being more clear about the main motivation behind the paper. Is it to better understand the role of various groups of fungi bacteria in plant root systems or is it to develop a method for the identification of keystone taxa? In the first case it would seem natural to start the introduction with fungi bacteria (which is done), but also start the results section with the sequencing data then move on to identifying keystone taxa. In the second case it would seem natural that the first sentence in the intro is about the problem of defining identifying keystone taxa (using fungi bacteria in root systems more as an example/illustration of the problem a good system for method validation). Then, in the result section, describe method development test the method with real sequencing data for validation (which is also a matter for the discussion).

iii) In my view the results include sections that seem to fit better in the introduction: See for example L97-104 which describe a motivation for doing the study, but not results. L105-113 is also more about motivation the authors continue to refer to Fig1 (explained in the Intro). L114 onwards sounds more like results, but it is a bit confusing that the authors refer to Fig1a-right, which seems to be an illustration not actual results.
From L116 the authors explain what an anergy landscape analysis is how it has been used previously, which I also think would fit better in the introduction. L119-136 could also fit better in intro methods, I think. L137-138 describes a result. But L138-147 describes "strategy" asks the reader to "imagine" things, which are not results.
Here are some suggestion: Keep Fig1 out of the intro. Rephrase a subtitle in the results (L95) into something like 'developing a method for identification of assembly landscapes detection of possible keystone taxa'. Under this section focus on objectively explaining how the method works (Fig 1) (not describing results of applying the method), then (as is done now) show the results of applying the method on your amplicon datasets.

iv) It would be nice if the authors could add more detailed information about the workflow in the README file in the github repository (https://github.com/ngchngch/Assembly_landscape_of_the_forest_root_microbiome) to make the method code more accessible.

Minor comments:

L66-70: "Although directly reconstructing the architecture of stability landscapes (Fig. 1a-left) from empirical data". But it is Fig1a-right that shows the empirical assembly landscape - Should it be "Fig. 1a-right [...] Fig. 1a-left"?

L245-247: should it be 'were inferred to exert'?

L474: What defines the presence or absence of taxon i? From Fig 1f absence does not seem to a absence of reads, but rather based on a threshold (presumably based on relative abundance not absolute number of reads).

Is CLR center log ratio? I don't think CLR is defined in the text.

It would be instructing to see an illustration of the difference between a keystone-taxon a non-keystone-taxon among the main figures - either as an extension of Fig1 or as a separate figure. At least indicate in the legend of Fig1 how Fig1d-g can be used to infer that the target taxon is a keystone taxon.

(Remarks on code availability)
The repository contains a README file, but details on how to use the code (e.g. example workflow) is lacking.

Reviewer #3

(Remarks to the Author)
The authors proposed an computational/statistical analysis method based on the so-called energy landscape analysis based on the inverse Ising model. This method has been studied in particular for functional magnetic resonance imaging (fMRI) data from the brain more recently in various ecological/microbiological data such as the present one. The present authors introduced two metrics to the Ising-based energy landscape analysis, "\Delta topography" "\Delta evenness", to measure the impact of the change in the abundance of a species on the shape of the energy landscape inferred from data. In this way, they proposed to quantify the "keystone-ness" of individual species as the size of "\Delta topography" "\Delta evenness". They applied the methods to microbial abundance data they collected from a Japanese forest.

The paper is vaguely written in various methodological aspects, which confused me a lot (see below). Putting this issue

aside, my main criticism is that there is no biological ground truth data analyzed to validate outcomes of the energy landscape analysis (called assembly landscapes in the manuscripts). In other words, they suggested some keystone species (and ran other analyses) based on their method, but there is no way to tell whether the inferred keystone species are really keystone species in some biological or ecological sense. Therefore, one cannot tell whether the results are practically useful. On this ground, I do not recommend the publication of this article in Nature Communications. See below for specific feedback.

(1) Taking neuroscience as example (because this sort of analysis has been used a lot there), once one infers energy landscapes from fMRI data, the results are probably publishable but the impact of the work is weak. The reason is that we cannot then tell whether products of the energy landscape analysis are useful. This is why almost all papers using this analysis method (or other relatively similar methods) published in high tier journals (even journals that neuroscientists (including computational neuroscientists) consider substantially below Nature Communications) do some "ground truth analysis", which for example means that some quantity obtained from energy landscape analysis is correlated with some non-fMRI quantity measured from the participants in the experiment (e.g., severity of the disease, score of the cognitive task). I do not work on microbial biology, so I cannot tell the difference between the two fields; but I apply the same criterion here. What are *biological/ecological* knowledge obtained by this work? How is the present method obtained/proposed biological/ecological knowledge validated? These are entirely lacking in the present manuscript. This is related to the fact that this manuscript is full of "be inferred", "be estimated", "could govern", etc., says few things decisively.

(2) The presentation of the methods is poor, confusing readers (at least me). I understand that details of the methods need to sit in the Methods section that appears after the Discussion/Conclusion section. However, the Results section should read smoothly, even without precise definitions of the quantities used. Furthermore, at least in the Results section, several terms are undefined/unexplained, which practically confused me. Introduction materials are explained in the beginning of the Results section. All these make reading understanding of the results harder. Specifically,

(2-1) lines 97-113: These two paragraphs are Intro (i.e., background) materials. I also note that if we move these two paragraphs to the Introduction section, the Introduction section is a bit long. In a related vein, in lines ~65-80, What is the key question in this paper? The new methods are developed because the authors want to find something biological. I suggest reformulating the Introduction section completely to deliver the key questions knowledge gaps more clearly, no matter the journal.

(2-2) line 123: "each step". "step" means what?

(2-3) line ~123: I do not even understand what the node is by reading the text around here. It is also crucial to mention that each species abundance (?) is binarized for this analysis. The authors should not have too much methodological details in the Results section of this type of journal, indeed, but a minimum is necessary for readers to be able to read through.

(2-4) lines 143-145: I do not understand the idea of external variables (including later in the Results section), despite that changing the external variable running the same analysis to get different results seems to be a key development later in this Results section.

(2-5) lines 152-153: What are "community-state probabilities"? In fact, the authors might better not to explain all terms in the Results section. Anyways, the methodological explanation in the Results section should be concise enough also good enough so that hasty readers who would skip reading the Methods section can understand the results grossly. I think rework is needed to reach that standard.

(2-6) lines 220-236: No need to repeat the methods explained above.

(2-7) lines 257, 259: "organizer" means what?

[other minor]

(3) line 118: "nerval ignition patterns" --> "brain activity patterns", "neuronal activity patterns", something like that? fMRI does not measure spiking/firing of neurons (which the authors meant by ignition?) directly.

(4) line 119: "When applied to community ecology". This sentence should be reformulated because "the framework employs ..." is also true when applied to any other areas.

(5) lines 155-157: This stabilizing effect holds true only for negative "\Delta evenness" values, not for low but positive values.

(6) lines 196-203: The results depend on the host-plant background. What do we learn from this result?

(Remarks on code availability)

Reviewer #4

(Remarks to the Author)
In this manuscript, the authors conduct a comprehensive analysis of the assembly landscape of forest root microbiomes,

examining both fungal prokaryotic communities. By treating the relative abundance of a focal taxon as an environmental variable, they define its “keystoneness” based on the extent to which changes in that taxon’s abundance alter the inferred assembly landscape. The authors apply this framework to a well-collected forest root microbiome dataset, the results figures are clearly presented. However, the method lacks validation using in silico simulations (e.g., generalized Lotak-Volterra model or others), several methodological aspects remain unclear. Therefore, I have several comments concerns as listed below.

Major comments
1. The effectiveness ecological relevance of the statistical energy-landscape framework are not sufficiently demonstrated. The method needs to be validated using in silico simulations based on mechanistic models, such as gLV or consumer-resource models. It remains unclear how accurately the approach can identify basins of attraction, the extent to which its outputs correspond to underlying community stability properties, how sensitive the results are to data sparsity (i.e. sample size vs. ecosystem complexity).
2. The authors present the taxonomic compositions of fungal prokaryotic communities across all root-tip samples; however, the manuscript lacks PCoA analysis that compares these compositional patterns with the basins of attraction inferred from the statistical energy-landscape model. As the statistical model relies on presence/absence data, it is unclear whether samples assigned to the same basin truly share similar community composition profiles. For example, do samples from different plants that fall into the same basin exhibit similar taxonomic profiles?
3. The ecological interpretation of the “keystone taxa” identified by this statistical framework is not well articulated. Several key studies that define characterize keystone species or keystone taxa in microbial communities (e.g., Amit & Bashan 2023; Wang et al. 2024) are not cited or discussed, leaving the reader without a clear comparison between this framework existing ecological definitions or methodological approaches. Integrating these perspectives is important to clarify whether the taxa identified by the energy-landscape method align with established ecological concepts of keystone effects. The author should benchmark the method against simulated community dynamics or providing empirical evidence that the identified taxa exert biologically meaningful influence within root microbiomes. Thus, both conceptual grounding validation are needed to strengthen the interpretation practical utility of the identified keystone taxa.

Other comments:
1. The authors use the term “50% abundance-quantile” in multiple places in the manuscript. This terminology may confuse readers without a statistical background, as it could be misinterpreted as “50% relative abundance” rather than the median relative abundance across samples. Consider replacing “50% abundance-quantile” with “median abundance” throughout the manuscript for clarity.
2. The authors identify keystone taxa by removing the corresponding genus from the dataset. It remains unclear whether removing a genus that is a major contributor to its family could introduce bias into the analysis.
3. The authors analyze keystoneness only for genera detected in more than 100 root-tip samples (Line 499). It is unclear whether the energy landscape analysis includes samples where the target genus is absent (0 abundance) or only those where it is present. Extending the inferred keystoneness to 0 abundance may introduce bias, particularly if absent samples were not included in the analysis. Moreover, the prevalence overall abundance of a genus could influence its keystoneness. The authors should evaluate whether taxa are identified as keystone simply because they are highly prevalent or abundant, rather than due to genuine ecological influence.
4. The authors do not sufficiently explore or hypothesize about the mechanistic biological drivers that could underlie the inferred basins keystone taxa. Linking the statistical keystones to ecological functon(e.g., nutrient cycling, plant fitness, stability) would make the findings more biologically meaningful; some explicit hypotheses or suggestions for experimental validation would strengthen the ecological relevance of the study.
5. The authors should expand the analysis discussion on cross-kingdom interactions (i.e. fungi-bacteria interactions).

(Remarks on code availability)

Version 1:

Reviewer comments:

Reviewer #1

(Remarks to the Author)
I thank the authors for their careful, detailed, comprehensive response to my comments. They have addressed all of my concerns satisfactorily through substantial revisions, additional analyses, clearer methodological explanations, improved presentation of the manuscript. As a result, the quality, clarity, overall rigor of the paper have been greatly improved. I am satisfied with the revised version have no further substantial comments.

(Remarks on code availability)

Reviewer #2

(Remarks to the Author)
I appreciate the authors’ careful detailed responses to my comments. The revisions have substantially improved the clarity, structure, presentation of the manuscript, including a clearer explanation of the theoretical framework. All of my concerns

have been adequately addressed, I am happy to recommend acceptance of the manuscript without further changes.

(Remarks on code availability)

Reviewer #4

(Remarks to the Author)
The authors have incorporated substantial new analyses in the revised manuscript, including validation with the gLV model additional analyses using independent datasets. These efforts further broaden the scope of the study have the potential to deepen our understanding of the proposed energy landscape framework the keystone concept introduced by the authors. However, the newly added results are not sufficiently integrated with the original narrative of the manuscript are only briefly described. More importantly, the implementation of the gLV model appear to be potentially problematic requires further clarification from the authors. Please see the detailed comments below.

1. The authors conducted in silico simulations based on generalized Lotka–Volterra (gLV) model. As shown in Supplementary Fig. S6, the model appears to operate in a chaotic regime, which may result from the introduction of random noise. This differs from some previous studies that assume microbial communities typically reside near a steady state. In addition, the authors statistically infer community assembly patterns by sampling the system at random time points during the simulations. However, if the concept of a community basin is intended to correspond to a steady-state attractor, it is unclear why random sampling from a chaotic trajectory would be appropriate for predicting the basin structure. The authors should clarify the rationale for this approach its ecological interpretation.
Using the data generated by the gLV model, the authors also benchmarked the number of identified basins (Supplementary Fig. S48a). However, this was not compared with the number of attractors that the underlying gLV system may possess. For instance, how many potential basins (attractors) would emerge if community compositions were randomly initialized in the gLV simulations? Furthermore, how do properties of the interaction network, such as connectivity or mean interaction strength, influence the number of basins in the system? Clarifying these points would help relate the statistically inferred basins to the ecological dynamics implied by the model provide a clearer ecological interpretation of the framework.

2. The authors have added a discussion of the broader literature on keystone species keystone taxa in microbial communities in the Discussion section. However, I still feel that the term “keystone” should be used with caution. Traditionally, a keystone species refers to a species whose removal has a disproportionately large impact on community structure or ecosystem function. If the authors intend to identify keystone taxa in this traditional sense using Δ topography Δ evenness derived from the assembly landscape framework, they should provide evidence that the taxa identified by their method indeed exert such ecological impacts. Alternatively, if the authors intend to extend or reinterpret the traditional definition of “keystone” within the context of the assembly landscape framework, the rationale necessity for this conceptual shift should be clearly explained. This point should be clarified in the Introduction to better illustrate the authors’ motivation for developing this framework.

3. The authors added PCoA analyses for the fungal prokaryotic community datasets based on family-level compositions using Bray–Curtis Jaccard dissimilarities (Supplementary Fig. S12). However, these results are not sufficiently integrated into the main text. Although Fig. S12 is cited at lines 157 181, no explanation or interpretation is provided. Consequently, it is unclear how this supplementary analysis supports or strengthens the main arguments of the manuscript. The authors should clarify whether the basins defined in their statistical model are intended to correspond, at least conceptually, to the ecological assembly patterns reflected in the PCoA ordination. Clarifying this relationship would help bridge the statistical framework the ecological interpretation of the results.

4. The authors state that “Notably, genera showing relatively strong effects on these indices were not necessarily those that were highly prevalent or abundant in the root microbiome dataset (Supplementary Figs. S15–S18)” (lines 201–203). However, inspection of Figs. S15b S16b suggests that there may be some degree of association between the abundance or occupancy of prokaryotic taxa the assembly landscape metrics (Δ topography Δ evenness). While formal statistical testing would be required to evaluate this relationship, the current evidence does not appear sufficient to conclude that these variables are unrelated. The authors should clarify this point or provide additional analyses to support their statement.

5. The section headings in the Results are largely descriptive do not clearly highlight the main conclusions of each section. The authors may consider revising some of the section titles to better reflect the main findings, which would help readers get the key results more quickly.

6. In the Figure 1 panel g, please use median abundance instead of 50% abundance-quantile.

(Remarks on code availability)

Version 2:

Reviewer comments:

Reviewer #4

(Remarks to the Author)
I appreciate the authors'careful detailed responses to my comments. The revisions, including the additional analyses, clearer methodological explanations, improved presentation, have substantially enhanced the quality, clarity, overall rigor of the manuscript. I am satisfied with the revised version have no further substantive comments. I believe the manuscript is now suitable for publication.

(Remarks on code availability)

Open Access This Peer Review File is licensed under a Creative Commons Attribution 4.0 International License, which permits use, sharing, adaptation, distribution and reproduction in any medium or format, as long as you give appropriate credit to the original author(s) and the source, provide a link to the Creative Commons license, and indicate if changes were made.
In cases where reviewers are anonymous, credit should be given to 'Anonymous Referee' and the source.
The images or other third party material in this Peer Review File are included in the article’s Creative Commons license, unless indicated otherwise in a credit line to the material. If material is not included in the article’s Creative Commons license and your intended use is not permitted by statutory regulation or exceeds the permitted use, you will need to obtain permission directly from the copyright holder.
To view a copy of this license, visit https://creativecommons.org/licenses/by/4.0/

RESPONSES TO REVIEWER COMMENTS

*** Reviewer comments are followed by our RESPONSEs. Line numbers in the revised manuscript are indicated for each revised point. ***

-- Reviewer 1--

Summary and significance

This study applies a maximum-entropy, Ising-type model to infer assembly landscapes for root fungal and prokaryotic communities from 1,270 root-tip samples across multiple host tree species. Steepest-descent simulations on the inferred energy function are used to define basins and basin bottoms. You then introduce two indices, Δtopography (changes in which basins community states flow into) and Δevenness (changes in the entropy of basin frequencies), to quantify how changes in the abundance of a focal genus reorganize the assembly landscape. This framework highlights several endophytic fungi and some bacterial genera as putative "organizers" of community structure and suggests possible cross-kingdom influences. The work is timely, the presentation is generally clear, and the idea of an "assembly landscape" is likely to attract interest beyond the specific system studied here.

[RESPONSE] Thank you very much for the constructive and encouraging evaluation of the study. In accordance with the following suggestions, we revised the manuscript to clarify the methodological framework and to more explicitly demonstrate the robustness and interpretability of the assembly landscape approach as detailed in the following response to each comment.

Major comments

1) Taxonomic-level mismatch and information loss

The assembly landscapes are built from family-level presence/absence matrices, whereas the sensitivity analysis manipulates genus-level relative abundances and removes the focal genus from the community matrix. This creates a conceptual and statistical mismatch between the representation of the community state (family-level binary) and the perturbation (genus-level abundance). Please clarify in detail how you implement the exclusion of a focal genus when multiple genera belong to the same family: how is family presence defined after genus removal, and how sensitive are the inferred basins to alternative rules?

[RESPONSE] To clarify how family-level entries were defined after removing a focal genus in the sensitivity analysis, we revised the Methods section to describe the data reconstruction procedure more explicitly.

Within the sensitivity analysis framework, we sequentially reconstructed the community data matrix by excluding each focal genus. Specifically, OTUs annotated to the focal genus were removed from the dataset. The remaining OTUs were then aggregated at the genus or family level (Fig. 1f and Supplementary Fig. S9b). When multiple genera belonged to the same family, family presence was defined based on the occurrence of any remaining genus within that family after the focal genus was removed. We then binarized the abundance of the taxa targeted in the above energy landscape analysis based on the procedure detailed in Supplementary Figure S9. During this procedure, taxa occurring in 30 or fewer samples were excluded.

The procedure is explained in the revised manuscript as follows:

(lines 523–528 in the main text)

> "Within the sensitivity analysis framework, we sequentially re-defined the community data matrix by excluding each focal genus. Specifically, OTUs annotated to the focal genus were removed, and the remaining OTUs were aggregated at the family-level community compositions (Fig. 1f and Supplementary Fig. S9b). We then binarized the abundance of the taxa targeted in the above energy landscape analysis using the same criteria. During this procedure, taxa occurring in 30 or fewer samples were excluded."

In addition, please justify the family-level aggregation and binarization by providing robustness checks, for example by: (i) constructing landscapes at a finer taxonomic resolution (e.g. genus level) for a reduced subset of taxa, and (ii) comparing pure presence/absence landscapes to those built from simple abundance-thresholded or compositional encodings.

[RESPONSE] Following the reviewer's suggestion (i), we conducted additional analyses of assembly landscape inference using genus-level community matrices (Supplementary Figs. S34–S36 and Tables S8–S9), as well as corresponding keystone evaluation analyses (Supplementary Fig. S38). Overall, the number of major basins and their dominant taxonomic signatures were largely conserved between family- and genus-level reconstructions. In addition, genera identified as candidate keystones at the family level exhibited consistently high *Δtopography* and/or *Δevenness* values in the genus-level landscapes (Supplementary Fig. S38). These results indicate that family-level aggregation does not qualitatively alter the inferred assembly landscape structure or keystone identification (lines 509–514 in the main text; lines 229–274 in the Supplementary Information).

Following the reviewer's suggestion (ii), we also performed additional analyses comparing alternative binarization procedures for community data, namely a method based on a uniform abundance threshold and a method based on each taxon's abundance distribution. We found that the uniform abundance-threshold method could introduce biased community membership information for arbuscular mycorrhizal fungi because these taxa are often systematically underrepresented in amplicon sequencing datasets, as reported previously (Suzuki et al., 2020; https://doi.org/10.1007/s00374-020-01431-6). In contrast, the method based on each taxon's abundance distribution (the method used throughout the main analyses) better preserved the relative representation of these taxa and therefore provided a more reliable basis for assembly landscape inference (Supplementary Fig. S24; Supplementary Tables S6–S7; lines 97–120 in the Supplementary Information).

2) Confounding by microenvironment, spatial structure, and technical factors Host-plant identity is included as a covariate, but other potential confounders (soil chemistry, microclimate, spatial coordinates, sampling plot, sequencing batch) are not explicitly modeled. Because your main conclusions attribute landscape reorganization to particular taxa, it is important to ensure that these effects are not simply capturing unmodeled environmental or technical gradients. Please report which environmental and technical metadata are available and, where possible, include them as explicit variables in the energy model or show that your results are robust to their inclusion. At minimum, consider spatial blocking or within-plot shuffle null models to address spatial autocorrelation beyond host-stratified permutations.

[RESPONSE] The environmental and technical metadata available for the fungal and prokaryotic community datasets are limited. Specifically, soil pH measurements were available for 29 of the 124 sampling points, as reported in Noguchi and Toju (2024). However, because this environmental variable was available only for a subset of samples, the sample size was insufficient to reliably incorporate it as an explicit covariate in the energy landscape analysis. Other environmental variables (e.g., microclimate measurements) and technical batch information were not systematically recorded for all samples in the dataset. Therefore, direct incorporation of these factors into the statistical energy landscape model was not feasible.

To address potential confounding effects associated with spatial structure and unmeasured environmental gradients, we conducted additional keystone-exploration analyses based on permutation-derived null models incorporating spatial blocking. Specifically, we performed *z*-standardization using null models that preserved both host-plant identity and spatial structure while randomizing community states within spatial blocks. The resulting keystoneness estimates were

qualitatively consistent with those obtained under the host-plant-stratified permutations used in the main analyses (Supplementary Figs. S42–S43; lines 296–312 in the Supplementary Information).

3) Model fit and out-of-sample performance. The manuscript would benefit from more explicit evaluation of model fit and predictive performance. In particular, it would be useful to: (i) report held-out or cross-validated likelihoods, or accuracy of predicting basin assignments for unseen samples, and (ii) demonstrate that basin bottoms and disconnectivity graphs are stable under resampling (bootstrapping of samples) and under different numbers of initial states used in the steepest-descent simulations. This would help readers judge how robust the inferred assembly landscapes are to sampling noise and the choice of simulation settings.

[RESPONSE] As suggested in comment (i), we conducted 100-fold cross validation of basin assignments in energy landscape analysis. Basin assignments were highly reproducible across cross-validation folds (Supplementary Fig. S25), indicating that the inferred energy landscape model has substantial predictive power for unseen samples. The results of the cross-validation analysis are described in Supplementary Results as follows.

(lines 137-155 in Supplementary Information)

"**Cross-validation confirms predictive robustness of inferred assembly landscapes**

To evaluate whether the inferred assembly landscapes possess predictive capability beyond the fitted data, we performed 100-fold cross-validation of basin assignments. Basin identities inferred from the model fitted to the full dataset were used as reference classifications (Supplementary Fig. S12). For each cross-validation fold, models were reconstructed using datasets excluding a subset of focal community samples, and basin assignments for the excluded samples were predicted using the refitted models.

Predictive performance was evaluated in two ways: (i) agreement in basin assignments and (ii) similarity of basin-bottom community compositions. Assignment accuracy was quantified as the proportion of samples assigned to the same basins as in the full-dataset analysis, whereas deviations in basin identities were assessed using Jaccard dissimilarity between basin-bottom community memberships (Supplementary Fig. S25).

Across the fungal and prokaryotic datasets, 92.7% and 86.1% of community samples, respectively, were assigned to identical basins as those inferred from the full dataset (Supplementary Fig. S25a). Differences in basin-bottom compositions were also minimal. Across host-plant backgrounds, only a small fraction of samples showed noticeable deviations (Jaccard dissimilarity > 0.1: 7.17% in fungi and 4.74% in prokaryotes; Supplementary Fig. S25b).

Together, these results indicate that the energy landscape framework provides stable and predictive estimates of assembly landscapes for the present root microbiome datasets."

As suggested in the reviewer's comment (ii), we performed a series of bootstrap resampling analyses (1,000 replicates) to evaluate uncertainty in the energy landscape inference. We conducted steepest-descent procedures for basin detection using different numbers of initial states ranging from 100 to 200,000 (Supplementary Fig. S23). The results of the additional analysis are detailed in Supplementary Results as follows:

(lines 122–135 in Supplementary Information)

"**Robustness of basin detection to the number of initial states**

To evaluate whether the number of initial states used in steepest-descent simulations influences the detection of basin bottoms in energy landscape analysis, we examined how basin estimates changed across a wide range of initial-state counts. For each host-plant background in the fungal and prokaryotic community datasets, steepest-descent procedures were conducted using ten different numbers of initial states, ranging from 100 to 200,000 (100, 1,000, 5,000, 10,000, 20,000, 40,000, 70,000, 100,000, 150,000, and 200,000; Supplementary Fig. S23).

In fungal assembly landscapes, the number of detected basins remained consistent across all evaluated initial-state settings for each host-plant background (Supplementary Fig. S23a), indicating that basin detection was robust to the choice of initial-state number. In prokaryotic assembly landscapes, basin numbers converged when 5,000 or more initial states were used (Supplementary Fig. S23b), suggesting that sufficiently large initial-state sampling stabilizes basin detection.

These results confirm that the number of initial states adopted in the main analyses was sufficient to reliably identify basin bottoms in both fungal and prokaryotic assembly landscapes."

The bootstrapping approach further indicated that the compositions of basin bottoms of the inferred assembly landscapes are largely robust to sampling variation (Supplementary Figs. S26, S28–S29, S31–S32). The analysis further indicated that energy differences between basin bottoms and boundary states are preserved across bootstrap replicates in terms of major community compositional shifts between basin groups (i.e., transitions between arbuscular mycorrhizal and ectomycorrhizal states for fungal communities and those among Basin Groups I, II, and III for prokaryotic communities; Supplementary Figs. S27, S30b, and S33b). The methods and results for the bootstrap analysis are detailed in Supplementary Results as follows:

(lines 157–227 in Supplementary Information)

"**Bootstrap analysis on the consistency of inferred basin structures**

To evaluate how sampling variability influences assembly landscape inference, we performed bootstrap resampling analyses (1,000 replicates) for both fungal and prokaryotic datasets. For each bootstrap replicate (i.e., generated by resampling the binarized community-states matrix), the energy landscape inference procedure was repeated. We evaluated robustness of the reconstructed landscape architecture from two complementary perspectives: the compositional consistency of basin bottoms (Supplementary Fig. S26) and the stability of energy differences between community states corresponding to basin bottoms in the original landscape and boundary states identified in bootstrap landscapes (Supplementary Fig. S27).

We first assessed uncertainty in the family-level composition of basin bottoms identified in the reconstructed landscapes (Figs. 2c and 3c). Steepest-descent simulations were performed to identify basin bottoms using an identical set of 150,000 initial states for the assembly landscape inferred from the original dataset and for each bootstrap replicate (Supplementary Fig. S26). Using the same initial states allowed direct comparison of the basin bottom reached from a given initial community state across the original and bootstrap landscapes. For each basin bottom identified in the original landscape, we collected the set of initial states that converged to that basin and then tracked the basin bottoms reached from those same initial states in each bootstrap replicate. For each family, we calculated the proportion of bootstrap outcomes in which the family was present in the corresponding bootstrap-inferred basin bottom. These proportions were summarized as family-wise basin representation scores for each original basin bottom.

In addition to compositional consistency, we evaluated robustness and uncertainty in landscape topography by examining energy differences between community states corresponding to basin bottoms in the original landscape and their corresponding lowest-energy boundary states (Supplementary Fig. S27). Because absolute energy values are comparable only among community states within a given energy landscape, for each bootstrap replicate we recalculated the energy of the community states corresponding to each basin bottom and its corresponding lowest-energy boundary state (defined in the original landscape) under the bootstrap-inferred landscape and calculated the energy difference between them as follows:

$$\text{energy difference} = E_{\text{boundary}} - E_{\text{basin}},$$

where E_{basin} and E_{boundary} denote the energy of the community states corresponding to the basin bottom and its corresponding boundary state, respectively, evaluated under the bootstrap-inferred landscape. A positive value of this energy difference indicates that the relative ordering between basin-bottom states (defined in the original landscape) and their corresponding

boundary states is preserved (Supplementary Fig. S27a). This energy-difference index was calculated for each basin in the original energy landscape across bootstrap replicates, yielding a distribution summarized as a histogram. This framework was further used to examine the consistency of inferred compositional transitions between major basin groups (Supplementary Fig. S27b). Specifically, transitions between arbuscular mycorrhizal and ectomycorrhizal basins (Fig. 2c) were examined for fungal communities, whereas transitions between Group I, II, and III basins (Fig. 3c) were examined for prokaryotic communities.

In the fungal assembly landscapes, the ectomycorrhizal family Russulaceae was consistently detected in ectomycorrhizal basin bottoms across bootstrap replicates (Supplementary Figs. S28–S29; F_B1–B3), indicating high robustness of these basins. The arbuscular mycorrhizal family Glomeraceae was also frequently detected in arbuscular mycorrhizal basins inferred for dual-mycorrhizal host plants, although its mean basin representation score across bootstrap replicates was relatively low (Supplementary Fig. S29; F_B4–B5 and F_B7). Consistent with these compositional patterns, the relative ordering between basin-bottom states defined in the original landscape and their boundary states was largely preserved for ectomycorrhizal basins, with boundary states typically having higher energy than the corresponding community states identified as basin bottoms in the original dataset (Supplementary Fig. S30a). Further analysis focusing on transitions between mycorrhizal types indicated that boundary states separating ectomycorrhizal basin-bottom states in the original landscape from arbuscular mycorrhizal states were detected in all 1,000 bootstrap replicates (Supplementary Fig. S30b). For arbuscular mycorrhizal basins, the relationships between basin-bottom states and inter-basin-group boundaries observed in the original analysis were preserved in 69.1%, 73.2%, and 75.4% of bootstrap replicates for *Populus* (F_B4), *Acer* (F_B4), and *Juglans* (mean of F_B5 and F_B7), respectively (Supplementary Fig. S30b).

In the prokaryotic assembly landscapes, dominant basin-bottom families identified in Figure 3c (e.g., Gemmataceae, Pedosphaeraceae, and Solibacteraceae) were consistently recovered across host-plant backgrounds (Supplementary Figs. S31–S32). Families characterizing Group II and Group III basins, including members of Pseudomonadota (e.g., Sterellaceae) and Actinomycetota (e.g., Microbacteriaceae), also showed stable detection patterns (Supplementary Figs. S31–S32). Regarding the relative energy levels between basin-bottom states defined in the original landscape and boundary states inferred from bootstrap landscapes, some basins exhibited high consistency (e.g., P_B3 in *Populus* and P_B12 in *Juglans*; Supplementary Fig. S33a). For other basin-bottom states, the energy difference relative to the minimum-energy boundary state occasionally reversed compared with the original landscape (Supplementary Fig. S33a). Nevertheless, the relative ordering of basin bottoms and boundary states remained largely preserved across bootstrap replicates for transitions among basin groups (i.e., Group I, Group II, and Group III basins) (Supplementary Fig. S33b).

Overall, taxa with relatively sparse occurrence patterns (e.g., arbuscular mycorrhizal fungi) showed greater sensitivity to sampling variability, whereas topographic relationships between compositionally distinct basin types were comparatively stable across bootstrap replicates. "

4) Definition and statistical testing of Δtopography and Δevenness

The Δ indices are defined by comparing landscapes constructed under a "low" and a "high" abundance setting for the focal genus (e.g. 0% vs 50% quantile, with some additional analyses using 25%/75%). The choice of quantiles and thresholds should be justified more fully, and effect sizes should be accompanied by measures of uncertainty, for example bootstrap confidence intervals for Δtopography and Δevenness. Please also clarify the scope of multiple-testing correction (e.g. whether FDR is controlled across all genera and hosts jointly, or within each host). Given the large number of tests, a careful definition of the FDR control procedure is important.

[RESPONSE] As suggested, performed a series of additional sensitivity analyses based on additional abundance-quantile criteria (25% and 75%). The set of taxa highlighted in the keystone-exploration analysis was largely consistent across the different quantile settings (Supplementary Figs. S40–S41). The reason we chose the 50% quantile (median) for the analyses shown in the main text is that it is less sensitive to extreme values than other possible settings. To make clear this point, we reorganized the Method section as follows:

(lines 529-538 in the main text)

"After estimating the parameters of the energy landscape statistical model (eq. 3), we compared the landscape architecture reconstructed under the absence of the focal genus with that estimated under the assumption of an intermediate abundance level. The absence of each genus was defined as the condition in which its abundance was lower than the binarization threshold, and the mean relative abundance across samples classified as absent was used for the landscape reconstruction under the absence scenario. For the intermediate-abundance scenario, the abundance level was set, by default, to the median abundance (50% quantile) across samples in which the focal genus was detected. This choice was made because alternative settings, such as maximum values, could strongly depend on species-specific abundance distributions, whereas median values are least affected by the presence of outliers."

We also followed the second suggestion. Because evaluating all genera was computationally prohibitive, we focused on representative keystone candidates detected in our analyses: *Cladophialophora* in the fungal communities of *Populus*, *Oidiodendron* in the fungal communities of

Acer, *Candidatus* Udaeobacter in the prokaryotic communities of *Juglans*, and *Meliniomyces* in *Pinus*. Although the analysis was still computationally intensive and required several weeks of computation even on a supercomputer, we eventually completed the keystoneness calculations with bootstrap resampling. For these taxa, we evaluated the uncertainty of z-standardized *Δtopography* and *Δevenness* using bootstrap resampling of samples (100 replicates). The bootstrap distributions showed that the estimated *Δtopography* and *Δevenness* values remained consistently elevated for these focal taxa, indicating that the inferred keystoneness signals are robust to sampling variation. The results are presented in Supplementary Figure S44 in the revised manuscript. Correspondingly, we have added descriptions to Supplementary Results as follows:

(lines 314-332 in Supplementary Information)

"Uncertainty in the calculation of *Δtopography* and *Δevenness*

To assess uncertainty in the keystoneness metrics introduced in this study, we quantified the variability of *z*-standardized *Δtopography* and *Δevenness* values using bootstrap resampling (100 replicates). Because each bootstrap replicate required 10,000 permutations constrained within the six host-plant backgrounds, exhaustive evaluation across all host–genus combinations was computationally prohibitive. We therefore focused on representative focal taxa highlighted in the main text.

Among the examined genera, the inferred effects of *Oidiodendron* and *Cladophialophora* on fungal assembly landscapes showed moderate variation among bootstrap replicates. Mean *Δtopography* values were 7.36 ± 4.24 (mean ± standard deviation) for *Oidiodendron* and 8.56 ± 6.70 for *Cladophialophora* (Supplementary Fig. S44a). In addition, *Cladophialophora* exhibited consistent effects on higher-order landscape structure, with *Δevenness* values of 4.54 ± 2.51 (Supplementary Fig. S44b).

In prokaryotic assembly landscapes, *Candidatus* Udaeobacter and *Candidatus* Solibacter consistently showed high *Δtopography* values together with negative *Δevenness* across bootstrap replicates (Supplementary Fig. S44c–d).

Overall, although uncertainty was present in the absolute magnitudes of the indices, the bootstrap analyses demonstrated that the proposed metrics robustly captured consistent directional effects of focal genera on assembly landscape reorganization."

We also added a description of the scope of multiple-testing correction for *Δtopography* and *Δevenness*, as suggested in the final comment, as follows.

(lines 571-574 in the main text)

"Empirical P-values for the two indices were separately corrected for multiple testing using the Benjamini–Hochberg method[79]. Corrections were applied separately to fungal and prokaryotic assembly landscapes by jointly considering all focal genera across the six host backgrounds."

5) Quantifying uncertainty in basin energies and topography

Figures showing basin bottoms and disconnectivity graphs provide an appealing qualitative view of the assembly landscapes, but they do not convey uncertainty. Please consider summarizing uncertainty in basin energies and in the heights of disconnectivity barriers, for example by bootstrap resampling of samples or by perturbing fitted parameters within their estimated uncertainty range. Even if only approximate, such uncertainty quantification would help readers gauge the robustness of the inferred landscape structure.

[RESPONSE] As noted in our response to comment 3(ii), we conducted bootstrap resampling analyses to evaluate the uncertainty and reliability of basin-energy estimates and assembly landscape topography. Because absolute energy values are comparable only among community states within a given energy landscape, the uncertainty in basin and boundary-state energies cannot be directly represented as error bars on a disconnectivity graph. Therefore, for each bootstrap replicate, we recalculated the energy of the community states corresponding to each basin bottom and its corresponding lowest-energy boundary state (defined in the original landscape) under the bootstrap-inferred landscape and calculated the energy difference between them as follows:

$$\text{energy difference} = E_{\text{boundary}} - E_{\text{basin}},$$

where E_{basin} and E_{boundary} denote the energy of the community states corresponding to the basin bottom and its corresponding boundary state, respectively, evaluated under the bootstrap-inferred landscape (Supplementary Fig. S27). We found that although some local topographic features representing minor shifts between compositionally similar basin bottoms were not consistently retained across bootstrap replicates, the major topographic features representing transitions between basin groups (i.e., arbuscular mycorrhizal and ectomycorrhizal basin groups for fungi and Basin Groups I, II, and III for prokaryotes) were robust to sampling stochasticity (Supplementary Figs. S30, S33; lines 157–227 in Supplementary Information).

6) Cross-kingdom effects

Some of the most interesting claims concern fungal genera that appear to reorganize prokaryotic assembly landscapes, and vice versa. These cross-kingdom effects are provocative and biologically plausible, but difficult to distinguish from shared responses to underlying environmental gradients.

Where feasible, please provide additional analyses to probe directionality, for instance by including fungal abundance alongside environmental covariates in models of the prokaryotic landscape, or by performing partial correlation / partial regression analyses. If such analyses are not possible with the available data, I would suggest softening the language to emphasize that the observed patterns are consistent with, but do not prove, cross-kingdom causal influence.

[RESPONSE] As discussed in our reply to comment (2), because the available environmental metadata for our dataset are limited, we instead accounted for potential effects of unobserved environmental gradients using spatially blocked permutation tests (Supplementary Figs. S42–S43). The set of taxa highlighted as candidate keystones was largely consistent between the two alternative randomization settings in the keystone-exploration analysis.

To further examine whether the inferred cross-kingdom associations could be explained by environmental covariates, we conducted an additional analysis using an independent forest microbiome dataset compiled from multiple sites across Europe (Netherway et al., 2024; https://doi.org/10.1038/s41467-023-44172-4). This dataset included amplicon sequencing data of root-associated prokaryotes and fungi, microscopic measurements of mycorrhizal fungal root colonization, and soil environmental variables. For the candidate keystone genera highlighted in our keystone-exploration analysis of the cool-temperate forest dataset from Japan, we examined their presence in the European dataset. For each candidate genus detected in the European dataset, we constructed generalized linear mixed models (GLMMs) with arbuscular mycorrhizal or ectomycorrhizal root colonization rate as a response variable, including the abundance of the focal microbial genus and soil environmental condition (soil pH or soil moisture) as explanatory variables (lines 280–316, 715–743 in the main text).

This additional analysis indicated that 10 of 13 bacterial taxa for which cross-kingdom impacts on fungal assembly landscapes were estimated in our keystone-exploration framework exhibited significant associations with variation in mycorrhizal fungal root colonization (Fig. 7). Although these analyses do not demonstrate causal interactions, they indicate that the cross-kingdom associations identified by our framework are robust across independent datasets and are not solely attributable to shared environmental gradients.

7) Fundamental limitations of the energy-landscape framework under asymmetric interactions
A more fundamental theoretical issue concerns the applicability of an energy-landscape (free-energy / Lyapunov potential) framework to real microbial communities. The statistical-physics concept of an energy landscape is strictly compatible with systems whose effective interactions are symmetric (or more generally whose dynamics admit a global Lyapunov function), in which case the system can be viewed as moving downhill on a potential surface. However, microbial interaction networks are in

general asymmetric: competitive, exploitative, inhibitory and facilitative interactions need not satisfy J_ij = J_ji, and generalized Lotka-Volterra type models with asymmetric interaction matrices typically do not possess a global Lyapunov function. In such systems, the dynamics can exhibit non-equilibrium attractors, including limit cycles, quasiperiodic or chaotic dynamics, and noise-sustained oscillations, rather than convergence to fixed-point attractors that can be neatly organized into basins of a scalar potential. I think the manuscript would benefit from an explicit discussion of how your "assembly landscapes" should be interpreted in this more general, non-gradient setting. Are they best viewed as effective probabilistic summaries of occupancy patterns, valid even when the microscopic dynamics are non-gradient and support non-equilibrium attractors? Have you explored, for example in simulations, how your Δtopography and Δevenness metrics behave when applied to data generated by systems with strongly asymmetric interactions and non-equilibrium dynamics? At minimum, I suggest adding a dedicated paragraph in the Discussion that acknowledges this limitation of the energy-landscape framework, clarifies that the inferred "energy" is an effective statistical potential rather than a proven Lyapunov function of the underlying ecological dynamics, and explains what aspects of real community dynamics can and cannot be captured within this framework.

[RESPONSE] We first conducted simulation analyses to examine the extent to which our framework can capture community dynamics in situations where asymmetric interactions are present, as noted in the comment. Specifically, we performed simulations based on generalized Lotka–Volterra (gLV) models with randomly generated asymmetric interspecific interaction matrices (lines 670–713 in the main text). Although the simulated dynamics could reflect local properties of the assembly landscape (e.g., basin transitions along simulated trajectories), deriving landscape-level descriptors corresponding to *Δevenness* is less straightforward. We therefore evaluated the applicability of our framework for quantifying changes in the assembly landscape along abundance gradients of microbial taxa by assessing *Δtopography* and by comparing the dissimilarity between community compositions obtained from simulations in the presence versus absence of a focal taxon. The benchmark analyses based on the gLV model indicated that species with higher *Δtopography* values tended to exert stronger influences on simulated community outcomes (Supplementary Figs. S7–S8), suggesting that the proposed metrics capture meaningful shifts in community assembly even under asymmetric ecological interactions.

In addition, we added the following sentences to the Supplementary Information to address potential concerns regarding the application of energy landscape analysis to microbial community dynamics involving asymmetric interactions and to clarify the interpretation of the inferred landscapes.

(lines 367-394 in Supplementary Information)

"**Supplementary Discussion**

Energy landscape inferences for complex microbiomes

In this study, we applied an energy landscape framework to reconstruct probabilistic assembly landscapes of complex root microbiomes from empirical data. In statistical physics, energy landscape formulations (e.g., free energy or Lyapunov potentials) are formally justified for systems with symmetric interactions or, more generally, for dynamics that admit a global Lyapunov function[21]. Microbial communities, however, are typically governed by strongly asymmetric interactions, including predator–prey relationships, antibiotic-mediated inhibition, and metabolic cross-feeding[22,23]. Such asymmetries violate detailed balance and can generate non-equilibrium dynamics such as sustained oscillations or limit cycles[24,25]. Consequently, the assumption that community dynamics follow monotonic descent along a global landscape gradient may not strictly hold for complex microbiomes.

Notably, despite these theoretical limitations, potential-like descriptions of system states have successfully characterized diverse complex systems, including protein folding, brain activity, and ecological communities[26–30]. In ecological applications, energy landscape approaches have captured compositional instability among community states and have successfully predicted observed transitions in community composition[30]. Consistent with this perspective, our simulation-based validation indicated that structural changes in inferred landscape topography correspond directly to shifts in community destinations (Supplementary Figs. S7–S8). These results suggest that the inferred landscapes reflect meaningful statistical constraints governing community assembly outcomes. In this sense, the inferred landscape should be interpreted as a probabilistic summary of community-state distributions rather than a deterministic Lyapunov potential governing ecological dynamics.

Although broader experimental validation incorporating explicit biological mechanisms remains necessary, the present findings suggest that landscape-based frameworks can capture key statistical features of microbiome assembly even under non-equilibrium ecological dynamics. From this viewpoint, the assembly landscape inferred here can be interpreted as a statistical potential that summarizes the probabilistic distribution of microbiome states emerging from asymmetric ecological interactions."

Minor comments

- Clarify the exact definition of "presence" in the presence/absence matrices (e.g. any non-zero read count after rarefaction, or a specific minimum read threshold).

[RESPONSE] We added explanations of the data-filtering process to the main text and Supplementary Methods to clarify the definitions of "presence" and "absence" in our framework, as follows:

(lines 495-501 in the main text)

" The statistical framework of energy landscape analysis was applied to the family-level compositional data of fungal and prokaryotic communities, respectively, after filtering the raw data with abundance-based binarization thresholds optimized for this dataset (Supplementary Figs. S9–11, S24, and Tables S4–S7; see Supplementary Methods and Supplementary Results for details). The presence/absence matrices comprising 34 fungal families and that comprising 45 prokaryotic families were respectively subjected to energy landscape analysis, in which host plants were included as categorical (dummy) explanatory variables, using the "rELA" package[14] v0.60 of R 4.4.1[78].."

(lines 74-79 in Supplementary Information)

"After excluding five fungal samples and three prokaryotic samples in which more than 50% of sequence reads could not be annotated at the family level, the community data matrices were converted into binary presence/absence format. The binarization threshold for each taxon *i* was defined as

$$\mathrm{Mean}(\log(X_i)) - 2\ \mathrm{SD}(\log(X_i)),$$

where X_i denotes the read counts of taxon *i* across samples in which the taxon was detected."

- Provide more detail on the permutation/null-model schemes used to assess the significance of Δtopography and Δevenness, including which properties of the data they preserve (host identity, community richness, library size, etc.).

[RESPONSE] As suggested, we added a description of the permutation procedures to clarify that one approach restricts permutations within host plants, thereby preserving host identity only (line 559-563 in the main text), whereas the other restricts permutations within host and spatial blocks, thereby preserving both host identity and spatial structure (line 581-584 in the main text).

(lines 559-563 in the main text)

"To standardize the estimated species' effects on assembly landscape and evaluate their statistical significance, we performed null-model simulation tests. Specifically, we repeated the sensitivity analysis described above, but with randomly shuffled relative abundance of the focal genus using two different approaches (10,000 permutations each). In the randomization, shuffling was performed among samples sharing the same host plant."

(line 581-584 in the main text)

"Third, a supplementary calculation of $\Delta topography_{i,h}$ and $\Delta evenness_{i,h}$ scores was performed based on an alternative randomization-block setting (shuffling among samples with both the same host plant identity and spatial sampling block; Supplementary Figs. S42–S43)."

-- Reviewer 2---

This is a very well written manuscript that describes a novel approach to identify keystone taxa using an approach inspired by statistical physics. The approach is tested on real sequencing data from microbial communities associated with ectomycorrhizal and dual-mycorihizal plants. Importantly, capabilities and potential ecological roles of identified keystone taxa are discussed in detail, revealing that the their inferred status as keystone taxa is biologically meaningful, thereby validating the approach. The study provides new insights and is highly relevant to the understanding of characteristics and identification of keystone taxa. The manuscript comprises careful and detailed analyses, and all conclusions are clearly derived from the presented data. However, I think that the structure and the clariyt of the manuscript can be improved (see comments below). I struggle with understanding the theoretical framework. This is not necessarily due to a weakness of the paper, but may rather reflect that statistical physics is not within my area of expertise.

[RESPONSE] Thank you very much for your encouraging comments and constructive suggestions on the manuscript. As suggested in the following comments, we carefully revised the manuscript structure and clarified the presentation of the concepts and methods introduced.

General comments:
i) The authors make use of the term 'assembly rules' numerous places in the text (including the title). But it is not clearly explained what an assembly rule is or can be. And what is the relationship between assembly rules and assembly landscapes? Is the stability of a community only dependent on the assembly landscape or a combination of the assembly landscape and assembly rules?

[RESPONSE] We thank the reviewer for pointing out the need to clarify the meaning of the term "assembly rules" and its relationship to assembly landscapes. In community ecology, "assembly rules" generally refer to the ecological processes that constrain which combinations of species can coexist in a community, including environmental filtering, dispersal limitation, and species interactions. From the conceptual framework of stability landscapes, such assembly rules are reflected in the number and configuration of alternative stable states.

In this study, the assembly landscape is used as a statistical representation of the observed distribution of community states that emerges from these underlying assembly rules. Specifically, the inferred landscape describes the probability structure of community compositions, where basin bottoms correspond to frequently observed or stable community states and basin boundaries represent compositional transitions. From this perspective, assembly rules are not directly observed but are reflected in the structure of the assembly landscape.

Accordingly, changes in the number or configurations of basins within assembly landscapes can be interpreted as changes in the ecological constraints governing community assembly. We clarified this relationship in the Introduction as follows.

(lines 52-63 in the main text)

> "The bottoms of these valleys are interpreted as "alternative stable states" toward which community structures tend to converge from nearby configurations[18,19]. Large shifts in community states are often interpreted as stochastic transitions between neighboring basins[17,19], whereas major shifts may also occur when the landscape topography itself is reorganized[18,19]. Factors altering the number or positions of basins are therefore central to understanding community-scale dynamics. Importantly, such factors are not necessarily abiotic. Because species interactions contribute to the constraints governing community assembly (assembly rules), changes in the abundance of particular taxa (or species) that strongly influence other community members can reshape stability landscape topography. Therefore, quantifying such reorganizations of landscape architecture, reflected in changes in the number and configuration of alternative stable states, provides a statistical basis for evaluating the extent to which a taxon reorganizes the rules underlying community assembly."

Regarding the reviewer's question about stability, the stability of a community is not determined solely by the landscape structure itself but by how community dynamics interact with the inferred landscape topography. In previous work, indices derived from energy landscape analysis, such as energy gaps and stable-state entropy, have been used to quantify community-scale stability (Fujita et al. 2023; https://doi.org/10.1186/s40168-023-01474-5). However, because the focus of the present study is on identifying taxa that reorganize assembly landscapes, we did not further analyze these stability indices here.

ii) I think the 'flow' of the paper can be improved by being more clear about the main motivation behind the paper. Is it to better understand the role of various groups of fungi and bacteria in plant root systems or is it to develop a method for the identification of keystone taxa? In the first case it would seem natural to start the introduction with fungi and bacteria (which is done), but also start the

results section with the sequencing data and then move on to identifying keystone taxa. In the second case it would seem natural that the first sentence in the intro is about the problem of defining and identifying keystone taxa (using fungi and bacteria in root systems more as an example/illustration of the problem and a good system for method validation). Then, in the result section, describe method development and test the method with real sequencing data for validation (which is also a matter for the discussion).

[RESPONSE] Our primary goal is to understand the ecological roles of fungi and prokaryotes in forest root-associated microbiomes in light of community assembly rules. To address this ecological question, we developed a statistical framework for identifying candidate keystone taxa from observational microbiome data. In other words, the methodological development serves as a tool to investigate the ecological importance of microbial taxa rather than being an end in itself.

To clarify this motivation and improve the overall flow of the manuscript, we revised the first paragraph of the Introduction as follows:

(lines 37-48 in the main text)

> "Fungi and bacteria associated with plant roots support host survival, nutrient acquisition, and ecosystem functioning in terrestrial environments[1–4]. Numerous studies have suggested that such plant microbiomes may harbor “keystone” taxa exerting disproportionate influences on community structure and ecosystem processes[5–10]. However, comprehensive experimental validation of candidate keystone microbes remains constrained by practical limitations. Many plant-associated microbes depend strongly on host-derived carbon and are therefore difficult to examine using culture-based approaches[11]. Even when culturing is feasible, demonstrating ecosystem-scale impacts would generally require large spatial-scale manipulations conducted over long temporal scales. Conducting such experiments for thousands of microbial taxa present in natural communities is therefore generally impractical. Consequently, data-driven analytical frameworks capable of evaluating the potential roles of diverse microbes directly from observational data are essential for advancing our understanding of below-ground ecosystem processesc."

In addition, we restructured the Results section to clarify this flow. The Results now first present the sequencing dataset and the overall patterns of fungal and prokaryotic community composition, followed by the identification of candidate keystone taxa using the proposed analytical framework (lines 93–144 in the main text).

iii) In my view the results include sections that seem to fit better in the introduction: See for example L97-104 which describe a motivation for doing the study, but not results. L105-113 is also more about motivation and the authors continue to refer to Fig1 (explained in the Intro). L114 and onwards sounds more like results, but it is a bit confusing that the authors refer to Fig1a-right, which seems to be an illustration not actual results. From L116 the authors explain what an anergy landscape analysis is and how it has been used previously, which I also think would fit better in the introduction. L119-136 could also fit better in intro and methods, I think. L137-138 describes a result. But L138-147 describes "strategy" and asks the reader to "imagine" things, which are not results. Here are some suggestion: Keep Fig1 out of the intro. Rephrase a subtitle in the results (L95) into something like 'developing a method for identification of assembly landscapes and detection of possible keystone taxa'. Under this section focus on objectively explaining how the method works (Fig 1) (not describing results of applying the method), then (as is done now) show the results of applying the method on your amplicon datasets.

[RESPONSE] We thank the reviewer for the helpful suggestions regarding the structure of this section. Following these comments, we revised this part of the manuscript to more clearly separate conceptual motivation, methodological explanation, and empirical results.

First, sentences describing the motivation for the study (previously lines 97–104 and 105–113) were summarized and moved to the Introduction so that the Results section focuses on methodological explanation and empirical findings (lines 49–77 in the main text).

Second, the description of the analytical framework (previously lines 116–136) was entirely reorganized to improve the flow of the Result section (lines 110–138 in the main text). Detailed descriptions of the energy landscape analysis and the keystone-exploration framework were moved to the Method section (lines 463–514 and 516–585 in the main text).

We also reorganized the references to Figure 1. Specifically, Figure 1a, which illustrates the conceptual relationship between stability landscapes and inferred assembly landscapes, is now presented only in the Introduction. In contrast, Figure 1b–g are referred to in the Results section, in which they are used to explain the analytical framework and to facilitate presentation of the empirical results.

To further clarify the purpose of this subsection, we also rephrased the subtitle of this section as suggested by the reviewer (line 109 in the main text).

iv) It would be nice if the authors could add more detailed information about the workflow in the README file in the github repository (https://github.com/ngchngch/Assembly_landscape_of_the_forest_root_microbiome) to make the

method and code more accessible.

[RESPONSE] As suggested, we added detailed explanations about our workflow and examples of R scripts for performing analyses used in this study to the the github repository (https://github.com/ngchngch/Assembly_landscape_of_the_forest_root_microbiome).

Minor comments:

L66-70: "Although directly reconstructing the architecture of stability landscapes (Fig. 1a-left) from empirical data". But it is Fig1a-right that shows the empirical assembly landscape - Should it be "Fig. 1a-right [...] Fig. 1a-left"?

[RESPONSE] We compared conceptual stability landscape (Fig. 1a-left) and statistically inferred "assembly landscape" (Fig. 1a-right) in this sentence. To clarify the relationship between the concept and empirical reconstruction, we revised the sentence as follows.

(lines 65-68 in the main text)

> "Although directly reconstructing stability landscapes from empirical data remains challenging[20,21], statistical-physics-inspired approaches enable reconstruction of landscapes describing community-state probability distributions[14,15,22,23]. We refer to these empirically derived representations as "assembly landscapes" (Fig. 1a-right)."

L245-247: should it be 'were inferred to exert'?

[RESPONSE] The sentence was reorganized as follows:

(lines 204-206 in the main text)

> "Among the examined microbes, several genera of root endophytic fungi were significantly associated with both the overall architecture and higher-order properties of fungal assembly landscapes (Fig. 4b)."

L474: What defines the presence or absence of taxon i? From Fig 1f absence does not seem to a absence of reads, but rather based on a threshold (presumably based on relative abundance and not absolute number of reads).

[RESPONSE] To clarify how presence and absence were defined in this study, we revised the description of the data preprocessing procedure in both the main text and the Supplementary Information. In our analysis, taxon presence was determined based on a relative-abundance threshold applied to the sequencing data rather than the complete absence of reads (Supplementary Fig. S9a; lines 73–94 in Supplementary Information). Taxa whose relative abundance exceeded the predefined threshold were treated as present, whereas those below the threshold were treated as absent for the purpose of binarizing the community data.

The revised sentence in the main text reads as follows:

(lines 474-476 in the main text)

> "Each element ${\sigma_i}^{(k)}$ is a binary variable indicating the presence (1) or absence (0) of taxon *i*, defined using optimized thresholds applied to relative-abundance data (Supplementary Methods; Supplementary Fig. S9a)."

Is CLR center log ratio? I don't think CLR is defined in the text.

[RESPONSE] Yes, we used the term CLR for representing centered log-ratio. We added the definition of this abbreviation throughout the manuscript.

It would be instructing to see an illustration of the difference between a keystone-taxon and a non-keystone-taxon among the main figures - either as an extension of Fig1 or as a separate figure. At least indicate in the legend of Fig1 how Fig1d-g can be used to infer that the target taxon is a keystone taxon.

[RESPONSE] We thank the reviewer for this helpful suggestion. We agree that readers may benefit from a clearer explanation of how the proposed framework distinguishes taxa with strong landscape-reorganizing effects from those with weaker effects.

In our framework, the influence of taxa on community assembly is treated as a continuous property rather than a binary classification. Therefore, instead of defining keystone taxa using a strict threshold, we evaluate the magnitude and statistical significance of two indices (*Δtopography* and *Δevenness*) and highlight taxa with large and significant values as representative candidate keystone taxa.

To clarify how the inference is made, we revised the legend of Figure 1 to explicitly explain how the panels d–g illustrate the detection of candidate keystone taxa. In particular, the legend now describes how changes in landscape architecture between low- and high-abundance conditions of a focal taxon

lead to large values of *Δtopography* and/or *Δevenness*, which indicate strong effects on the assembly landscape.

(lines 971–975 in the main text)

> “Based on comparison with randomized null model simulations, these two indices were standardized, and their statistical significance was evaluated. Taxa exhibiting large and significant *Δtopography* and/or *Δevenness* values are highlighted as candidates of "keystone taxa", reflecting their strong influence on the topography of the inferred assembly landscape.”

The repository contains a README file, but details on how to use the code (e.g. example workflow) is lacking.

[RESPONSE] As suggested, we added example workflow for performing analyses used in this study to the GitHub repository (https://github.com/ngchngch/Assembly_landscape_of_the_forest_root_microbiome).

-- Reviewer 3---

The authors proposed an computational/statistical analysis method based on the so-called energy landscape analysis based on the inverse Ising model. This method has been studied in particular for functional magnetic resonance imaging (fMRI) data from the brain and more recently in various ecological/microbiological data such as the present one. The present authors introduced two metrics to the Ising-based energy landscape analysis, "¥Delta topography" and "¥Delta evenness", to measure the impact of the change in the abundance of a species on the shape of the energy landscape inferred from data. In this way, they proposed to quantify the "keystone-ness" of individual species as the size of "¥Delta topography" and "¥Delta evenness". They applied the methods to microbial abundance data they collected from a Japanese forest.
The paper is vaguely written in various methodological aspects, which confused me a lot (see below). Putting this issue aside, my main criticism is that there is no biological ground truth data analyzed to validate outcomes of the energy landscape analysis (called assembly landscapes in the manuscripts). In other words, they suggested some keystone species (and ran other analyses) based on their method, but there is no way to tell whether the inferred keystone species are really keystone species in some biological or ecological sense. Therefore, one cannot tell whether the results are practically useful. On this ground, I do not recommend the publication of this article in Nature Communications. See below for specific feedback.

[RESPONSE] We appreciate the reviewer's careful evaluation of our manuscript and the detailed comments regarding both methodological clarity and biological validation. The reviewer raises two important concerns: (i) that several methodological aspects of the framework were not sufficiently explained, and (ii) that the ecological relevance of the inferred keystone taxa requires stronger validation.

In response, we substantially revised the manuscript in two major ways. First, we expanded the methodological descriptions of the energy landscape framework, including the procedures for data binarization, basin detection, and keystone-sensitivity analysis, to improve transparency and reproducibility. Second, we added multiple validation analyses to evaluate the biological and ecological relevance of the proposed framework. These include in silico benchmark tests using generalized Lotka–Volterra simulations, robustness analyses using cross-validation and bootstrapping, and additional empirical analyses examining associations between candidate keystone taxa and independent ecological indicators.

Together, these additions provide both mechanistic benchmarking and empirical support for the practical interpretability of the inferred assembly landscapes and candidate keystone taxa. Detailed responses to the reviewer's specific comments are provided below.

(1) Taking neuroscience as example (because this sort of analysis has been used a lot there), once one infers energy landscapes from fMRI data, the results are probably publishable but the impact of the work is weak. The reason is that we cannot then tell whether products of the energy landscape analysis are useful. This is why almost all papers using this analysis method (or other relatively similar methods) published in high tier journals (even journals that neuroscientists (including computational neuroscientists) consider substantially below Nature Communications) do some "ground truth analysis", which for example means that some quantity obtained from energy landscape analysis is correlated with some non-fMRI quantity measured from the participants in the experiment (e.g., severity of the disease, score of the cognitive task). I do not work on microbial biology, so I cannot tell the difference between the two fields; but I apply the same criterion here. What are *biological/ecological* knowledge obtained by this work? How is the present method and obtained/proposed biological/ecological knowledge validated? These are entirely lacking in the present manuscript. This is related to the fact that this manuscript is full of "be inferred", "be estimated", "could govern", etc., and says few things decisively.

[RESPONSE] The reviewer raises an important point regarding the need for biological validation of the inferred keystone taxa and the ecological interpretation of the assembly landscape analysis. In response, we added two complementary lines of validation to the revised manuscript: (i) cross-

system empirical validation using an independent microbiome dataset and (ii) in silico benchmarking based on mechanistic ecological models.

First, we examined whether the taxa highlighted by the keystone exploration analysis in a cool-temperate forest in Japan were also associated with large-scale patterns in root-associated microbiomes across Europe. Using a recently published dataset of woody-plant root microbiomes spanning a broad geographic range across European forests (Netherway et al., 2024; https://doi.org/10.1038/s41467-023-44172-4), which includes both amplicon sequencing data and microscopic observations of mycorrhizal colonization, we evaluated the ecological impacts of candidate keystone taxa using generalized linear mixed models (GLMMs). These analyses showed that several fungal and bacterial genera identified by the assembly landscape analysis in Japan were significantly associated with variation in the prevalence of arbuscular mycorrhizal and ectomycorrhizal symbiosis across European forest ecosystems (Fig. 7). These cross-system patterns provide independent ecological support for the biological relevance of the candidate keystone taxa highlighted by our framework.

Second, we conducted *in silico* benchmark analyses to evaluate the methodological performance of the proposed framework. Using generalized Lotka–Volterra (gLV) models incorporating multiple types of ecological interactions (e.g., competition, parasitism, commensalism, and mutualism), we examined the robustness of the energy landscape analysis under different data properties, including community complexity (species richness) and sample size (number of replicate communities) (Supplementary Fig. S48). In addition, simulations of community dynamics were used to test whether the proposed sensitivity analysis can identify species exerting disproportionate influence on community assembly. These analyses demonstrated that taxa with large *Δtopography* values correspond to species with stronger impacts on simulated community dynamics (Supplementary Figs. S7–S8).

Together, these empirical and simulation-based validations provide complementary evidence supporting both the ecological interpretability and the methodological robustness of the proposed assembly landscape framework. Although these analyses cannot definitively prove keystone effects in natural ecosystems, they demonstrate that the taxa highlighted by the framework correspond to ecologically meaningful patterns and generate testable hypotheses for future experimental validation.

(2) The presentation of the methods is poor, confusing readers (at least me). I understand that details of the methods need to sit in the Methods section that appears after the Discussion/Conclusion section. However, the Results section should read smoothly, even without precise definitions of the quantities used. Furthermore, at least in the Results section, several terms are undefined/unexplained, which practically confused me. Introduction materials are explained in the beginning of the Results section. All these make reading and understanding of the results harder. Specifically,

(2-1) lines 97-113: These two paragraphs are Intro (i.e., background) materials. I also note that if we move these two paragraphs to the Introduction section, the Introduction section is a bit long. In a related vein, in lines ~65-80, What is the key question in this paper? The new methods are developed because the authors want to find something biological. I suggest reformulating the Introduction section completely to deliver the key questions and knowledge gaps more clearly, no matter the journal.

[RESPONSE] The reviewer raises an important point regarding the clarity of the manuscript structure and the presentation of the key research questions. In response, we revised both the Introduction and the Results sections to improve the logical flow and ensure that background material and methodological explanations are presented in appropriate sections.

First, the opening part of the Introduction was reorganized to more clearly articulate the key question and knowledge gap addressed in this study. In particular, we now emphasize that the central challenge in microbiome ecology is identifying candidate keystone taxa among the thousands of microbial species present in natural communities, especially in systems where large-scale manipulative experiments are impractical. This perspective motivates the development of data-driven statistical frameworks that can prioritize taxa for subsequent experimental validation.

To reflect this change, we revised the Introduction as follows:

(lines 37–48 in the main text)

> "Fungi and bacteria associated with plant roots support host survival, nutrient acquisition, and ecosystem functioning in terrestrial environments[1–4]. Numerous studies have suggested that such plant microbiomes may harbor “keystone” taxa exerting disproportionate influences on community structure and ecosystem processes[5–10]. However, comprehensive experimental validation of candidate keystone microbes remains constrained by practical limitations. Many plant-associated microbes depend strongly on host-derived carbon and are therefore difficult to examine using culture-based approaches[11]. Even when culturing is feasible, demonstrating ecosystem-scale impacts would generally require large spatial-scale manipulations conducted over long temporal scales. Conducting such experiments for thousands of microbial taxa present in natural communities is therefore generally impractical. Consequently, data-driven analytical frameworks capable of evaluating the potential roles of diverse microbes directly from observational data are essential for advancing our understanding of below-ground ecosystem processes."

Second, introductory background material previously included in the Results section (lines 97–113 in the original manuscript) has been summarized and moved to the Introduction. The Results section was then reorganized so that it focuses on presenting the analytical framework and the empirical findings, whereas key concepts and terms are introduced at appropriate points to improve readability.

These revisions were made to ensure that the Results section can be read more smoothly without requiring detailed methodological definitions at the outset, while maintaining clear explanations of the analytical framework (lines 110–144 in the main text).

(2-2) line 123: "each step". "step" means what?

[RESPONSE] To avoid potential ambiguity associated with the term “step”, we revised the text to describe the structure of the binarized state space more explicitly without using this term. The revised sentence clarifies that community states are represented as binary presence–absence configurations and that the corresponding state space consists of possible combinations of taxa.

(lines 111–116 in the main text)

> "This approach reconstructs a probabilistic representation of community states, hereafter referred to as an "assembly landscape", based on model-implied probabilities derived from an Ising-model framework in statistical physics[14,27,28]. In this context, community states are represented in binary format (presence or absence of taxa), following the assembly-graph concept in community ecology[29–31]. For a system containing S taxa, the corresponding state space consists of 2^S possible states."

(2-3) line ~123: I do not even understand what the node is by reading the text around here. It is also crucial to mention that each species abundance (?) is binarized for this analysis. The authors should not have too much methodological details in the Results section of this type of journal, indeed, but a minimum is necessary for readers to be able to read through.

[RESPONSE] We appreciate the reviewer’s comment regarding the clarity of the terminology used in this section. In the revised manuscript, we simplified the description of the assembly landscape framework to improve readability and avoid potentially confusing technical terms such as “node” in the Results section. Instead, we describe community states more directly as presence–absence configurations of taxa (lines 111–119 in the main text).

We also clarified that species abundances were binarized for the energy landscape analysis, such that each taxon is represented as either present or absent in a given sample. Specific descriptions of the binarization procedure have been moved to the Methods section (lines 464–477 in the main text) and Supplementary Methods (lines 73–94 in Supplementary Information) referred to in the Methods section.

These revisions were made to ensure that the Results section can be read more smoothly while still providing the minimum conceptual explanation necessary to understand the analytical framework.

(2-4) lines 143-145: I do not understand the idea of external variables (including later in the Results section), despite that changing the external variable and running the same analysis to get different results seems to be a key development later in this Results section.

[RESPONSE] The role of the external variable was clarified in the revised manuscript. In our framework, the relative abundance of a focal taxon is treated as an external variable that conditions the probabilistic structure of community states in the energy landscape model (i.e., ε_j in eq. 3 in Methods). By varying the abundance level of the focal taxon and recalculating the corresponding probability distributions of community states for the remaining taxa, we evaluate how the inferred landscape architecture changes along the abundance gradient of that taxon.

To make this idea clearer without introducing excessive mathematical detail in the Results section, we revised the text as follows:

(lines 120–127 in the main text)

> "Based on this framework, we developed a sensitivity-analysis approach to identify candidate keystone taxa associated with assembly landscape reorganization (Fig. 1d–g; see Methods for details). The central idea is to quantify how landscape architecture changes along the abundance gradient of each focal species or taxon. If the number or positions of basins vary markedly across abundance levels, the focal taxon is considered strongly associated with shifts in assembly processes (Fig. 1d–e). In this analysis, the relative abundance of the focal taxon is treated as an external variable influencing the modeled probabilities of community states (i.e., assembly landscape architecture) for the remaining community members (Fig. 1f–g)."

(2-5) lines 152-153: What are "community-state probabilities"? In fact, the authors might better not to explain all terms in the Results section. Anyways, the methodological explanation in the Results section should be concise enough and also good enough so that hasty readers who would skip

reading the Methods section can understand the results grossly. I think rework is needed to reach that standard.

[RESPONSE] The expression "community-state probabilities" in the previous manuscript referred to the probabilities assigned by the fitted statistical model to each possible community state (i.e., each presence–absence configuration of taxa). To avoid ambiguity, we revised the description in the Results section to refer explicitly to the probability distribution of community states.

In addition, following the reviewer's suggestion, we revised the Results section to reduce the use of technical terminology and improve readability for readers who may not immediately refer to the Methods section. The explanation of the assembly landscape framework was simplified to focus on the conceptual interpretation of the analysis, while detailed methodological definitions were reorganized in the Methods section.

The revised sentence reads as follows:

(lines 131–132 in the main text)

"The second index, *Δevenness*, captures changes in the evenness of the probability distribution of community states along the abundance gradient of the focal taxon (Fig. 1e)."

(2-6) lines 220-236: No need to repeat the methods explained above.

[RESPONSE] We removed the repetitive descriptions of the methods and reorganized the paragraph (lines 191-193 in the main text).

(2-7) lines 257, 259: "organizer" means what?

[RESPONSE] The term "organizer" used in the previous manuscript could be ambiguous in this context. To avoid potential confusion, we removed this expression and replaced it with more explicit descriptions of taxa influencing assembly landscape structure in the revised manuscript.

[other minor]

(3) line 118: "nerval ignition patterns" --> "brain activity patterns", "neuronal activity patterns",

something like that? fMRI does not measure spiking/firing of neurons (which the authors meant by ignition?) directly.

[RESPONSE] The term "neural ignition patterns" in the previous manuscript was inappropriate in this context. Following the reviewer's suggestion, we replaced it with "brain activity patterns" in the revised manuscript. In addition, the sentence was moved from the Results section to the Methods section during the reorganization of the manuscript (lines 465–468 in the main text).

(4) line 119: "When applied to community ecology". This sentence should be reformulated because "the framework employs ..." is also true when applied to any other areas.

[RESPONSE] This sentence was removed during the reorganization of the Result and Method sections.

(5) lines 155-157: This stabilizing effect holds true only for negative "¥Delta evenness" values, not for low but positive values.

[RESPONSE] Yes. To clarify the suggested point, the sentence was revised as follows (lines 132-136 in the main text):

> "A positive *Δevenness* value is associated with redistribution toward many narrower basins, implying the focal taxon's destabilizing effects on community assembly. In contrast, a negative *Δevenness* value indicates assembly landscapes with fewer but broader basins, implying increased dominance of major community states."

(6) lines 196-203: The results depend on the host-plant background. What do we learn from this result?

[RESPONSE] This result indicates that the structure of assembly landscapes can depend strongly on host-plant background. In particular, the number and composition of alternative stable states in fungal communities varied among host plants, suggesting that host identity can modify the ecological constraints shaping microbiome assembly. These findings suggest that candidate keystone taxa and their impacts on community organization may also be context dependent across host plants.

To clarify this ecological interpretation, we reorganized the sentence as follows:

(lines 166–167 in the main text)

"The topography of the fungal assembly landscape varied among host plants, suggesting that assembly rules differ depending on host ecological and physiological traits."

--- Reviewer 4--

In this manuscript, the authors conduct a comprehensive analysis of the assembly landscape of forest root microbiomes, examining both fungal and prokaryotic communities. By treating the relative abundance of a focal taxon as an environmental variable, they define its "keystoneness" based on the extent to which changes in that taxon's abundance alter the inferred assembly landscape. The authors apply this framework to a well-collected forest root microbiome dataset, and the results and figures are clearly presented. However, the method lacks validation using in silico simulations (e.g., generalized Lotak-Volterra model or others), and several methodological aspects remain unclear. Therefore, I have several comments and concerns as listed below.

[RESPONSE] We thank the reviewer for the careful evaluation of our manuscript and for the constructive suggestions. As suggested, we conducted additional in silico simulations based on generalized Lotka–Volterra (gLV) models to evaluate the performance of the proposed framework under controlled ecological dynamics. The results of these simulation analyses are now presented in the revised manuscript (Supplementary Figs. S6–S8).

In addition, we revised and clarified several methodological descriptions and conceptual explanations in both the main text and the Supplementary Information. In particular, we expanded the explanations of the energy landscape inference procedures and the keystone-exploration framework to improve clarity and reproducibility.

Detailed responses to the reviewer's specific comments are provided below.

Major comments

1. The effectiveness and ecological relevance of the statistical energy-landscape framework are not sufficiently demonstrated. The method needs to be validated using in silico simulations based on mechanistic models, such as gLV or consumer-resource models. It remains unclear how accurately the approach can identify basins of attraction, the extent to which its outputs correspond to underlying community stability properties, and how sensitive the results are to data sparsity (i.e. sample size vs. ecosystem complexity).

[RESPONSE] To evaluate the effectiveness and ecological relevance of the statistical energy-landscape framework, we conducted validation analyses using *in silico* simulations based on generalized Lotka–Volterra (gLV) models.

First, we examined the robustness of basin detection under different combinations of sample size and community complexity (species richness). gLV simulations were performed across a range of species numbers, and assembly landscapes were reconstructed from simulated community states. The reproducibility of the inferred basin structures was quantified using Jensen–Shannon divergence among simulation replicates. The results showed consistently low divergence values, indicating highly reproducible basin structures (Supplementary Fig. S48b). In particular, when assuming a sample size comparable to that of our empirical dataset (approximately 1,200 samples), the Jensen–Shannon divergence fell below 0.05 for most combinations of species richness and parameter settings (Supplementary Fig. S48b; lines 334–350 in Supplementary information).

Second, we evaluated whether the proposed keystone-exploration framework captures the ecological influence of species on community dynamics (Supplementary Fig. S6). For each species in the gLV simulations, we ran simulations with and without the focal species and quantified the resulting changes in community composition (excluding the focal species) as a measure of its ecological impact. In parallel, we calculated the *Δtopography* index for each species based on the sensitivity analysis of the inferred assembly landscapes. Species with higher *Δtopography* values produced larger changes in community composition, indicating that the proposed index captures taxa with stronger influences on community-scale dynamics (Supplementary Figs. S6–S8).

Descriptions of these benchmark analyses have been added to the Results (lines 139–144 in the main text) and Methods (lines 606–713 in the main text) sections of the revised main text and to the Supplementary Result section (lines 334–350 in Supplementary Information).

2. The authors present the taxonomic compositions of fungal and prokaryotic communities across all root-tip samples; however, the manuscript lacks PCoA analysis that compares these compositional patterns with the basins of attraction inferred from the statistical energy-landscape model. As the statistical model relies on presence/absence data, it is unclear whether samples assigned to the same basin truly share similar community composition profiles. For example, do samples from different plants that fall into the same basin exhibit similar taxonomic profiles?

[RESPONSE] As suggested, we performed principal coordinate analysis (PCoA) of the fungal and prokaryotic community datasets and added the results to the revised manuscript (Supplementary Fig. S12). The ordinations were constructed based on family-level community compositions using Bray–Curtis dissimilarity (abundance-based) and Jaccard dissimilarity (presence–absence-based).

These analyses showed that samples assigned to the same basin in the energy-landscape framework tended to occupy similar regions in the ordination space, even when they originated from different host plants. This result indicates that samples grouped into the same basin generally share similar community composition profiles.

At the same time, the correspondence between abundance-based community composition and basin assignment is not necessarily perfect. Because the energy landscape analysis relies on binarized community matrices after taxon-selection steps, some information contained in abundance data may be partially lost. We therefore clarified this point in the Discussion section.

(lines 428-433 in the main text)

> "Third, due to its exhaustive computational approach, our current analysis targeted assembly landscapes defined with the binary-format (presence/absence) data of the family-level community structure. More detailed simulations of community dynamics will be achieved by extending computational capacities and applying emerging algorithms for summarizing high-dimensional microbiome data (e.g., natural language processing of the latent Dirichlet allocation model for quantitative microbiome data[22])."

3. The ecological interpretation of the "keystone taxa" identified by this statistical framework is not well articulated. Several key studies that define and characterize keystone species or keystone taxa in microbial communities (e.g., Amit & Bashan 2023; Wang et al. 2024) are not cited or discussed, leaving the reader without a clear comparison between this framework and existing ecological definitions or methodological approaches. Integrating these perspectives is important to clarify whether the taxa identified by the energy-landscape method align with established ecological concepts of keystone effects. The author should benchmark the method against simulated community dynamics or providing empirical evidence that the identified taxa exert biologically meaningful influence within root microbiomes. Thus, both conceptual grounding and validation are needed to strengthen the interpretation and practical utility of the identified keystone taxa.

[RESPONSE] As suggested, we expanded the Discussion to better position the proposed framework within the broader literature on keystone species and keystone taxa in microbial communities. In particular, we incorporated recent studies that propose alternative approaches for identifying microbial keystone taxa (Amit & Bashan 2023; Wang et al. 2024) and clarified how the present framework relates to these existing concepts and methods.

Specifically, the revised manuscript now discusses how previous studies have identified candidate keystone taxa using approaches such as co-occurrence network topology or *in silico* perturbation analyses. In contrast, the framework proposed here evaluates how changes in the abundance of a

focal taxon reshape the topography of the inferred assembly landscape. This perspective connects the identification of keystone taxa with theoretical concepts from stability landscape analysis and community assembly dynamics.

(lines 323-335 in the main text)

“A keystone species or taxon was originally defined as "one whose impact on its community or ecosystem is large, and disproportionately large relative to its abundance[9]”. Whereas this classic definition has primarily been applied in studies of macro-organisms (e.g., plants and animals), microbiome research has continued to propose modified operational definitions to address the complex and dynamic nature of microbial interactions[5,37–39]. Specifically, network hubs identified from microbial co-occurrence patterns are often regarded as candidate keystone taxa[5,40–42]. More recently, several *in silico* approaches have been proposed to evaluate how the presence or removal of particular taxa may affect overall community structure[37,38]. Building upon these advances, our approach extends such pattern-oriented frameworks by integrating theoretical perspectives on system-scale principles underlying community dynamics. Based on the stability landscape concept, a keystone species (or taxon) can therefore be more explicitly conceptualized as one whose increase or decrease in abundance reshapes the rules governing community assembly.”

References added:

Amit, G. & Bashan, A. Top-down identification of keystone taxa in the microbiome. *Nat. Commun.* **14**, 3951 (2023).

Wang, X.-W. *et al.* Identifying keystone species in microbial communities using deep learning. *Nat. Ecol. Evol.* **8**, 22–31 (2024).

Garza, D. R., Gonze, D. & Faust, K. Keystone concept revisited: insights into microbial community dynamics and control. *Nat. Rev. Microbiol.* https://doi.org/10.1038/s41579-025-01266-8 (2026)

We also added validation analyses to evaluate whether the taxa highlighted by the framework correspond to biologically meaningful influences on community dynamics. First, in silico benchmark tests based on generalized Lotka–Volterra (gLV) models were conducted to assess the ability of the method to detect taxa exerting strong influences on community dynamics (Supplementary Figs. S6–S8 and Methods). Second, we examined an independent empirical dataset of forest microbiomes across Europe (Netherway et al., 2024; https://doi.org/10.1038/s41467-023-44172-4) and found that several candidate keystone taxa identified in our analysis showed significant associations with mycorrhizal symbiosis across a broad geographic range (Fig. 7; lines 280–316 in the main text).

Other comments:

1. The authors use the term “50% abundance-quantile” in multiple places in the manuscript. This terminology may confuse readers without a statistical background, as it could be misinterpreted as “50% relative abundance” rather than the median relative abundance across samples. Consider replacing “50% abundance-quantile” with “median abundance” throughout the manuscript for clarity.

[RESPONSE] We clarified in the revised manuscript that the term “50% abundance quantile” refers to the median relative abundance across samples. To improve clarity for readers without a statistical background, we now explicitly indicate this as “median abundance (50% quantile)” where appropriate in the text.

2. The authors identify keystone taxa by removing the corresponding genus from the dataset. It remains unclear whether removing a genus that is a major contributor to its family could introduce bias into the analysis.

[RESPONSE] In our framework, the effect of each genus on the assembly landscape is evaluated using standardized keystoneness indices derived from comparisons with randomized null models. Because these indices are calculated as deviations from null expectations generated under the same analytical procedure, the removal of a focal genus is unlikely to introduce systematic bias into the analysis.

To clarify how genus removal is handled, we revised the Methods section to explicitly describe the procedure used in the sensitivity analysis. In brief, OTUs annotated to the focal genus were removed, after which the remaining OTUs were aggregated at the genus or family level before reconstructing the community matrix. The subsequent binarization and taxon-selection procedures were then applied using the same criteria as in the original analysis. This ensures that the influence of genus removal is evaluated consistently relative to comparable null-model expectations.

(lines 523-528 in the main text)

> “Within the sensitivity analysis framework, we sequentially re-defined the community data matrix by excluding each focal genus. Specifically, OTUs annotated to the focal genus were removed, and the remaining OTUs were aggregated at the family-level community compositions (Fig. 1f and Supplementary Fig. S9b). We then binarized the abundance of the taxa targeted in

the above energy landscape analysis using the same criteria. During this procedure, taxa occurring in 30 or fewer samples were excluded."

3. The authors analyze keystoneness only for genera detected in more than 100 root-tip samples (Line 499). It is unclear whether the energy landscape analysis includes samples where the target genus is absent (0 abundance) or only those where it is present. Extending the inferred keystoneness to 0 abundance may introduce bias, particularly if absent samples were not included in the analysis. Moreover, the prevalence and overall abundance of a genus could influence its keystoneness. The authors should evaluate whether taxa are identified as keystone simply because they are highly prevalent or abundant, rather than due to genuine ecological influence.

[RESPONSE] The energy landscape analysis explicitly includes both presence and absence states of the focal genera. In the revised manuscript, we examined the number of samples classified as absent for each focal fungal and prokaryotic genus based on the predefined detection threshold. This analysis confirmed that even for the genus with the fewest absence records, at least 40 samples were classified as absent. Therefore, absence conditions were sufficiently represented in the dataset used for landscape inference, making substantial bias from extrapolating to zero abundance unlikely.

To clarify this point, we added the following sentence to the main text.

(lines 522-523 in the main text)

"Prior to this analysis, we confirmed that each focal genus was absent in at least 40 samples."

In addition, we evaluated whether taxa with high keystoneness values were simply those with high prevalence or abundance. We confirmed that genera showing relatively strong *Δtopography* or *Δevenness* were not necessarily those that were highly prevalent or abundant in the dataset. This point is now clarified in the Results section (line 201-203 in the main text).

"Notably, genera showing relatively strong effects on these indices were not necessarily those that were highly prevalent or abundant in the root microbiome dataset (Supplementary Figs. S15–S18)."

4. The authors do not sufficiently explore or hypothesize about the mechanistic biological drivers that could underlie the inferred basins and keystone taxa. Linking the statistical keystones to ecological functon(e.g., nutrient cycling, plant fitness, stability) would make the findings more

biologically meaningful; some explicit hypotheses or suggestions for experimental validation would strengthen the ecological relevance of the study.

[RESPONSE] As suggested, we expanded the Discussion to better connect the statistically identified candidate keystone taxa with potential ecological mechanisms and functional roles in root-associated microbiomes. In particular, we added examples of biological traits that could explain the strong influence of certain taxa on community assembly dynamics. For instance, we discuss how melanin production in dark septate endophytes may contribute to competitive superiority during early root colonization through resistance to chemical and physical stressors.

(lines 348–350 in the main text)

> "For example, melanin, which characterizes the appearance of dark septate endophytes, likely contributes to competitive superiority in chemical and physical interactions with other early-colonizing fungi in plant root systems[48,49]."

In addition, we reorganized the paragraphs discussing the potential ecological functions of candidate keystone taxa to more clearly relate the statistical results to known microbial traits and ecological processes (lines 351–369; 388–402 in the main text). We also added brief discussion of how the proposed framework could guide future experimental validation, for example by testing whether the presence or removal of candidate keystone taxa alters microbiome assembly or plant–microbe interactions (lines 415–422 in the main text).

5. The authors should expand the analysis and discussion on cross-kingdom interactions (i.e. fungi-bacteria interactions).

[RESPONSE] As suggested, we expanded both the analyses and the discussion regarding potential cross-kingdom interactions between fungi and prokaryotes. In the revised manuscript, we more explicitly examined cases in which taxa from one kingdom were identified as candidate keystone taxa influencing the assembly landscapes of the other kingdom. These results suggest that certain bacterial taxa may strongly affect fungal community assembly, and vice versa.

To interpret these patterns biologically, we expanded the Discussion to highlight potential ecological mechanisms underlying such cross-kingdom effects. For example, the candidate keystone bacterium *Collimonas*, identified in the fungal assembly landscape analysis (Fig. 4b and Supplementary Fig. S19), has been reported to extract nutrients from living fungal hyphae, including both arbuscular and

ectomycorrhizal fungi, through mycoparasitic activity. Conversely, certain endophytic fungi, such as *Meliniomyces* and *Hyaloscypha* (Figs. 4d, 6 and Supplementary Fig. S21), may influence microbiome assembly dynamics by modulating host–prokaryote feedback through their diverse extracellular metabolites.

(lines 403–413 in the main text)

> "Although careful consideration of potential confounding factors is required, cross-kingdom interactions between fungi and prokaryotes likely act as key determinants of both fungal and prokaryotic community assembly[67,68]. Interestingly, the candidate keystone bacterium *Collimonas*, highlighted in the fungal assembly landscape analysis (Fig. 4b and Supplementary Fig. S19), has been reported to extract nutrients from living fungal hyphae, including both arbuscular and ectomycorrhizal ones, through mycoparasitic activity[35]. Conversely, certain endophytic fungi, such as *Meliniomyces* and *Hyaloscypha* (Figs. 4d, 6 and Supplementary Fig. S21), may also influence microbiome assembly dynamics by modulating host–prokaryote positive feedback via their diverse extracellular metabolites[69,70] (e.g., amino acids, peptides, and lipids). Therefore, these fungi and prokaryotes could interact metabolically and jointly shape the overall dynamics of the root microbiome."

REPLY TO REVIEWERS

*** Reviewer comments are followed by our RESPONSEs. Line numbers in the revised manuscript are indicated for each revised point. ***

-- Reviewer 1--

I thank the authors for their careful, detailed, and comprehensive response to my comments. They have addressed all of my concerns satisfactorily through substantial revisions, additional analyses, clearer methodological explanations, and improved presentation of the manuscript. As a result, the quality, clarity, and overall rigor of the paper have been greatly improved. I am satisfied with the revised version and have no further substantial comments.

[Response] We sincerely thank the reviewer for the constructive comments and careful evaluation of our previous revision. We are grateful that the revisions have satisfactorily addressed the reviewer's concerns.

-- Reviewer 2--

I appreciate the authors' careful and detailed responses to my comments. The revisions have substantially improved the clarity, structure, and presentation of the manuscript, including a clearer explanation of the theoretical framework. All of my concerns have been adequately addressed, and I am happy to recommend acceptance of the manuscript without further changes.

[Response] We sincerely thank the reviewer for the constructive comments and careful evaluation of our previous revision. We are grateful for the reviewer's positive assessment of the revised manuscript.

-- Reviewer 4--

The authors have incorporated substantial new analyses in the revised manuscript, including validation with the gLV model and additional analyses using independent datasets. These efforts further broaden the scope of the study and have the potential to deepen our understanding of the proposed energy landscape framework and the keystone concept introduced by the authors. However, the newly added results are not sufficiently integrated with the original narrative of the manuscript and are only briefly described. More importantly, the implementation of the gLV model appear to be potentially problematic and requires further clarification from

the authors. Please see the detailed comments below.

[Response] We thank the reviewer for these constructive comments and for acknowledging the potential value of the additional analyses incorporated into the revised manuscript. Following the reviewer's suggestion, we revised the manuscript and added some analyses to better integrate the newly added analyses with the main narrative of the study. We also addressed the reviewer's concerns regarding the gLV model by revising the relevant descriptions and adding further analyses where appropriate. The details of these revisions are provided in our point-by-point responses below.

1. The authors conducted in silico simulations based on generalized Lotka–Volterra (gLV) model. As shown in Supplementary Fig. S6, the model appears to operate in a chaotic regime, which may result from the introduction of random noise. This differs from some previous studies that assume microbial communities typically reside near a steady state. In addition, the authors statistically infer community assembly patterns by sampling the system at random time points during the simulations. However, if the concept of a community basin is intended to correspond to a steady-state attractor, it is unclear why random sampling from a chaotic trajectory would be appropriate for predicting the basin structure. The authors should clarify the rationale for this approach and its ecological interpretation.

[Response] We thank the reviewer for raising this important point regarding the role of stochasticity in our gLV simulations and the rationale for sampling community compositions at random time points.

Our aim is to identify candidate keystone microbes from observational microbiome data, which are influenced by immigration/emigration and disturbances (e.g., rainfall). It is crucial to evaluate how such stochastic influences affect our inference. In Supplementary Figs. S6–S8, we generated datasets with stochastic perturbations introduced at each Euler integration step. This motivation has been clarified in the Methods (lines 638–645 in the main text), specifically:

> "Furthermore, to assess the performance of assembly landscape inference from observational data influenced by immigration, emigration, and external disturbances, stochastic perturbations were introduced at each Euler integration step as
>
> $$\epsilon_i(t) = \eta_i(t)\, \xi_i(t) \qquad \text{[eq. 10]},$$
>
> where $\eta_i(t) \sim \mathcal{N}(0, 0.01)$ represents small symmetric fluctuations (mean = 0; standard deviation = 0.01), and $\xi_i(t) \sim \mathrm{LogNormal}(0, 2)$ introduces positively skewed multiplicative variability in perturbation magnitude."

We also clarify the ecological interpretation of "basins" and the appropriateness of random sampling under non-equilibrium or perturbed dynamics. In our assembly landscape framework, "basins" refer to operationally defined regions of high occupancy, or high probability, in community-state space. Thus, basins are interpreted as high-probability zones rather than strict steady-state attractors (Fig. 1a; lines 71–74 in main text). This interpretation is important here because it supports the use of random sampling along trajectories to approximate the occupancy distribution across community states, that is, the empirical probability distribution resulting from the dynamics, rather than solely identifying terminal attractors.

To reflect cross-sectional observational sampling, we sampled one time point uniformly at random from each simulation replicate and treated it as a single community composition. This strategy captures both frequently observed compositions near stable regions and low-frequency transitional compositions, consistent with real datasets in which communities are not necessarily observed after convergence. We made these rationales explicit by revising the main text (lines 683–691) to:

> "Because natural communities are not always observed at a converged state, community compositions during transitional phases or other non-equilibrium dynamics may also appear in observational datasets. To reflect cross-sectional sampling and to reconstruct assembly landscapes over a broad state space, we drew one time point uniformly at random from each independent simulation replicate, yielding one community composition per replicate. Because basins in an assembly landscape are defined as high-probability regions of the state-occupancy distribution rather than strict dynamical attractors, this strategy captures both frequently visited compositions near stable regions and low-frequency compositions arising from transitional phases or other non-equilibrium dynamics."

Finally, to assess whether our conclusions depend on stochasticity or the sampling scheme, we added two supplementary gLV-based validations (Supplementary Figs. S9–S10) and described them in the main text (lines 756–775) and Supplementary Information (lines 393–429). First, we removed the stochastic perturbation term and repeated the entire benchmark pipeline for the same selected model systems. Under this deterministic setting, the correspondence between *Δtopography* and the community dissimilarity induced by focal-species removal remained significant in 14 of the 15 parameter sets (Supplementary Fig. S9), suggesting that the association is largely maintained even in the absence of perturbation noise. Second, we analyzed systems with perturbation-driven switching among alternative quasi-stable states, excluding the initial transient. Under this setting, *Δtopography* identified taxa whose removal caused relatively large shifts in community composition, although its system-wide correlation with removal-induced dissimilarity was more limited (Supplementary Fig. S10). Together, these analyses indicate that *Δtopography* can remain informative across different dynamical settings, while also clarifying conditions under which its performance is stronger or more limited.

Using the data generated by the gLV model, the authors also benchmarked the number of identified basins (Supplementary Fig. S48a). However, this was not compared with the number of attractors that the underlying gLV system may possess. For instance, how many potential basins (attractors) would emerge if community compositions were randomly initialized in the gLV simulations?

[Response] As suggested, we evaluated how many terminal community states, corresponding to putative attractor-like outcomes of the underlying gLV systems, could be reached from randomly initialized community compositions. Specifically, for each gLV parameter set used in Supplementary Figure S48, we simulated community dynamics from 100 independently generated initial conditions and summarized the terminal community composition as the mean abundance vector over $t = 95–100$. We then clustered these terminal community compositions to estimate the number of discrete terminal states. To account for uncertainty in the optimal number of clusters estimated by the silhouette method, we repeated this procedure 15 times for each parameter set.

This additional analysis showed that the number of basins inferred by the energy landscape analysis was broadly consistent with the estimated number of discrete terminal community-state clusters, although distinguishing between one and two clusters remained difficult because of the methodological limitations of clustering and silhouette-based model selection. We have added this analysis to the revised manuscript and now present the results in Supplementary Figure S50a. We have also added and revised the Methods and Supplementary Results accordingly (lines 659–678 in the main text and lines 359–368 in the Supplementary Results).

(lines 659–678 in main text)

> "For each community size setting (S = 20, 30, 40, or 50), three independent parameter sets were generated. For each parameter set, we first evaluated the number of discrete terminal community states that could be reached from randomly initialized community compositions. To this end, we performed simulations from 100 independently generated initial abundance vectors and defined the terminal community composition of each simulation as the mean abundance vector over $t = 95–100$. This procedure was repeated 15 times for each parameter set to account for uncertainty in the estimated number of clusters. Each simulation was run using the first-order Euler method ($\Delta t = 10^{-3}$) until $t = 100$, which was sufficient to allow initial transient dynamics to decay in most simulations. The simulated abundance data were then converted into binarized community-state matrices required for energy landscape analysis. To determine an appropriate binarization threshold, we evaluated 30 candidate thresholds evenly spaced between the 10% and 90% abundance quantiles. For each threshold, Bray–Curtis dissimilarities calculated from abundance data and Jaccard dissimilarities calculated from binarized data were compared. The threshold maximizing Kendall's rank correlation coefficient between Bray–Curtis and Jaccard dissimilarities was selected so that binarization preserved the underlying community structure (Supplementary Fig. S6a). The resulting binarized terminal community states were subjected to hierarchical clustering based on Jaccard

dissimilarity using Ward's method. The number of clusters varied from 2 to 10, and the optimal number of clusters was selected using the silhouette method (Supplementary Fig. S50a). These clusters were used as an approximation of the number of discrete terminal community states, or putative attractor-like outcomes, that could arise under each gLV parameter set."

(lines 359–368 in the Supplementary Results)

"We also compared the number of basins inferred by energy landscape analysis with the number of distinct final community-composition groups observed in randomly initialized gLV simulations.

When the number of community samples was 300 or fewer, the number of detected basins varied among replicate datasets and, in some cases, differed from the number of terminal community-state clusters, depending on community complexity (i.e., species richness) and the gLV parameter settings (Supplementary Fig. S50a–b). In contrast, when the number of community samples exceeded 300, variation in the number of detected basins was reduced, and the detected number of basins often became closer to the number of distinct final community-composition groups observed in randomly initialized simulations (Supplementary Fig. S50a–b)."

Furthermore, how do properties of the interaction network, such as connectivity or mean interaction strength, influence the number of basins in the system? Clarifying these points would help relate the statistically inferred basins to the ecological dynamics implied by the model and provide a clearer ecological interpretation of the framework.

[Response] Following the reviewer's suggestion, we performed an additional analysis to examine whether global properties of the gLV interaction network are associated with the number of basins inferred by the energy landscape analysis. Specifically, we analyzed the 15 binarized community datasets generated from the gLV parameter sets used in the benchmark analysis shown in Supplementary Figures S6–S8. For each dataset, we reconstructed the energy landscape and estimated the number of basins using the steepest-descent procedure. We then tested the relationships between the inferred number of basins and two global properties of the corresponding interaction matrix: connectance, corresponding to network connectivity, and mean interaction strength.

This additional analysis did not detect a significant association between the number of inferred basins and either of these interaction-network properties. Thus, under the parameter settings examined here, the basin structure reconstructed by our assembly landscape framework was not explained by connectance or mean interaction strength alone. We interpret this result as indicating that these basic interaction-network properties were insufficient to account for variation in basin number in our benchmark systems, rather than as evidence

for a general absence of such relationships between network architecture and basin number across all gLV models. We agree that linking statistically inferred basins to ecological dynamics is an important issue, and we now clarify that the inferred basins are related to, but not necessarily identical to, deterministic attractors of the underlying gLV system. We also note that basin structure may depend on more detailed features of the interaction matrix, nonlinear dynamics, stochastic perturbations, extinction/immigration processes, and the sampling scheme. We have added the following description to the Methods section and Supplementary Results to clarify the analysis and its ecological interpretation.

(lines 777–789 in main text)

"**Benchmark analysis: interaction properties and basin number**

To examine the relationship between interspecific interaction networks and the structure of the reconstructed assembly landscapes, we analyzed the 15 binarized community datasets generated from the gLV parameter sets used in the keystone-exploration benchmark analysis. For each dataset, we performed energy landscape analysis and estimated the number of basins using a steepest-descent procedure initiated from 150,000 randomly generated community states. For each corresponding gLV parameter set, we calculated two global properties of the interaction matrix: connectance levels (0.2, 0.3, and 0.4) and mean interaction strength, defined as the mean absolute value of nonzero off-diagonal interaction coefficients in the interaction matrix (Supplementary Fig. S51). We evaluated whether the number of inferred basins differed among connectance levels using pairwise Wilcoxon rank-sum tests, with false discovery rate correction using the Benjamini–Hochberg procedure[79]. The relationship between the number of inferred basins and mean interaction strength was assessed using Kendall's rank correlation test."

(lines 431–453 in Supplementary information)

"**Benchmark analysis: interaction properties and inferred basin number**

Previous theoretical studies have suggested that properties of interspecific interaction networks can influence the number and stability of alternative attractors in ecological dynamical systems[22]. However, quantitatively predicting the number of attractors from the interaction matrix remains difficult for general gLV systems[23]. Moreover, basins inferred in our assembly landscape framework are operationally defined as high-probability regions in the reconstructed state-occupancy landscape, and therefore are related to, but not necessarily identical to, deterministic attractors of the underlying gLV model.

To examine how global properties of the interaction network are associated with basin structure in the reconstructed assembly landscapes, we analyzed the 15 gLV parameter sets used in Supplementary Figs. S6–S8. For each parameter set, we reconstructed the energy landscape from the corresponding binarized community matrix and estimated the number of basins using a steepest-descent procedure

initiated from 150,000 randomly generated community states. We then tested whether the inferred number of basins was associated with interaction-network connectance or mean interaction strength.

We did not detect a significant difference in the number of inferred basins among the three connectance levels used in the benchmark analysis: $C = 0.2,\ 0.3$, and 0.4 (Supplementary Fig. S51a). In addition, mean interaction strength was not significantly correlated with the number of inferred basins (Kendall's rank correlation coefficient: $\tau = -9.66 \times 10^{-3},\ P > 0.05$; Supplementary Fig. S51b). These results suggest that, under the parameter settings examined here, the number of basins inferred by the assembly landscape analysis was not explained by connectance or mean interaction strength alone. Instead, basin structure may depend on more detailed features of the interaction network, nonlinear community dynamics, stochastic perturbations, extinction/immigration processes, and the sampling scheme."

2. The authors have added a discussion of the broader literature on keystone species and keystone taxa in microbial communities in the Discussion section. However, I still feel that the term "keystone" should be used with caution. Traditionally, a keystone species refers to a species whose removal has a disproportionately large impact on community structure or ecosystem function. If the authors intend to identify keystone taxa in this traditional sense using Δ topography and Δ evenness derived from the assembly landscape framework, they should provide evidence that the taxa identified by their method indeed exert such ecological impacts. Alternatively, if the authors intend to extend or reinterpret the traditional definition of "keystone" within the context of the assembly landscape framework, the rationale and necessity for this conceptual shift should be clearly explained. This point should be clarified in the Introduction to better illustrate the authors' motivation for developing this framework.

[Response] We appreciate the reviewer's concern and agree that the classical keystone concept is grounded in disproportionate effects demonstrated under taxon-specific removal or perturbation. Our central contribution is a conceptual and methodological advance that adapts this idea to observational microbiome data, where direct experimental validation is often impractical. We do not claim that the taxa identified here are experimentally validated keystone species in the classical removal-based sense. Instead, we extend the keystone concept by shifting the focus from removal-validated effects that are rarely feasible for diverse microbiomes to a landscape-based notion of ecological importance, namely taxa that are associated with major reorganization of community assembly architecture. We then operationalize this extended concept through *Δtopography* and *Δevenness*, enabling quantitative evaluation directly from observational data.

As suggested by the reviewer, we revised the first paragraph of Introduction to state this motivation and conceptual shift explicitly (lines 39–51 in the main text):

(lines 39–51 in the main text)

"Fungi and bacteria associated with plant roots support host survival, nutrient acquisition, and ecosystem functioning in terrestrial environments[1–4]. Numerous studies have suggested that such plant microbiomes

may harbor "keystone" taxa exerting strong influences on community structure and ecosystem processes[5–10]. However, identifying such taxa remains difficult under traditional definitions of keystone taxa, which emphasize disproportionate impacts relative to abundance and typically require targeted perturbations of specific taxa for empirical validation[9,10]. Many plant-associated microbes depend strongly on host-derived carbon and are therefore difficult to examine using culture-based experiments[11]. Even when culturing is feasible, demonstrating ecosystem-scale impacts would generally require large spatial-scale manipulations conducted over long temporal scales. Conducting such experiments for thousands of microbial taxa present in natural communities is therefore impractical. Consequently, data-driven analytical frameworks that translate keystone concepts into operational criteria for inferring microbial roles from observational data are essential for advancing our understanding of below-ground ecosystem processes."

This revision makes explicit that we do not use *Δtopography* and *Δevenness* as direct experimental evidence for classical keystone effects. Rather, these indices provide an operational, landscape-based framework inspired by the classical keystone concept, designed to prioritize candidate taxa whose abundance gradients are associated with major reorganization of microbiome assembly landscapes.

3. The authors added PCoA analyses for the fungal and prokaryotic community datasets based on family-level compositions using Bray–Curtis and Jaccard dissimilarities (Supplementary Fig. S12). However, these results are not sufficiently integrated into the main text. Although Fig. S12 is cited at lines 157 and 181, no explanation or interpretation is provided. Consequently, it is unclear how this supplementary analysis supports or strengthens the main arguments of the manuscript. The authors should clarify whether the basins defined in their statistical model are intended to correspond, at least conceptually, to the ecological assembly patterns reflected in the PCoA ordination. Clarifying this relationship would help bridge the statistical framework and the ecological interpretation of the results.

[Response] As suggested, we revised the main text to explicitly describe and interpret the PCoA results and to clarify how they support the basin structures inferred from the assembly landscape framework. Specifically, we added a new paragraph in the Results section to explain how the ordination patterns relate to the basin-based representation of community states as follows (lines 192–206 in the main text). We also documented the full procedures for the ordination and permutation-based tests in a new Supplementary Methods subsection, "Relationships between community structures and assembly landscape architectures" (lines 98–115 in the Supplementary Information).

(lines 192–206 in the main text)

"To evaluate how basin structures inferred from the assembly landscape framework relate to observed community composition, we performed principal coordinate analysis (PCoA) based on family-level dissimilarities (Supplementary Fig. S14). Differences in abundance-based community composition associated with basin identity were statistically detectable (PerMANOVA; Supplementary Table S1).

> However, the separations in ordination space were not visually pronounced (Supplementary Fig. S14a, c), likely because several basin-indicative families occurred at low relative abundances, including Glomeraceae (arbuscular mycorrhizal fungi; relative abundance = 2.01×10^{-2}) and Nocardioidaceae (Actinomycetota bacteria; relative abundance = 5.87×10^{-4}; see Figs. 2c and 3c), thereby contributing little to abundance-weighted dissimilarity patterns. In contrast, clearer separations were observed under Jaccard dissimilarities based on presence–absence profiles (Supplementary Fig. S14b, d; Supplementary Table S1), as expected from the presence–absence definition of community states in the assembly landscape analysis. Thus, this comparison helps clarify that the inferred basin structures primarily reflect differences in taxon membership, including low-abundance but ecologically informative families, rather than abundance variation among dominant taxa."

4. The authors state that "Notably, genera showing relatively strong effects on these indices were not necessarily those that were highly prevalent or abundant in the root microbiome dataset (Supplementary Figs. S15–S18)" (lines 201–203). However, inspection of Figs. S15b and S16b suggests that there may be some degree of association between the abundance or occupancy of prokaryotic taxa and the assembly landscape metrics (Δ topography and Δ evenness). While formal statistical testing would be required to evaluate this relationship, the current evidence does not appear sufficient to conclude that these variables are unrelated. The authors should clarify this point or provide additional analyses to support their statement.

[Response] Our intention in the original text was not to claim that abundance/occupancy is unrelated to the assembly landscape metrics, but rather to emphasize that genera highlighted as keystone candidates (i.e., those showing particularly large effects on the metrics) are not restricted to the most abundant or prevalent taxa. We agree, however, that our previous wording could be interpreted as implying no overall relationship, and we therefore revised the manuscript for clarity.

Following the reviewer's suggestion, we conducted formal correlation analyses between taxon abundance and/or occupancy, and the landscape metrics shown in Supplementary Figs. S15–S18. We added the statistical results as Supplementary Tables S3–S4 and revised the corresponding text in the Results section (lines 219–224 in the main text) as follows:

> "Although taxon abundance and/or occupancy were significantly associated with a subset of landscape metrics across genera (Kendall's rank correlation test; Supplementary Tables S3–S4), many genera highlighted as having large effects showed intermediate, rather than particularly high, abundance and occupancy (Supplementary Figs. S17–S20). These results indicate that abundance and occupancy alone do not determine which taxa contribute the largest effects."

5. The section headings in the Results are largely descriptive and do not clearly highlight the main conclusions of each section. The authors may consider revising some of the section titles to better reflect the main

findings, which would help readers get the key results more quickly.

[Response] Following this suggestion, we revised some Results subheadings to highlight the main findings of each section rather than descriptive labels. The revised subheadings are:

"**Assembly landscapes enable keystone exploration**" (line 112 in the main text)

"**Distinct basin groups recur in assembly landscapes**" (line 149 in the main text)

"**Endophytic fungi emerge as candidate keystones**" (line 208 in the main text)

"**Key bacteria consistently reshape prokaryotic landscapes**" (line 249 in the main text)

"**Candidate keystones are associated with basin turnover**" (line 270 in the main text)

"**Candidate keystones reflect mycorrhizal colonization**" (line 301 in the main text)

To maintain consistency with these revisions, we also revised the following Methods subheading:

"**Candidate keystones and mycorrhizal states in Europe**" (line 791 in the main text)

6. In the Figure 1 panel g, please use median abundance instead of 50% abundance-quantile.

[Response] Following this reviewer's suggestion, we clarified the notation in Figure 1g by explicitly indicating that the 50% quantile corresponds to the median. We also applied the same clarification to Supplementary Figures S6 and S11, where similar expressions were used.

REPLY TO A REVIEWER

*** A reviewer comment is followed by our RESPONSE. ***

-- Reviewer 4---------------------------------------

I appreciate the authors'careful and detailed responses to my comments. The revisions, including the additional analyses, clearer methodological explanations, and improved presentation, have substantially enhanced the quality, clarity, and overall rigor of the manuscript. I am satisfied with the revised version and have no further substantive comments. I believe the manuscript is now suitable for publication.

[Response] We sincerely thank the reviewer for the constructive comments and careful evaluation of our previous revision. We are grateful that the revisions have satisfactorily addressed the reviewer’s concerns.
